# Data-driven design of new chiral carboxylic acid for construction of indoles with *C*-central and C–N axial chirality via cobalt catalysis

**DOI:** 10.1038/s41467-023-38872-0

**Published:** 2023-05-31

**Authors:** Zi-Jing Zhang, Shu-Wen Li, João C. A. Oliveira, Yanjun Li, Xinran Chen, Shuo-Qing Zhang, Li-Cheng Xu, Torben Rogge, Xin Hong, Lutz Ackermann

**Affiliations:** 1 Institut für Organische und Biomolekulare Chemie, Georg-August-Universität Göttingen, Tammannstraße 2, 37077 Göttingen, Germany; 2 Center of Chemistry for Frontier Technologies, Department of Chemistry, State Key Laboratory of Clean Energy Utilization, Zhejiang University, Hangzhou, 310027 PR China; 3 Beijing National Laboratory for Molecular Sciences, Zhongguancun North First Street No. 2, Beijing, 100190 PR China; 4 Key Laboratory of Precise Synthesis of Functional Molecules of Zhejiang Province, School of Science, Westlake University, 18 Shilongshan Road, Hangzhou, 310024 Zhejiang Province PR China; 5 Wöhler Research Institute for Sustainable Chemistry (WISCh), Georg-August-Universität Göttingen, Tammannstraße 2, 37077 Göttingen, Germany

**Keywords:** Asymmetric catalysis, Synthetic chemistry methodology

## Abstract

Challenging enantio- and diastereoselective cobalt-catalyzed C–H alkylation has been realized by an innovative data-driven knowledge transfer strategy. Harnessing the statistics of a related transformation as the knowledge source, the designed machine learning (ML) model took advantage of delta learning and enabled accurate and extrapolative enantioselectivity predictions. Powered by the knowledge transfer model, the virtual screening of a broad scope of 360 chiral carboxylic acids led to the discovery of a new catalyst featuring an intriguing furyl moiety. Further experiments verified that the predicted chiral carboxylic acid can achieve excellent stereochemical control for the target C–H alkylation, which supported the expedient synthesis for a large library of substituted indoles with *C*-central and C–N axial chirality. The reported machine learning approach provides a powerful data engine to accelerate the discovery of molecular catalysis by harnessing the hidden value of the available structure-performance statistics.

## Abstract (web summary)

The design of efficient and selective catalysts is a formidable challenge in chemical science. Here the authors design a data-driven workflow to achieve the digitalized knowledge transfer between the synthetically relevant transformations, which was demonstrated in the prediction of chiral carboxylic acid co-catalyst for the cobalt-catalyzed asymmetric C–H alkylation of indoles.

## Introduction

**Fig. 1:**
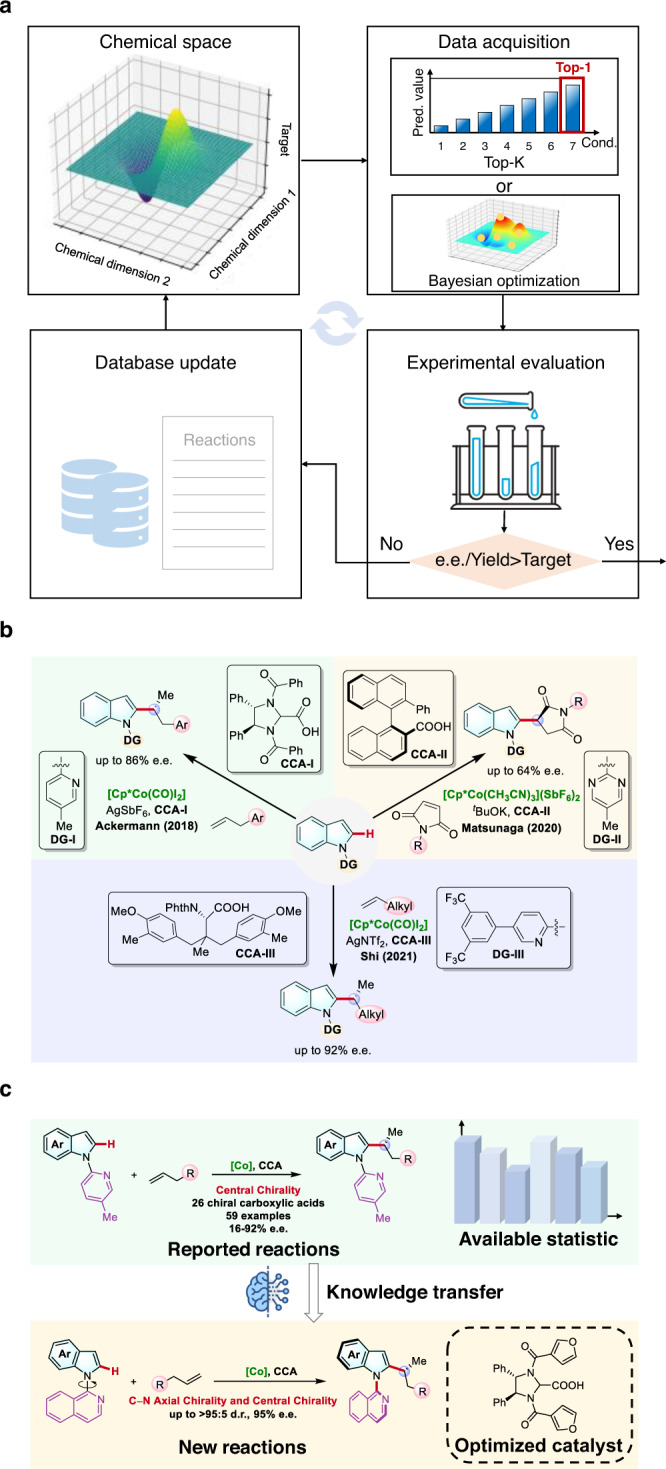
Data-driven discovery of molecular catalysis. **a** General workflow of current machine learning-assisted reaction optimization. **b** Cp*Co(III)/CCA-catalyzed asymmetric C−H functionalization of indoles. **c** Designed knowledge transfer model for predicting new CCAs of asymmetric C−H functionalization of indoles.

The design of efficient and selective catalysts is a formidable challenge in chemical science. Because of the magnificent molecular universe and the transformation-dependent catalysis property, the complexity of the structure-performance relationship (SPR) in molecular catalysis is beyond imagination. As a revolutionary change to the classic experience-driven strategy of catalyst development, machine learning (ML) has recently emerged as a powerful approach for exploring the high-dimensional SPR^1,2^. A series of breakthroughs have realized the accurate and efficient ML predictions of new catalysts and transformations^3–7^, Fig. 1a highlights the general workflow of the current data-driven exploration of chemical space. Relying on the statistics of the target catalysis, ML is able to create an SPR model, which drives the subsequent data acquisition. This data acquisition is essentially an optimization problem, and greedy search^8^ (Top-*k* method) or Bayesian optimization^9^ are the representative engines for providing the candidate reaction designs. Experimental evaluations of these ML designs offer new data sources to improve the ML model, which completes a feedback loop until the target synthetic performance is achieved. This process, in principle, does not involve human intervention and can be accelerated by automatic synthesis. Landmark studies by Cronin^8^, Cooper^10^, Doyle^9^, Jensen^11^, Denmark^3^ and others^12,13^ have highlighted that this data-driven workflow can discover powerful catalysis conditions starting from zero knowledge of the target transformation.

Despite the remarkable success of ML-assisted reaction optimizations, it should be noted that this logic of optimization starting from zero knowledge or data is fundamentally different as compared to the way that human chemists are typically practicing. It is extremely rare to design a catalyst that has absolutely no related knowledge available. In the typical scenario, the chemist’s catalyst design is based on the careful evaluation of related SPR data and the judicious chemical innovation of a given compound^14–16^. This is essentially a knowledge transfer process where the explored chemical space facilitated the rational expansion of the known SPR to new catalyst. In recent years, the concept of knowledge transfer has also been applied to the data-driven modeling in synthetic chemistry, which has shown great potential in addressing the problem of limited sample size. By leveraging innovative modeling strategies, knowledge transfer modeling can connect chemically related data and reduce the data demand for target domain. For example, through the unsupervised ML that increases the model’s differentiation ability of phosphine ligands, Schoenebeck and co-workers^17^ were able to achieve the successful prediction of dinuclear palladium catalyst with only five labeled data. We recently developed a hierarchical learning approach which can select appropriate datasets for layered modeling based on the proximity in chemical space, thereby improving the predictive performance of the ML model^18,19^. These knowledge transfer models not only improve the efficiency of catalyst design but also help expand the known chemical space in a data-driven fashion. Therefore, the integration of knowledge transfer modeling into data-driven synthetic discovery is of great significance for advancing the field of catalysis and beyond.

Over the last years, cobalt-catalyzed asymmetric C–H functionalization has garnered significant attention^20,21^. The groups of Yoshikai^22–24^, Dong^25^, Lautens^26^, Yang^27^, Wencel-Delord^28^, and Shi^29^ have successively combined low-valent cobalt catalysts with chiral ligands to achieve stereoselective C–H functionalization, while Cramer and co-workers developed chiral Cp^x^Co(III) complexes for this purpose^30–32^. In addition, achiral Cp*Co(III)/chiral carboxylic acid (CCA) systems^33–41^ have also been widely deployed to catalyze asymmetric C–H alkylation of indoles^39–41^ (Fig. 1b). However, the use of synthetically demanding chiral acids requires laborious multi-step synthesis, limiting the potential of these transformations^33–40^. In 2018, Ackermann and coworkers achieved the enantioselective cobalt-catalyzed C–H alkylation by a designed C2-symmetric CCA that can be easily synthesized^41^. This CCA-based chiral catalysis serves as a powerful platform^41,42^, and the engineering of the CCA structure is of great potential for the enrichment of asymmetric derivatization of indoles.

Axial chirality is of major importance for modern pharmaceutical industry^43,44^, and synthetically challenging C–N axially chiral indoles are privileged motifs in drug design, crop protection, and material science^45,46^. Thus, the efficient synthesis of these compounds has become a rapidly expanding field^47,48^. However previous studies mainly relied on the use of noble 4d and 5d transition metal catalysts^49–52^, while sustainable 3d-metal-catalyzed transformation^53,54^ remains underdeveloped. Therefore, the development of an efficient CCA co-catalyst for cobalt-catalyzed C–H activation to enable the assembly of atropisomeric compounds bearing C–N axial chirality, and simultaneously construct *C*-centered chirality with high stereoselectivity is a tremendously important and unrealized challenge.

In light of the critical knowledge transfer for catalyst development, we envisioned that the digitalization of the knowledge transfer process can serve as an innovative data-driven strategy for catalyst design. This requires the ML model to capture the key differences between the given transformation and the target reaction, so that the available statistics of the given transformation can serve as a knowledge source and guide the design of the target reaction.

Herein we report the development of a data-driven transfer learning workflow to achieve the ML prediction of catalytic performance using related synthetic data (Fig. 1c). Demonstrated in the discovery of new CCA catalyst, our ML model provided a powerful CCA prediction that realized the challenging enantio- and diastereoselective C–H alkylation of indoles utilizing earth-abundant cobalt catalyst. The ML-predicted CCA catalyst enabled the target transformation that can simultaneously control both the *C*-centered and the C–N axial chirality, providing the atropisomeric indoles with excellent diastereo- and enantioselectivities (Fig. 1c). This work offered a paradigm-shifting tool for the discovery of molecular catalyst, which is expected to serve as a powerful data engine to support the innovation of catalysis science.

## Results and discussions

### Design of knowledge transfer model

**Fig. 2:**
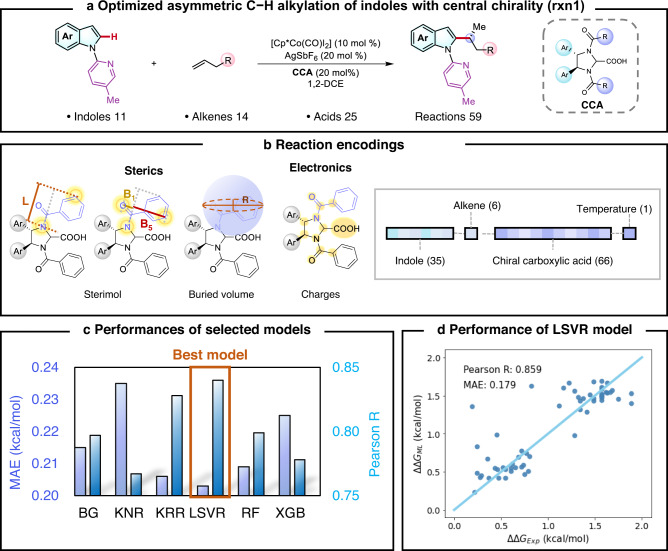
Machine learning enantioselectivity prediction for the Cp*Co(III)/CCA-catalyzed C–H alkylation of indoles with central chirality. **a** Overview of accumulated data of Cp*Co(III)/CCA-catalyzed asymmetric C−H alkylation of indoles with central chirality. **b** Reaction encodings and highlighted descriptors of steric and electronic effect using CCA as an example. **c** Regression performances of various algorithms in 10-fold cross validation. **d** Performance of enantioselectivity predictions using LSVR model. Source data are presented in the *Source_Data*.

To achieve the desired knowledge transfer, the first step is to create a reliable SPR model using the available statistics of the optimized transformation (Fig. 2). The already optimized Cp*Co(III)/CCA-catalyzed asymmetric C−H alkylation of indoles (**rxn1**) does not involve the control of the axial chirality, which was previously discovered by the Ackermann group; 59 SPR data of **rxn1** were accumulated during the catalysis screening, involving the variations of 11 indoles, 14 alkenes and 25 CCAs^41^ (Fig. 2a). The detailed data distribution is provided in the Supplementary Information (Supplementary Fig. 2). Inspired by recent data-driven selectivity prediction studies using physical organic descriptors^55,56^, we applied a series of steric (i.e. Sterimol parameters) and electronic (i.e. charge) features to describe the influence of the *N*-substituent of CCA; the entire catalysis encoding is a 108-dimensional physical organic space containing 35 descriptors for indoles, 6 descriptors for alkenes, 66 descriptors for CCAs and 1 descriptor for temperature (Fig. 2b). Based on the regression performances in the 10-fold cross-validation, linear support vector regression^57^ emerged as the most suitable algorithm with a Pearson R of 0.859 and MAE of 0.179 kcal/mol; the detailed regression results are shown in Fig. 2c, in which a nice correlation between the ML-predicted and the experimental enantioselectivities was identified. The detailed results of all tested ML models are provided in the Supplementary Information (Supplementary Table 4).

**Fig. 3:**
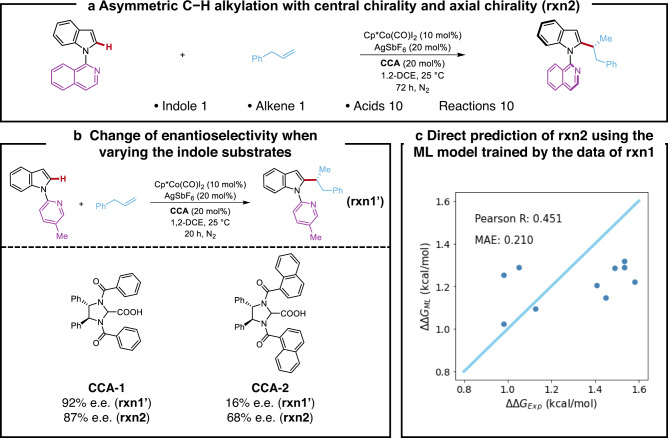
Enantioselectivity prediction of the Cp*Co(III)/CCA-catalyzed C–H alkylation of indoles with central and axial chirality using machine learning modelling without knowledge transfer. **a** Overview of the target C–H alkylation of indoles with central and axial chirality. **b** Enantioselectivity change of the Cp*Co(III)/CCA-catalyzed C–H alkylation when varying the indole substrates. **c** Enantioselectivity predictions of the target C–H alkylation of indoles with central and axial chirality using the machine learning model trained by the statistics of the C–H alkylation without axial chirality. Source data are presented in the *Source_Data*.

With the ML model of **rxn1** in hand, we tested its direct application in the target C−H alkylation with axial chirality (**rxn2**). Among the tested CCAs for **rxn1**, ten representative ones were experimentally evaluated for **rxn2** with the axial chirality challenge (Fig. 3a). The selection of representative CCAs were based on the diversity of their chemical structures and enantioselectivities. Due to the introduction of isoquinoline moiety in the indole substrate, the two transformations do not follow the exact same SPR. Figure 3b showed two highlighted examples: the optimized **CCA-1** for **rxn1'** achieved a 92% e.e. for this transformation, while its application in the atroposelective **rxn2** delivered a 87% e.e., which was one of the major motivations for the data-driven design of new CCAs; in addition, the naphthyl **CCA-2** only achieved a 16% e.e. in **rxn1'**, but the corresponding **rxn2** has the enantioselectivity of 68% e.e. This non-intuitive perturbation of SPR widely exists in molecular catalysis, which results in the unsatisfying prediction performance of the trained ML model in **rxn2**; the Pearson R is only 0.451, which is in sharp contrast to its performance in **rxn1** (Fig. 3c vs. Fig. 2d).

**Fig. 4:**
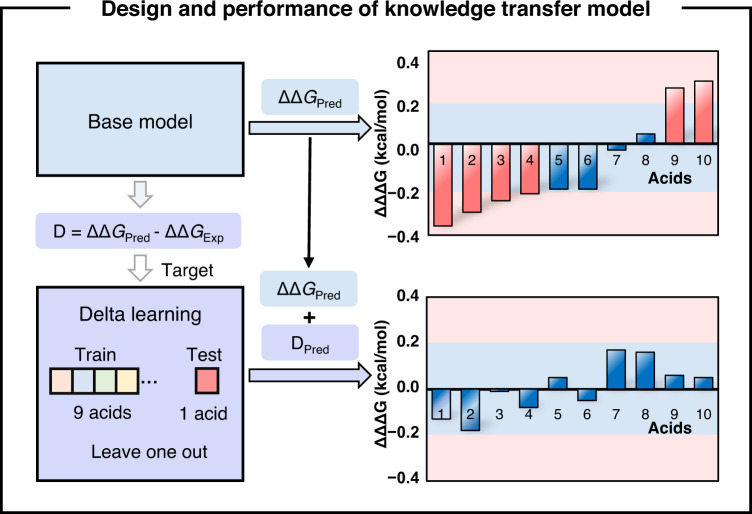
Design and performance of knowledge transfer model for making accurate predictions of the target C–H alkylation of indoles with central and axial chirality. Source data are presented in the *Source_Data*.

We next trained a delta ML model to capture the SPR perturbation, in order to correct the enantioselectivity predictions of the **rxn1** model. For the ten evaluated CCAs in **rxn2**, each CCA has the experimentally measured enantioselectivity (ΔΔ*G*_exp_) as well as the predicted value (ΔΔ*G*_pred_) from the **rxn1** model (Fig. 4). The differences between the two values (*D* = ΔΔ*G*_pred_ − ΔΔ*G*_exp_) provided a limited but valuable data source for the delta learning. Using the same physical organic encodings, the leave-one-out (LOO) training provided the delta learning model, which significantly improved the predictions of the **rxn1** model (Fig. 4); the MAE decreases from 0.210 kcal/mol to 0.095 kcal/mol, and the outliner predictions (highlighted in red) were all eliminated. Therefore, the final prediction of **rxn2** is the sum of the **rxn1** model’s prediction and the delta model’s prediction. This ML approach represents the digitalized knowledge transfer. The training of **rxn1** model harnessed the SPR from the available data of related catalysis screening, and subsequent delta learning corrected the understanding of **rxn1** using the limited data from the experimental reoptimization of **rxn2**, which mimics the logic of a human chemist.

**Fig. 5:**
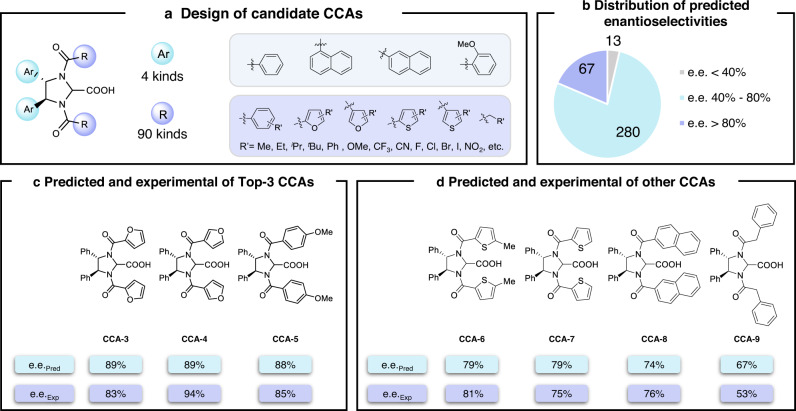
Virtual screening of highly selective CCAs using the knowledge transfer model and experimental verifications. **a** Designed structures of the 360 candidate CCAs. **b** Distribution of the predicted enantioselectivities. **c** Predicted and experimental enantioselectivities of the Top-3 CCAs. **d** Predicted and experimental enantioselectivities of CCAs with medium performances. Source data are presented in the *Source_Data*.

Using the established knowledge transfer model, we performed the virtual screening of CCAs to identify the highly selective catalyst for the atroposelective **rxn2**. Considering the synthetic access of the derivatized CCAs, 4 representative aryl substituents with different steric hindrance and electronic effects were evaluated for the CCA backbone, and a selection of 90 variations was explored for the *N*-substitution (including aromatic and heteroaromatic rings with different electronic effects and sterically hindered substituents, as well as alkyl substituents), which allowed the thorough evaluation of the CCA candidates (Fig. 5a). A few highlighted examples of the 90 substituents are provided in Fig. 5. The combination of considered substitutions together created 360 candidate C2-symmetric CCAs including the 10 CCAs that have been used in the knowledge transfer modeling, and their predicted enantioselectivities for **rxn2** are summarized in Fig. 5b. 13 out of the 360 have a predicted selectivity below 40%; 280 were predicted to have an enantioselectivity between 40 and 80%; 67 have the predicted enantioselectivity >80%. Figure 5c shows the chemical structures of the predicted Top-3 CCAs. It is interesting that the furan moiety was identified as a privileged choice of the *N*-substitution. Both the 2-furyl and the 3-furyl substituted **CCA-3** and **CCA-4** were predicted to have an 89% enantioselectivity, which ranked the first and the second of the 360 predictions. The third CCA has the *para*-OMe-phenyl substituent, whose predicted enantioselectivity was 88%. It is worth noticing that these three substitutions are all electron-rich aryl moieties with limited steric repulsions, which indicated that the chirality control may involve non-covalent interactions with the *N*-substitution. Subsequently, the predicted Top-3 CCAs were synthesized and evaluated for **rxn2**. Excellent enantioselectivities were found for all three cases, with the 3-furyl substituted **CCA-4** as the optimal catalyst. This CCA achieved a 94% enantioselectivity for **rxn2**, which highlighted the predictive power of the data-driven knowledge transfer approach. We want to emphasize that the naïve training with all the enantioselective C–H alkylation data (59 data of **rxn1** and 10 data of **rxn2**) without the usage of delta learning led to a significantly reparametrized model. The virtual screening using this reparametrized naïve model provided a reshuffled ranking, and the 3-furyl substituted **CCA-4** was predicted to have an 81% enantioselectivity with a ranking of 98, which is in sharp contrast to the outcome of the knowledge transfer model.

In order to further validate the accuracy of the model’s prediction for the entire value range and its discriminative ability for CCA’s catalytic performance, we selected a series of CCAs with medium to low predicted performances and conducted experimental synthesis and verification. Figure 5d shows the prediction and experimental results of the four tested CCAs (**CCA-6** to **CCA-9**), with a maximum error of only 14% e.e. These results further demonstrated the predictive ability of the developed knowledge transfer model, indicating that it can effectively discriminate the enantioselectivities of the candidate CCAs and uncover the useful catalysts with superior performance. To ensure the reliability of the training and prediction of the knowledge transfer model, we also evaluated the model predictions with five additional delta data. Using a total of 15 delta data to retrain the knowledge transfer model, we compared the prediction results of the seven experimentally verified CCAs (**CCA-3** to **CCA-9**) with those obtained by training with the 10 delta data. The two sets of prediction values were highly correlated (Pearson *R* = 0.961, Supplementary Fig. 8), which indicated that the additional five data had a relatively small impact on the modeling. To confirm that the success of knowledge transfer model is not accidental in substrate **1a**, we also performed the same knowledge transfer learning process on substrate **1k**; the delta learning achieved similarly effectiveness in correcting the base model’s predictions (Supplementary Fig. 10). These comparisons further validated the knowledge transfer approach, highlighting the effectiveness of the hierarchical usage of synthetic data based on chemical heuristics.

### Substrate scope for cobalt-catalyzed asymmetric C–H alkylation

**Fig. 6:**
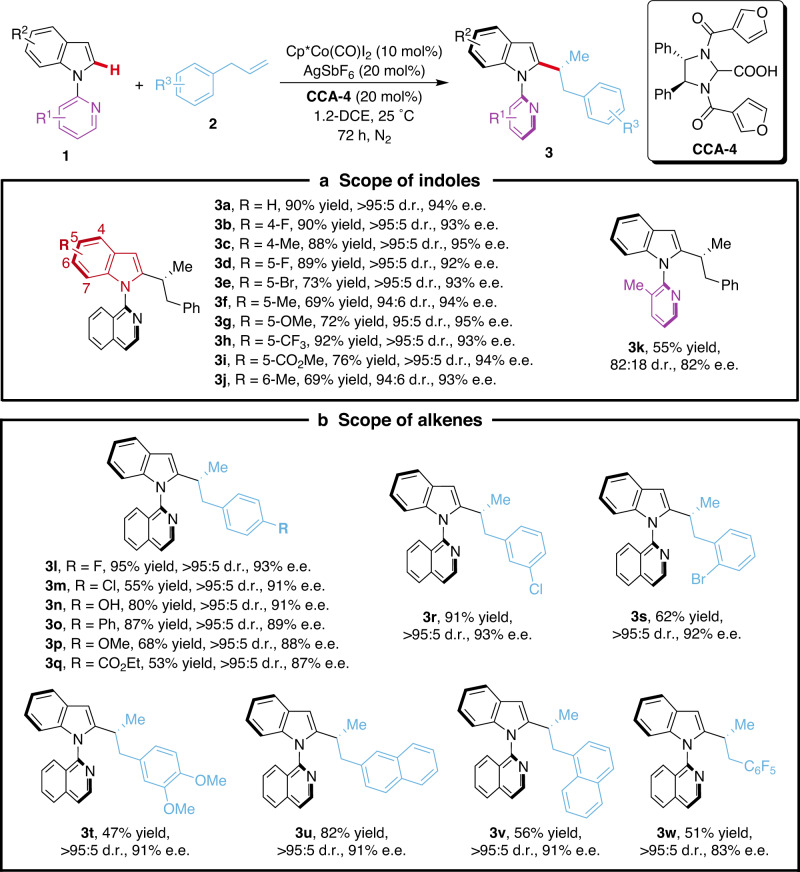
Substrate scope for asymmetric C-H alkylation. **a** Scope of indoles. **b** Scope of alkenes. Reaction conditions: 1 (0.1 mmol), 2 (0.3 mmol), Cp*Co(CO)I_2_ (10 mol%), AgSbF_6_ (20 mol%), and **CCA-4** (20 mol%) in 1,2-DCE (0.5 mL) at 25 °C for 72 h under N_2_. Unless noted, the ratio of b:l is >95:5. Yields are those of the isolated products. The diastereomeric ratio (d.r.) were determined by ^1^H NMR spectroscopy. The enantiomeric excess (e.e.) was determined by HPLC.

After locating the optimal CCA by the data-driven knowledge transfer, the substrate scope was explored under the optimized reaction conditions to delineate the potential of this transformation (Fig. 6). A variety of indole substrates were investigated (Fig. 6a). Both electron-withdrawing and electron-donating groups at the 4-, 5- or 6-position of the indole ring were tolerated to afford the desired products **3a**–**3j** in good yields with excellent diastereo- and enantioselectivities (94:6– > 95:5 d.r., 92–95% e.e.). The atropostability of the products is conserved even for the less hindered methyl-substituted product **3k**, although with a slight decrease in stereoselectivity. A broad range of alkenes bearing different substituents on *para*-, *meta*- or *ortho*-position of the arene were well tolerated and gave the desired products **3l**–**3t** in high yields and high levels of stereocontrol (all >95:5 d.r., 87–93% e.e.) (Fig. 6b). Additionally, 2-allylnaphthalene, 1-allylnaphthalene and allylpentafluorobenzene efficiently underwent the cobalt-catalysis providing the target products **3u**–**3w** with good stereoselectivities (all >95:5 d.r., up to 91% e.e.). The absolute configuration of the alkylation products was unambiguously confirmed by single-crystal X-ray diffraction analysis of **3c** and **3w**.

In conclusion, we have designed a data-driven workflow to achieve the digitalized knowledge transfer between the synthetically relevant transformations, which was demonstrated in the prediction of chiral carboxylic acid co-catalyst for the asymmetric C–H alkylation of indoles with atropselectivity challenge utilizing non-precious cobalt catalyst. Using the available catalysis screening data of a related asymmetric cobalt-catalyzed C–H alkylation, the physical organic descriptors and linear support vector regression algorithm provided a predictive machine learning model. This model serves as the knowledge base, whose predictions were further corrected using the delta learning method. The delta learning method only requires a handful of selectivity data of the target atroposelective transformation, which captures the perturbation of the structure-performance relationship between the two synthetically relevant transformations and enabled the desired data-driven knowledge transfer.

The designed data-driven knowledge transfer model enabled a powerful virtual screening of 360 candidate chiral carboxylic acids for the target atroposelective C–H alkylation of indoles. The top-3 predicted acids were synthesized and experimentally evaluated. The three predicted chiral carboxylic acids featured good to excellent experimental enantioselectivities, with the 3-furyl substituted one presenting the highest selectivity. These successful predictions and the identification of the suitable *N*-substituent provided strong support for the effectiveness of the designed knowledge transfer approach. The robustness of the enantio- and diastereoselective cobalt-catalyzed C–H alkylation promoted by the predicted chiral carboxylic acid was further explored, leading to the assembly of a large family of substituted indoles in good yields and with excellent stereoselectivities. This work provides a new data-driven strategy for knowledge transfer of synthetic chemistry. The established machine learning model was able to capture the non-intuitive perturbation of structure-performance relationship and make useful predictions in the few-shot learning scenario of synthetic optimization, which provides a powerful smart engine to accelerate the discovery of molecular catalysis.

## Methods

### General procedure for cobalt-catalyzed asymmetric C–H alkylation

To a flame-dried and N_2_-purged Schlenk tube were added indole substrate **1** (0.1 mmol), Cp*Co(CO)I_2_ (0.01 mmol, 10 mol%, 4.8 mg), AgSbF_6_ (0.02 mmol, 20 mol%, 6.9 mg), and chiral carboxylic acid **CCA-4** (0.02 mmol, 20 mol%, 9.1 mg). The vial was then sealed, purged and backfilled with N_2_ three times before adding alkene substrate **2** (0.3 mmol) and 1,2-dichloroethane (0.5 mL) at room temperature. The resulting solution was then stirred at 25 °C for 72 h. The resulting solution was diluted with dichloromethane (2.0 mL), filtered through a pad of Celite (eluted with dichloromethane), then the solvent was removed *in vacuo*. The diastereomeric ratio was determined by ^1^H NMR analysis of the crude reaction mixture. The residue was purified by column chromatography on silica gel (*n*-hexane: ethyl acetate = 15:1) to afford the desired product **3**.

## Supplementary information

Supplementary Information

## Data Availability

The data that support the findings of this study are available within the main text, the Supplementary Information and https://github.com/Shuwen-Li/FindBestChiralAcid^58^. Source data are presented in the *Source_Data*. Details about materials and methods, experimental procedures, characterization data, NMR and HPLC spectra are available in the Supplementary Information, and all other data are available from the corresponding author upon request. Crystallographic data are available free of charge under Cambridge Crystallographic Data Centre (CCDC) reference numbers 2176897 (**3c**), 2176898 (**3w**) [www.ccdc.cam.ac.uk/data_request/cif]. Source data are provided with this paper.

## Source data

Source Data

## Code availability

All codes needed to run this model are available at https://github.com/Shuwen-Li/FindBestChiralAcid^58^.
