# Supplementary material for: Data-driven design of new chiral carboxylic acid for construction of indoles with C-central and C–N axial chirality via cobalt catalysis
Source: Nat Commun. 2023 May 31;14:3149. doi: 10.1038/s41467-023-38872-0 (PMC10232535; doi:10.1038/s41467-023-38872-0)
Supplement: Supplementary file 1 — Supplementary Information [file 41467_2023_38872_MOESM1_ESM.pdf]

Supplementary Information

Data-driven design of new chiral carboxylic acid for construction of indoles with *C*-central and C–N axial chirality *via* cobalt catalysis

Zi-Jing Zhang, Shu-Wen Li, João C. A. Oliveira, Yanjun Li, Xinran Chen, Shuo-Qing Zhang, Li-Cheng Xu, Torben Rogge, Xin Hong* and Lutz Ackermann*

**Email: Lutz.Ackermann@chemie.unigoettingen.de; hxchem@zju.edu.cn.*

Table of Contents:

1. General information......2
2. Details of the dataset used in machine learning......3
3. Details of the predicted chiral carboxylic acids......6
4. Structural optimization......10
5. Details of descriptors......11
6. Details of machine learning algorithms......18
7. Details of model predictions......20
8. Comparison of the delta learning predictions using 10 and 15 data input......35
9. Additional validation in substrate **1k**......37
10. Predictions without the knowledge transfer approach......39
11. Data and code availability......42
12. Synthesis of chiral carboxylic acid......43
13. Synthesis of indole substrates......56
14. General procedure for cobalt(III)-catalyzed asymmetric C–H alkylation......63
15. Characterization data of products......63
16. X-ray single crystal data for **3c** and **3w**......95
17. NMR spectra......99
18. References......154

1. General information

General data:

NMR spectra were recorded on Bruker-300 MHz spectrometer or Bruker-400 MHz spectrometer. Chemical shifts (δ) are given in ppm relative to TMS. The residual solvent signals were used as references and the chemical shifts converted to the TMS scale ($CDCl_3$: δH = 7.26 ppm, δC = 77.16 ppm; CD_3CO_2D: δH = 11.65 ppm, δC = 178.99 ppm).

High resolution mass spectra were recorded on a Thermo LTQ Orbitrap XL (ESI+) or a P-SIMS-Gly of Bruker Daltonics Inc (EI+).

Infrared spectra were recorded on a Nicolet MX-1E FT-IR spectrometer.

Enantiomeric excesses were measured on Agilent 1290 Infinity HPLC (2487 Dual λ Absorbance Detector and 1525 Binary HPLC Pump, UV detection monitored at 250 nm). Chiralpak IA-3, IC-3 and ID-3 columns were purchased from Daicel Chemical Industries, Ltd.

Optical rotations were measured at 589 nm (sodium D line) by using a Perkin-Elmer 343 polarimeter.

Melting point ranges are measured with a Stuart Melting Point Apparatus SMP3 (Barloworld Scientific, Ltd.).

Materials:

All starting materials, reagents and solvents were purchased from commercial suppliers (Aldrich, Alfa, TCI, Daicel, etc.) and used as supplied unless otherwise stated. Chiral carboxylic acids (CCAs)[1-3] and indole substrates[4,5] were synthesized in accordance with the similar procedures in literature. 1,2-Dichloroethane was dried over CaH_2 and distilled prior to use.

2. Details of the dataset used in machine learning

2.1 Data of the asymmetric C−H alkylation of indoles with central chirality (rxn1)

59 data of the optimized asymmetric C−H alkylation of indoles with central chirality (**rxn1**) were accumulated during the catalysis screening[1], involving the variations of 11 indoles, 14 alkenes and 25 CCAs. The details of the structural variations and the number of occurrences were elaborated in Supplementary Fig. 1, and the distribution of the recorded enantioselectivities (e.e. and $\Delta\Delta G$) are shown in Supplementary Fig. 2b.

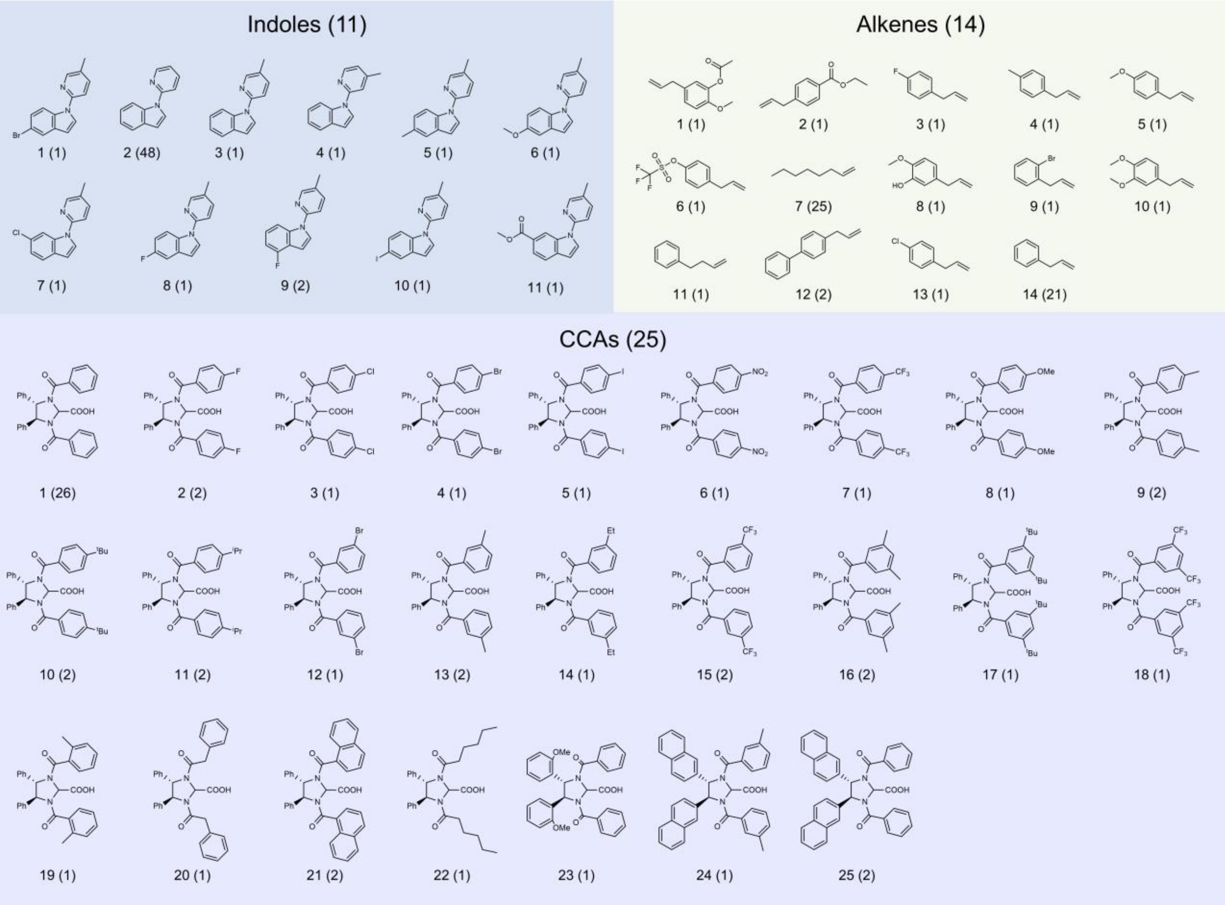

Supplementary Fig. 1 | Details of structural variations of indoles, alkenes and CCAs.

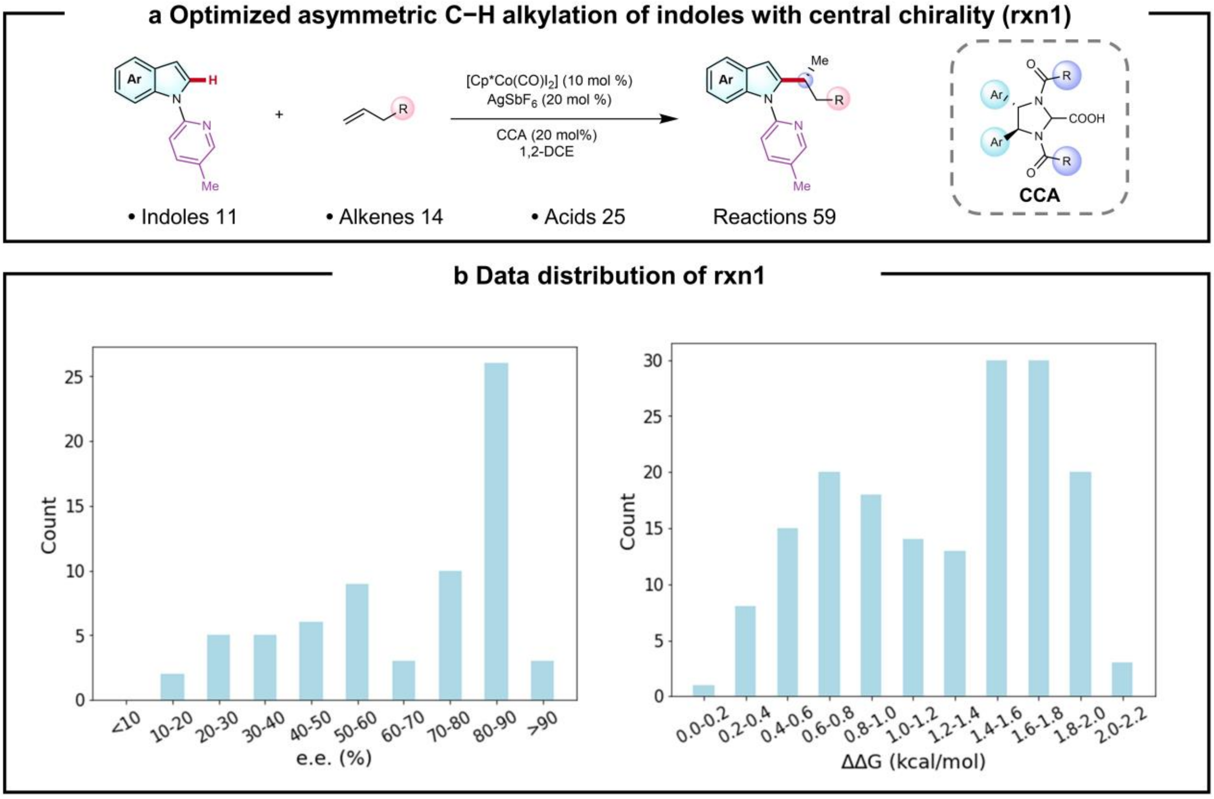

Supplementary Fig. 2 | Details of the accumulated data of the optimized asymmetric C−H alkylation of indoles with central chirality. a, The optimized asymmetric C−H alkylation of indoles with central chirality. **b**, Data distribution of the recoded enantioselectivities.

2.2 Experimental data of the asymmetric C−H alkylation of indoles

In order to evaluate the CCA-stereoselectivity relationship in **rxn2**, we examined the experimental stereoselectivities of 10 CCAs that were used in **rxn1** as well. The experimental data are shown in Supplementary Fig. 3.

Asymmetric C−H alkylation with central chirality

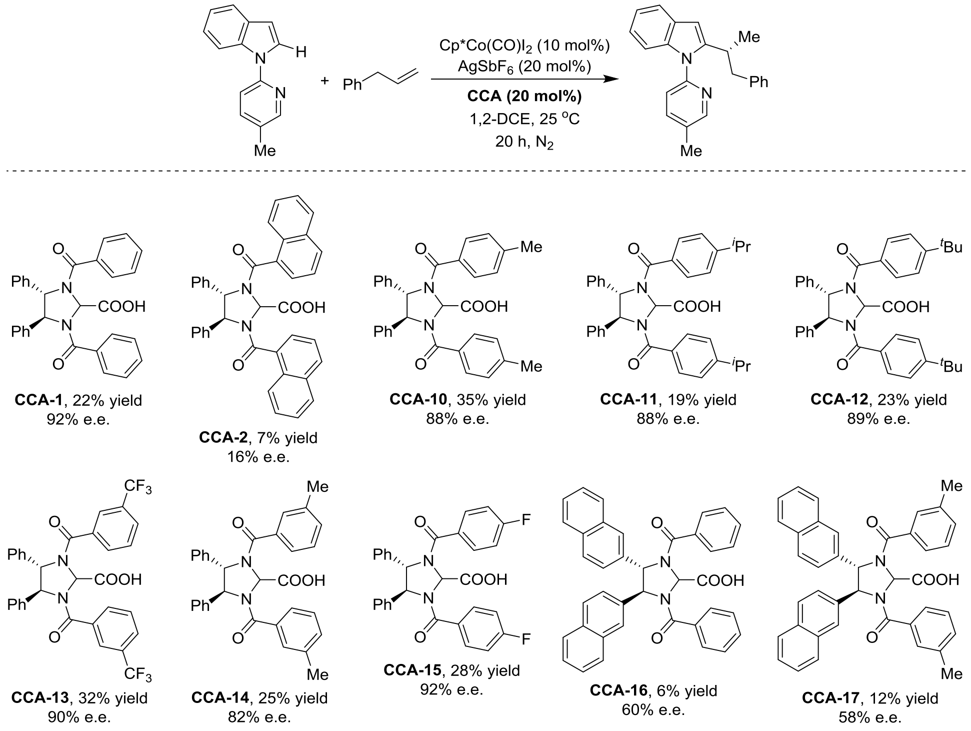

Asymmetric C−H alkylation with central chirality and axial chirality

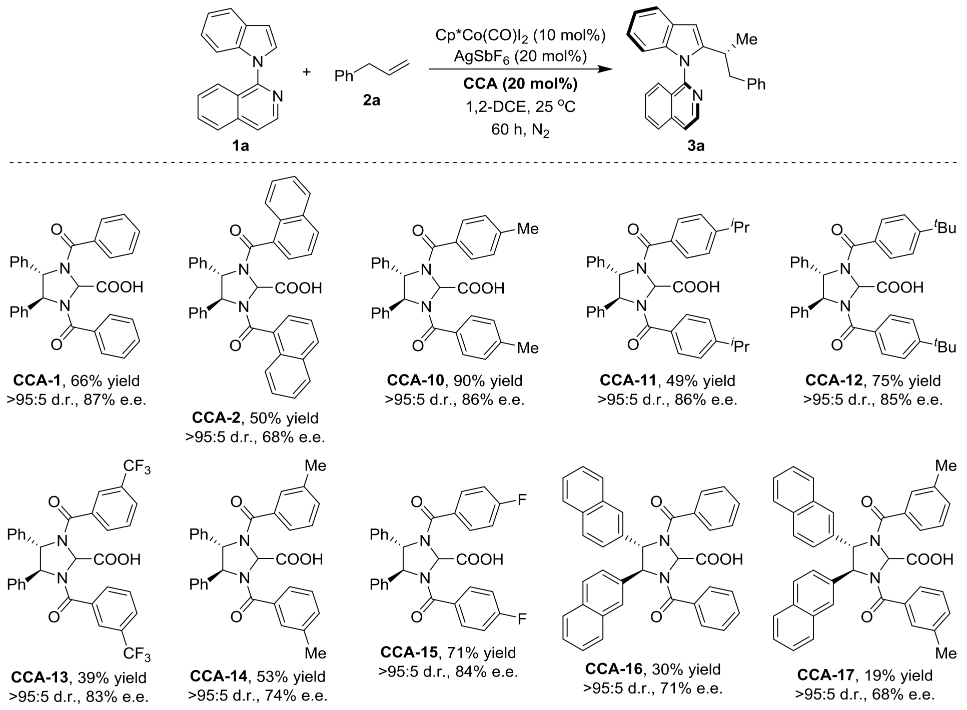

Supplementary Fig. 3 | Experimental stereoselectivities of 10 tested CCAs for rxn1 and rxn2.

3. Details of the predicted chiral carboxylic acids

For the candidate CCAs, we selected four aryl substitutions on the five-membered ring backbone and 90 *N*-acyl substitutions considering the variation of steric and electronic effects. The structural details of the substitutions are shown in Supplementary Table 1. The combinations of these selections created the 360 C2-symmetric candidate chiral carboxylic acids for virtual screening, whose SMILESs were automatically generated. All the related scripts for this process are available in our GitHub project (https://github.com/Shuwen-Li/FindBestChiralAcid).

Supplementary Table 1. Considered substitutions of the candidate chiral carboxylic acids for virtual screening.

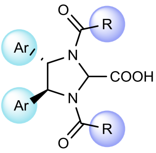

Substitution	Structure	Substitution	Structure
Ar-1	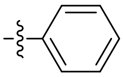	Ar-2	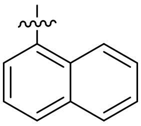
Ar-3	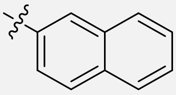	Ar-4	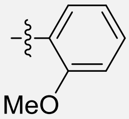
R-1	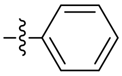	R-2	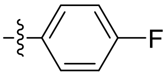
R-3	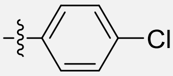	R-4	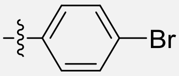
R-5	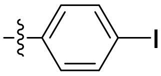	R-6	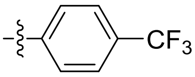
R-7	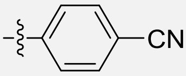	R-8	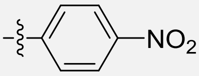
R-9	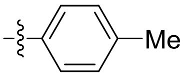	R-10	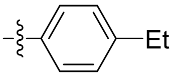
R-11	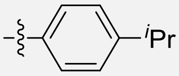	R-12	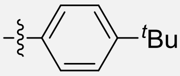
R-13	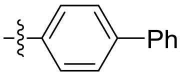	R-14	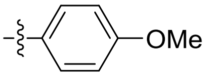
R-15	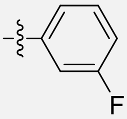	R-16	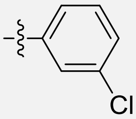
R-17	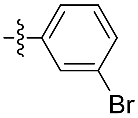	R-18	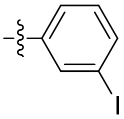

R-19	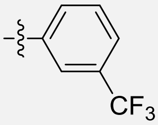	R-20	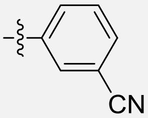
R-21	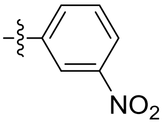	R-22	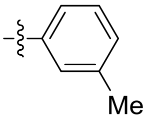
R-23	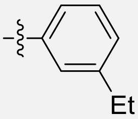	R-24	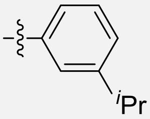
R-25	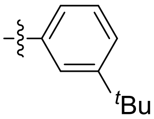	R-26	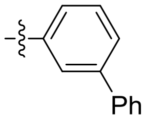
R-27	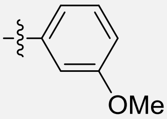	R-28	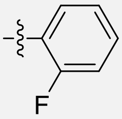
R-29	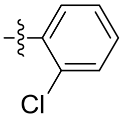	R-30	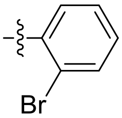
R-31	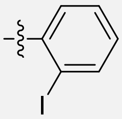	R-32	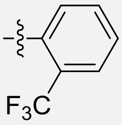
R-33	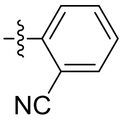	R-34	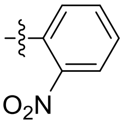
R-35	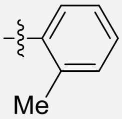	R-36	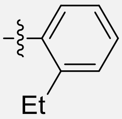
R-37	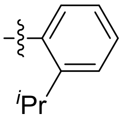	R-38	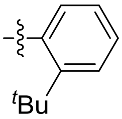
R-39	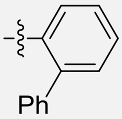	R-40	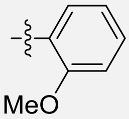
R-41	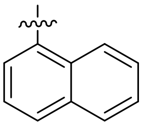	R-42	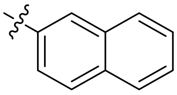
R-43	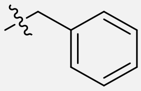	R-44	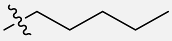

R-45	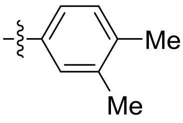	R-46	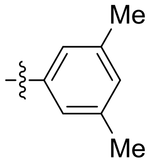
R-47	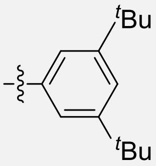	R-48	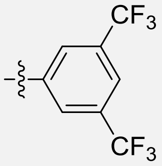
R-49	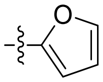	R-50	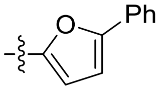
R-51	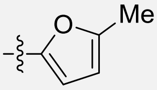	R-52	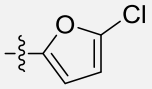
R-53	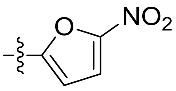	R-54	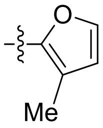
R-55	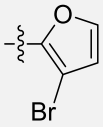	R-56	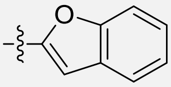
R-57	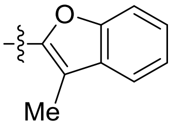	R-58	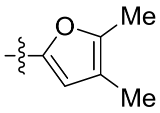
R-59	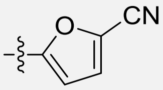	R-60	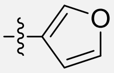
R-61	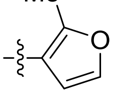	R-62	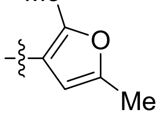
R-63	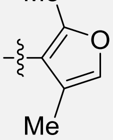	R-64	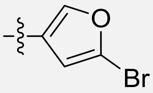
R-65	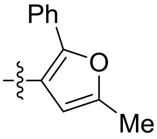	R-66	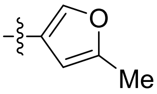

R-67	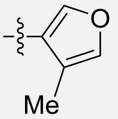	R-68	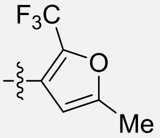
R-69	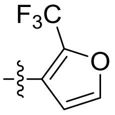	R-70	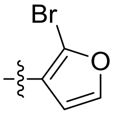
R-71	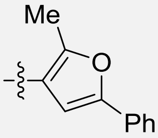	R-72	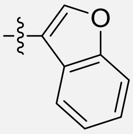
R-73	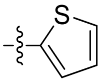	R-74	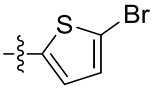
R-75	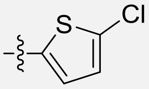	R-76	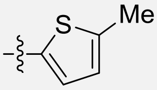
R-77	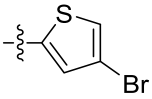	R-78	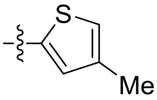
R-79	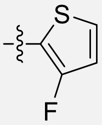	R-80	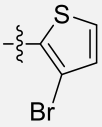
R-81	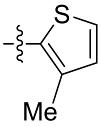	R-82	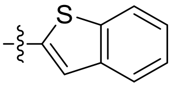
R-83	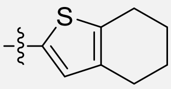	R-84	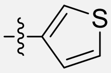
R-85	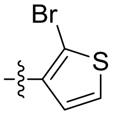	R-86	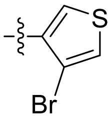
R-87	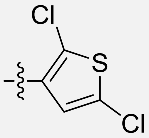	R-88	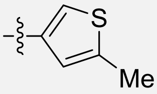
R-89	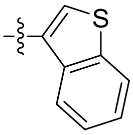	R-90	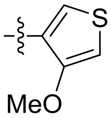

4. Structural optimization

To generate the 3D geometry for the recorded compounds, we used RDKit's[6] built-in ETKDG[7] method to generate the initial 3D structure. Subsequent geometric optimization was performed using the semiempirical extended tight-binding program package xTB[8], at the GFN2-xTB[9] level of theory. All the optimized geometries are available in our GitHub project (https://github.com/Shuwen-Li/FindBestChiralAcid).

5. Details of descriptors

A series of steric and electronic features were applied to describe the indoles, alkenes and chiral carboxylic acids in the asymmetric C−H alkylation, including Sterimol, buried volume, atomic charge, bond length, Wiberg Bond Order and frontier molecular orbital. All the descriptors were obtained based on the xTB-optimized geometries. Sterimol and buried volume were generated by moRFeus[10]. The entire catalysis encoding is a 108-dimensional physical organic space containing 35 descriptors for indoles, 6 descriptors for alkenes, 66 descriptors for CCAs and 1 descriptor for temperature. Full details of the used descriptors are elaborated in Supplementary Table 2.

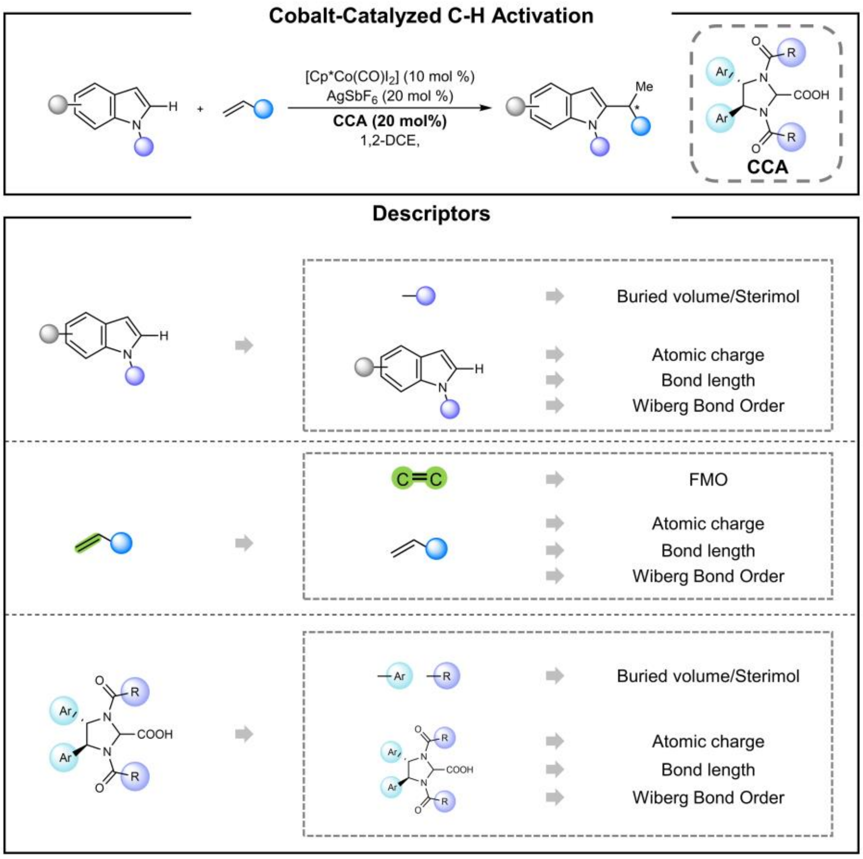

Supplementary Fig. 4 | Considered descriptors for the cobalt-catalyzed C–H alkylation of indoles.

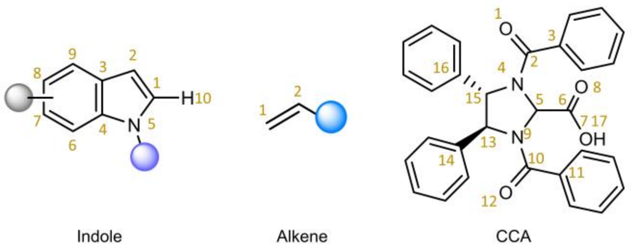

Supplementary Fig. 5 | Atom index of indoles, alkenes and CCAs.

Supplementary Table 2. Symbol, definition and access of descriptors.

Entry	Molecule	Symbol	Definition	Access
1	Indole	$\%V_{Bur}^{3.5Å}$	The buried volume of nitrogen-atom at indoles, sphere radius = 3.5Å	Calculation based on xTB optimized geometries
2	Indole	B_1	Sterimol parameter B_1 of nitrogen-atom at indoles, dummy index: nitrogen-atom, attached index: atom in substituent group attach to nitrogen-atom	Calculation based on xTB optimized geometries
3	Indole	B_5	Sterimol parameter B_5 of nitrogen-atom at indoles, dummy index: nitrogen-atom, attached index: atom in substituent group attach to nitrogen-atom	Calculation based on xTB optimized geometries
4	Indole	L	Sterimol parameter L of nitrogen-atom at indoles, dummy index: nitrogen-atom, attached index: atom in substituent group attach to nitrogen-atom	Calculation based on xTB optimized geometries
5	Indole	q^1	Charge of atom 1 at indoles	Based on xTB optimization
6	Indole	q^2	Charge of atom 2 at indoles	Based on xTB optimization
7	Indole	q^3	Charge of atom 3 at indoles	Based on xTB optimization
8	Indole	q^4	Charge of atom 4 at indoles	Based on xTB optimization
9	Indole	q^5	Charge of atom 5 at indoles	Based on xTB optimization
10	Indole	q^6	Charge of atom 6 at indoles	Based on xTB optimization
11	Indole	q^7	Charge of atom 7 at indoles	Based on xTB optimization
12	Indole	q^8	Charge of atom 8 at indoles	Based on xTB optimization
13	Indole	q^9	Charge of atom 9 at indoles	Based on xTB optimization
14	Indole	$B^{1,10}$	Wiberg Bond Order of atom 1 and atom 10 at indoles	Based on xTB optimization
15	Indole	$B^{1,5}$	Wiberg Bond Order of atom 1 and atom 5 at indoles	Based on xTB optimization
16	Indole	$B^{5,4}$	Wiberg Bond Order of atom 5 and atom 4 at indoles	Based on xTB optimization
17	Indole	$B^{4,3}$	Wiberg Bond Order of atom 4 and atom 3 at indoles	Based on xTB optimization
18	Indole	$B^{3,2}$	Wiberg Bond Order of atom 3 and atom 2 at indoles	Based on xTB optimization
19	Indole	$B^{2,1}$	Wiberg Bond Order of atom 2 and atom 1 at	Based on xTB

			indoles	optimization
20	Indole	$B^{4,6}$	Wiberg Bond Order of atom 4 and atom 6 at indoles	Based on xTB optimization
21	Indole	$B^{6,7}$	Wiberg Bond Order of atom 6 and atom 7 at indoles	Based on xTB optimization
22	Indole	$B^{7,8}$	Wiberg Bond Order of atom 7 and atom 8 at indoles	Based on xTB optimization
23	Indole	$B^{8,9}$	Wiberg Bond Order of atom 8 and atom 9 at indoles	Based on xTB optimization
24	Indole	$B^{9,3}$	Wiberg Bond Order of atom 9 and atom 3 at indoles	Based on xTB optimization
25	Indole	$d^{1,10}$	Bond length of atom 1 and atom 10 at indoles	Based on xTB optimized geometries
26	Indole	$d^{1,5}$	Bond length of atom 1 and atom 5 at indoles	Based on xTB optimized geometries
27	Indole	$d^{5,4}$	Bond length of atom 5 and atom 4 at indoles	Based on xTB optimized geometries
28	Indole	$d^{4,3}$	Bond length of atom 4 and atom 3 at indoles	Based on xTB optimized geometries
29	Indole	$d^{3,2}$	Bond length of atom 3 and atom 2 at indoles	Based on xTB optimized geometries
30	Indole	$d^{2,1}$	Bond length of atom 2 and atom 1 at indoles	Based on xTB optimized geometries
31	Indole	$d^{4,6}$	Bond length of atom 4 and atom 6 at indoles	Based on xTB optimized geometries
32	Indole	$d^{6,7}$	Bond length of atom 6 and atom 7 at indoles	Based on xTB optimized geometries
33	Indole	$d^{7,8}$	Bond length of atom 7 and atom 8 at indoles	Based on xTB optimized geometries
34	Indole	$d^{8,9}$	Bond length of atom 8 and atom 9 at indoles	Based on xTB optimized geometries
35	Indole	$d^{9,3}$	Bond length of atom 9 and atom 3 at indoles	Based on xTB optimized geometries
36	Alkene	$\pi_{C=C}^{HOMO}$	The energy of π occupied molecular orbital of the C=C bond of alkenes	Based on xTB optimization
37	Alkene	$\pi_{C=C}^{LUMO}$	The energy of π unoccupied molecular orbital of the C=C bond of alkenes	Based on xTB optimization
38	Alkene	q^1	Charge of atom 1 at alkenes	Based on xTB optimization
39	Alkene	q^2	Charge of atom 2 at alkenes	Based on xTB optimization
40	Alkene	$B^{1,2}$	Wiberg Bond Order of atom 1 and atom 2 at alkenes	Based on xTB optimization
41	Alkene	$d^{1,2}$	Bond length of atom 1 and atom 2 at alkenes	Based on xTB optimized geometries

42	CCA	$\%V_{Bur}^{3.5Å}$	The buried volume of atom 4 at CCAs, sphere radius = 3.5Å	Calculation based on xTB optimized geometries
43	CCA	$\%V_{Bur}^{3.5Å}$	The buried volume of atom 3 at CCAs, sphere radius = 3.5Å	Calculation based on xTB optimized geometries
44	CCA	$\%V_{Bur}^{3.5Å}$	The buried volume of atom 9 at CCAs, sphere radius = 3.5Å	Calculation based on xTB optimized geometries
45	CCA	$\%V_{Bur}^{3.5Å}$	The buried volume of atom 11 at CCAs, sphere radius = 3.5Å	Calculation based on xTB optimized geometries
46	CCA	$\%V_{Bur}^{3.5Å}$	The buried volume of atom 13 at CCAs, sphere radius = 3.5Å	Calculation based on xTB optimized geometries
47	CCA	$\%V_{Bur}^{3.5Å}$	The buried volume of atom 14 at CCAs, sphere radius = 3.5Å	Calculation based on xTB optimized geometries
48	CCA	$\%V_{Bur}^{3.5Å}$	The buried volume of atom 15 at CCAs, sphere radius = 3.5Å	Calculation based on xTB optimized geometries
49	CCA	$\%V_{Bur}^{3.5Å}$	The buried volume of atom 16 at CCAs, sphere radius = 3.5Å	Calculation based on xTB optimized geometries
50	CCA	B_1	Sterimol parameter B_1 of atom 2 at CCAs, dummy index: atom 2, attached index: atom in substituent group attach to atom 2	Calculation based on xTB optimized geometries
51	CCA	B_1	Sterimol parameter B_1 of atom 10 at CCAs, dummy index: atom 10, attached index: atom in substituent group attach to atom 10	Calculation based on xTB optimized geometries
52	CCA	B_1	Sterimol parameter B_1 of atom 13 at CCAs, dummy index: atom 13 attached index: atom in substituent group attach to atom 13	Calculation based on xTB optimized geometries
53	CCA	B_1	Sterimol parameter B_1 of atom 15 at CCAs, dummy index: atom 15 attached index: atom in substituent group attach to atom 15	Calculation based on xTB optimized geometries
54	CCA	B_5	Sterimol parameter B_5 of atom 2 at CCAs, dummy index: atom 2, attached index: atom in substituent group attach to atom 2	Calculation based on xTB optimized geometries
55	CCA	B_5	Sterimol parameter B_5 of atom 10 at CCAs, dummy index: atom 10, attached index: atom in substituent group attach to atom 10	Calculation based on xTB optimized geometries
56	CCA	B_5	Sterimol parameter B_5 of atom 13 at CCAs, dummy index: atom 13 attached index: atom in substituent group attach to atom 13	Calculation based on xTB optimized geometries
57	CCA	B_5	Sterimol parameter B_5 of atom 15 at CCAs, dummy index: atom 15 attached index: atom in substituent group attach to atom 15	Calculation based on xTB optimized geometries
58	CCA	L	Sterimol parameter L of atom 2 at CCAs, dummy index: atom 2, attached index: atom in substituent group attach to atom 2	Calculation based on xTB optimized geometries
59	CCA	L	Sterimol parameter L of atom 10 at CCAs, dummy index: atom 10, attached index: atom in substituent group attach to atom 10	Calculation based on xTB optimized geometries

60	CCA	L	Sterimol parameter L of atom 13 at CCAs, dummy index: atom 13, attached index: atom in substituent group attach to atom 13	Calculation based on xTB optimized geometries
61	CCA	L	Sterimol parameter L of atom 15 at CCAs, dummy index: atom 15, attached index: atom in substituent group attach to atom 15	Calculation based on xTB optimized geometries
62	CCA	q^1	Charge of atom 1 at CCAs	Based on xTB optimization
63	CCA	q^2	Charge of atom 2 at CCAs	Based on xTB optimization
64	CCA	q^3	Charge of atom 3 at CCAs	Based on xTB optimization
65	CCA	q^4	Charge of atom 4 at CCAs	Based on xTB optimization
66	CCA	q^5	Charge of atom 5 at CCAs	Based on xTB optimization
67	CCA	q^6	Charge of atom 6 at CCAs	Based on xTB optimization
68	CCA	q^7	Charge of atom 7 at CCAs	Based on xTB optimization
69	CCA	q^8	Charge of atom 8 at CCAs	Based on xTB optimization
70	CCA	q^9	Charge of atom 9 at CCAs	Based on xTB optimization
71	CCA	q^{10}	Charge of atom 10 at CCAs	Based on xTB optimization
72	CCA	q^{11}	Charge of atom 11 at CCAs	Based on xTB optimization
73	CCA	q^{12}	Charge of atom 12 at CCAs	Based on xTB optimization
74	CCA	q^{13}	Charge of atom 13 at CCAs	Based on xTB optimization
75	CCA	q^{14}	Charge of atom 14 at CCAs	Based on xTB optimization
76	CCA	q^{15}	Charge of atom 15 at CCAs	Based on xTB optimization
77	CCA	q^{16}	Charge of atom 16 at CCAs	Based on xTB optimization
78	CCA	$B^{5,4}$	Wiberg Bond Order of atom 5 and atom 4 at CCAs	Based on xTB optimization
79	CCA	$B^{4,15}$	Wiberg Bond Order of atom 4 and atom 15 at CCAs	Based on xTB optimization
80	CCA	$B^{15,13}$	Wiberg Bond Order of atom 15 and atom 13 at CCAs	Based on xTB optimization
81	CCA	$B^{13,9}$	Wiberg Bond Order of atom 13 and atom 9 at CCAs	Based on xTB optimization

82	CCA	$B^{9,5}$	Wiberg Bond Order of atom 9 and atom 5 at CCAs	Based on xTB optimization
83	CCA	$B^{5,6}$	Wiberg Bond Order of atom 5 and atom 6 at CCAs	Based on xTB optimization
84	CCA	$B^{6,7}$	Wiberg Bond Order of atom 6 and atom 7 at CCAs	Based on xTB optimization
85	CCA	$B^{6,8}$	Wiberg Bond Order of atom 6 and atom 8 at CCAs	Based on xTB optimization
86	CCA	$B^{4,2}$	Wiberg Bond Order of atom 4 and atom 2 at CCAs	Based on xTB optimization
87	CCA	$B^{2,3}$	Wiberg Bond Order of atom 2 and atom 3 at CCAs	Based on xTB optimization
88	CCA	$B^{9,10}$	Wiberg Bond Order of atom 9 and atom 10 at CCAs	Based on xTB optimization
89	CCA	$B^{10,11}$	Wiberg Bond Order of atom 10 and atom 11at CCAs	Based on xTB optimization
90	CCA	$B^{2,1}$	Wiberg Bond Order of atom 2 and atom 1 at CCAs	Based on xTB optimization
91	CCA	$B^{10,12}$	Wiberg Bond Order of atom 10 and atom 12 at CCAs	Based on xTB optimization
92	CCA	$B^{15,16}$	Wiberg Bond Order of atom 15 and atom 16 at CCAs	Based on xTB optimization
93	CCA	$B^{13,14}$	Wiberg Bond Order of atom 13 and atom 14 at CCAs	Based on xTB optimization
94	CCA	$B^{7,17}$	Wiberg Bond Order of atom 7 and atom 17 at CCAs	Based on xTB optimization
95	CCA	$d^{15,16}$	Bond length of atom 8 and atom 9 at CCAs	Based on xTB optimized geometries
96	CCA	$d^{13,14}$	Bond length of atom 8 and atom 9 at CCAs	Based on xTB optimized geometries
97	CCA	$d^{4,2}$	Bond length of atom 8 and atom 9 at CCAs	Based on xTB optimized geometries
98	CCA	$d^{9,10}$	Bond length of atom 8 and atom 9 at CCAs	Based on xTB optimized geometries
99	CCA	$d^{5,9}$	Bond length of atom 8 and atom 9 at CCAs	Based on xTB optimized geometries
100	CCA	$d^{9,13}$	Bond length of atom 8 and atom 9 at CCAs	Based on xTB optimized geometries
101	CCA	$d^{13,15}$	Bond length of atom 8 and atom 9 at CCAs	Based on xTB optimized geometries
102	CCA	$d^{15,4}$	Bond length of atom 8 and atom 9 at CCAs	Based on xTB optimized geometries
103	CCA	$d^{4,5}$	Bond length of atom 8 and atom 9 at CCAs	Based on xTB optimized geometries
104	CCA	$d^{5,6}$	Bond length of atom 8 and atom 9 at CCAs	Based on xTB optimized geometries

105	CCA	$d^{6,7}$	Bond length of atom 8 and atom 9 at CCAs	Based on xTB optimized geometries
106	CCA	$d^{6,8}$	Bond length of atom 8 and atom 9 at CCAs	Based on xTB optimized geometries
107	CCA	$d^{7,17}$	Bond length of atom 8 and atom 9 at CCAs	Based on xTB optimized geometries
108		Tempera ture	Reaction temperature	Temperature value

6. Details of machine learning algorithms

A series of widely used machine learning algorithms were tested for model training, including Bagging regression[11], Decision Trees[12], Extra-Trees[13], Gradient Boosting[14], k-Nearest Neighbors regression[15], Kernel Ridge regression[16], Linear Support Vector Regression[17], Random Forest Regression[18], Ridge[19], Support Vector Regression[17] and XGBoost[20]. The model training was performed using scikit-learn[21] and xgboost python packages[22]. The parameters of each tested algorithm were included in Supplementary Table 3. All the related scripts for model training were available in our GitHub project (https://github.com/Shuwen-Li/FindBestChiralAcid).

Supplementary Table 3. The hyper-parameters of the tested machine learning algorithms for model training.

Model	Modules and parameters
Bagging (BG)	sklearn.ensemble.BaggingRegressor(base_estimator=None, n_estimators=10, max_samples=1.0, max_features=1.0, bootstrap=True, bootstrap_features=False, oob_score=False, warm_start=False, n_jobs=60, random_state=None, verbose=0)
Decision Tree (DT)	sklearn.tree.DecisionTreeRegressor(criterion='mse', splitter='best', max_depth=None, min_samples_split=2, min_samples_leaf=1, min_weight_fraction_leaf=0.0, max_features=None, random_state=None, max_leaf_nodes=None, min_impurity_decrease=0.0, min_impurity_split=None, presort='deprecated', ccp_alpha=0.0)
Extra-Trees (ET)	sklearn.ensemble.ExtraTreesRegressor(n_estimators=100, criterion='mse', max_depth=None, min_samples_split=2, min_samples_leaf=1, min_weight_fraction_leaf=0.0, max_features='auto', max_leaf_nodes=None, min_impurity_decrease=0.0, bootstrap=False, oob_score=False, n_jobs=60, random_state=None, verbose=0, warm_start=False, ccp_alpha=0.0, max_samples=None)
Gradient Boosting (GB)	sklearn.ensemble.GradientBoostingRegressor(loss='ls', learning_rate=0.1, n_estimators=100, subsample=1.0, criterion='friedman_mse', min_samples_split=2, min_samples_leaf=1, min_weight_fraction_leaf=0.0, max_depth=3, min_impurity_decrease=0.0, init=None, random_state=None, max_features=None, alpha=0.9, verbose=0, max_leaf_nodes=None, warm_start=False, validation_fraction=0.1, n_iter_no_change=None, tol=0.0001, ccp_alpha=0.0)
k-Nearest Neighbors Regression (KNR)	sklearn.neighbors.NearestNeighbors(n_neighbors=5,weights='uniform',algorithm='auto',leaf_size=30,p=2,metric='minkowski',metric_params=None,n_jobs=None)
KernelRidge (KRR)	sklearn.kernel_ridge.KernelRidge(alpha=1, kernel='linear', gamma=None, degree=3, coef0=1, kernel_params=None)
Linear Support Vector Regression (LSVR)	sklearn.svm.LinearSVR(epsilon=0.0,tol=0.0001,C=1.0,loss='epsilon_insensitive',fit_intercept=True,intercept_scaling=1.0,dual=True,verbose=0,random_state=None,max_iter=1000)
RandomForest (RF)	sklearn.ensemble.RandomForestRegressor(n_estimators=100,criterion='mse', max_depth=None,min_samples_split=2,min_samples_leaf=1,min_weight_fra

	ction_leaf=0.0,max_features='auto',max_leaf_nodes=None,min_impurity_decrease=0.0,bootstrap=True,oob_score=False,n_jobs=60,random_state=None,verbose=0,warm_start=False,ccp_alpha=0.0,max_samples=None)
Ridge	sklearn.linear_model.Ridge(alpha=1.0,fit_intercept=True,copy_X=True,max_iter=None,tol=0.001)
Support Vector Regression (SVR)	sklearn.svm.SVR(kernel='rbf', degree=3, gamma='scale', coef0=0.0, tol=0.001, C=1.0, epsilon=0.1, shrinking=True, cache_size=200, verbose=False, max_iter=- 1)
XGBoost (XGB)	xgboost.XGBRegressor(base_score=0.5, oster='gbtree', olsample_bylevel=1, colsample_bynode=1, colsample_bytree=1, gamma=0, gpu_id=-1, importance_type='gain', interaction_constraints='', learning_rate=0.3, max_delta_step=0, max_depth=10, min_child_weight=1, missing=np.nan, monotone_constraints='()', n_estimators=60, num_parallel_tree=1, random_state=0, reg_alpha=0, reg_lambda=1, scale_pos_weight=1, subsample=1, tree_method='exact', validate_parameters=1, verbosity=None)

7. Details of model predictions

7.1 Model selection

Eleven regression algorithms (Bagging Regression, Decision Trees, Extra-Trees, Gradient Boosting, k-Nearest Neighbors Regression, Kernel Ridge Regression, Linear Support Vector Regression, Random Forest Regression, Ridge, Support Vector Regression, and XGBoost) were tested for the enantioselectivity prediction of **rxn1**, using the full 108-dimensional encodings. The Pearson Rs in 10-fold cross-validation are shown in Supplementary Table 4.

Supplementary Table 4. Pearson R and MAE of the enantioselectivity predictions for **rxn1** in 10-fold cross-validation using various algorithms.

Model	MAE (kcal/mol)	Pearson R
Bagging	0.215	0.791
Decision Tree	0.268	0.665
Extra-Trees	0.208	0.790
Gradient Boosting	0.212	0.819
k-Nearest Neighbors Regression	0.235	0.777
Kernel Ridge Regression	0.205	0.834
Linear Support Vector Regression	0.196	0.841
Random Forest	0.203	0.812
Ridge	0.209	0.833
Support Vector Regression	0.222	0.822
XGBoost	0.228	0.770

7.2 Descriptor selection

Using Linear Support Vector Regression which is the optimal algorithm for 10-fold cross validation, descriptor selection was performed. For each round of descriptor addition, the highest performance-improving descriptor was selected and added until no further improvement can be achieved. The regression performances for **rxn1** prior to and after the descriptor selection are shown in Supplementary Fig. 6. Python script is available at GitHub: https://github.com/Shuwen-Li/FindBestChiralAcid.

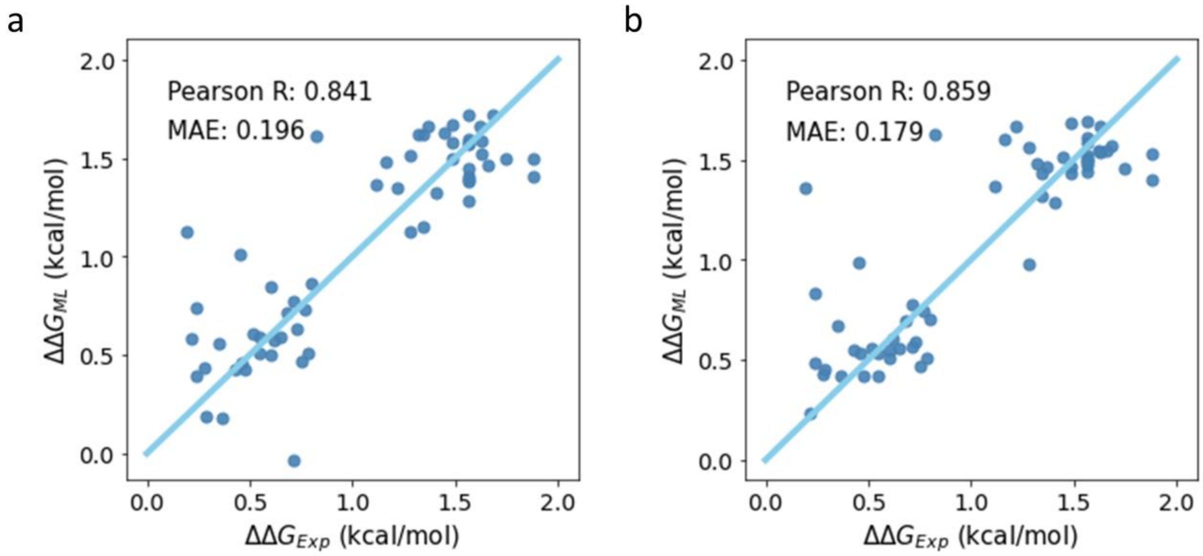

Supplementary Fig. 6 | Regression performances for rxn1 prior to the descriptor selection (a) and after descriptor selection (b).

7.3 Details of the prediction for rxn2

Using the trained machine learning model for **rxn1**, its direct predictions in **rxn2** provided the predicted enantioselectivities for the ten experimentally tested chiral carboxylic acids ($\Delta\Delta G_{pred}$). For these ten examples of **rxn2**, each acid has a predicted enantioselectivity ($\Delta\Delta G_{pred}$) and the experimentally measured enantioselectivity ($\Delta\Delta G_{exp}$). The differences between the two values ($D = \Delta\Delta G_{pred} - \Delta\Delta G_{exp}$) were used as the target for the training of the delta model. In the delta model training, the leave-one-out (LOO) training was used due to the limit data size. The final prediction of **rxn2** is the sum of the $\Delta\Delta G_{pred}$ and D_{pred}. The details of the experimental enantioselectivity as well as the model's predicted enantioselectivities prior to and after the LOO delta learning are shown in Supplementary Table 5.

Supplementary Table 5. The details of the experimental enantioselectivity as well as the model's predicted enantioselectivities prior to and after the LOO delta learning

CCA	Experimental $\Delta\Delta G$	Predicted $\Delta\Delta G$ prior to delta learning	Predicted $\Delta\Delta G$ after delta learning
1	1.58	1.22	1.45
2	1.45	1.15	1.27
3	1.53	1.29	1.52
4	1.53	1.32	1.45
5	1.49	1.29	1.54
6	1.41	1.21	1.35
7	1.13	1.10	1.29
8	0.98	1.03	1.15
9	1.05	1.29	1.11
10	0.98	1.25	1.03
MAE (kcal/mol)		0.210	0.095
Pearson R		0.451	0.898

7.4 Predicted enantioselectivities of the 360 candidate acids

Using the developed knowledge transfer model, the enantioselectivities of the 360 candidate chiral carboxylic acids for **rxn2** were predicted. The structural details of the CCAs are provided in Supplementary Table 1. The detailed prediction results are provided in Supplementary Table 6.

Supplementary Table 6. Details of the predicted enantioselectivities for rxn2 using the knowledge transfer approach.

CCA	Ar	R	Predicted e.e. (%)	Predicted $\Delta\Delta G$
1	Ar-1	R-1	86	1.55
2	Ar-1	R-2	81	1.35
3	Ar-1	R-3	75	1.15
4	Ar-1	R-4	74	1.13
5	Ar-1	R-5	82	1.38
6	Ar-1	R-6	80	1.30
7	Ar-1	R-7	88	1.65
8	Ar-1	R-8	79	1.28
9	Ar-1	R-9	86	1.53
10	Ar-1	R-10	87	1.57
11	Ar-1	R-11	86	1.53
12	Ar-1	R-12	85	1.49
13	Ar-1	R-13	78	1.25
14	Ar-1	R-14	88	1.66
15	Ar-1	R-15	80	1.29
16	Ar-1	R-16	77	1.22
17	Ar-1	R-17	80	1.30
18	Ar-1	R-18	85	1.48
19	Ar-1	R-19	83	1.41
20	Ar-1	R-20	79	1.27
21	Ar-1	R-21	83	1.40
22	Ar-1	R-22	76	1.19
23	Ar-1	R-23	72	1.08
24	Ar-1	R-24	63	0.87

25	Ar-1	R-25	70	1.02
26	Ar-1	R-26	75	1.17
27	Ar-1	R-27	71	1.05
28	Ar-1	R-28	67	0.96
29	Ar-1	R-29	73	1.09
30	Ar-1	R-30	72	1.08
31	Ar-1	R-31	67	0.96
32	Ar-1	R-32	81	1.32
33	Ar-1	R-33	73	1.09
34	Ar-1	R-34	69	1.01
35	Ar-1	R-35	75	1.16
36	Ar-1	R-36	75	1.14
37	Ar-1	R-37	66	0.94
38	Ar-1	R-38	76	1.17
39	Ar-1	R-39	70	1.04
40	Ar-1	R-40	72	1.08
41	Ar-1	R-41	70	1.03
42	Ar-1	R-42	74	1.12
43	Ar-1	R-43	67	0.96
44	Ar-1	R-44	88	1.63
45	Ar-1	R-45	79	1.28
46	Ar-1	R-46	85	1.48
47	Ar-1	R-47	79	1.27
48	Ar-1	R-48	71	1.05
49	Ar-1	R-49	89	1.70
50	Ar-1	R-50	88	1.65
51	Ar-1	R-51	86	1.53
52	Ar-1	R-52	86	1.51
53	Ar-1	R-53	86	1.52
54	Ar-1	R-54	73	1.10
55	Ar-1	R-55	74	1.12
56	Ar-1	R-56	87	1.56

57	Ar-1	R-57	84	1.43
58	Ar-1	R-58	84	1.45
59	Ar-1	R-59	85	1.47
60	Ar-1	R-60	89	1.69
61	Ar-1	R-61	77	1.21
62	Ar-1	R-62	73	1.10
63	Ar-1	R-63	71	1.05
64	Ar-1	R-64	77	1.22
65	Ar-1	R-65	72	1.07
66	Ar-1	R-66	87	1.56
67	Ar-1	R-67	80	1.30
68	Ar-1	R-68	82	1.37
69	Ar-1	R-69	79	1.26
70	Ar-1	R-70	73	1.10
71	Ar-1	R-71	83	1.43
72	Ar-1	R-72	83	1.39
73	Ar-1	R-73	79	1.26
74	Ar-1	R-74	83	1.41
75	Ar-1	R-75	86	1.51
76	Ar-1	R-76	79	1.27
77	Ar-1	R-77	78	1.24
78	Ar-1	R-78	88	1.62
79	Ar-1	R-79	83	1.42
80	Ar-1	R-80	65	0.91
81	Ar-1	R-81	83	1.43
82	Ar-1	R-82	68	0.99
83	Ar-1	R-83	81	1.34
84	Ar-1	R-84	77	1.20
85	Ar-1	R-85	72	1.09
86	Ar-1	R-86	69	1.01
87	Ar-1	R-87	73	1.11
88	Ar-1	R-88	87	1.57

89	Ar-1	R-89	84	1.46
90	Ar-1	R-90	75	1.16
91	Ar-2	R-1	71	1.05
92	Ar-2	R-2	64	0.90
93	Ar-2	R-3	76	1.17
94	Ar-2	R-4	74	1.13
95	Ar-2	R-5	74	1.13
96	Ar-2	R-6	68	0.97
97	Ar-2	R-7	82	1.38
98	Ar-2	R-8	77	1.22
99	Ar-2	R-9	76	1.18
100	Ar-2	R-10	68	0.99
101	Ar-2	R-11	79	1.27
102	Ar-2	R-12	70	1.02
103	Ar-2	R-13	79	1.28
104	Ar-2	R-14	81	1.34
105	Ar-2	R-15	75	1.15
106	Ar-2	R-16	58	0.79
107	Ar-2	R-17	63	0.89
108	Ar-2	R-18	71	1.06
109	Ar-2	R-19	64	0.90
110	Ar-2	R-20	71	1.06
111	Ar-2	R-21	71	1.05
112	Ar-2	R-22	79	1.26
113	Ar-2	R-23	71	1.06
114	Ar-2	R-24	84	1.44
115	Ar-2	R-25	81	1.33
116	Ar-2	R-26	69	1.02
117	Ar-2	R-27	78	1.25
118	Ar-2	R-28	65	0.92
119	Ar-2	R-29	74	1.12
120	Ar-2	R-30	67	0.95

121	Ar-2	R-31	60	0.82
122	Ar-2	R-32	54	0.72
123	Ar-2	R-33	66	0.94
124	Ar-2	R-34	60	0.81
125	Ar-2	R-35	75	1.16
126	Ar-2	R-36	67	0.96
127	Ar-2	R-37	64	0.90
128	Ar-2	R-38	62	0.85
129	Ar-2	R-39	57	0.76
130	Ar-2	R-40	43	0.55
131	Ar-2	R-41	61	0.84
132	Ar-2	R-42	77	1.22
133	Ar-2	R-43	65	0.92
134	Ar-2	R-44	70	1.02
135	Ar-2	R-45	63	0.88
136	Ar-2	R-46	64	0.90
137	Ar-2	R-47	68	0.98
138	Ar-2	R-48	74	1.12
139	Ar-2	R-49	72	1.07
140	Ar-2	R-50	79	1.27
141	Ar-2	R-51	74	1.12
142	Ar-2	R-52	82	1.37
143	Ar-2	R-53	75	1.15
144	Ar-2	R-54	77	1.22
145	Ar-2	R-55	75	1.16
146	Ar-2	R-56	80	1.29
147	Ar-2	R-57	80	1.29
148	Ar-2	R-58	80	1.30
149	Ar-2	R-59	63	0.89
150	Ar-2	R-60	75	1.14
151	Ar-2	R-61	68	0.98
152	Ar-2	R-62	76	1.18

153	Ar-2	R-63	70	1.03
154	Ar-2	R-64	81	1.32
155	Ar-2	R-65	79	1.28
156	Ar-2	R-66	83	1.42
157	Ar-2	R-67	64	0.91
158	Ar-2	R-68	66	0.93
159	Ar-2	R-69	73	1.10
160	Ar-2	R-70	55	0.74
161	Ar-2	R-71	86	1.55
162	Ar-2	R-72	78	1.24
163	Ar-2	R-73	71	1.06
164	Ar-2	R-74	81	1.33
165	Ar-2	R-75	74	1.12
166	Ar-2	R-76	77	1.22
167	Ar-2	R-77	69	1.00
168	Ar-2	R-78	73	1.10
169	Ar-2	R-79	65	0.92
170	Ar-2	R-80	58	0.78
171	Ar-2	R-81	77	1.21
172	Ar-2	R-82	79	1.28
173	Ar-2	R-83	62	0.86
174	Ar-2	R-84	69	1.01
175	Ar-2	R-85	61	0.83
176	Ar-2	R-86	70	1.03
177	Ar-2	R-87	71	1.05
178	Ar-2	R-88	73	1.09
179	Ar-2	R-89	67	0.96
180	Ar-2	R-90	61	0.84
181	Ar-3	R-1	71	1.05
182	Ar-3	R-2	75	1.16
183	Ar-3	R-3	81	1.33
184	Ar-3	R-4	81	1.32

185	Ar-3	R-5	79	1.28
186	Ar-3	R-6	73	1.10
187	Ar-3	R-7	68	0.99
188	Ar-3	R-8	75	1.16
189	Ar-3	R-9	73	1.10
190	Ar-3	R-10	70	1.03
191	Ar-3	R-11	78	1.23
192	Ar-3	R-12	66	0.94
193	Ar-3	R-13	81	1.32
194	Ar-3	R-14	82	1.38
195	Ar-3	R-15	73	1.11
196	Ar-3	R-16	84	1.45
197	Ar-3	R-17	60	0.82
198	Ar-3	R-18	73	1.11
199	Ar-3	R-19	68	0.99
200	Ar-3	R-20	71	1.06
201	Ar-3	R-21	79	1.29
202	Ar-3	R-22	68	0.98
203	Ar-3	R-23	76	1.19
204	Ar-3	R-24	74	1.12
205	Ar-3	R-25	65	0.91
206	Ar-3	R-26	81	1.35
207	Ar-3	R-27	76	1.17
208	Ar-3	R-28	52	0.68
209	Ar-3	R-29	65	0.92
210	Ar-3	R-30	68	0.98
211	Ar-3	R-31	51	0.67
212	Ar-3	R-32	70	1.04
213	Ar-3	R-33	67	0.96
214	Ar-3	R-34	63	0.88
215	Ar-3	R-35	64	0.90
216	Ar-3	R-36	64	0.90

217	Ar-3	R-37	54	0.71
218	Ar-3	R-38	40	0.51
219	Ar-3	R-39	53	0.69
220	Ar-3	R-40	75	1.15
221	Ar-3	R-41	68	0.97
222	Ar-3	R-42	85	1.49
223	Ar-3	R-43	73	1.09
224	Ar-3	R-44	78	1.25
225	Ar-3	R-45	84	1.46
226	Ar-3	R-46	84	1.47
227	Ar-3	R-47	77	1.21
228	Ar-3	R-48	80	1.31
229	Ar-3	R-49	80	1.30
230	Ar-3	R-50	85	1.48
231	Ar-3	R-51	84	1.45
232	Ar-3	R-52	82	1.37
233	Ar-3	R-53	86	1.55
234	Ar-3	R-54	88	1.65
235	Ar-3	R-55	66	0.93
236	Ar-3	R-56	86	1.51
237	Ar-3	R-57	78	1.24
238	Ar-3	R-58	77	1.20
239	Ar-3	R-59	76	1.19
240	Ar-3	R-60	81	1.33
241	Ar-3	R-61	67	0.96
242	Ar-3	R-62	77	1.20
243	Ar-3	R-63	61	0.84
244	Ar-3	R-64	77	1.20
245	Ar-3	R-65	59	0.80
246	Ar-3	R-66	71	1.04
247	Ar-3	R-67	58	0.79
248	Ar-3	R-68	63	0.87

249	Ar-3	R-69	67	0.95
250	Ar-3	R-70	57	0.77
251	Ar-3	R-71	78	1.25
252	Ar-3	R-72	77	1.20
253	Ar-3	R-73	69	1.02
254	Ar-3	R-74	78	1.23
255	Ar-3	R-75	81	1.34
256	Ar-3	R-76	86	1.53
257	Ar-3	R-77	81	1.34
258	Ar-3	R-78	64	0.89
259	Ar-3	R-79	67	0.96
260	Ar-3	R-80	70	1.03
261	Ar-3	R-81	65	0.91
262	Ar-3	R-82	78	1.25
263	Ar-3	R-83	70	1.03
264	Ar-3	R-84	67	0.96
265	Ar-3	R-85	49	0.63
266	Ar-3	R-86	78	1.24
267	Ar-3	R-87	52	0.69
268	Ar-3	R-88	73	1.11
269	Ar-3	R-89	54	0.71
270	Ar-3	R-90	59	0.80
271	Ar-4	R-1	66	0.94
272	Ar-4	R-2	47	0.60
273	Ar-4	R-3	60	0.81
274	Ar-4	R-4	67	0.97
275	Ar-4	R-5	63	0.88
276	Ar-4	R-6	48	0.62
277	Ar-4	R-7	64	0.91
278	Ar-4	R-8	72	1.07
279	Ar-4	R-9	61	0.84
280	Ar-4	R-10	63	0.88

281	Ar-4	R-11	66	0.94
282	Ar-4	R-12	62	0.87
283	Ar-4	R-13	59	0.80
284	Ar-4	R-14	63	0.87
285	Ar-4	R-15	66	0.94
286	Ar-4	R-16	47	0.60
287	Ar-4	R-17	48	0.62
288	Ar-4	R-18	31	0.38
289	Ar-4	R-19	62	0.86
290	Ar-4	R-20	62	0.87
291	Ar-4	R-21	49	0.63
292	Ar-4	R-22	54	0.71
293	Ar-4	R-23	19	0.23
294	Ar-4	R-24	58	0.78
295	Ar-4	R-25	56	0.75
296	Ar-4	R-26	53	0.70
297	Ar-4	R-27	49	0.64
298	Ar-4	R-28	66	0.94
299	Ar-4	R-29	41	0.52
300	Ar-4	R-30	39	0.49
301	Ar-4	R-31	45	0.57
302	Ar-4	R-32	39	0.48
303	Ar-4	R-33	26	0.31
304	Ar-4	R-34	47	0.61
305	Ar-4	R-35	62	0.85
306	Ar-4	R-36	44	0.56
307	Ar-4	R-37	53	0.70
308	Ar-4	R-38	46	0.59
309	Ar-4	R-39	55	0.73
310	Ar-4	R-40	60	0.82
311	Ar-4	R-41	47	0.61
312	Ar-4	R-42	70	1.03

313	Ar-4	R-43	36	0.45
314	Ar-4	R-44	70	1.02
315	Ar-4	R-45	46	0.59
316	Ar-4	R-46	60	0.82
317	Ar-4	R-47	61	0.85
318	Ar-4	R-48	59	0.81
319	Ar-4	R-49	69	1.02
320	Ar-4	R-50	77	1.20
321	Ar-4	R-51	73	1.10
322	Ar-4	R-52	74	1.12
323	Ar-4	R-53	56	0.75
324	Ar-4	R-54	59	0.81
325	Ar-4	R-55	19	0.23
326	Ar-4	R-56	67	0.97
327	Ar-4	R-57	61	0.84
328	Ar-4	R-58	65	0.92
329	Ar-4	R-59	68	0.98
330	Ar-4	R-60	67	0.97
331	Ar-4	R-61	60	0.82
332	Ar-4	R-62	71	1.04
333	Ar-4	R-63	75	1.17
334	Ar-4	R-64	68	0.98
335	Ar-4	R-65	41	0.51
336	Ar-4	R-66	58	0.78
337	Ar-4	R-67	44	0.55
338	Ar-4	R-68	45	0.57
339	Ar-4	R-69	52	0.69
340	Ar-4	R-70	26	0.32
341	Ar-4	R-71	68	0.98
342	Ar-4	R-72	66	0.93
343	Ar-4	R-73	66	0.94
344	Ar-4	R-74	70	1.02

345	Ar-4	R-75	70	1.03
346	Ar-4	R-76	71	1.05
347	Ar-4	R-77	49	0.64
348	Ar-4	R-78	60	0.82
349	Ar-4	R-79	55	0.74
350	Ar-4	R-80	55	0.74
351	Ar-4	R-81	31	0.38
352	Ar-4	R-82	63	0.87
353	Ar-4	R-83	66	0.93
354	Ar-4	R-84	39	0.49
355	Ar-4	R-85	30	0.36
356	Ar-4	R-86	21	0.25
357	Ar-4	R-87	50	0.66
358	Ar-4	R-88	57	0.76
359	Ar-4	R-89	39	0.49
360	Ar-4	R-90	50	0.65

8. Comparison of the knowledge transfer predictions using 10 and 15 data input

In order to test the stability of model prediction, we synthesized five additional CCAs and generated five extra data to further validate the reliability of our model built with the ten original delta data (Supplementary Fig. 7). We then used these 15 delta data to retrain the delta learning model and compared the prediction results with those obtained by training with the ten original data. Supplementary Fig. 8a shows the prediction results and the corresponding experimental selectivities for the seven CCAs evaluated for the target substrate **1a**. The additional five data had a relatively small impact on the modeling, and the two sets of prediction values were highly correlated (Pearson R = 0.961, Supplementary Fig. 8b). These additional data further validated the previous delta learning results.

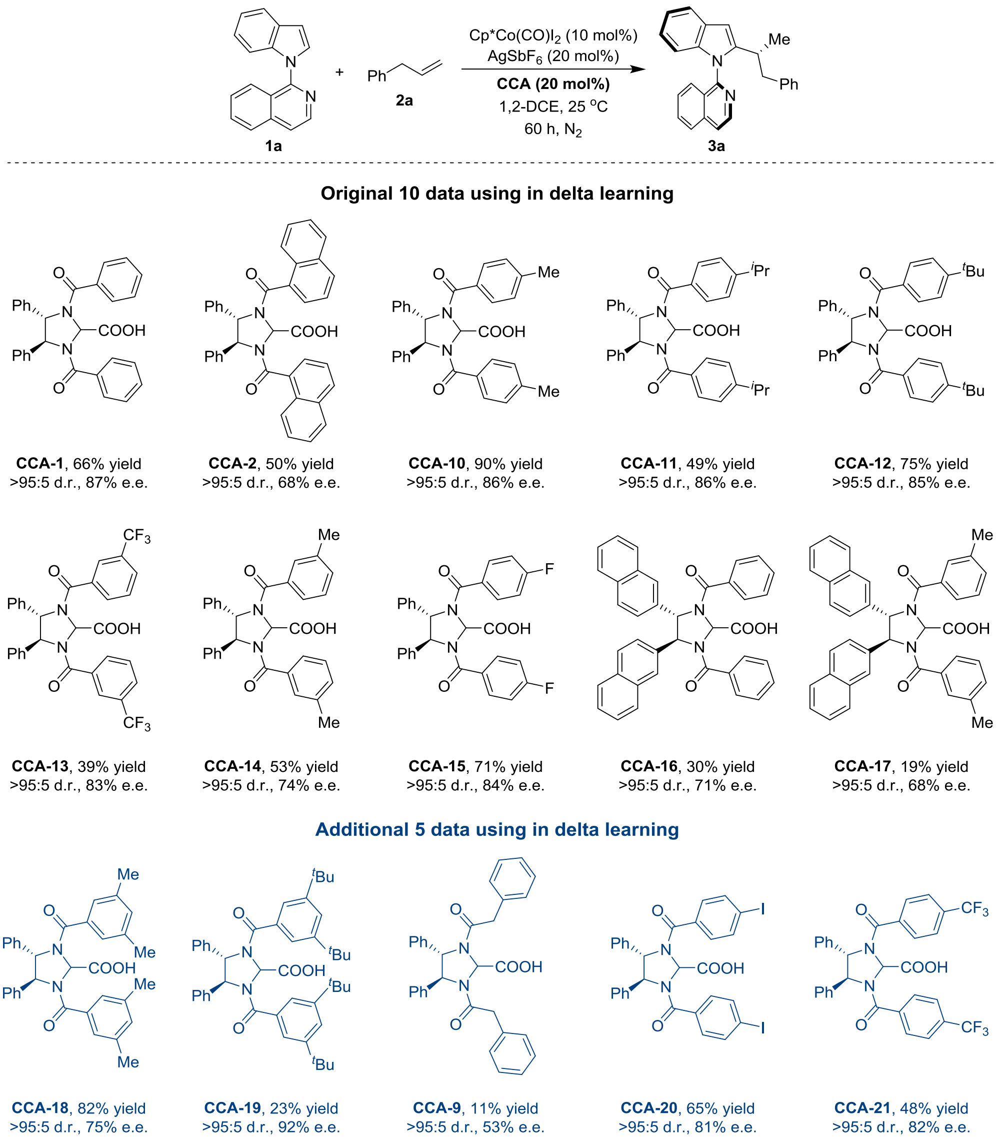

Supplementary Fig. 7 | Original and additional CCAs tested for the delta learning of substrate 1a.

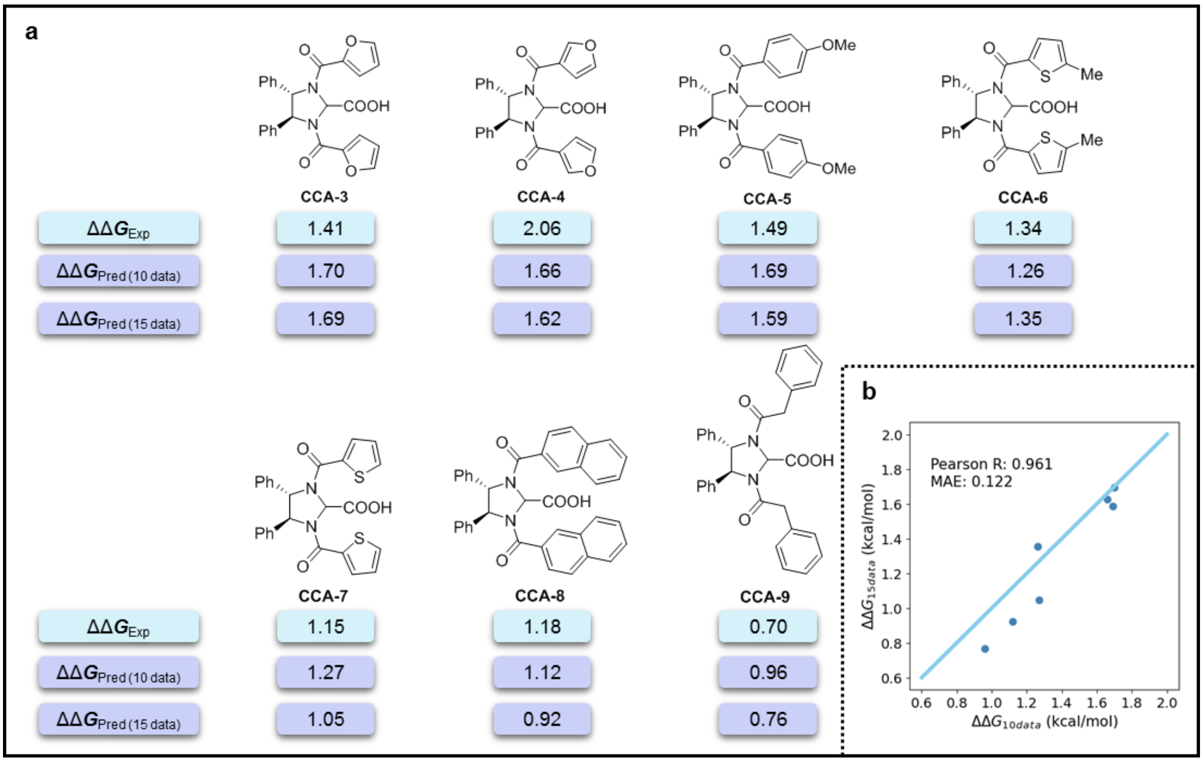

Supplementary Fig. 8 | Comparison of the knowledge transfer predictions using 10 and 15 data input. **a,** Examples of the delta learning predictions using 10 and 15 data input. **b,** Regression comparison of the delta learning predictions using 10 and 15 data input.

9. Additional validation in substrate 1k

In order to further verify the effectiveness of our strategy, we experimentally determined the enantioselectivities of 10 CCAs using substrate **1k** (Supplementary Fig. 9), and applied the knowledge transfer approach using these data. The base model trained on the 59 data of the source reaction performed poorly in predicting the enantioselectivities of substrate **1k** (Pearson R = 0.354, Supplementary Fig. 10a). This is consistent with the base model results for substrate **1a**, indicating the perturbations of the structure-performance relationship between the source and target reactions. Subsequently, we trained a new delta learning model by using the difference between the predicted values of the base model and the experimental selectivities of **1k** as the target (D_{exp} = $\Delta\Delta G_{pred}$ - $\Delta\Delta G_{exp}$). The delta model significantly improved the prediction performance for substrate **1k**, and the LOO prediction results are present in Supplementary Fig. 10b (Pearson R = 0.971). These additional results further validated the effectiveness of the proposed delta learning method.

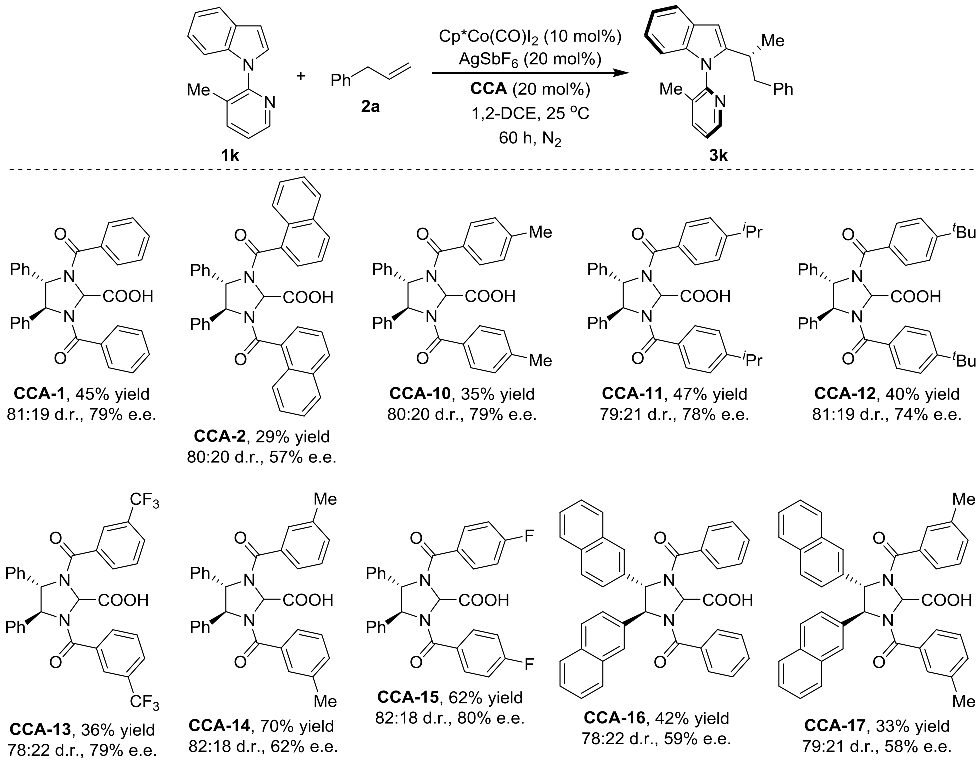

Supplementary Fig. 9 | Experimental enantioselectivities of the ten representative CCAs on substrate 1k.

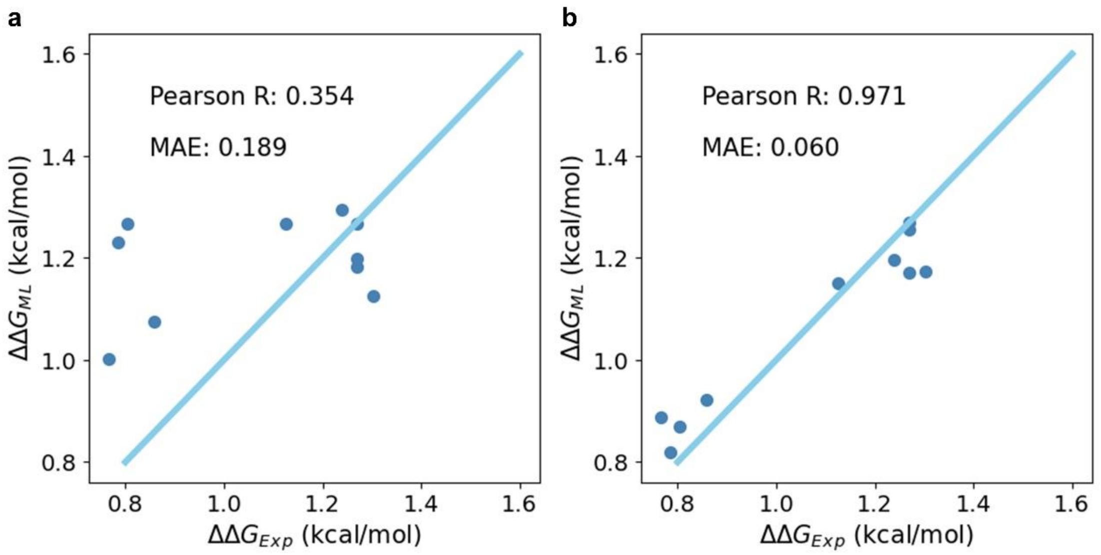

Supplementary Fig. 10 | Prediction performances of the base model and the delta learning model on the substrate 1k. The models are trained using the leave-one-out (LOO) data splitting.

10. Predictions without the knowledge transfer approach

To confirm the effectiveness of the knowledge transfer model, we also tested the model predictions of the **rxn2** without the delta learning.

10.1 Model selection

Combing the data of **rxn1** (59 data) and **rxn2** (10 data), the naïve training using the entire 108-dimenisonal descriptors was performed to select the optimal algorithm. In the 10-fold cross-validation, eleven regression algorithms (Bagging Regression, Decision Trees, Extra-Trees, Gradient Boosting, k-Nearest Neighbors Regression, Kernel Ridge Regression, Linear Support Vector Regression, Random Forest Regression, Ridge, Support Vector Regression, and XGBoost) were tested, and the Pearson Rs are shown in Supplementary Table 7. Random Forest was identified as the optimal algorithm.

Supplementary Table 7. Pearson R and MAE of the enantioselectivity predictions using all data of **rxn1** and **rxn2** in 10-fold cross-validation with various algorithms.

Model	MAE (kcal/mol)	Pearson R
Bagging	0.195	0.843
Decision Tree	0.235	0.785
Extra-Trees	0.179	0.856
Gradient Boosting	0.199	0.839
k-Nearest Neighbors Regression	0.229	0.781
Kernel Ridge Regression	0.196	0.842
Linear Support Vector Regression	0.203	0.850
Random Forest	0.184	0.858
Ridge	0.195	0.844
Support Vector Regression	0.214	0.831
XGBoost	0.208	0.825

10.2 Descriptor selection

Using Linear Random Forest which is the optimal algorithm for 10-fold cross validation, descriptor selection was performed. For each round of descriptor addition, the highest performance-improving descriptor was selected and added until no further improvement can be achieved. The regression performances for all data of **rxn1** and **rxn2** prior to and after the descriptor selection are shown in Supplementary Fig. 11. Python script is available at GitHub: https://github.com/Shuwen-Li/FindBestChiralAcid.

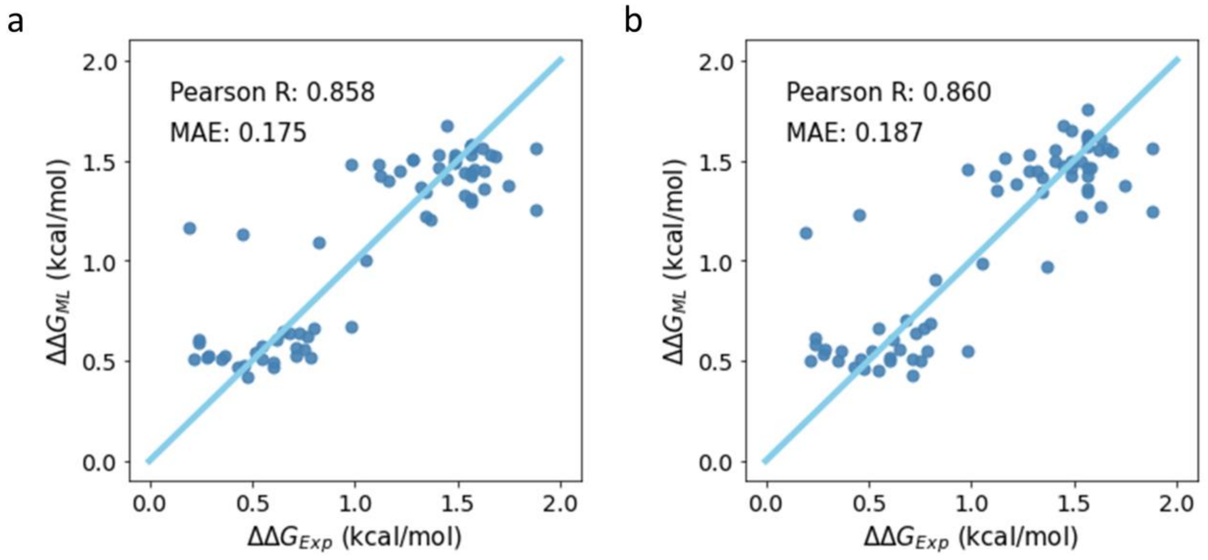

Supplementary Fig. 11 | Regression performances for all data of rxn1 and rxn2 prior to the descriptor selection (a) and after descriptor selection (b).

10.3 Predicted enantioselectivities of the 360 candidate acids

Using the naïve training model, the predictions of the 360 candidate chiral carboxylic acids were significantly varied as compared to the prediction results of the knowledge transfer model. For the predicted top-3 acids using the knowledge transfer model, the naïve training model gave a predicted rank of 154, 98 and 6 respectively, which is significantly different as compared to the results of the knowledge transfer model (Supplementary Fig. 12). Especially, the acid **CCA-4** whose experimental enantioselectivity is 94% is inaccurately predicted to be 81%, which highlights the predictive ability of the knowledge transfer model.

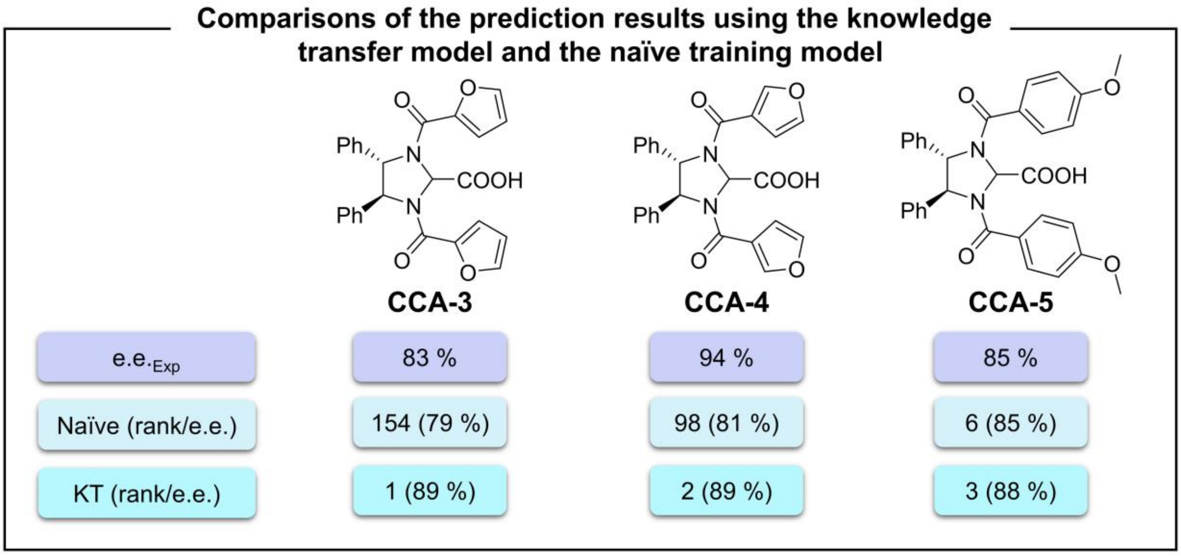

Supplementary Fig. 12 | Comparisons of the prediction results using the knowledge transfer model and the naïve training model.

11. Data and code availability

All the involved codes and data in this study were freely available at https://github.com/Shuwen-Li/FindBestChiralAcid.

12. Synthesis of chiral carboxylic acid[1-3]

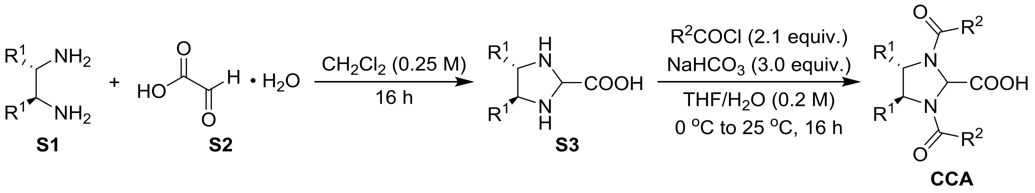

To a solution of (1*S*,2*S*)-disubstituted ethylenediamine **S1** (1.0 mmol) in CH_2Cl_2 (4.0 mL) was added glyoxylic acid monohydrate **S2** (1.0 mmol, 92.1 mg) under vigorous stirring. After 16 hours, the solvent was removed to afford (4*S*,5*S*)-4,5-disubstituted imidazolidine-2-carboxylic acid **S3**, which was used in the next step without further purification.

To a solution of (4*S*,5*S*)-4,5-disubstituted imidazolidine-2-carboxylic acid **S3** in THF/H_2O (1:1, 5.0 mL) at 0 °C, $NaHCO_3$ (3.0 mmol, 252.0 mg) and acid chloride (2.1 mmol) were added, and the reaction was allowed to reach ambient temperature. After 16 hours, the reaction mixture was diluted with CH_2Cl_2 (10.0 mL) and washed with aqueous HCl solution (1.0 M, 10.0 mL). The aqueous phase was extracted with CH_2Cl_2 (10.0 mL ×3). The combined organic phase was washed with brine, dried over Na_2SO_4, filtered and the solvent was removed *in vacuo*. The crude mixture was purified via flash chromatography (CH_2Cl_2 100% to CH_2Cl_2: CH_3OH = 90:10) to afford the desired chiral carboxylic acid.

(4*S*,5*S*)-1,3-Dibenzoyl-4,5-diphenylimidazolidine-2-carboxylic acid (CCA-1)

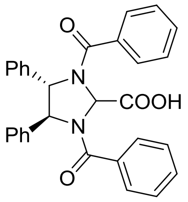

General procedure for the preparation of chiral carboxylic acid was followed using (1*S*,2*S*)-1,2-diphenylethane-1,2-diamine (1.0 mmol, 212.1 mg) and benzoyl chloride (2.1 mmol, 294.0 mg) to afford **CCA-1** (312.3 mg, 66% yield over 2 steps) as a white solid. **^{1}H NMR (400 MHz, CD_3CO_2D)** δ 7.53 – 7.25 (m, 18H), 7.23 – 7.07 (m, 3H), 5.59 (s, 1H), 5.34 (s, 1H). **^{13}C NMR (101 MHz, CD_3CO_2D)** δ 174.08 (C_q), 173.45 (C_q), 172.44 (C_q), 141.83 (C_q), 139.91 (C_q), 136.51 (C_q), 135.49 (C_q), 132.58 (CH), 132.30 (CH), 130.73 (CH), 130.49 (CH), 129.99 (CH), 129.94 (CH), 129.87 (CH), 128.65 (CH), 128.53 (CH), 128.06 (CH), 127.47 (CH), 74.70 (CH), 73.50 (CH), 71.73 (CH). The analytical data are in accordance with these reported in literature[1].

(4*S*,5*S*)-1,3-Di(1-naphthoyl)-4,5-diphenylimidazolidine-2-carboxylic acid (CCA-2)

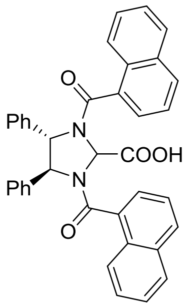

General procedure for the preparation of chiral carboxylic acid was followed using (1*S*,2*S*)-1,2-diphenylethane-1,2-diamine (1.0 mmol, 212.1 mg) and 1-naphthoyl chloride (2.1 mmol, 399.0 mg) to afford **CCA-2** (367.0 mg, 64% yield over 2 steps) as a white solid. **^{1}H NMR (400 MHz, CD_3CO_2D)** δ 8.23 – 7.65 (m, 7H), 7.61 – 7.02 (m, 16H), 6.85 (s, 2H), 5.03 (s, 1H), 4.87 (s, 1H). **^{13}C NMR (101 MHz, CD_3CO_2D)** δ 173.21 (C_q), 172.80 (C_q), 171.87 (C_q), 141.38 (C_q), 139.74 (C_q), 139.69 (C_q), 134.97 (C_q), 134.88 (C_q), 134.74 (C_q), 133.71 (C_q), 133.30 (C_q), 131.83 (CH), 131.80 (CH), 130.45 (CH), 130.19 (CH), 129.99 (CH), 129.90 (CH), 129.83 (CH), 129.79 (CH), 129.65 (CH), 129.62 (CH), 129.48 (CH), 128.72 (CH), 128.20 (CH), 128.04 (CH), 127.95 (CH), 127.38 (CH), 127.01 (CH), 126.24 (CH), 126.15 (CH), 126.10 (CH), 75.02 (CH), 74.14 (CH), 72.29 (CH). The analytical data are in accordance with these reported in literature[1].

(4*S*,5*S*)-1,3-Di(furan-2-carbonyl)-4,5-diphenylimidazolidine-2-carboxylic acid (CCA-3)

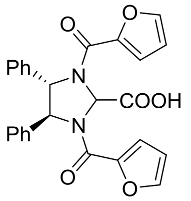

General procedure for the preparation of chiral carboxylic acid was followed using (1*S*,2*S*)-1,2-diphenylethane-1,2-diamine (1.0 mmol, 212.1 mg) and furan-2-carbonyl chloride (2.1 mmol, 273.0 mg) to afford **CCA-3** (300.0 mg, 66% yield over 2 steps) as a white solid. **^{1}H NMR (400 MHz, CD_3CO_2D)** δ 7.67 – 7.52 (m, 5H), 7.49 – 7.32 (m, 7H), 7.34 – 7.21 (m, 3H), 6.51 (s, 2H), 6.18 (s, 1H), 5.92 (s, 1H). **^{13}C NMR (101 MHz, CD_3CO_2D)** δ 172.23 (C_q), 161.01 (C_q), 160.19 (C_q), 148.44 (C_q), 148.06 (CH), 147.44 (C_q), 147.38 (CH), 143.05 (C_q), 139.92 (C_q), 130.78 (CH), 130.36 (CH), 129.76 (CH), 129.74 (CH), 127.81 (CH), 127.02 (CH), 121.50 (CH), 120.64 (CH), 113.66 (CH), 73.90 (CH), 71.40 (CH), 70.28 (CH). **IR** (ATR, cm^{-1}) γ 3108, 3060, 3029, 3007,

1633, 1575, 1476, 1393, 761, 700. **HRMS** (ESI) m/z $(M–H)^-$: calculated for $(C_{26}H_{19}N_2O_6)^-$: 455.1249, found: 455.1243. $[\alpha]_D^{25}$ = –126.0 (c = 0.20, $CHCl_3$).

(4*S*,5*S*)-1,3-Di(furan-3-carbonyl)-4,5-diphenylimidazolidine-2-carboxylic acid (CCA-4)

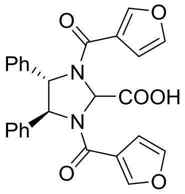

General procedure for the preparation of chiral carboxylic acid was followed using (1*S*,2*S*)-1,2-diphenylethane-1,2-diamine (1.0 mmol, 212.1 mg) and furan-3-carbonyl chloride (2.1 mmol, 273.0 mg) to afford **CCA-4** (329.2 mg, 72% yield over 2 steps) as a white solid. **^{1}H NMR (400 MHz, CD_3CO_2D)** δ 7.84 (s, 2H), 7.63 (d, *J* = 7.3 Hz, 2H), 7.54 – 7.31 (m, 10H), 7.12 (s, 1H), 6.64 (s, 1H), 6.60 (s, 1H), 5.90 (s, 1H), 5.70 (s, 1H). **^{13}C NMR (101 MHz, CD_3CO_2D)** δ 172.13 (C_q), 166.96 (C_q), 165.97 (C_q), 147.87 (CH), 147.50 (CH), 145.49 (CH), 145.16 (CH), 142.49 (C_q), 139.54 (C_q), 131.11 (CH), 130.67 (CH), 130.04 (CH), 127.70 (CH), 127.04 (CH), 122.13 (C_q), 121.24 (C_q), 111.59 (CH), 111.44 (CH), 74.78 (CH), 72.72 (CH), 70.25 (CH). **IR** (ATR, cm^{-1}) *γ* 3110, 3064, 3033, 3007, 1637, 1562, 1503, 1359, 1161, 752, 700, 598. **HRMS** (ESI) m/z $(M–H)^-$: calculated for $(C_{26}H_{19}N_2O_6)^-$: 455.1249, found: 455.1238. $[\alpha]_D^{25}$ = –118.2 (c = 0.45, $CHCl_3$).

(4*S*,5*S*)-1,3-Bis(4-methoxybenzoyl)-4,5-diphenylimidazolidine-2-carboxylic acid (CCA-5)

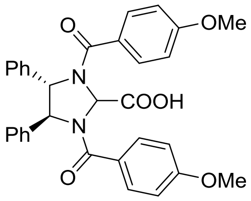

General procedure for the preparation of chiral carboxylic acid was followed using (1*S*,2*S*)-1,2-diphenylethane-1,2-diamine (1.0 mmol, 212.1 mg) and 4-methoxybenzoyl chloride (2.1 mmol, 357.0 mg) to afford **CCA-5** (353.9 mg, 66% yield over 2 steps) as a white solid. **^{1}H NMR (400 MHz, CD_3CO_2D)** δ 7.58 – 7.09 (m, 15H), 6.83 (d, *J* = 8.2 Hz, 2H), 6.78 (d, *J* = 8.3 Hz, 2H), 5.63 (s, 1H), 5.37 (s, 1H), 3.79 (s, 3H), 3.75 (s, 3H). **^{13}C NMR (101 MHz, CD_3CO_2D)** δ 174.06 (C_q), 173.37 (C_q), 172.58 (C_q), 163.73 (C_q), 163.41 (C_q), 142.07 (C_q), 140.18 (C_q), 130.97 (CH), 130.90 (CH), 130.71 (CH), 130.53 (CH), 129.91 (CH), 129.81 (CH), 128.50 (C_q), 128.02 (CH),

127.33 (CH), 127.19 (C_q), 115.24 (CH), 74.56 (CH), 73.58 (CH), 71.85 (CH), 56.36 (CH_3), 56.34 (CH_3). **IR** (ATR, cm^{-1}) γ 3060, 3029, 1637, 1600, 1376, 1174, 761, 699. **HRMS** (ESI) m/z (M–H)$^-$: calculated for $(C_{32}H_{27}N_2O_6)^-$: 535.1875, found: 535.1866. $[\alpha]_D^{25}$ = –51.7 (c = 0.71, $CHCl_3$).

(4*S*,5*S*)-1,3-Bis(5-methylthiophene-2-carbonyl)-4,5-diphenylimidazolidine-2-carboxylic acid (CCA-6)

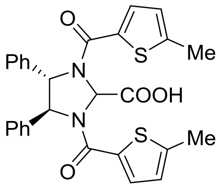

General procedure for the preparation of chiral carboxylic acid was followed using (1*S*,2*S*)-1,2-diphenylethane-1,2-diamine (1.0 mmol, 212.1 mg) and 5-methylthiophene-2-carbonyl chloride (2.1 mmol, 335.9 mg) to afford **CCA-6** (182.0 mg, 35% yield over 2 steps) as a white solid. **^{1}H NMR (400 MHz, CD_3CO_2D)** δ 7.69 (d, *J* = 7.6 Hz, 2H), 7.53 (t, *J* = 7.5 Hz, 2H), 7.49 – 7.37 (m, 6H), 7.37 – 7.27 (m, 2H), 7.27 – 7.17 (m, 1H), 6.69 (s, 1H), 6.63 (s, 1H), 6.07 (s, 1H), 5.83 (s, 1H), 2.43 (s, 3H), 2.41 (s, 3H). **^{13}C NMR (101 MHz, CD_3CO_2D)** δ 172.13 (C_q), 166.18 (C_q), 164.80 (C_q), 150.04 (C_q), 148.76 (C_q), 142.53 (C_q), 139.57 (C_q), 135.14 (C_q), 134.25 (C_q), 133.94 (CH), 131.03 (CH), 130.68 (CH), 130.04 (CH), 129.95 (CH), 128.10 (CH), 127.75 (CH), 127.15 (CH), 75.12 (CH), 73.47 (CH), 71.15 (CH), 15.90 (CH_3), 15.87 (CH_3). The analytical data are in accordance with these reported in literature[3].

(4*S*,5*S*)-4,5-Diphenyl-1,3-di(thiophene-2-carbonyl)imidazolidine-2-carboxylic acid (CCA-7)

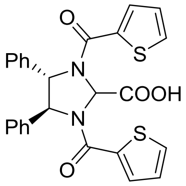

General procedure for the preparation of chiral carboxylic acid was followed using (1*S*,2*S*)-1,2-diphenylethane-1,2-diamine (1.0 mmol, 212.1 mg) and thiophene-2-carbonyl chloride (2.1 mmol, 306.5 mg) to afford **CCA-7** (205.5 mg, 42% yield over 2 steps) as a white solid. **^{1}H NMR (400 MHz, CD_3CO_2D)** δ 7.68 (d, *J* = 7.6 Hz, 2H), 7.66 – 7.55 (m, 2H), 7.57 – 7.48 (m, 3H), 7.45 (d, *J* = 7.2 Hz, 1H), 7.42 – 7.24 (m, 7H), 6.99 (t, *J* = 4.4 Hz, 1H), 6.94 (t, *J* = 4.5 Hz,

1H), 6.07 (s, 1H), 5.83 (s, 1H). **^{13}C NMR (101 MHz, CD_3CO_2D)** δ 172.18 (C_q), 166.41 (C_q), 165.13 (C_q), 142.35 (C_q), 139.50 (C_q), 137.70 (C_q), 136.78 (C_q), 134.30 (CH), 133.40 (CH), 133.17 (CH), 132.96 (CH), 131.05 (CH), 130.73 (CH), 130.12 (CH), 130.00 (CH), 129.36 (CH), 128.99 (CH), 127.78 (CH), 127.21 (CH), 75.47 (CH), 73.54 (CH), 71.41 (CH). **IR** (ATR, cm^{-1}) γ 3111, 3063, 3029, 3006, 1635, 1521, 1417, 1360, 849, 801, 699. **HRMS** (ESI) m/z $(M–H)^-$: calculated for $(C_{26}H_{19}N_2O_4S_2)^-$: 487.0792, found: 487.0787. **$[\alpha]_D^{25}$** = –137.0 (c = 1.00, $CHCl_3$).

(4*S*,5*S*)-1,3-Di(2-naphthoyl)-4,5-diphenylimidazolidine-2-carboxylic acid (CCA-8)

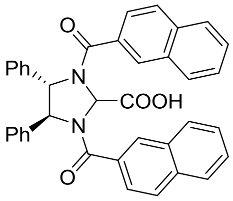

General procedure for the preparation of chiral carboxylic acid was followed using (1*S*,2*S*)-1,2-diphenylethane-1,2-diamine (1.0 mmol, 212.1 mg) and 2-naphthoyl chloride (2.1 mmol, 399.0 mg) to afford **CCA-8** (450.1 mg, 78% yield over 2 steps) as a white solid. **^{1}H NMR (400 MHz, CD_3CO_2D)** δ 7.97 – 7.73 (m, 7H), 7.69 – 7.35 (m, 12H), 7.32 – 7.13 (m, 6H), 5.68 (s, 1H), 5.42 (s, 1H). **^{13}C NMR (101 MHz, CD_3CO_2D)** δ 174.22 (C_q), 173.45 (C_q), 172.50 (C_q), 141.74 (C_q), 140.05 (C_q), 135.81 (C_q), 135.76 (C_q), 133.94 (C_q), 133.75 (C_q), 133.75 (C_q), 132.57 (C_q), 130.68 (CH), 130.51 (CH), 130.15 (CH), 130.09 (CH), 129.98 (CH), 129.91 (CH), 129.85 (CH), 129.43 (CH), 129.32 (CH), 129.27 (CH), 129.19 (CH), 129.17 (CH), 128.54 (CH), 128.40 (CH), 128.17 (CH), 127.53 (CH), 125.38 (CH), 125.14 (CH), 75.04 (CH), 73.70 (CH), 71.67 (CH). **IR** (ATR, cm^{-1}) γ 3060, 3029, 1639, 1598, 1473, 824, 757, 699. **HRMS** (ESI) m/z $(M–H)^-$: calculated for $(C_{38}H_{27}N_2O_4)^-$: 575.1976, found: 575.1974. **$[\alpha]_D^{25}$** =–46.0 (c = 0.52, $CHCl_3$).

(4*S*,5*S*)-4,5-Diphenyl-1,3-bis(2-phenylacetyl)imidazolidine-2-carboxylic acid (CCA-9)

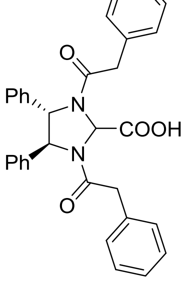

General procedure for the preparation of chiral carboxylic acid was followed using (1*S*,2*S*)-1,2-diphenylethane-1,2-diamine (1.0 mmol, 212.1 mg) and 2-phenylacetyl chloride (2.1 mmol, 323.4 mg) to afford **CCA-9** (251.6 mg, 50% yield over 2 steps) as a white solid. **^{1}H NMR (400 MHz, CD_3CO_2D)** δ 7.55 – 7.12 (m, 16H), 7.11 – 6.87 (m, 4H), 6.66 – 6.47 (m, 1H), 5.57 (d, *J* = 39.5 Hz, 1H), 5.34 (d, *J* = 37.2 Hz, 1H), 4.47 – 3.25 (m, 4H). **^{13}C NMR (101 MHz, CD_3CO_2D)** δ 174.25 (C_q), 173.92 (C_q), 173.53 (C_q), 142.65 (C_q), 141.97 (C_q), 140.03 (C_q), 139.34 (C_q), 131.36 (CH), 131.15 (CH), 131.04 (CH), 130.88 (CH), 130.53 (CH), 130.12 (CH), 128.99 (CH), 128.69 (CH), 128.40 (CH), 128.14 (CH), 127.61 (CH), 127.04 (CH), 76.76 (CH), 74.00 (CH), 73.06 (CH), 42.49 (CH_2), 42.02 (CH_2). The analytical data are in accordance with these reported in literature[1].

(4*S*,5*S*)-1,3-Bis(4-methylbenzoyl)-4,5-diphenylimidazolidine-2-carboxylic acid (CCA-10)

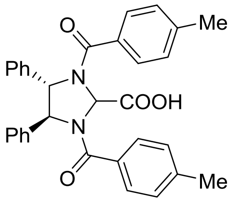

General procedure for the preparation of chiral carboxylic acid was followed using (1*S*,2*S*)-1,2-diphenylethane-1,2-diamine (1.0 mmol, 212.1 mg) and 4-methylbenzoyl chloride (2.1 mmol, 323.4 mg) to afford **CCA-10** (352.0 mg, 70% yield over 2 steps) as a white solid. **^{1}H NMR (400 MHz, CD_3CO_2D)** δ 7.60 – 7.42 (m, 5H), 7.43 – 7.25 (m, 7H), 7.25 – 7.01 (m, 7H), 5.65 (s, 1H), 5.37 (s, 1H), 2.35 (s, 3H), 2.32 (s, 3H). **^{13}C NMR (101 MHz, CD_3CO_2D)** δ 174.40 (C_q), 173.68 (C_q), 172.50 (C_q), 143.45 (C_q), 142.96 (C_q), 142.05 (C_q), 140.02 (C_q), 133.57 (C_q), 132.39 (C_q), 130.74 (CH), 130.59 (CH), 130.55 (CH), 130.54 (CH), 129.95 (CH), 129.86 (CH), 128.86 (CH), 128.78 (CH), 127.99 (CH), 127.35 (CH), 74.64 (CH), 73.48 (CH), 71.68 (CH), 22.09 (CH_3), 22.02 (CH_3). The analytical data are in accordance with these reported in literature[2].

(4*S*,5*S*)-1,3-Bis(4-isopropylbenzoyl)-4,5-diphenylimidazolidine-2-carboxylic acid (CCA-11)

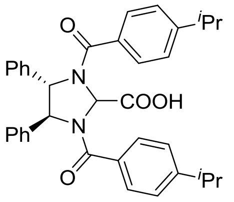

General procedure for the preparation of chiral carboxylic acid was followed using (1*S*,2*S*)-1,2-diphenylethane-1,2-diamine (1.0 mmol, 212.1 mg) and 4-isopropylbenzoyl chloride (2.1 mmol, 382.3 mg) to afford **CCA-11** (414.5 mg, 74% yield over 2 steps) as a white solid. **^{1}H NMR (400 MHz, CD_3CO_2D)** δ 7.54 – 7.42 (m, 5H), 7.42 – 7.29 (m, 7H), 7.27 – 7.08 (m, 7H), 5.63 (s, 1H), 5.41 (s, 1H), 3.06 – 2.83 (m, *J* = 7.3 Hz, 2H), 1.29 (d, *J* = 6.9 Hz, 6H), 1.26 (d, *J* = 6.9 Hz, 6H). **^{13}C NMR (101 MHz, CD_3CO_2D)** δ 174.21 (C_q), 173.61 (C_q), 172.66 (C_q), 154.00 (C_q), 153.60 (C_q), 141.84 (C_q), 140.06 (C_q), 134.00 (C_q), 132.89 (C_q), 130.65 (CH), 130.47 (CH), 129.87 (CH), 129.79 (CH), 128.93 (CH), 128.11 (CH), 127.97 (CH), 127.88 (CH), 127.58 (CH), 74.83 (CH), 73.57 (CH), 71.99 (CH), 35.58 (CH), 35.52 (CH), 24.66 (CH_3), 24.60 (CH_3), 24.56 (CH_3). **IR** (ATR, cm^{-1}) *γ* 3061, 3029, 2960, 2927, 2870, 1649, 1383, 841, 767, 698. **HRMS** (ESI) m/z (M–H)$^-$: calculated for $(C_{36}H_{35}N_2O_4)^-$: 559.2602, found: 559.2626. $[\alpha]_D^{25}$ = –57.5 (c = 0.57, $CHCl_3$).

(4*S*,5*S*)-1,3-Bis(4-(*tert*-butyl)benzoyl)-4,5-diphenylimidazolidine-2-carboxylic acid (CCA-12)

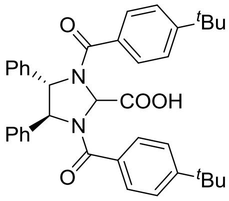

General procedure for the preparation of chiral carboxylic acid was followed using (1*S*,2*S*)-1,2-diphenylethane-1,2-diamine (1.0 mmol, 212.1 mg) and 4-(*tert*-butyl)benzoyl chloride (2.1 mmol, 411.7 mg) to afford **CCA-12** (399.0 mg, 68% yield over 2 steps) as a white solid. **^{1}H NMR (400 MHz, CD_3CO_2D)** δ 7.56 – 7.24 (m, 16H), 7.23 – 7.02 (m, 3H), 5.59 (s, 1H), 5.37 (s, 1H), 1.33 (s, 9H), 1.30 (s, 9H). **^{13}C NMR (101 MHz, CD_3CO_2D)** δ 174.30 (C_q), 173.60 (C_q), 172.59 (C_q), 156.17 (C_q), 155.73 (C_q), 141.94 (C_q), 140.08 (C_q), 133.73 (C_q), 132.66 (C_q), 130.65 (CH), 130.45 (CH), 129.85 (CH), 129.75 (CH), 128.67 (CH), 128.13 (CH), 127.62 (CH), 126.87 (CH), 126.76 (CH), 74.68 (CH), 73.53 (CH), 71.95 (CH), 36.09 (C_q), 36.06 (C_q), 32.01 (CH_3), 31.96 (CH_3). **IR** (ATR, cm^{-1}) *γ* 3062, 3031, 2964, 2904, 2865, 1650, 1391, 752, 698. **HRMS** (ESI) m/z (M–H)$^-$: calculated for $(C_{38}H_{39}N_2O_4)^-$: 587.2915, found: 587.2916. $[\alpha]_D^{25}$ = –62.7 (c = 0.55, $CHCl_3$).

(4*S*,5*S*)-4,5-Diphenyl-1,3-bis(3-(trifluoromethyl)benzoyl)imidazolidine-2-carboxylic acid (CCA-13)

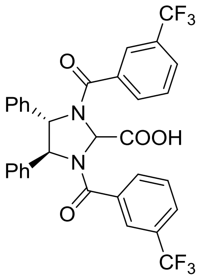

General procedure for the preparation of chiral carboxylic acid was followed using (1*S*,2*S*)-1,2-diphenylethane-1,2-diamine (1.0 mmol, 212.1 mg) and 3-(trifluoromethyl)benzoyl chloride (2.1 mmol, 436.8 mg) to afford **CCA-13** (367.1 mg, 60% yield over 2 steps) as a white solid. **^{1}H NMR (400 MHz, CD_3CO_2D)** δ 7.74 – 7.57 (m, 5H), 7.57 – 7.42 (m, 3H), 7.39 – 7.25 (m, 8H), 7.19 – 7.11 (m, 2H), 7.07 (s, 1H), 5.47 (d, *J* = 4.4 Hz, 1H), 5.26 (s, 1H). **^{13}C NMR (101 MHz, CD_3CO_2D)** δ 172.91 (C_q), 172.22 (C_q), 171.65 (C_q), 140.66 (C_q), 139.40 (C_q), 137.79 (C_q), 136.87 (C_q), 132.22 (q, *J* = 4.2 Hz, C_q), 132.03 (CH), 131.97 (CH), 131.79 (q, *J* = 4.1 Hz, C_q), 130.93 (CH), 130.84 (CH), 130.71 (CH), 130.51 (CH), 130.08 (CH), 128.77 (q, *J* = 3.4 Hz, CH), 128.52 (q, *J* = 3.1 Hz, CH), 128.32 (CH), 128.09 (CH), 127.08 (q, *J* = 6.5 Hz, C_q), 125.50 (CH), 125.47 (CH), 123.48 (q, *J* = 6.5 Hz, C_q), 75.69 (CH), 74.09 (CH), 72.36 (CH). **^{19}F NMR (282 MHz, CD_3CO_2D)** δ -63.65, -63.73. The analytical data are in accordance with these reported in literature[2].

(4*S*,5*S*)-1,3-Bis(3-methylbenzoyl)-4,5-diphenylimidazolidine-2-carboxylic acid (CCA-14)

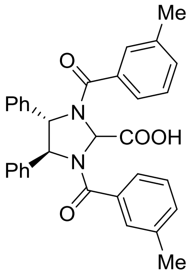

General procedure for the preparation of chiral carboxylic acid was followed using (1*S*,2*S*)-1,2-diphenylethane-1,2-diamine (1.0 mmol, 212.1 mg) and 3-methylbenzoyl chloride (2.1 mmol, 323.4 mg) to afford **CCA-14** (352.8 mg, 70% yield over 2 steps) as a white solid. **^{1}H NMR (400 MHz, CD_3CO_2D)** δ 7.54 – 7.26 (m, 9H), 7.27 – 7.10 (m, 8H), 7.08 (s, 2H), 5.56 (s, 1H), 5.29 (s, 1H), 2.25 (s, 3H), 2.19 (s, 3H). **^{13}C NMR (101 MHz, CD_3CO_2D)** δ 174.31 (C_q), 173.59 (C_q), 172.68 (C_q), 141.92 (C_q), 140.12 (C_q), 139.95 (C_q), 139.87 (C_q), 136.48 (C_q), 135.49 (C_q),

133.17 (CH), 132.93 (CH), 130.69 (CH), 130.43 (CH), 129.93 (CH), 129.88 (CH), 129.86 (CH), 129.80 (CH), 129.23 (CH), 129.20 (CH), 128.11 (CH), 127.54 (CH), 125.66 (CH), 125.46 (CH), 74.75 (CH), 73.58 (CH), 71.59 (CH), 21.83 (CH_3), 21.80 (CH_3). The analytical data are in accordance with these reported in literature[1].

(4*S*,5*S*)-1,3-Bis(4-fluorobenzoyl)-4,5-diphenylimidazolidine-2-carboxylic acid (CCA-15)

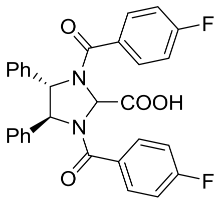

General procedure for the preparation of chiral carboxylic acid was followed using (1*S*,2*S*)-1,2-diphenylethane-1,2-diamine (1.0 mmol, 212.1 mg) and 4-fluorobenzoyl chloride (2.1 mmol, 331.8 mg) to afford **CCA-15** (312.0 mg, 61% yield over 2 steps) as a white solid. **^{1}H NMR (400 MHz, CD_3CO_2D)** δ 7.50 – 7.33 (m, 9H), 7.38 – 7.23 (m, 3H), 7.23 – 7.12 (m, 2H), 7.10 – 6.94 (m, 5H), 5.53 (d, J = 3.5 Hz, 1H), 5.32 (s, 1H). **^{13}C NMR (101 MHz, CD_3CO_2D)** δ 171.36 (C_q), 170.89 (C_q), 170.72 (C_q), 165.53 (d, *J* = 13.4 Hz, C_q), 162.22 (d, *J* = 12.4 Hz, C_q), 139.72 (C_q), 138.02 (C_q), 131.20 (d, *J* = 3.3 Hz, C_q), 131.14 (d, *J* = 3.3 Hz, C_q), 129.57 (CH), 129.45 (CH), 128.98 (CH), 128.77 (CH), 128.26 (CH), 128.19 (CH), 126.44 (CH), 125.97 (CH), 115.38 (d, *J* = 6.0 Hz, CH), 115.08 (d, *J* = 5.9 Hz, CH), 73.39 (CH), 72.01 (CH), 70.30 (CH). **^{19}F NMR (282 MHz, CD_3CO_2D)** δ -109.36, -109.82. The analytical data are in accordance with these reported in literature[2].

(4*S*,5*S*)-1,3-Dibenzoyl-4,5-di(naphthalen-2-yl)imidazolidine-2-carboxylic acid (CCA-16)

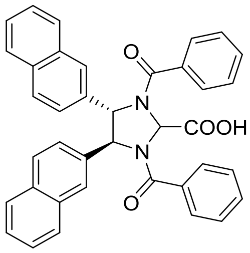

General procedure for the preparation of chiral carboxylic acid was followed using (1*S*,2*S*)-1,2-di(naphthalen-2-yl)ethane-1,2-diamine (1.0 mmol, 312.2 mg) and benzoyl chloride (2.1 mmol, 294.0 mg) to afford **CCA-16** (275.0 mg, 48% yield over 2 steps) as a white solid. **^{1}H NMR (400 MHz, CD_3CO_2D)** δ 8.04 – 7.84 (m, 6H), 7.82 – 7.50 (m, 8H), 7.49 – 7.12 (m, 11H), 5.89 (s,

1H), 5.63 (s, 1H). **^{13}C NMR (101 MHz, CD_3CO_2D)** δ 174.21 (C_q), 173.58 (C_q), 173.06 (C_q), 138.85 (C_q), 137.25 (C_q), 136.52 (C_q), 135.47 (C_q), 134.85 (C_q), 134.81 (C_q), 134.78 (C_q), 134.67 (C_q), 132.58 (CH), 132.24 (CH), 131.05 (CH), 130.76 (CH), 129.92 (CH), 129.83 (CH), 129.48 (CH), 129.31 (CH), 129.27 (CH), 128.64 (CH), 128.54 (CH), 128.33 (CH), 128.26 (CH), 128.23 (CH), 128.20 (CH), 127.69 (CH), 127.39 (CH), 125.56 (CH), 124.64 (CH), 75.30 (CH), 73.76 (CH), 71.93 (CH). **IR** (ATR, cm^{-1}) γ 3059, 3030, 1649, 1600, 1386, 752, 699, 478. **HRMS** (ESI) m/z $(M–H)^-$: calculated for $(C_{38}H_{27}N_2O_4)^-$: 575.1976, found: 575.1979. $[\alpha]_D^{25}$ = –98.8 (c = 0.60, $CHCl_3$).

(4*S*,5*S*)-1,3-Bis(3-methylbenzoyl)-4,5-di(naphthalen-2-yl)imidazolidine-2-carboxylic acid (CCA-17)

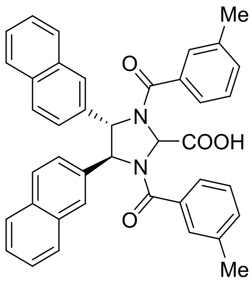

General procedure for the preparation of chiral carboxylic acid was followed using (1*S*,2*S*)-1,2-di(naphthalen-2-yl)ethane-1,2-diamine (1.0 mmol, 312.2 mg) and 3-methylbenzoyl chloride (2.1 mmol, 323.4 mg) to afford **CCA-17** (254.1 mg, 42% yield over 2 steps) as a white solid. **^{1}H NMR (400 MHz, CD_3CO_2D)** δ 8.05 – 7.67 (m, 8H), 7.65 – 7.37 (m, 6H), 7.35 – 6.92 (m, 9H), 5.84 (s, 1H), 5.57 (s, 1H), 2.03 (s, 3H), 1.96 (s, 3H). **^{13}C NMR (101 MHz, CD_3CO_2D)** δ 174.39 (C_q), 173.70 (C_q), 173.18 (C_q), 139.87 (C_q), 139.77 (C_q), 138.64 (C_q), 137.34 (C_q), 136.44 (C_q), 135.47 (C_q), 134.81 (C_q), 134.76 (C_q), 134.68 (C_q), 133.06 (CH), 132.82 (CH), 130.95 (CH), 130.68 (CH), 129.82 (CH), 129.77 (CH), 129.49 (CH), 129.28 (CH), 129.23 (CH), 129.15 (CH), 128.29 (CH), 128.21 (CH), 128.18 (CH), 127.92 (CH), 127.67 (CH), 125.66 (CH), 125.55 (CH), 125.33 (CH), 124.83 (CH), 75.48 (CH), 73.95 (CH), 72.03 (CH), 21.63 (CH_3), 21.61 (CH_3). **IR** (ATR, cm^{-1}) γ 3056, 3022, 1644, 1601, 1386, 753, 478. **HRMS** (ESI) m/z $(M-H)^-$: calculated for $(C_{40}H_{31}N_2O_4)^-$: 603.2289, found: 603.2314. $[\alpha]_D^{25}$ = –144.0 (c = 0.68, $CHCl_3$).

(4*S*,5*S*)-1,3-Bis(3,5-dimethylbenzoyl)-4,5-diphenylimidazolidine-2-carboxylic acid (CCA-18)

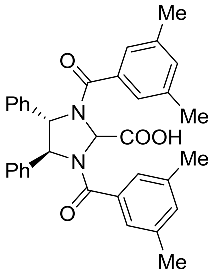

General procedure for the preparation of chiral carboxylic acid was followed using (1*S*,2*S*)-1,2-diphenylethane-1,2-diamine (1.0 mmol, 212.1 mg) and 3,5-dimethylbenzoyl chloride (2.1 mmol, 352.9 mg) to afford **CCA-18** (329.0 mg, 62% yield over 2 steps) as a white solid. **^{1}H NMR (400 MHz, CD_3CO_2D)** δ 7.51 – 7.29 (m, 8H), 7.20 (s, 2H), 7.09 – 6.84 (m, 7H), 5.56 (s, 1H), 5.28 (s, 1H), 2.23 (s, 6H), 2.17 (s, 6H). **^{13}C NMR (101 MHz, CD_3CO_2D)** δ 174.62 (C_q), 173.71 (C_q), 172.52 (C_q), 141.96 (C_q), 140.32 (C_q), 139.86 (C_q), 139.78 (C_q), 136.39 (C_q), 135.42 (C_q), 133.94 (CH), 133.74 (CH), 130.67 (CH), 130.42 (CH), 129.86 (CH), 129.77 (CH), 128.17 (CH), 127.61 (CH), 126.34 (CH), 126.23 (CH), 74.85 (CH), 73.57 (CH), 71.41 (CH), 21.78 (CH_3), 21.75 (CH_3). The analytical data are in accordance with these reported in literature[2].

(4*S*,5*S*)-1,3-Bis(3,5-di-*tert*-butylbenzoyl)-4,5-diphenylimidazolidine-2-carboxylic acid (CCA-19)

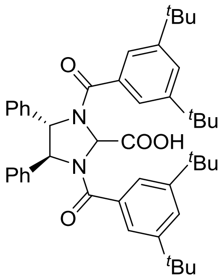

General procedure for the preparation of chiral carboxylic acid was followed using (1*S*,2*S*)-1,2-diphenylethane-1,2-diamine (1.0 mmol, 212.1 mg) and 3,5-di-*tert*-butylbenzoyl chloride (2.1 mmol, 529.5 mg) to afford **CCA-19** (496.9 mg, 71% yield over 2 steps) as a pale yellow solid. **^{1}H NMR (400 MHz, CD_3CO_2D)** δ 7.58 – 7.41 (m, 7H), 7.39 – 7.24 (m, 5H), 7.23 – 6.94 (m, 5H), 5.48 (s, 1H), 5.21 (s, 1H), 1.27 (s, 18H), 1.16 (s, 18H). **^{13}C NMR (101 MHz, CD_3CO_2D)** δ 175.36 (C_q), 174.46 (C_q), 172.56 (C_q), 152.93 (C_q), 152.76 (C_q), 142.22 (C_q), 140.32 (C_q), 135.90 (C_q), 134.74 (C_q), 130.80 (CH), 130.59 (CH), 129.94 (CH), 127.82 (CH), 127.25 (CH), 126.62 (CH), 125.88 (CH), 122.91 (CH), 122.77 (CH), 73.96 (CH), 73.68 (CH), 72.64 (CH),

36.10 (C_q), 32.14 (CH_3), 31.99 (CH_3). **IR** (ATR, cm^{-1}) γ 3060, 3029, 2965, 2905, 2867, 1649, 1595, 1455, 1394, 1364, 1249, 699. **HRMS** (ESI) m/z $(M–H)^-$: calculated for $(C_{46}H_{55}N_2O_4)^-$: 699.4167, found: 699.4173. $[\alpha]_D^{25}$ = –34.0 (c = 0.50, $CHCl_3$).

(4*S*,5*S*)-1,3-Bis(4-iodobenzoyl)-4,5-diphenylimidazolidine-2-carboxylic acid (CCA-20)

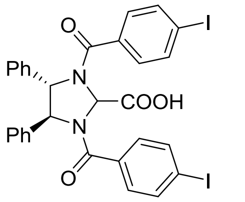

General procedure for the preparation of chiral carboxylic acid was followed using (1*S*,2*S*)-1,2-diphenylethane-1,2-diamine (1.0 mmol, 212.1 mg) and 4-iodobenzoyl chloride (2.1 mmol, 558.4 mg) to afford **CCA-20** (420.3 mg, 58% yield over 2 steps) as a white solid. **^{1}H NMR (400 MHz, CD_3CO_2D)** δ 7.72 – 7.53 (m, 4H), 7.42 – 7.25 (m, 8H), 7.20 – 6.97 (m, 7H), 5.50 (s, 1H), 5.28 (s, 1H). **^{13}C NMR (101 MHz, CD_3CO_2D)** δ 173.01 (C_q), 172.55 (C_q), 172.46 (C_q), 141.25 (C_q), 139.56 (C_q), 139.21 (CH), 139.15 (CH), 136.04 (C_q), 134.98 (C_q), 130.72 (CH), 130.51 (CH), 130.27 (CH), 130.18 (CH), 130.00 (CH), 129.97 (CH), 128.15 (CH), 127.76 (CH), 98.70 (C_q), 98.32 (C_q), 75.04 (CH), 73.68 (CH), 71.84 (CH). **IR** (ATR, cm^{-1}) γ 3064, 3029, 3007, 1642, 1600, 1577, 1494, 1447, 1377, 1346, 756, 696. **HRMS** (ESI) m/z $(M–H)^-$: calculated for $(C_{30}H_{21}I_2N_2O_4)^-$: 726.9596, found: 726.9596. $[\alpha]_D^{25}$ = –25.8 (c = 1.00, $CHCl_3$).

(4*S*,5*S*)-4,5-Diphenyl-1,3-bis(4-(trifluoromethyl)benzoyl)imidazolidine-2-carboxylic acid (CCA-21)

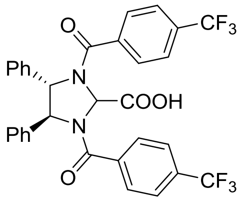

General procedure for the preparation of chiral carboxylic acid was followed using (1*S*,2*S*)-1,2-diphenylethane-1,2-diamine (1.0 mmol, 212.1 mg) and 4-(trifluoromethyl)benzoyl chloride (2.1 mmol, 436.8 mg) to afford **CCA-21** (378.6 mg, 62% yield over 2 steps) as a white solid. **^{1}H NMR (400 MHz, CD_3CO_2D)** δ 7.63 – 7.41 (m, 8H), 7.35 – 7.21 (m, 8H), 7.18 – 6.98 (m, 3H), 5.44 (s, 1H), 5.27 (s, 1H). **^{13}C NMR (101 MHz, CD_3CO_2D)** δ 172.83 (C_q), 172.33 (C_q), 171.90 (C_q), 140.73 (C_q), 140.49 (C_q), 139.52 (C_q), 139.40 (C_q), 133.64 (q, *J* = 32.5 Hz, C_q),

133.33 (q, *J* = 32.8 Hz, C_q), 130.70 (CH), 130.53 (CH), 130.06 (CH), 129.15 (CH), 129.14 (CH), 128.40 (CH), 128.27 (CH), 127.21 (q, *J* = 8.7 Hz, C_q), 126.93 (CH), 126.86 (CH), 126.83 (CH), 123.60 (q, *J* = 9.0 Hz, C_q), 75.74 (CH), 74.06 (CH), 72.29 (CH). **^{19}F NMR (282 MHz, CD_3CO_2D)** δ -63.82, -63.90. **IR** (ATR, cm^{-1}) *γ* 3024, 2921, 1702, 1350, 1047, 752, 667, 583. **HRMS** (ESI) m/z $(M–H)^-$: calculated for $(C_{32}H_{21}F_6N_2O_4)^-$: 611.1411, found: 611.1409. **$[\alpha]_D^{25}$** = –53.5 (c = 1.00, $CHCl_3$).

13. Synthesis of indole substrates[4,5]

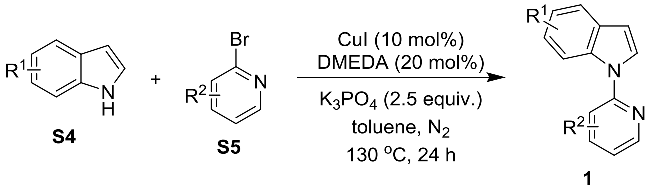

To a flame-dried and N_2-purged Schlenk tube were added indole derivative **S4** (10.0 mmol), 2-bromopyridine derivative **S5** (10.0 mmol), CuI (1.0 mmol, 190.5 mg) and K_3PO_4 (25.0 mmol, 5.30 g). The vial was then sealed, purged and backfilled with N_2 three times before adding DMEDA (2.0 mmol, 215 μL) and toluene (20.0 mL) at room temperature. The resulting mixture was refluxed at 130 °C under nitrogen atmosphere for 24 hours. After cooling to room temperature, the mixture was filtered and concentrated under reduced pressure. The residue was purified by column chromatography on silica gel (*n*-hexane: ethyl acetate = 10:1) to afford the desired indole substrate **1**.

1-(1*H*-Indol-1-yl)isoquinoline (1a)

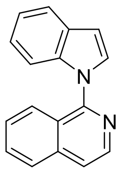

General procedure for the preparation of indole substrate was followed using 1*H*-indole (10.0 mmol, 1.17 g) and 1-bromoisoquinoline (10.0 mmol, 2.07 g) to afford **1a** (2.20 g, 90% yield) as a white solid. M.p.: 93-94 °C. **^{1}H NMR (400 MHz, CDCl$_3$)** δ 8.54 (d, *J* = 5.6 Hz, 1H), 8.01 – 7.92 (m, 2H), 7.82 – 7.69 (m, 3H), 7.63 (d, *J* = 3.2 Hz, 1H), 7.56 (t, *J* = 7.7 Hz, 1H), 7.41 (d, *J* = 8.2 Hz, 1H), 7.29 – 7.17 (m, 2H), 6.84 (d, *J* = 3.0 Hz, 1H). **^{13}C NMR (101 MHz, CDCl$_3$)** δ 151.00 (C_q), 141.63 (CH), 138.65 (C_q), 137.11 (C_q), 130.87 (CH), 129.43 (C_q), 128.88 (CH), 127.91 (CH), 127.14 (CH), 125.82 (CH), 123.79 (C_q), 122.71 (CH), 121.10 (CH), 120.97 (CH), 120.48 (CH), 111.96 (CH), 104.65 (CH). **IR** (ATR, cm^{-1}) *γ* 3051, 1624, 1585, 1561, 1455, 1406, 1364, 1217, 825, 741. **HRMS** (ESI) m/z $(M+H)^+$: calculated for $(C_{17}H_{13}N_2)^+$: 245.1073, found: 245.1077.

1-(4-Fluoro-1*H*-indol-1-yl)isoquinoline (1b)

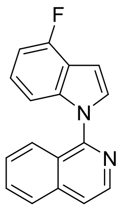

General procedure for the preparation of indole substrate was followed using 4-fluoro-1*H*-indole (10.0 mmol, 1.35 g) and 1-bromoisoquinoline (10.0 mmol, 2.07 g) to afford **1b** (2.41 g, 92% yield) as a white solid. M.p.: 106-108 °C. **^{1}H NMR (300 MHz, CDCl$_3$)** δ 8.52 (d, *J* = 5.7 Hz, 1H), 7.97 (d, *J* = 8.3 Hz, 1H), 7.90 (d, *J* = 8.5 Hz, 1H), 7.82 – 7.71 (m, 2H), 7.64 – 7.53 (m, 2H), 7.19 – 7.04 (m, 2H), 6.97 – 6.78 (m, 2H). **^{13}C NMR (75 MHz, CDCl$_3$)** δ 156.45 (d, *J* = 247.1 Hz, C_q), 150.66 (C_q), 141.64 (CH), 139.55 (d, *J* = 10.7 Hz, C_q), 138.73 (C_q), 131.07 (CH), 128.84 (CH), 128.20 (CH), 127.27 (CH), 125.56 (CH), 123.83 (C_q), 123.31 (d, *J* = 7.6 Hz, CH), 120.97 (CH), 118.46 (d, *J* = 22.6 Hz, C_q), 108.09 (d, *J* = 3.7 Hz, CH), 105.83 (d, *J* = 18.8 Hz, CH), 100.50 (CH). **^{19}F NMR (282 MHz, CDCl$_3$)** δ -122.05. **IR** (ATR, cm^{-1}) γ 3054, 1627, 1577, 1561, 1486, 1442, 1360, 1242, 1217, 999, 931, 827, 742. **HRMS** (ESI) m/z $(M+H)^+$: calculated for $(C_{17}H_{12}FN_2)^+$: 263.0979, found: 263.0987.

1-(4-Methyl-1*H*-indol-1-yl)isoquinoline (1c)

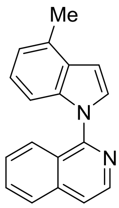

General procedure for the preparation of indole substrate was followed using 4-methyl-1*H*-indole (10.0 mmol, 1.31 g) and 1-bromoisoquinoline (10.0 mmol, 2.07 g) to afford **1c** (2.22 g, 86% yield) as a brown oil. **^{1}H NMR (300 MHz, CDCl$_3$)** δ 8.52 (d, *J* = 5.6 Hz, 1H), 7.95 (dd, *J* = 8.2, 3.0 Hz, 2H), 7.74 (dd, *J* = 16.3, 6.7 Hz, 2H), 7.61 (d, *J* = 2.9 Hz, 1H), 7.56 (t, *J* = 7.7 Hz, 1H), 7.20 (d, *J* = 8.2 Hz, 1H), 7.11 (t, *J* = 7.6 Hz, 1H), 7.02 (d, *J* = 7.0 Hz, 1H), 6.84 (d, *J* = 2.8 Hz, 1H), 2.66 (s, 3H). **^{13}C NMR (75 MHz, CDCl$_3$)** δ 151.20 (C_q), 141.71 (CH), 138.68 (C_q), 136.92 (C_q), 130.89 (CH), 130.56 (C_q), 129.24 (C_q), 128.33 (CH), 127.90 (CH), 127.15 (CH), 125.97 (CH), 123.90 (C_q), 122.82 (CH), 121.18 (CH), 120.51 (CH), 109.58 (CH), 103.11 (CH),

18.93 (CH_3). **IR** (ATR, cm^{-1}) γ 3052, 2916, 1560, 1485, 1454, 1427, 1402, 1359, 1161, 925, 826, 748. **HRMS** (ESI) m/z $(M+H)^+$: calculated for $(C_{18}H_{15}N_2)^+$: 259.1230, found: 259.1233.

1-(5-Fluoro-1*H*-indol-1-yl)isoquinoline (1d)

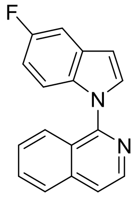

General procedure for the preparation of indole substrate was followed using 5-fluoro-1*H*-indole (10.0 mmol, 1.35 g) and 1-bromoisoquinoline (10.0 mmol, 2.07 g) to afford **1d** (2.17 g, 83% yield) as a yellow solid. M.p.: 70-72 °C. **^{1}H NMR (300 MHz, $CDCl_3$)** δ 8.50 (d, *J* = 5.7 Hz, 1H), 8.00 – 7.90 (m, 2H), 7.81 – 7.70 (m, 2H), 7.63 (d, *J* = 3.3 Hz, 1H), 7.59 (ddd, *J* = 8.3, 6.9, 1.3 Hz, 1H), 7.39 – 7.30 (m, 2H), 6.94 (td, *J* = 9.1, 2.5 Hz, 1H), 6.76 (dd, *J* = 3.4, 0.8 Hz, 1H). **^{13}C NMR (75 MHz, $CDCl_3$)** δ 158.62 (d, *J* = 235.8 Hz, C_q), 150.80 (C_q), 141.63 (CH), 138.80 (C_q), 133.67 (C_q), 131.03 (CH), 130.44 (CH), 129.93 (d, *J* = 10.1 Hz, C_q), 128.14 (CH), 127.30 (CH), 125.64 (CH), 123.71 (C_q), 120.68 (CH), 112.82 (d, *J* = 9.6 Hz, CH), 111.05 (d, *J* = 26.1 Hz, CH), 106.00 (d, *J* = 23.6 Hz, CH), 104.59 (d, *J* = 4.4 Hz, CH). **^{19}F NMR (282 MHz, $CDCl_3$)** δ -123.51. **IR** (ATR, cm^{-1}) γ 3056, 1620, 1584, 1561, 1470, 1446, 1405, 1208, 1176, 1113, 952, 824, 750, 720. **HRMS** (ESI) m/z $(M+H)^+$: calculated for $(C_{17}H_{12}FN_2)^+$: 263.0979, found: 263.0986.

1-(5-Bromo-1*H*-indol-1-yl)isoquinoline (1e)

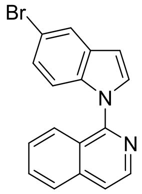

General procedure for the preparation of indole substrate was followed using 5-bromo-1*H*-indole (10.0 mmol, 1.95 g) and 1-bromoisoquinoline (10.0 mmol, 2.07 g) to afford **1e** (2.74 g, 85% yield) as a yellow solid. M.p.: 112-114 °C. **^{1}H NMR (300 MHz, $CDCl_3$)** δ 8.51 (d, *J* = 5.7 Hz, 1H), 7.97 (d, *J* = 8.3 Hz, 1H), 7.91 – 7.83 (m, 2H), 7.81 – 7.72 (m, 2H), 7.64 – 7.54 (m, 2H), 7.33 – 7.19 (m, 2H), 6.74 (dd, *J* = 3.3, 0.5 Hz, 1H). **^{13}C NMR (75 MHz, $CDCl_3$)** δ 150.56 (C_q), 141.66 (CH), 138.79 (C_q), 135.83 (C_q), 131.14 (C_q), 131.12 (CH), 130.04 (CH), 128.23 (CH),

127.34 (CH), 125.63 (CH), 125.56 (CH), 123.75 (C_q), 123.67 (CH), 120.90 (CH), 114.24 (C_q), 113.50 (CH), 104.12 (CH). **IR** (ATR, cm^{-1}) γ 3051, 1560, 1499, 1449, 1401, 1352, 1198, 1173, 871, 825, 797, 751, 721, 678, 420. **HRMS** (ESI) m/z $(M+H)^+$: calculated for $(C_{17}H_{12}BrN_2)^+$: 323.0178, found: 323.0179.

1-(5-Methyl-1*H*-indol-1-yl)isoquinoline (1f)

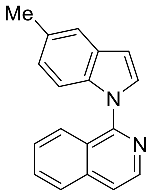

General procedure for the preparation of indole substrate was followed using 5-methyl-1*H*-indole (10.0 mmol, 1.31 g) and 1-bromoisoquinoline (10.0 mmol, 2.07 g) to afford **1f** (2.34 g, 91% yield) as a yellow oil. **^{1}H NMR (300 MHz, $CDCl_3$)** δ 8.50 (d, *J* = 5.7 Hz, 1H), 8.00 – 7.91 (m, 2H), 7.79 – 7.68 (m, 2H), 7.61 – 7.49 (m, 3H), 7.25 (d, *J* = 8.4 Hz, 1H), 7.02 (dd, *J* = 8.4, 1.6 Hz, 1H), 6.72 (dd, *J* = 3.3, 0.7 Hz, 1H), 2.48 (s, 3H). **^{13}C NMR (75 MHz, $CDCl_3$)** δ 151.21 (C_q), 141.70 (CH), 138.74 (C_q), 135.54 (C_q), 130.89 (CH), 130.32 (C_q), 129.78 (C_q), 128.98 (CH), 127.87 (CH), 127.17 (CH), 126.03 (CH), 124.29 (CH), 123.80 (C_q), 120.83 (CH), 120.35 (CH), 111.70 (CH), 104.34 (CH), 21.56 (CH_3). **IR** (ATR, cm^{-1}) γ 3052, 3012, 2915, 2863, 1560, 1475, 1406, 1351, 1216, 1177, 826, 797, 750, 718. **HRMS** (ESI) m/z $(M+H)^+$: calculated for $(C_{18}H_{15}N_2)^+$: 259.1230, found: 259.1237.

1-(5-Methoxy-1*H*-indol-1-yl)isoquinoline (1g)

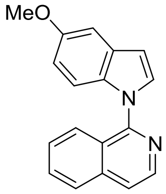

General procedure for the preparation of indole substrate was followed using 5-methoxy-1*H*-indole (10.0 mmol, 1.47 g) and 1-bromoisoquinoline (10.0 mmol, 2.07 g) to afford **1g** (2.41 g, 88% yield) as a brown oil. **^{1}H NMR (300 MHz, $CDCl_3$)** δ 8.49 (d, *J* = 5.7 Hz, 1H), 8.05 – 7.92 (m, 2H), 7.75 (ddd, *J* = 8.2, 6.9, 1.2 Hz, 1H), 7.71 – 7.67 (m, 1H), 7.61 – 7.53 (m, 2H), 7.31 (d, *J* = 9.0 Hz, 1H), 7.19 (d, *J* = 2.4 Hz, 1H), 6.86 (dd, *J* = 9.0, 2.4 Hz, 1H), 6.73 (dd, *J* = 3.3, 0.8 Hz, 1H), 3.89 (s, 3H). **^{13}C NMR (75 MHz, $CDCl_3$)** δ 155.04 (C_q), 151.10 (C_q), 141.64 (CH), 138.78

(C_q), 132.32 (C_q), 130.90 (CH), 130.09 (C_q), 129.47 (CH), 127.91 (CH), 127.20 (CH), 125.93 (CH), 123.67 (C_q), 120.31 (CH), 112.88 (CH), 112.71 (CH), 104.52 (CH), 102.90 (CH), 55.98 (CH_3). **IR** (ATR, cm^{-1}) γ 3052, 3013, 2915, 2829, 1621, 1584, 1559, 1472, 1447, 1030, 943, 827, 751, 719. **HRMS** (ESI) m/z $(M+H)^+$: calculated for $(C_{18}H_{15}N_2O)^+$: 275.1179, found: 275.1185.

1-(5-(Trifluoromethyl)-1*H*-indol-1-yl)isoquinoline (1h)

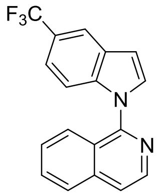

General procedure for the preparation of indole substrate was followed using 5-(trifluoromethyl)-1*H*-indole (10.0 mmol, 1.85 g) and 1-bromoisoquinoline (10.0 mmol, 2.07 g) to afford **1h** (2.78 g, 89% yield) as a white solid. M.p.: 87-88 °C. **^{1}H NMR (300 MHz, $CDCl_3$)** δ 8.53 (d, *J* = 5.7 Hz, 1H), 8.04 (s, 1H), 7.99 (d, *J* = 8.3 Hz, 1H), 7.86 (dq, *J* = 8.6, 1.0 Hz, 1H), 7.84 – 7.72 (m, 2H), 7.69 (d, *J* = 3.4 Hz, 1H), 7.60 (ddd, *J* = 8.3, 6.9, 1.2 Hz, 1H), 7.43 (d, *J* = 1.2 Hz, 2H), 6.89 (d, *J* = 3.4 Hz, 1H). **^{13}C NMR (75 MHz, $CDCl_3$)** δ 150.37 (C_q), 141.69 (CH), 138.78 (C_q), 138.41 (C_q), 131.19 (CH), 130.61 (CH), 128.79 (C_q), 128.37 (CH), 127.39 (CH), 125.36 (CH), 125.34 (q, *J* = 271.5 Hz, C_q), 123.80 (C_q), 123.42 (q, *J* = 31.9 Hz, C_q) 121.17 (CH), 119.55 (q, *J* = 3.4 Hz, CH), 118.89 (q, *J* = 4.1 Hz, CH), 112.29 (CH), 105.20 (CH). **^{19}F NMR (282 MHz, $CDCl_3$)** δ -60.49. **IR** (ATR, cm^{-1}) γ 3057, 1481, 1448, 1401, 1327, 1106, 1090, 1054, 827, 810, 726. **HRMS** (ESI) m/z $(M+H)^+$: calculated for $(C_{18}H_{12}F_3N_2)^+$: 313.0947, found: 313,0954.

Methyl 1-(isoquinolin-1-yl)-1*H*-indole-5-carboxylate (1i)

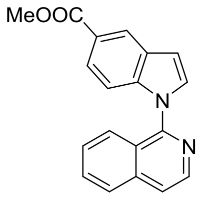

General procedure for the preparation of indole substrate was followed using methyl 1*H*-indole-5-carboxylate (10.0 mmol, 1.75 g) and 1-bromoisoquinoline (10.0 mmol, 2.07 g) to afford **1i** (2.87 g, 95% yield) as a yellow oil. **^{1}H NMR (300 MHz, $CDCl_3$)** δ 8.53 (d, *J* = 5.7 Hz, 1H), 8.49 (d, *J* = 1.2 Hz, 1H), 7.98 (d, *J* = 8.3 Hz, 1H), 7.88 (td, *J* = 8.8, 1.2 Hz, 2H), 7.81 – 7.74 (m, 2H),

7.65 (d, *J* = 3.4 Hz, 1H), 7.59 (ddd, *J* = 8.3, 6.9, 1.2 Hz, 1H), 7.35 (d, *J* = 8.7 Hz, 1H), 6.89 (d, *J* = 3.4 Hz, 1H), 3.95 (s, 3H). **^{13}C NMR (75 MHz, CDCl$_3$)** δ 168.10 (C_q), 150.49 (C_q), 141.69 (CH), 139.60 (C_q), 138.78 (C_q), 131.18 (CH), 130.28 (CH), 129.03 (C_q), 128.32 (CH), 127.36 (CH), 125.51 (CH), 124.14 (CH), 124.09 (CH), 123.84 (C_q), 123.11 (C_q), 121.13 (CH), 111.66 (CH), 105.72 (CH), 52.07 (CH_3). **IR** (ATR, cm^{-1}) *γ* 3056, 2948, 1706, 1432, 1401, 1274, 1197, 1095, 907, 752, 733. **HRMS** (ESI) m/z $(M+H)^+$: calculated for $(C_{19}H_{15}N_2O_2)^+$: 303.1128, found: 303.1132.

1-(6-Methyl-1*H*-indol-1-yl)isoquinoline (1j)

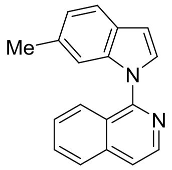

General procedure for the preparation of indole substrate was followed using 6-methyl-1*H*-indole (10.0 mmol, 1.31 g) and 1-bromoisoquinoline (10.0 mmol, 2.07 g) to afford **1j** (2.10 g, 81% yield) as a brown solid. M.p.: 82-83 °C. **^{1}H NMR (300 MHz, CDCl$_3$)** δ 8.52 (d, *J* = 5.7 Hz, 1H), 8.02 – 7.90 (m, 2H), 7.80 – 7.69 (m, 2H), 7.65 – 7.54 (m, 2H), 7.51 (d, *J* = 3.4 Hz, 1H), 7.21 (dd, *J* = 1.5, 0.8 Hz, 1H), 7.09 – 6.99 (m, 1H), 6.75 (dd, *J* = 3.3, 0.8 Hz, 1H), 2.41 (s, 3H). **^{13}C NMR (75 MHz, CDCl$_3$)** δ 151.24 (C_q), 141.67 (CH), 138.74 (C_q), 137.57 (C_q), 132.70 (C_q), 130.92 (CH), 128.37 (CH), 127.93 (CH), 127.23 (C_q), 127.16 (CH), 125.97 (CH), 123.97 (C_q), 122.79 (CH), 120.73 (CH), 120.42 (CH), 111.87 (CH), 104.50 (CH), 21.99 (CH_3). **IR** (ATR, cm^{-1}) *γ* 3053, 2917, 2857, 1624, 1560, 1455, 1435, 1357, 1212, 924, 803, 747, 717, 672. **HRMS** (ESI) m/z $(M+H)^+$: calculated for $(C_{18}H_{15}N_2)^+$: 259.1230, found: 259.1237.

1-(3-Methylpyridin-2-yl)-1*H*-indole (1k)

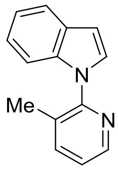

General procedure for the preparation of indole substrate was followed using 1*H*-indole (10.0 mmol, 1.17 g) and 2-bromo-3-methylpyridine (10.0 mmol, 1.71 g) to afford **1k** (1.97 g, 95% yield) as a brown oil. **^{1}H NMR (300 MHz, CDCl$_3$)** δ 8.50 (dd, *J* = 4.7, 1.3 Hz, 1H),

7.82 – 7.66 (m, 2H), 7.38 (d, *J* = 3.3 Hz, 1H), 7.34 – 7.08 (m, 4H), 6.73 (dd, *J* = 3.3, 0.6 Hz, 1H), 2.24 (s, 3H). **^{13}C NMR (75 MHz, $CDCl_3$)** δ 151.12 (C_q), 147.09 (CH), 140.62 (CH), 136.27 (C_q), 129.05 (C_q), 128.93 (C_q), 127.60 (CH), 122.92 (CH), 122.52 (CH), 121.08 (CH), 120.51 (CH), 111.30 (CH), 103.80 (CH), 18.01 (CH_3). **IR** (ATR, cm^{-1}) *γ* 3050, 1573, 1459, 1424, 1329, 1213, 1141, 742. **HRMS** (ESI) m/z $(M+H)^+$: calculated for $(C_{14}H_{13}N_2)^+$: 209.1073, found: 209.1075.

14. General procedure for cobalt(III)-catalyzed asymmetric C–H alkylation

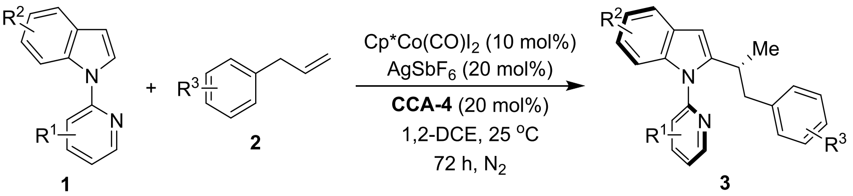

To a flame-dried and N_2-purged Schlenk tube were added indole substrate **1** (0.1 mmol), Cp*Co(CO)I_2 (0.01 mmol, 10 mol%, 4.8 mg), $AgSbF_6$ (0.02 mmol, 20 mol%, 6.9 mg), and chiral carboxylic acid **CCA-4** (0.02 mmol, 20 mol%, 9.1 mg). The vial was then sealed, purged and backfilled with N_2 three times before adding alkene substrate **2** (0.3 mmol) and 1,2-dichloroethane (0.5 mL) at room temperature. The resulting solution was then stirred at 25 °C for 72 hours. The resulting solution was diluted with CH_2Cl_2 (2.0 mL), filtered through a pad of Celite (eluted with CH_2Cl_2), then the solvent was removed *in vacuo*. The diastereomeric ratio was determined by ^{1}H NMR analysis of the crude reaction mixture. The residue was purified by column chromatography on silica gel (*n*-hexane: ethyl acetate = 15:1) to afford the desired product **3**.

15. Characterization data of products

(*R*$_a$, *R*)-1-(2-(1-Phenylpropan-2-yl)-1*H*-indol-1-yl)isoquinoline (3a)

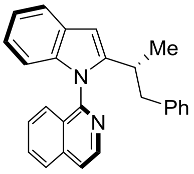

The general procedure was followed using 1-(1*H*-indol-1-yl)isoquinoline (0.1 mmol, 24.4 mg) and allylbenzene (0.3 mmol, 35.4 mg) to afford **3a** (32.6 mg, 90% yield, >95:5 d.r., b:l >95:5) as a colorless oil. **^{1}H NMR (400 MHz, CDCl$_3$)** δ 8.62 (d, *J* = 5.7 Hz, 1H), 7.97 (d, *J* = 8.3 Hz, 1H), 7.83 (d, *J* = 5.7 Hz, 1H), 7.73 (ddd, *J* = 8.2, 6.7, 1.4 Hz, 1H), 7.67 (d, *J* = 7.8 Hz, 1H), 7.45 – 7.39 (m, 1H), 7.36 (dd, *J* = 8.5, 1.2 Hz, 1H), 7.13 (td, *J* = 7.5, 7.1, 1.0 Hz, 1H), 7.08 – 6.96 (m, 4H), 6.74 – 6.65 (m, 3H), 6.64 (s, 1H), 3.33 – 3.16 (m, 1H), 2.77 (dd, *J* = 13.4, 5.8 Hz, 1H), 2.51 (dd, *J* = 13.5, 8.5 Hz, 1H), 1.30 (d, *J* = 6.9 Hz, 3H). **^{13}C NMR (101 MHz, CDCl$_3$)** δ 150.68 (C$_q$), 147.36 (C$_q$), 142.09 (CH), 140.15 (C$_q$), 138.75 (C$_q$), 138.32 (C$_q$), 131.06 (CH), 129.02 (CH), 128.49 (CH), 128.46 (C$_q$), 128.12 (CH), 127.06 (CH), 126.10 (C$_q$), 125.99 (CH), 125.95 (CH),

121.65 (CH), 121.60 (CH), 120.48 (CH), 120.27 (CH), 110.65 (CH), 100.48 (CH), 43.63 (CH_2), 33.63 (CH), 19.84 (CH_3). **IR** (ATR, cm^{-1}) γ 3057, 3028, 2962, 2927, 1561, 1496, 1456, 1406, 829, 785, 749, 698. **HRMS** (ESI) m/z $(M+H)^+$: calculated for $(C_{26}H_{23}N_2)^+$: 363.1856, found: 363.1856; $[\alpha]_D^{25}$ = +164.2 (c = 0.31, $CHCl_3$); The product was analyzed by HPLC to determine the enantiomeric excess: 94% e.e. (CHIRALPAK IC-3, *n*-hexane/*i*-PrOH = 95/5, flow rate: 1.0 mL/min, T = 20 °C, 250 nm), t_R (minor) = 9.40 min, t_R (major) = 15.30 min.

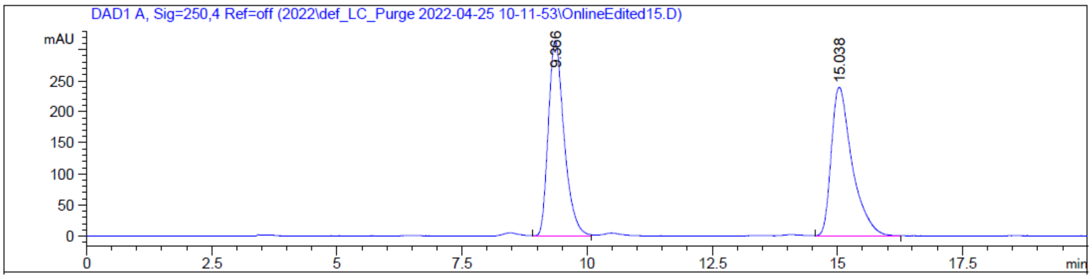

Peak #	RetTime [min]	Type	Width [min]	Area [mAU*s]	Height [mAU]	Area %
1	9.366	MF R	0.3610	6871.35352	314.15460	49.8958
2	15.038	BV R	0.4166	6900.05859	239.13156	50.1042

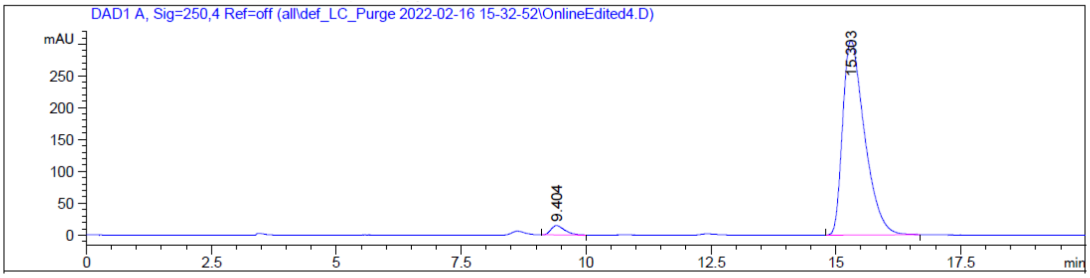

Peak #	RetTime [min]	Type	Width [min]	Area [mAU*s]	Height [mAU]	Area %
1	9.404	BB	0.2316	283.38852	14.72120	2.9469
2	15.303	BV R	0.4503	9333.09570	305.61261	97.0531

(*R*$_a$, *R*)-1-(4-Fluoro-2-(1-phenylpropan-2-yl)-1*H*-indol-1-yl)isoquinoline (3b)

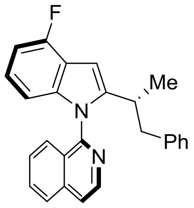

The general procedure was followed using 1-(4-fluoro-1*H*-indol-1-yl)isoquinoline (0.1 mmol,

26.2 mg) and allylbenzene (0.3 mmol, 35.4 mg) to afford **3b** (34.1 mg, 90% yield, >95:5 d.r., b:l >95:5) as a colorless oil. **^{1}H NMR (400 MHz, CDCl$_3$)** δ 8.63 (d, *J* = 5.7 Hz, 1H), 7.98 (d, *J* = 8.3 Hz, 1H), 7.86 (d, *J* = 5.7 Hz, 1H), 7.74 (ddd, *J* = 8.2, 6.9, 1.2 Hz, 1H), 7.42 (ddd, *J* = 8.1, 6.9, 1.1 Hz, 1H), 7.33 – 7.25 (m, 1H), 7.10 – 7.01 (m, 3H), 6.99 – 6.85 (m, 1H), 6.81 (ddd, *J* = 10.0, 7.9, 0.7 Hz, 1H), 6.74 (s, 1H), 6.71 – 6.65 (m, 2H), 6.48 (d, *J* = 8.0 Hz, 1H), 3.25 – 3.05 (m, 1H), 2.79 (dd, *J* = 13.4, 6.0 Hz, 1H), 2.54 (dd, *J* = 13.5, 8.3 Hz, 1H), 1.31 (d, *J* = 6.9 Hz, 3H). **^{13}C NMR (101 MHz, CDCl$_3$)** δ 156.09 (d, *J* = 246.2 Hz, C_q), 150.23 (C_q), 147.38 (C_q), 142.07 (CH), 141.07 (d, *J* = 11.3 Hz, C_q), 139.96 (C_q), 138.33 (C_q), 131.17 (CH), 128.99 (CH), 128.68 (CH), 128.17 (CH), 127.12 (CH), 126.07 (CH), 125.96 (C_q), 125.58 (CH), 122.10 (d, *J* = 7.6 Hz, CH), 121.94 (CH), 117.35 (d, *J* = 22.6 Hz, C_q), 106.78 (d, *J* = 3.5 Hz, CH), 105.41 (d, *J* = 19.0 Hz, CH), 96.19 (CH), 43.52 (CH_2), 33.75 (CH), 20.04 (CH_3). **^{19}F NMR (282 MHz, CDCl$_3$)** δ -122.99. **IR** (ATR, cm^{-1}) γ 3057, 3025, 2964, 2928, 1561, 1492, 1444, 1406, 1225, 1031, 830, 767, 742, 701. **HRMS** (ESI) m/z $(M+H)^+$: calculated for $(C_{26}H_{22}FN_2)^+$: 381.1762, found: 381.1767; $[\alpha]_D^{25}$ = +128.8 (c = 0.48, CHCl$_3$); The product was analyzed by HPLC to determine the enantiomeric excess: 93% e.e. (CHIRALPAK ID-3, *n*-hexane/*i*-PrOH = 98/2, flow rate: 1.0 mL/min, T = 20 °C, 250 nm), t_R (minor) = 10.94 min, t_R (major) = 15.23 min.

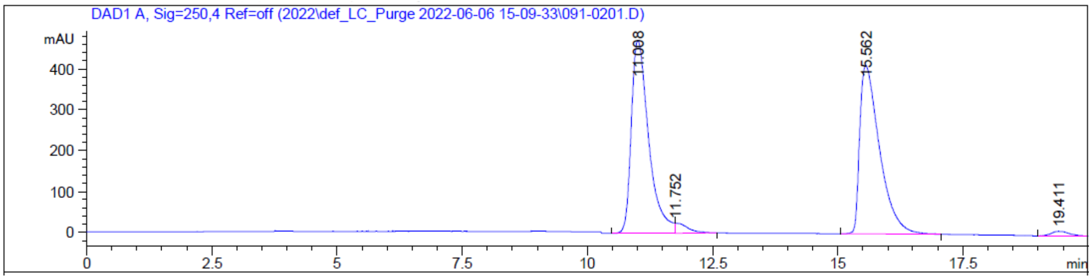

Peak #	RetTime [min]	Type	Width [min]	Area [mAU*s]	Height [mAU]	Area %
1	11.008	MF R	0.4024	1.13960e4	471.99448	48.5195
2	11.752	FM R	0.2893	430.01779	24.77355	1.8308
3	15.562	VB R	0.3995	1.13731e4	412.68903	48.4222
4	19.411	VV R	0.2922	288.31119	11.79509	1.2275

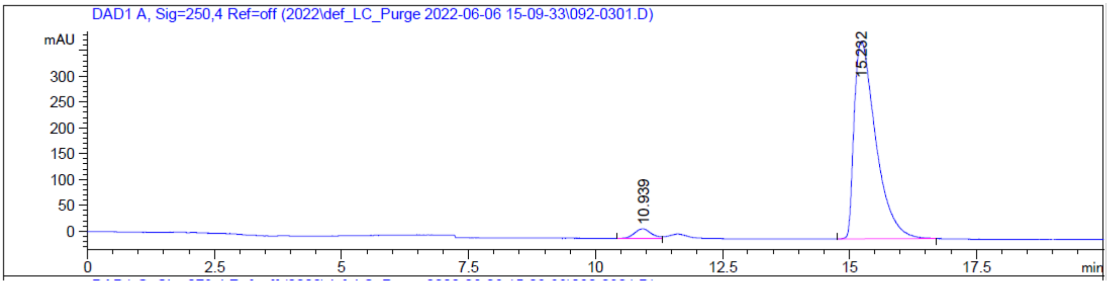

Peak #	RetTime [min]	Type	Width [min]	Area [mAU*s]	Height [mAU]	Area %
1	10.939	VV R	0.2732	427.57291	18.73896	3.6283
2	15.232	BV R	0.4302	1.13569e4	383.85370	96.3717

(*R*$_a$, *R*)-1-(4-Methyl-2-(1-phenylpropan-2-yl)-1*H*-indol-1-yl)isoquinoline (3c)

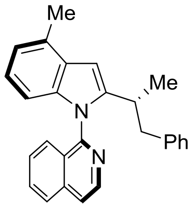

The general procedure was followed using 1-(4-methyl-1*H*-indol-1-yl)isoquinoline (0.1 mmol, 25.8 mg) and allylbenzene (0.3 mmol, 35.4 mg) to afford **3c** (33.1 mg, 88% yield, >95:5 d.r., b:l >95:5) as a white solid. M.p.: 125-126 °C. **^{1}H NMR (400 MHz, CDCl$_3$)** δ 8.63 (d, *J* = 5.7 Hz, 1H), 7.97 (d, *J* = 8.4 Hz, 1H), 7.83 (d, *J* = 5.7 Hz, 1H), 7.72 (ddd, *J* = 8.2, 6.0, 2.1 Hz, 1H), 7.45 – 7.36 (m, 2H), 7.10 – 7.01 (m, 3H), 6.98 – 6.90 (m, 2H), 6.74 – 6.67 (m, 2H), 6.66 (s, 1H), 6.55 (dd, *J* = 7.0, 1.9 Hz, 1H), 3.34 – 3.17 (m, 1H), 2.78 (dd, *J* = 13.4, 5.6 Hz, 1H), 2.65 (s, 3H), 2.51 (dd, *J* = 13.4, 8.6 Hz, 1H), 1.32 (d, *J* = 6.8 Hz, 3H). **^{13}C NMR (101 MHz, CDCl$_3$)** δ 150.72 (C_q), 146.70 (C_q), 141.97 (CH), 140.11 (C_q), 138.38 (C_q), 138.21 (C_q), 130.93 (CH), 129.60 (C_q), 128.91 (CH), 128.34 (CH), 128.05 (C_q), 128.02 (CH), 126.93 (CH), 125.99 (C_q), 125.92 (CH), 125.87 (CH), 121.66 (CH), 121.45 (CH), 120.64 (CH), 108.25 (CH), 98.82 (CH), 43.66 (CH_2), 33.54 (CH), 19.68 (CH_3), 18.86 (CH_3). **IR** (ATR, cm^{-1}) *γ* 3057, 3025, 2962, 2929, 1561, 1496, 1455, 1426, 1407, 829, 765, 737, 702. **HRMS** (ESI) m/z (M+H)$^+$: calculated for $(C_{27}H_{25}N_2)^+$: 377.2012, found: 377.2013; **$[\alpha]_D^{25}$** = +105.9 (c = 0.39, CHCl$_3$); The product was analyzed by HPLC to determine the enantiomeric excess: 95% e.e. (CHIRALPAK IC-3, *n*-hexane/*i*-PrOH = 98/2, flow rate: 1.0 mL/min, T = 20 °C, 250 nm), t_R (minor) = 16.85 min, t_R (major) = 27.47 min.

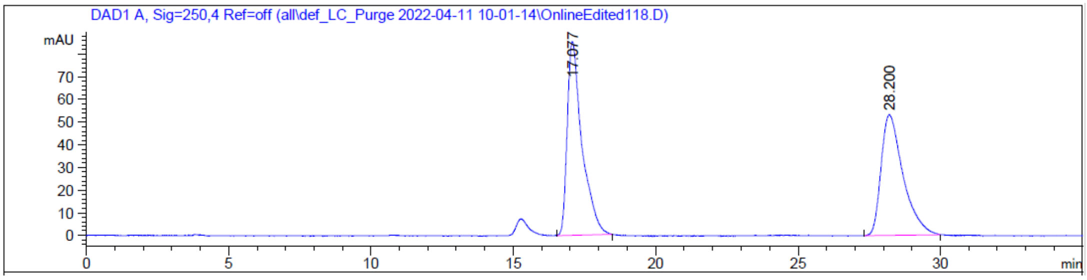

Peak #	RetTime [min]	Type	Width [min]	Area [mAU*s]	Height [mAU]	Area %
1	17.077	BB	0.4737	3069.78516	85.53126	51.7572
2	28.200	BV R	0.6302	2861.34448	53.31332	48.2428

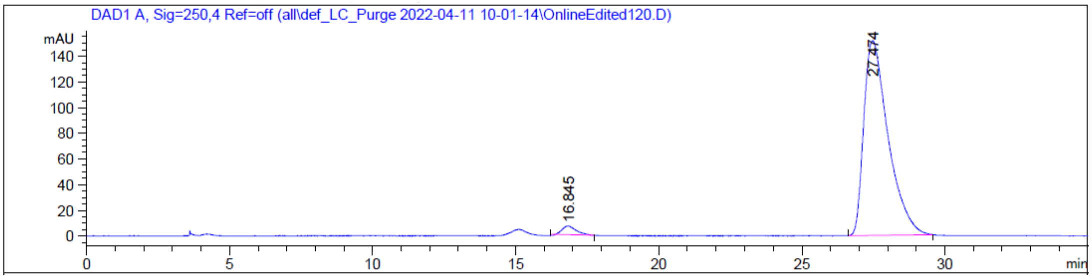

Peak #	RetTime [min]	Type	Width [min]	Area [mAU*s]	Height [mAU]	Area %
1	16.845	MM R	0.5825	232.71805	6.80422	2.5279
2	27.474	BV R	0.6938	8973.14746	151.54753	97.4721

(*R*$_a$, *R*)-1-(5-Fluoro-2-(1-phenylpropan-2-yl)-1*H*-indol-1-yl)isoquinoline (3d)

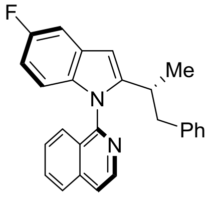

The general procedure was followed using 1-(5-fluoro-1*H*-indol-1-yl)isoquinoline (0.1 mmol, 26.2 mg) and allylbenzene (0.3 mmol, 35.4 mg) to afford **3d** (33.8 mg, 89% yield, >95:5 d.r., b:l >95:5) as a colorless oil. **^{1}H NMR (400 MHz, CDCl$_3$)** δ 8.62 (d, *J* = 5.7 Hz, 1H), 7.98 (d, *J* = 8.3 Hz, 1H), 7.84 (dd, *J* = 5.7, 0.9 Hz, 1H), 7.73 (ddd, *J* = 8.2, 6.8, 1.3 Hz, 1H), 7.43 (ddd, *J* = 8.2, 6.9, 1.2 Hz, 1H), 7.34 – 7.28 (m, 2H), 7.11 – 7.00 (m, 3H), 6.81 – 6.65 (m, 3H), 6.65 – 6.54 (m, 2H), 3.27 – 3.09 (m, 1H), 2.78 (dd, *J* = 13.5, 5.9 Hz, 1H), 2.53 (dd, *J* = 13.4, 8.3 Hz, 1H), 1.30 (d, *J* = 6.8 Hz, 3H). **^{13}C NMR (101 MHz, CDCl$_3$)** δ 158.51 (d, *J* = 234.8 Hz,

C_q), 150.38 (C_q), 149.09 (C_q), 142.09 (CH), 139.98 (C_q), 138.35 (C_q), 135.22 (C_q), 131.15 (CH), 128.99 (CH), 128.87 (d, J = 10.2 Hz, C_q), 128.63 (CH), 128.16 (CH), 127.13 (CH), 126.06 (CH), 126.01 (C_q), 125.65 (CH), 121.79 (CH), 111.18 (d, J = 9.7 Hz, CH), 109.67 (d, J = 26.1 Hz, CH), 105.27 (d, J = 23.6 Hz, CH), 100.45 (d, J = 4.3 Hz, CH), 43.53 (CH_2), 33.82 (CH), 19.97 (CH_3). **^{19}F NMR (282 MHz, $CDCl_3$)** δ -124.41. **IR** (ATR, cm^{-1}) γ 3058, 3025, 2964, 2927, 1472, 1451, 1406, 1182, 1114, 956, 748, 695. **HRMS** (ESI) m/z $(M+H)^+$: calculated for $(C_{26}H_{22}FN_2)^+$: 381.1762, found: 381.1758; $[\alpha]_D^{25}$ = +125.3 (c = 0.49, $CHCl_3$); The product was analyzed by HPLC to determine the enantiomeric excess: 92% e.e. (CHIRALPAK IC-3, *n*-hexane/*i*-PrOH = 95/5, flow rate: 1.0 mL/min, T = 20 °C, 250 nm), t_R (minor) = 7.15 min, t_R (major) = 9.69 min.

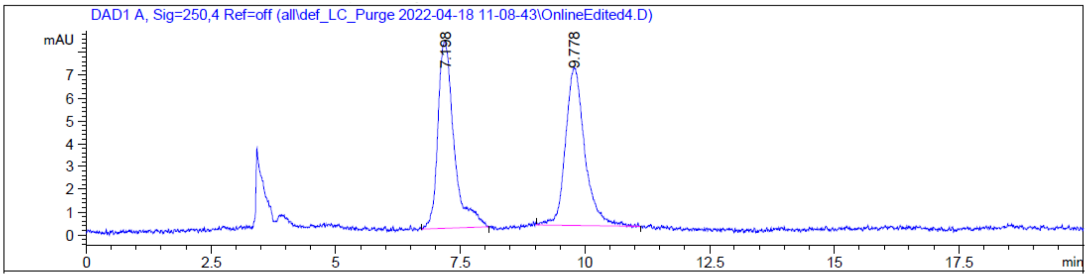

Peak #	RetTime [min]	Type	Width [min]	Area [mAU*s]	Height [mAU]	Area %
1	7.198	MM R	0.3743	184.37756	8.20913	49.6794
2	9.778	MM R	0.4480	186.75708	6.94833	50.3206

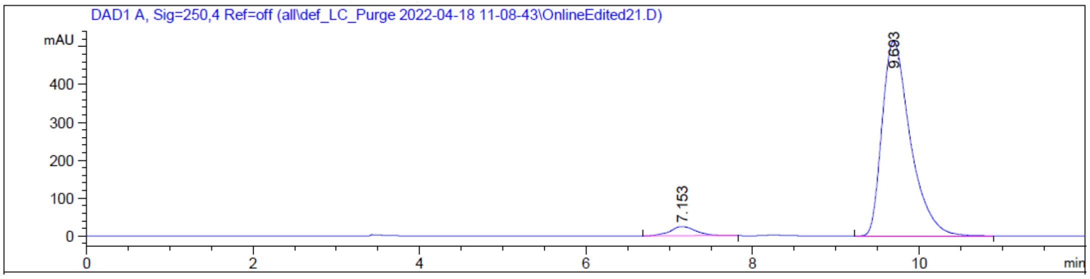

Peak #	RetTime [min]	Type	Width [min]	Area [mAU*s]	Height [mAU]	Area %
1	7.153	VV R	0.2584	527.52722	24.38125	4.1744
2	9.693	BV R	0.3595	1.21096e4	514.74207	95.8256

(R_a, *R*)-1-(5-Bromo-2-(1-phenylpropan-2-yl)-1*H*-indol-1-yl)isoquinoline (3e)

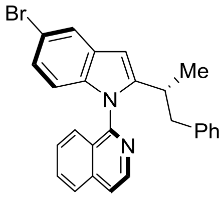

The general procedure was followed using 1-(5-bromo-1*H*-indol-1-yl)isoquinoline (0.1 mmol, 32.2 mg) and allylbenzene (0.3 mmol, 35.4 mg) to afford **3e** (32.1 mg, 73% yield, >95:5 d.r., b:l >95:5) as a colorless oil. **^{1}H NMR (400 MHz, $CDCl_3$)** δ 8.61 (d, *J* = 5.5 Hz, 1H), 7.97 (d, *J* = 8.3 Hz, 1H), 7.85 (d, *J* = 5.5 Hz, 1H), 7.78 (d, *J* = 2.0 Hz, 1H), 7.73 (t, *J* = 7.5 Hz, 1H), 7.41 (t, *J* = 7.6 Hz, 1H), 7.23 (d, *J* = 8.5 Hz, 1H), 7.08 (dd, *J* = 8.6, 2.0 Hz, 1H), 7.07 – 7.00 (m, 3H), 6.71 – 6.63 (m, 2H), 6.57 (s, 1H), 6.55 (d, *J* = 8.6 Hz, 1H), 3.27 – 3.09 (m, 1H), 2.75 (dd, *J* = 13.4, 6.0 Hz, 1H), 2.52 (dd, *J* = 13.4, 8.2 Hz, 1H), 1.29 (d, *J* = 6.8 Hz, 3H). **^{13}C NMR (101 MHz, $CDCl_3$)** δ 150.07 (C_q), 148.67 (C_q), 142.06 (CH), 139.88 (C_q), 138.34 (C_q), 137.29 (C_q), 131.21 (CH), 130.15 (C_q), 128.98 (CH), 128.71 (CH), 128.16 (CH), 127.15 (CH), 126.09 (CH), 125.92 (C_q), 125.54 (CH), 124.40 (CH), 122.78 (CH), 121.93 (CH), 113.72 (C_q), 112.07 (CH), 99.93 (CH), 43.48 (CH_2), 33.73 (CH), 20.00 (CH_3). **IR** (ATR, cm^{-1}) *γ* 3059, 3026, 2970, 2927, 1560, 1496, 1449, 1407, 797, 748, 701. **HRMS** (ESI) m/z $(M+H)^+$: calculated for $(C_{26}H_{22}BrN_2)^+$: 441.0961, found: 441.0959; $[\alpha]_D^{25}$ = +119.6 (c = 0.28, $CHCl_3$); The product was analyzed by HPLC to determine the enantiomeric excess: 93% e.e. (CHIRALPAK IC-3, *n*-hexane/*i*-PrOH = 98/2, flow rate: 1.0 mL/min, T = 20 °C, 250 nm), t_R (minor) = 10.78 min, t_R (major) = 19.71 min.

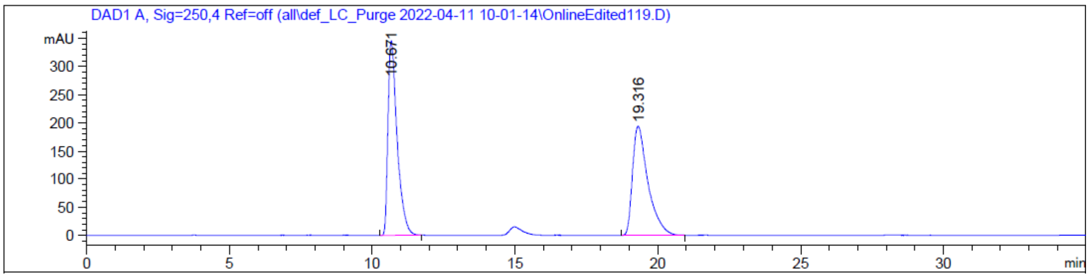

Peak #	RetTime [min]	Type	Width [min]	Area [mAU*s]	Height [mAU]	Area %
1	10.671	BB	0.3275	7710.67627	345.27066	51.4040
2	19.316	BV R	0.5161	7289.47510	193.65602	48.5960

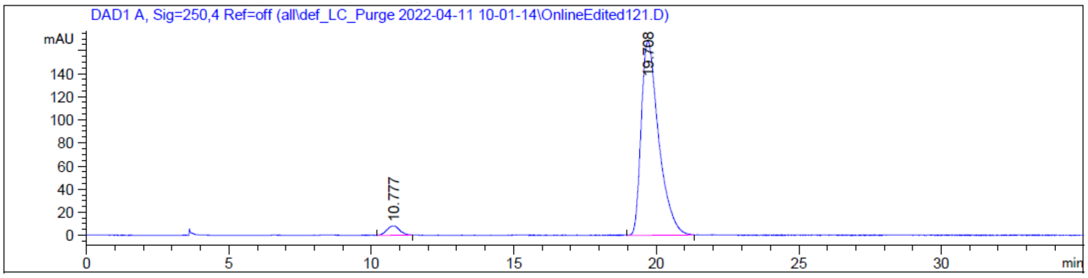

Peak #	RetTime [min]	Type	Width [min]	Area [mAU*s]	Height [mAU]	Area %
1	10.777	BB	0.3627	253.15404	8.23455	3.4209
2	19.708	BB	0.5511	7147.10596	168.06892	96.5791

(*R_a*, *R*)-1-(5-Methyl-2-(1-phenylpropan-2-yl)-1*H*-indol-1-yl)isoquinoline (3f)

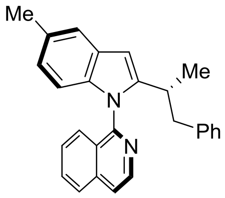

The general procedure was followed using 1-(5-methyl-1*H*-indol-1-yl)isoquinoline (0.1 mmol, 25.8 mg) and allylbenzene (0.3 mmol, 35.4 mg) to afford **3f** (26.0 mg, 69% yield, 94:6 d.r., b:l >95:5) as a colorless oil. **^{1}H NMR (400 MHz, $CDCl_3$)** δ 8.61 (d, J = 5.7 Hz, 1H), 7.96 (d, J = 8.2 Hz, 1H), 7.82 (d, J = 5.7 Hz, 1H), 7.72 (ddd, J = 8.2, 6.0, 2.1 Hz, 1H), 7.47 – 7.33 (m, 3H), 7.09 – 6.99 (m, 3H), 6.83 (dd, J = 8.3, 1.6 Hz, 1H), 6.73 – 6.62 (m, 2H), 6.59 (d, J = 8.4 Hz, 1H), 6.56 (s, 1H), 3.34 – 3.12 (m, 1H), 2.75 (dd, J = 13.4, 5.8 Hz, 1H), 2.49 (dd, J = 13.5, 8.4 Hz, 1H), 2.45 (s, 3H), 1.30 (d, J = 6.8 Hz, 3H). **^{13}C NMR (101 MHz, $CDCl_3$)** δ 150.82 (C_q), 147.42 (C_q), 142.04 (CH), 140.18 (C_q), 138.32 (C_q), 137.18 (C_q), 131.00 (CH), 129.68 (C_q), 129.02 (CH), 128.70 (C_q), 128.40 (CH), 128.09 (CH), 127.02 (CH), 126.05 (CH), 125.95 (CH), 123.08 (CH), 121.46 (CH), 120.06 (CH), 110.33 (CH), 100.12 (CH), 43.66 (CH_2), 33.60 (CH), 21.56 (CH_3), 19.79 (CH_3). **IR** (ATR, cm^{-1}) γ 3057, 3023, 2963, 2927, 1716, 1560, 1476, 1454, 1406, 1179, 828, 792, 749, 706. **HRMS** (ESI) m/z $(M+H)^+$: calculated for $(C_{27}H_{25}N_2)^+$: 377.2012, found: 377.2013; $[\alpha]_D^{25}$ = +133.3 (c = 0.33, $CHCl_3$); The product was analyzed by HPLC to determine the enantiomeric excess: 94% e.e. (CHIRALPAK IC-3, *n*-hexane/*i*-PrOH = 95/5, flow rate: 1.0 mL/min, T = 20 °C, 250 nm), t_R (minor) = 9.90 min, t_R (major) = 25.69 min.

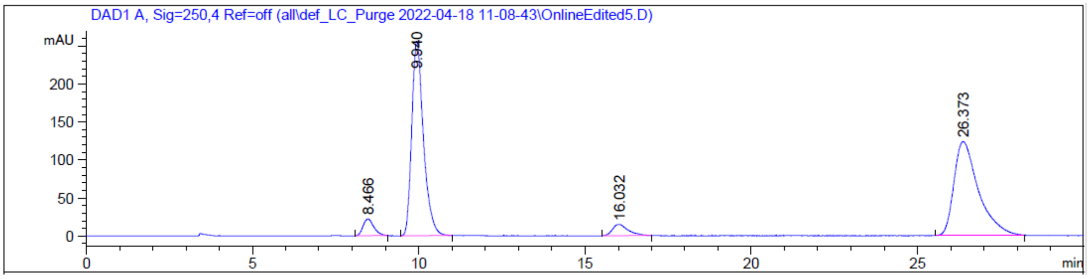

Peak #	RetTime [min]	Type	Width [min]	Area [mAU*s]	Height [mAU]	Area %
1	8.466	BB	0.2670	491.64209	21.97227	3.6216
2	9.940	BV R	0.3625	6270.52002	256.25281	46.1910
3	16.032	BV R	0.3821	485.05640	15.00779	3.5731
4	26.373	BV R	0.6006	6327.98779	123.53133	46.6143

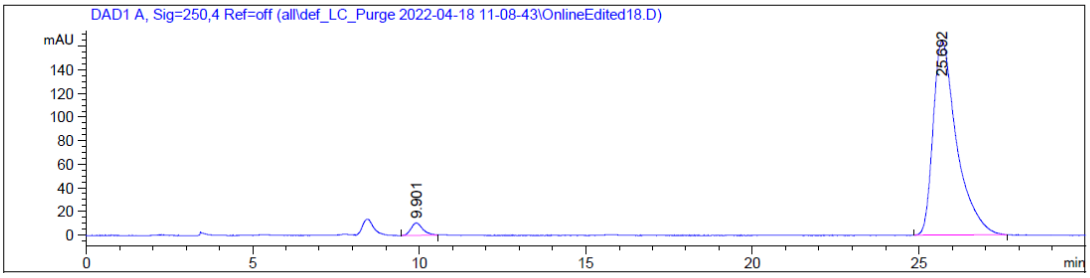

Peak #	RetTime [min]	Type	Width [min]	Area [mAU*s]	Height [mAU]	Area %
1	9.901	BB	0.2894	251.73320	10.40021	3.0206
2	25.692	BB	0.5990	8082.05371	163.98132	96.9794

(*R*$_a$, *R*)-1-(5-Methoxy-2-(1-phenylpropan-2-yl)-1*H*-indol-1-yl)isoquinoline (3g)

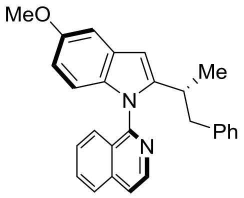

The general procedure was followed using 1-(5-methoxy-1*H*-indol-1-yl)isoquinoline (0.1 mmol, 27.4 mg) and allylbenzene (0.3 mmol, 35.4 mg) to afford **3g** (28.3 mg, 72% yield, 95:5 d.r., b:l >95:5) as a colorless oil. **^{1}H NMR (400 MHz, CDCl$_3$)** δ 8.61 (d, *J* = 5.7 Hz, 1H), 7.96 (dt, *J* = 8.3, 0.9 Hz, 1H), 7.81 (dd, *J* = 5.7, 0.8 Hz, 1H), 7.72 (ddd, *J* = 8.2, 6.4, 1.6 Hz, 1H), 7.52 – 7.32 (m, 2H), 7.14 (d, *J* = 2.4 Hz, 1H), 7.09 – 6.98 (m, 3H), 6.74 – 6.61 (m, 3H), 6.59 (d, *J* = 10.4 Hz, 2H), 3.87 (s, 3H), 3.32 – 3.13 (m, 1H), 2.76 (dd, *J* = 13.4, 5.8 Hz, 1H), 2.51 (dd,

J = 13.4, 8.4 Hz, 1H), 1.30 (d, J = 6.8 Hz, 3H). **^{13}C NMR (101 MHz, CDCl$_3$)** δ 154.76 (C_q), 150.73 (C_q), 148.04 (C_q), 142.04 (CH), 140.14 (C_q), 138.33 (C_q), 134.03 (C_q), 131.03 (CH), 129.01 (CH), 128.93 (C_q), 128.44 (CH), 128.11 (CH), 127.04 (CH), 126.04 (C_q), 125.98 (CH), 125.96 (CH), 121.50 (CH), 111.34 (CH), 111.26 (CH), 102.45 (CH), 100.35 (CH), 55.98 (CH_3), 43.66 (CH_2), 33.68 (CH), 19.85 (CH_3). **IR** (ATR, cm^{-1}) γ 3059, 3024, 2963, 2927, 2829, 1475, 1451, 1407, 1222, 1208, 1175, 1034, 829, 749. **HRMS** (ESI) m/z $(M+H)^+$: calculated for $(C_{27}H_{25}N_2O)^+$: 393.1961, found: 393.1954; **$[\alpha]_D^{25}$** = +85.9 (c = 0.39, $CHCl_3$); The product was analyzed by HPLC to determine the enantiomeric excess: 95% e.e. (CHIRALPAK IA-3, *n*-hexane/*i*-PrOH = 95/5, flow rate: 1.0 mL/min, T = 20 °C, 250 nm), t_R (major) = 11.34 min, t_R (minor) = 12.79 min.

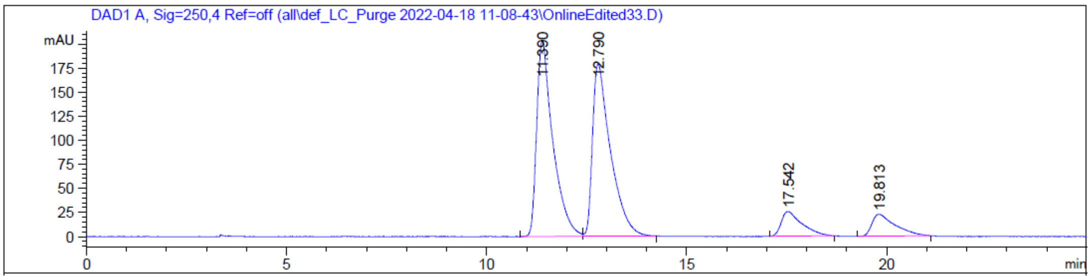

Peak #	RetTime [min]	Type	Width [min]	Area [mAU*s]	Height [mAU]	Area %
1	11.390	VV R	0.4033	5725.45801	202.85526	43.3922
2	12.790	VV R	0.4263	5614.26514	179.29132	42.5495
3	17.542	BV R	0.4250	928.84698	25.86042	7.0396
4	19.813	BB	0.4751	926.10919	22.93586	7.0188

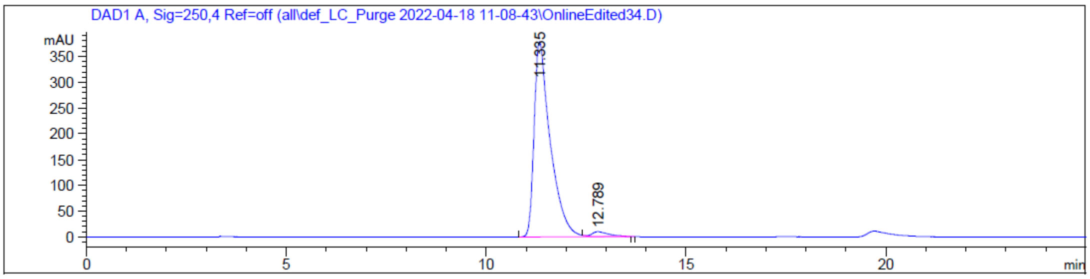

Peak #	RetTime [min]	Type	Width [min]	Area [mAU*s]	Height [mAU]	Area %
1	11.335	VV R	0.3988	1.06724e4	378.69360	97.3626
2	12.789	VV E	0.3500	289.09479	9.71874	2.6374

(R_a, R)-1-(2-(1-Phenylpropan-2-yl)-5-(trifluoromethyl)-1*H*-indol-1-yl)isoquinoline (3h)

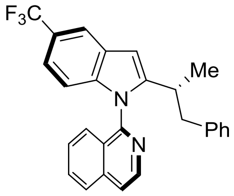

The general procedure was followed using 1-(5-(trifluoromethyl)-1*H*-indol-1-yl)isoquinoline (0.1 mmol, 31.2 mg) and allylbenzene (0.3 mmol, 35.4 mg) to afford **3h** (39.5 mg, 92% yield, >95:5 d.r., b:l >95:5) as a colorless oil. **^{1}H NMR (400 MHz, CDCl$_3$)** δ 8.64 (d, *J* = 5.7 Hz, 1H), 8.03 – 7.93 (m, 2H), 7.88 (d, *J* = 5.7 Hz, 1H), 7.75 (ddd, *J* = 8.2, 6.8, 1.2 Hz, 1H), 7.43 (ddd, *J* = 8.2, 6.9, 1.2 Hz, 1H), 7.28 – 7.18 (m, 2H), 7.09 – 7.01 (m, 3H), 6.78 – 6.63 (m, 4H), 3.31 – 3.04 (m, 1H), 2.78 (dd, *J* = 13.5, 6.1 Hz, 1H), 2.55 (dd, *J* = 13.5, 8.2 Hz, 1H), 1.32 (d, *J* = 6.8 Hz, 3H). **^{13}C NMR (101 MHz, CDCl$_3$)** δ 149.88 (C_q), 149.29 (C_q), 142.11 (CH), 139.82 (C_q), 138.37 (C_q), 131.28 (CH), 128.98 (CH), 128.84 (CH), 128.20 (CH), 127.84 (C_q), 127.22 (CH), 126.14 (CH), 125.93 (C_q), 125.48 (q, *J* = 271.4 Hz, C_q), 125.38 (CH), 122.89 (q, *J* = 31.8 Hz, C_q), 122.12 (CH), 118.46 (q, *J* = 3.5 Hz, CH), 117.95 (q, *J* = 4.1 Hz, CH), 110.79 (CH), 100.95 (CH), 43.44 (CH_2), 33.80 (CH), 20.11 (CH_3). **^{19}F NMR (282 MHz, CDCl$_3$)** δ -60.30. **IR** (ATR, cm^{-1}) *γ* 3059, 3027, 2963, 2931, 1452, 1406, 1331, 1153, 1111, 1055, 750, 698. **HRMS** (ESI) m/z (M+H)$^+$: calculated for $(C_{27}H_{22}F_3N_2)^+$: 431.1730, found: 431.1726; **$[\alpha]_D^{25}$** = +105.8 (c = 0.50, $CHCl_3$); The product was analyzed by HPLC to determine the enantiomeric excess: 93% e.e. (CHIRALPAK IC-3, *n*-hexane/*i*-PrOH = 95/5, flow rate: 1.0 mL/min, T = 20 °C, 250 nm), t_R (minor) = 5.18 min, t_R (major) = 6.99 min.

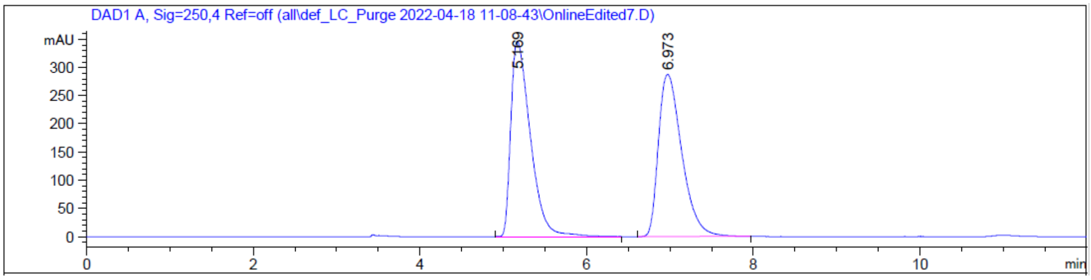

Peak #	RetTime [min]	Type	Width [min]	Area [mAU*s]	Height [mAU]	Area %
1	5.169	BV R	0.2465	5620.15186	346.08945	50.0897
2	6.973	BV R	0.2963	5600.03027	287.44205	49.9103

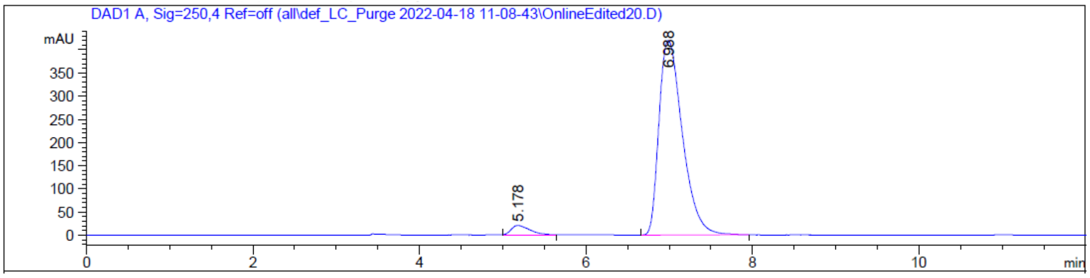

Peak #	RetTime [min]	Type	Width [min]	Area [mAU*s]	Height [mAU]	Area %
1	5.178	BB	0.2097	301.17508	19.66245	3.5371
2	6.988	BB	0.2982	8213.62012	419.99622	96.4629

Methyl (*R*$_a$, *R*)-1-(isoquinolin-1-yl)-2-(1-phenylpropan-2-yl)-1*H*-indole-5-carboxylate (3i)

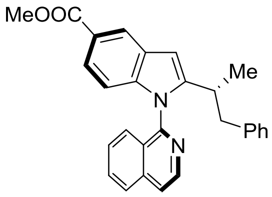

The general procedure was followed using methyl 1-(isoquinolin-1-yl)-1*H*-indole-5-carboxylate (0.1 mmol, 30.2 mg) and allylbenzene (0.3 mmol, 35.4 mg) to afford **3i** (31.9 mg, 76% yield, >95:5 d.r., b:l >95:5) as a colorless oil. **^{1}H NMR (400 MHz, CDCl$_3$)** δ 8.63 (d, *J* = 5.7 Hz, 1H), 8.42 (d, *J* = 1.5 Hz, 1H), 7.98 (d, *J* = 8.2 Hz, 1H), 7.86 (d, *J* = 5.7 Hz, 1H), 7.80 – 7.67 (m, 2H), 7.42 (ddd, *J* = 8.2, 6.8, 1.2 Hz, 1H), 7.23 (d, *J* = 8.5 Hz, 1H), 7.09 – 6.99 (m, 3H), 6.74 – 6.62 (m, 4H), 3.92 (s, 3H), 3.27 – 3.09 (m, 1H), 2.76 (dd, *J* = 13.4, 6.0 Hz, 1H), 2.52 (dd, *J* = 13.4, 8.3 Hz, 1H), 1.30 (d, *J* = 6.8 Hz, 3H). **^{13}C NMR (101 MHz, CDCl$_3$)** δ 168.27 (C$_q$), 149.98 (C$_q$), 148.86 (C$_q$), 142.06 (CH), 141.06 (C$_q$), 139.86 (C$_q$), 138.36 (C$_q$), 131.26 (CH), 128.98 (CH), 128.79 (CH), 128.17 (CH), 128.01 (C$_q$), 127.20 (CH), 126.10 (CH), 125.91 (C$_q$), 125.44 (CH), 123.20 (CH), 123.15 (CH), 122.57 (C$_q$), 122.06 (CH), 110.28 (CH), 101.43 (CH), 51.97 (CH$_3$), 43.45 (CH$_2$), 33.75 (CH), 19.95 (CH$_3$). **IR** (ATR, cm^{-1}) *γ* 3059, 3027, 2970, 2947, 2927, 1714, 1451, 1405, 1309, 1251, 1154, 1090, 768, 749. **HRMS** (ESI) m/z (M+H)$^+$: calculated for $(C_{28}H_{25}N_2O_2)^+$: 421.1911, found: 421.1906; **$[\alpha]_D^{25}$** = +153.6 (c = 0.36, CHCl$_3$); The product was analyzed by HPLC to determine the enantiomeric excess: 94% e.e. (CHIRALPAK ID-3, *n*-hexane/*i*-PrOH = 90/10, flow rate: 1.0 mL/min, T = 20 °C, 250 nm), t$_R$ (major) = 19.06 min, t$_R$ (minor) = 35.36 min.

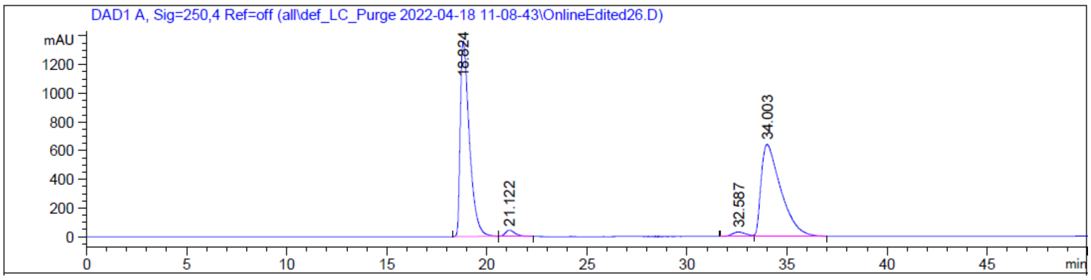

Peak #	RetTime [min]	Type	Width [min]	Area [mAU*s]	Height [mAU]	Area %
1	18.824	BV	0.3780	4.32564e4	1360.44165	48.1352
2	21.122	VV R	0.4040	1521.26367	44.25684	1.6928
3	32.587	VV E	0.5606	1413.65369	30.01004	1.5731
4	34.003	VV R	0.8013	4.36730e4	640.69299	48.5988

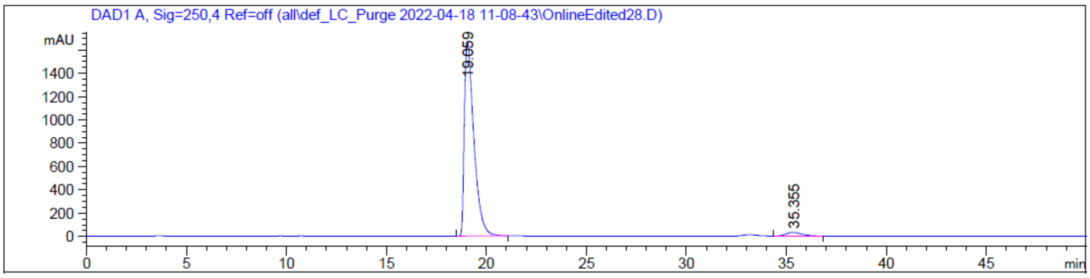

Peak #	RetTime [min]	Type	Width [min]	Area [mAU*s]	Height [mAU]	Area %
1	19.059	BV R	0.3898	5.52061e4	1673.57349	96.9305
2	35.355	VV R	0.6464	1748.18799	31.74996	3.0695

(R_a, R)-1-(6-Methyl-2-(1-phenylpropan-2-yl)-1*H*-indol-1-yl)isoquinoline (3j)

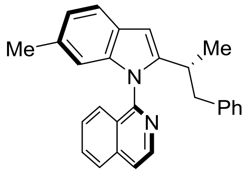

The general procedure was followed using 1-(6-methyl-1*H*-indol-1-yl)isoquinoline (0.1 mmol, 25.8 mg) and allylbenzene (0.3 mmol, 35.4 mg) to afford **3j** (26.0 mg, 69% yield, 94:6 d.r., b:l >95:5) as a colorless oil. **^{1}H NMR (400 MHz, $CDCl_3$)** δ 8.62 (d, J = 5.7 Hz, 1H), 7.97 (d, J = 8.2 Hz, 1H), 7.83 (d, J = 5.7 Hz, 1H), 7.73 (ddd, J = 8.2, 6.5, 1.5 Hz, 1H), 7.54 (d, J = 8.0 Hz, 1H), 7.46 – 7.33 (m, 2H), 7.10 – 7.01 (m, 3H), 6.96 (dd, J = 8.0, 1.4 Hz, 1H), 6.69 (dd, J = 6.6, 2.9 Hz, 2H), 6.58 (s, 1H), 6.50 (d, J = 0.8 Hz, 1H), 3.29 – 3.06 (m, 1H), 2.77 (dd, J = 13.4,

5.7 Hz, 1H), 2.50 (dd, J = 13.5, 8.4 Hz, 1H), 2.27 (s, 3H), 1.26 (d, J = 6.8 Hz, 3H). **^{13}C NMR (101 MHz, CDCl$_3$)** δ 150.86 (C_q), 146.70 (C_q), 142.12 (CH), 140.24 (C_q), 139.19 (C_q), 138.31 (C_q), 131.48 (C_q), 131.05 (CH), 129.05 (CH), 128.47 (CH), 128.10 (CH), 127.03 (CH), 126.23 (C_q), 126.02 (CH), 125.95 (CH), 122.12 (CH), 121.53 (CH), 119.88 (CH), 110.59 (CH), 100.23 (CH), 43.53 (CH_2), 33.66 (CH), 21.82 (CH_3), 19.94 (CH_3). **IR** (ATR, cm^{-1}) γ 3058, 3025, 2962, 2928, 2853, 1560, 1497, 1454, 1406, 829, 811, 748, 698. **HRMS** (ESI) m/z (M+H)$^+$: calculated for $(C_{27}H_{25}N_2)^+$: 377.2012, found: 377.2013; **$[\alpha]_D^{25}$** = +138.2 (c = 0.28, CHCl$_3$); The product was analyzed by HPLC to determine the enantiomeric excess: 93% e.e. (CHIRALPAK IC-3, *n*-hexane/*i*-PrOH = 95/5, flow rate: 1.0 mL/min, T = 20 °C, 250 nm), t_R (minor) = 7.54 min, t_R (major) = 11.47 min.

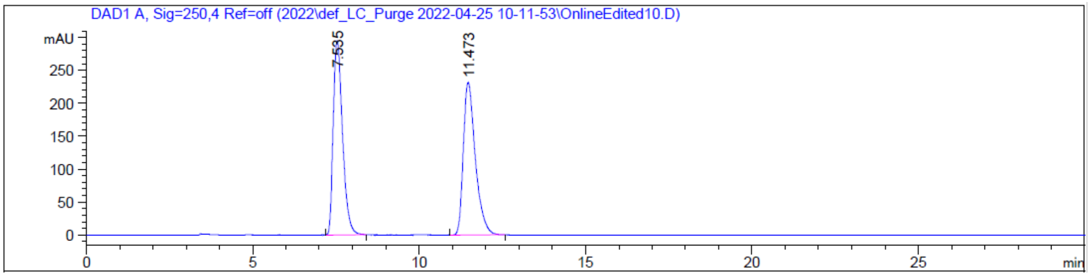

Peak #	RetTime [min]	Type	Width [min]	Area [mAU*s]	Height [mAU]	Area %
1	7.535	BB	0.3043	5751.38379	295.22836	49.6170
2	11.473	BV R	0.3706	5840.16846	232.06866	50.3830

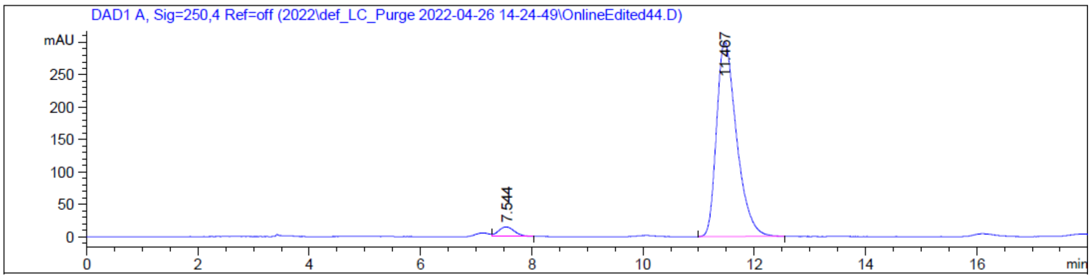

Peak #	RetTime [min]	Type	Width [min]	Area [mAU*s]	Height [mAU]	Area %
1	7.544	VV R	0.2389	290.48849	14.61182	3.7242
2	11.467	VV R	0.3697	7509.62549	301.39938	96.2758

(R_a, *R*)-1-(3-Methylpyridin-2-yl)-2-(1-phenylpropan-2-yl)-1*H*-indole (3k)

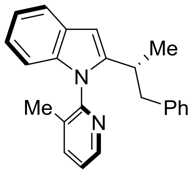

The general procedure was followed using 1-(3-methylpyridin-2-yl)-1*H*-indole (0.1 mmol, 20.8 mg) and allylbenzene (0.3 mmol, 35.4 mg) to afford **3k** (18.0 mg, 55% yield, 82:18 d.r., b:l >95:5) as a colorless oil. **^{1}H NMR (400 MHz, $CDCl_3$)** δ 8.56 (dd, *J* = 4.7, 1.8 Hz, 1H), 7.75 (ddd, *J* = 7.6, 1.9, 0.8 Hz, 1H), 7.65 – 7.60 (m, 1H), 7.38 (dd, *J* = 7.6, 4.7 Hz, 1H), 7.20 – 7.03 (m, 5H), 6.88 – 6.81 (m, 2H), 6.77 (d, *J* = 8.0 Hz, 1H), 6.53 (s, 1H), 3.16 – 3.02 (m, 1H), 2.83 (dd, *J* = 13.4, 5.7 Hz, 1H), 2.57 (dd, *J* = 13.3, 8.6 Hz, 1H), 1.89 (s, 3H), 1.29 (d, *J* = 6.8 Hz, 3H). **^{13}C NMR (101 MHz, $CDCl_3$)** δ 150.42 (C_q), 147.58 (CH), 146.30 (C_q), 140.39 (C_q), 140.25 (CH), 136.95 (C_q), 132.28 (C_q), 129.15 (CH), 128.27 (C_q), 128.25 (CH), 126.10 (CH), 123.83 (CH), 121.54 (CH), 120.37 (CH), 120.21 (CH), 109.96 (CH), 99.60 (CH), 43.53 (CH_2), 33.72 (CH), 19.88 (CH_3), 17.26 (CH_3). **IR** (ATR, cm^{-1}) γ 3049, 3023, 2961, 2925, 1458, 792, 749, 698, 417. **HRMS** (ESI) m/z $(M+H)^+$: calculated for $(C_{23}H_{23}N_2)^+$: 327.1856, found: 327.1854; $[\alpha]_D^{25}$ = +19.1 (c = 0.11, $CHCl_3$); The product was analyzed by HPLC to determine the enantiomeric excess: 82% e.e. (CHIRALPAK IC-3, *n*-hexane/*i*-PrOH = 95/5, flow rate: 1.0 mL/min, T = 20 °C, 250 nm), t_R (minor) = 8.54 min, t_R (major) = 14.26 min.

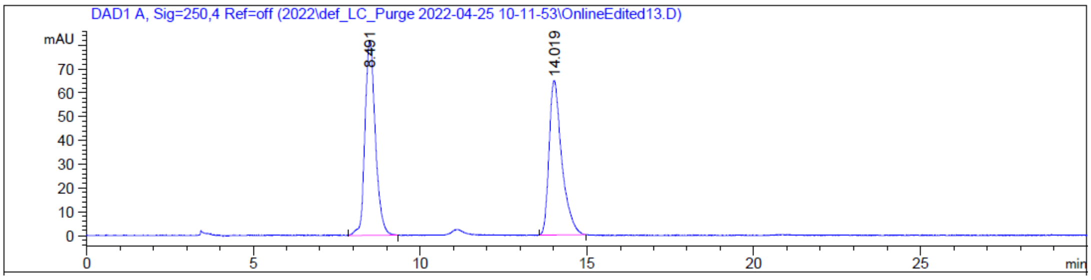

Peak #	RetTime [min]	Type	Width [min]	Area [mAU*s]	Height [mAU]	Area %
1	8.491	BV R	0.3122	1779.29956	81.99160	50.8297
2	14.019	BB	0.3467	1721.20911	65.03909	49.1703

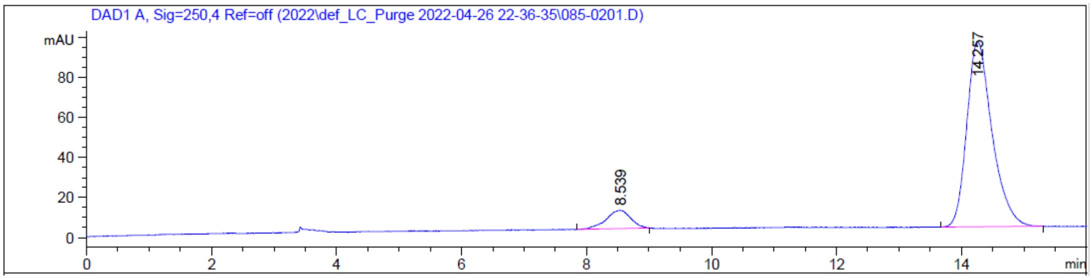

Peak #	RetTime [min]	Type	Width [min]	Area [mAU*s]	Height [mAU]	Area %
1	8.539	VB R	0.3247	251.45515	9.12061	8.8033
2	14.257	BV R	0.3751	2604.91846	92.62585	91.1967

(*Ra*, *R*)-1-(2-(1-(4-Fluorophenyl)propan-2-yl)-1*H*-indol-1-yl)isoquinoline (3l)**

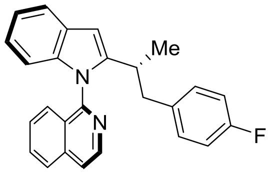

The general procedure was followed using 1-(1*H*-indol-1-yl)isoquinoline (0.1 mmol, 24.4 mg) and 1-allyl-4-fluorobenzene (0.3 mmol, 40.8 mg) to afford **3l** (36.1 mg, 95% yield, >95:5 d.r., b:l >95:5) as a colorless oil. **^{1}H NMR (400 MHz, $CDCl_3$)** δ 8.61 (d, *J* = 5.1 Hz, 1H), 7.96 (d, *J* = 8.3 Hz, 1H), 7.82 (d, *J* = 5.7 Hz, 1H), 7.73 (t, *J* = 7.6 Hz, 1H), 7.67 (d, *J* = 7.8 Hz, 1H), 7.40 (t, *J* = 7.7 Hz, 1H), 7.27 (d, *J* = 6.8 Hz, 1H), 7.14 (t, *J* = 7.4 Hz, 1H), 7.00 (t, *J* = 7.6 Hz, 1H), 6.74 – 6.57 (m, 6H), 3.37 – 3.07 (m, 1H), 2.73 (dd, *J* = 13.6, 6.6 Hz, 1H), 2.53 (dd, *J* = 13.6, 7.6 Hz, 1H), 1.33 (d, *J* = 6.8 Hz, 3H). **^{13}C NMR (101 MHz, $CDCl_3$)** δ 161.32 (d, *J* = 243.5 Hz, C_q), 150.59 (C_q), 147.01 (C_q), 142.04 (CH), 138.63 (C_q), 138.25 (C_q), 135.76 (d, *J* = 3.3 Hz, C_q), 131.06 (CH), 130.27 (d, *J* = 7.9 Hz, CH), 128.41 (CH), 127.02 (CH), 126.00 (C_q), 125.85 (CH), 121.67 (CH), 121.58 (CH), 120.52 (CH), 120.27 (CH), 114.83 (d, *J* = 21.1 Hz, CH), 110.71 (CH), 100.54 (CH), 42.97 (CH_2), 33.81 (CH), 20.27 (CH_3). **^{19}F NMR (282 MHz, $CDCl_3$)** δ -117.51. **IR** (ATR, cm^{-1}) *γ* 3057, 3027, 2963, 2928, 1561, 1509, 1456, 1406, 1219, 830, 791, 748. **HRMS** (ESI) m/z $(M+H)^+$: calculated for $(C_{26}H_{22}FN_2)^+$: 381.1762, found: 381.1759; **$[\alpha]_D^{25}$** = +88.4 (c = 0.50, $CHCl_3$); The product was analyzed by HPLC to determine the enantiomeric excess: 93% e.e. (CHIRALPAK ID-3, *n*-hexane/*i*-PrOH = 95/5, flow rate: 1.0 mL/min, T = 20 °C, 250 nm), t_R (minor) = 10.87 min, t_R (major) = 15.12 min.

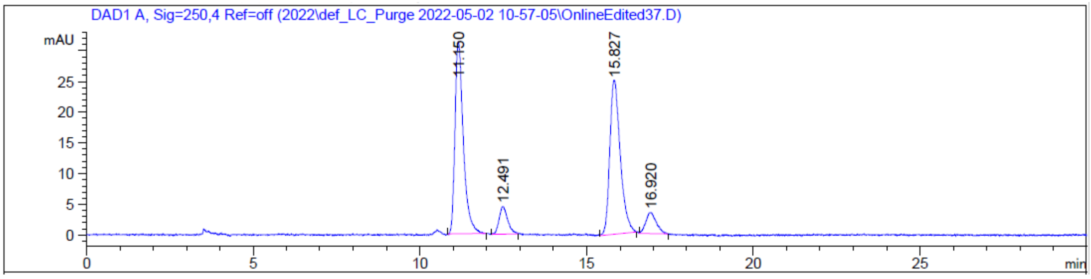

Peak #	RetTime [min]	Type	Width [min]	Area [mAU*s]	Height [mAU]	Area %
1	11.150	MM R	0.2813	541.21216	31.27962	43.5431
2	12.491	VV R	0.2147	80.78650	4.49393	6.4997
3	15.827	BV R	0.2721	546.80127	25.10778	43.9927
4	16.920	MM R	0.3589	74.13520	3.44272	5.9645

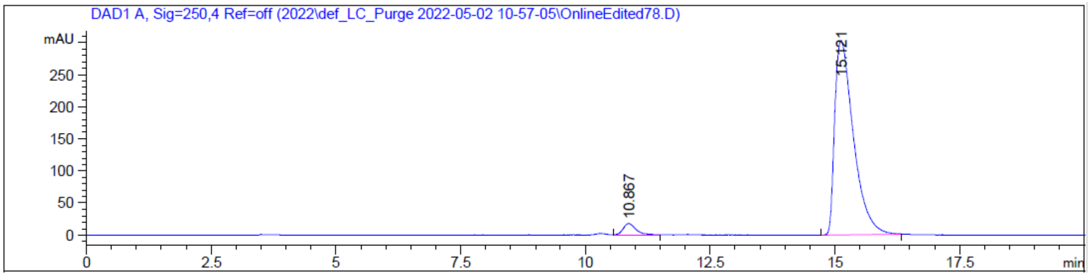

Peak #	RetTime [min]	Type	Width [min]	Area [mAU*s]	Height [mAU]	Area %
1	10.867	MM R	0.2955	297.24283	17.46528	3.6329
2	15.121	VB R	0.3738	7884.83936	302.76056	96.3671

(R_a, *R*)-1-(2-(1-(4-Chlorophenyl)propan-2-yl)-1*H*-indol-1-yl)isoquinoline (3m)

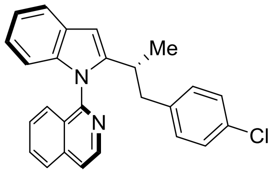

The general procedure was followed using 1-(1*H*-indol-1-yl)isoquinoline (0.1 mmol, 24.4 mg) and 1-allyl-4-chlorobenzene (0.3 mmol, 45.6 mg) to afford **3m** (21.6 mg, 55% yield, >95:5 d.r., b:l >95:5) as a colorless oil. **^{1}H NMR (400 MHz, $CDCl_3$)** δ 8.59 (d, *J* = 5.6 Hz, 1H), 7.95 (d, *J* = 8.3 Hz, 1H), 7.81 (d, *J* = 5.6 Hz, 1H), 7.73 (t, *J* = 7.5 Hz, 1H), 7.65 (d, *J* = 7.8 Hz, 1H), 7.39 (t, *J* = 7.6 Hz, 1H), 7.19 (d, *J* = 8.5 Hz, 1H), 7.12 (t, *J* = 7.4 Hz, 1H), 6.99 (t, *J* = 7.6 Hz, 1H), 6.93 (d, *J* = 7.8 Hz, 2H), 6.65 (d, *J* = 8.3 Hz, 1H), 6.63 (s, 1H), 6.59 (d, *J* = 7.8 Hz, 2H),

3.28 – 3.07 (m, 1H), 2.72 (dd, *J* = 13.5, 7.0 Hz, 1H), 2.54 (dd, *J* = 13.5, 7.3 Hz, 1H), 1.34 (d, *J* = 6.8 Hz, 3H). **^{13}C NMR (101 MHz, CDCl$_3$)** δ 150.55 (C$_q$), 146.85 (C$_q$), 142.02 (CH), 138.58 (C$_q$), 138.21 (C$_q$), 131.73 (C$_q$), 131.08 (CH), 130.26 (CH), 128.45 (CH), 128.40 (C$_q$), 128.19 (CH), 126.99 (CH), 125.97 (C$_q$), 125.77 (CH), 121.69 (CH), 121.59 (CH), 120.54 (CH), 120.28 (CH), 110.74 (CH), 100.56 (CH), 43.25 (CH$_2$), 33.75 (CH), 20.55 (CH$_3$). **IR** (ATR, cm^{-1}) γ 3057, 3022, 2963, 2927, 1560, 1491, 1456, 1407, 1091, 1014, 829, 803, 789, 749. **HRMS** (ESI) m/z (M+H)$^+$: calculated for $(C_{26}H_{22}ClN_2)^+$: 397.1466, found: 397.1462; **$[\alpha]_D^{25}$** = +132.7 (c = 0.15, CHCl$_3$); The product was analyzed by HPLC to determine the enantiomeric excess: 91% e.e. (CHIRALPAK IC-3, *n*-hexane/*i*-PrOH = 95/5, flow rate: 1.0 mL/min, T = 20 °C, 250 nm), t_R (minor) = 9.05 min, t_R (major) = 15.38 min.

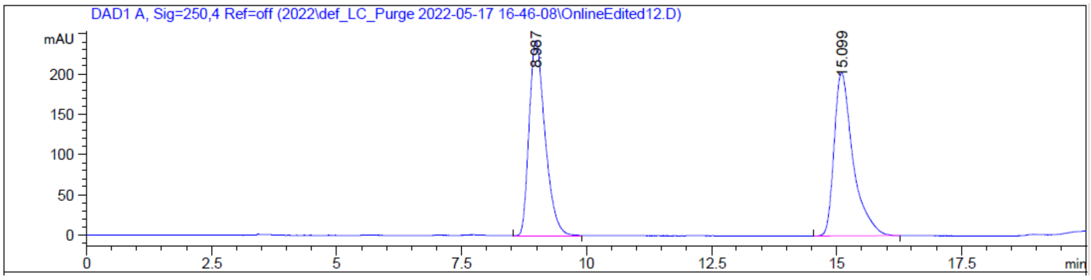

Peak #	RetTime [min]	Type	Width [min]	Area [mAU*s]	Height [mAU]	Area %
1	8.987	VV R	0.3336	5349.94189	242.30664	50.0637
2	15.099	BV R	0.3908	5336.33008	202.94223	49.9363

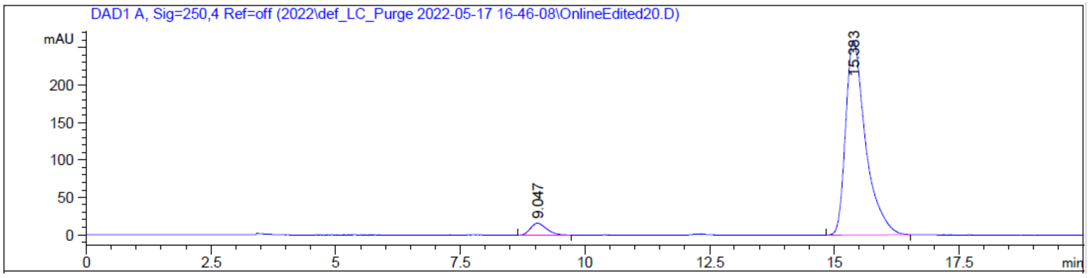

Peak #	RetTime [min]	Type	Width [min]	Area [mAU*s]	Height [mAU]	Area %
1	9.047	BV R	0.2672	358.80383	15.90459	4.7345
2	15.383	BV R	0.3983	7219.72803	259.71753	95.2655

(R_a, R)-4-(2-(1-(Isoquinolin-1-yl)-1*H*-indol-2-yl)propyl)phenol (3n)

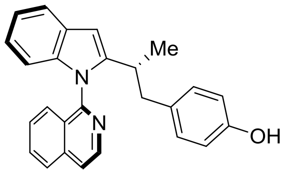

The general procedure was followed using 1-(1*H*-indol-1-yl)isoquinoline (0.1 mmol, 24.4 mg) and 4-allylphenol (0.3 mmol, 40.2 mg) to afford **3n** (30.2 mg, 80% yield, >95:5 d.r., b:l >95:5) as a colorless oil. **^{1}H NMR (400 MHz, $CDCl_3$)** δ 8.59 (d, J = 5.6 Hz, 1H), 7.95 (d, J = 8.3 Hz, 1H), 7.82 (d, J = 5.7 Hz, 1H), 7.71 (t, J = 7.5 Hz, 1H), 7.64 (d, J = 7.8 Hz, 1H), 7.40 (t, J = 7.6 Hz, 1H), 7.30 (d, J = 8.5 Hz, 1H), 7.12 (t, J = 7.4 Hz, 1H), 6.99 (t, J = 7.6 Hz, 1H), 6.67 (d, J = 8.2 Hz, 1H), 6.60 (s, 1H), 6.47 (d, J = 7.5 Hz, 2H), 6.40 (d, J = 7.7 Hz, 2H), 5.37 (brs, 1H), 3.19 – 3.03 (m, 1H), 2.65 (dd, J = 13.5, 6.0 Hz, 1H), 2.42 (dd, J = 13.4, 8.2 Hz, 1H), 1.26 (d, J = 6.7 Hz, 3H). **^{13}C NMR (101 MHz, $CDCl_3$)** δ 153.93 (C_q), 150.67 (C_q), 147.42 (C_q), 141.79 (CH), 138.68 (C_q), 138.31 (C_q), 132.03 (C_q), 131.16 (CH), 129.99 (CH), 128.53 (CH), 128.47 (C_q), 126.99 (CH), 126.10 (C_q), 126.00 (CH), 121.71 (CH), 121.62 (CH), 120.50 (CH), 120.27 (CH), 114.96 (CH), 110.62 (CH), 100.49 (CH), 42.86 (CH_2), 33.80 (CH), 19.93 (CH_3). **IR** (ATR, cm^{-1}) γ 3058, 3037, 2963, 2926, 1514, 1456, 1408, 1374, 1233, 1215, 830, 748. **HRMS** (ESI) m/z $(M+H)^+$: calculated for $(C_{26}H_{23}N_2O)^+$: 379.1805, found: 379.1808; **$[\alpha]_D^{25}$** = +133.0 (c = 0.37, $CHCl_3$); The product was analyzed by HPLC to determine the enantiomeric excess: 91% e.e. (CHIRALPAK IC-3, *n*-hexane/*i*-PrOH = 90/10, flow rate: 1.0 mL/min, T = 20 °C, 254 nm), t_R (minor) = 10.80 min, t_R (major) = 16.99 min.

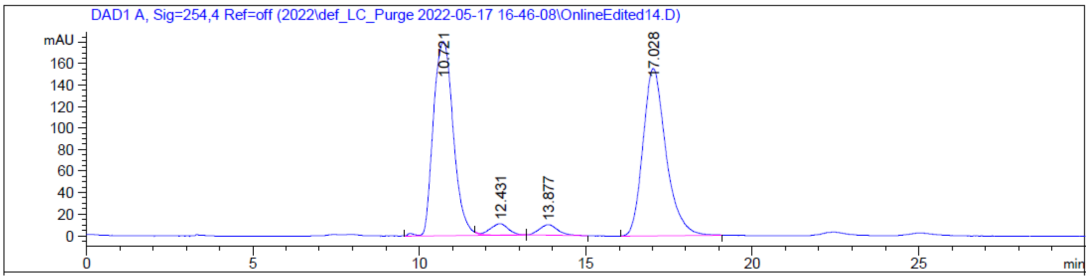

Peak #	RetTime [min]	Type	Width [min]	Area [mAU*s]	Height [mAU]	Area %
1	10.721	VV R	0.6575	7520.60254	180.70041	47.7100
2	12.431	VB E	0.4383	393.93823	10.63205	2.4991
3	13.877	BB	0.4570	385.09778	9.96222	2.4430
4	17.028	BB	0.6939	7463.50342	155.50668	47.3478

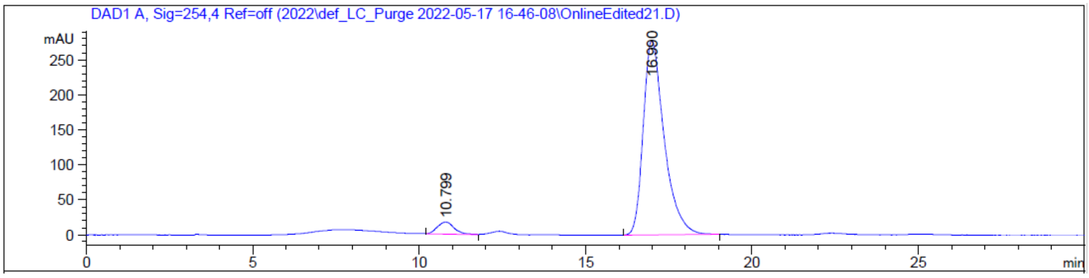

Peak #	RetTime [min]	Type	Width [min]	Area [mAU*s]	Height [mAU]	Area %
1	10.799	BB	0.4204	586.16608	17.16573	4.6055
2	16.990	BB	0.6443	1.21413e4	277.58237	95.3945

(*R*$_a$, *R*)-1-(2-(1-([1,1'-Biphenyl]-4-yl)propan-2-yl)-1*H*-indol-1-yl)isoquinoline (3o)

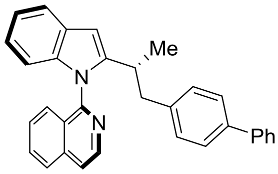

The general procedure was followed using 1-(1*H*-indol-1-yl)isoquinoline (0.1 mmol, 24.4 mg) and 4-allyl-1,1'-biphenyl (0.3 mmol, 58.2 mg) to afford **3o** (38.1 mg, 87% yield, >95:5 d.r., b:l >95:5) as a colorless oil. **^{1}H NMR (400 MHz, $CDCl_3$)** δ 8.63 (d, *J* = 5.6 Hz, 1H), 7.95 (d, *J* = 8.3 Hz, 1H), 7.84 (d, *J* = 5.6 Hz, 1H), 7.72 – 7.63 (m, 2H), 7.48 (d, *J* = 7.7 Hz, 2H), 7.40 (t, *J* = 7.4 Hz, 2H), 7.35 – 7.30 (m, 3H), 7.30 – 7.23 (m, 2H), 7.14 (t, *J* = 7.4 Hz, 1H), 7.01 (t, *J* = 7.6 Hz, 1H), 6.76 (d, *J* = 7.6 Hz, 2H), 6.73 – 6.65 (m, 2H), 3.38 – 3.15 (m, 1H), 2.82 (dd, *J* = 13.5, 6.2 Hz, 1H), 2.59 (dd, *J* = 13.5, 7.9 Hz, 1H), 1.37 (d, *J* = 6.7 Hz, 3H). **^{13}C NMR (101 MHz, $CDCl_3$)** δ 150.64 (C_q), 147.31 (C_q), 141.99 (CH), 140.97 (C_q), 139.32 (C_q), 138.81 (C_q), 138.70 (C_q), 138.30 (C_q), 131.10 (CH), 129.42 (CH), 128.80 (CH), 128.49 (C_q), 128.47 (CH), 127.14 (CH), 127.01 (CH), 126.99 (CH), 126.78 (CH), 126.08 (C_q), 125.91 (CH), 121.66 (CH), 121.62 (CH), 120.52 (CH), 120.29 (CH), 110.68 (CH), 100.54 (CH), 43.43 (CH_2), 33.75 (CH), 20.29 (CH_3). **IR** (ATR, cm^{-1}) *γ* 3057, 3025, 2962, 2926, 1560, 1486, 1456, 1407, 829, 763, 748, 697. **HRMS** (ESI) m/z $(M+H)^+$: calculated for $(C_{32}H_{27}N_2)^+$: 439.2169, found: 439.2165; **$[\alpha]_D^{25}$** = +130.0 (c = 0.20, $CHCl_3$); The product was analyzed by HPLC to determine the enantiomeric excess: 89% e.e. (CHIRALPAK IC-3, *n*-hexane/*i*-PrOH = 90/10, flow rate: 1.0 mL/min, T = 20 °C, 254 nm), t_R (minor) = 8.49 min, t_R (major) = 14.30 min.

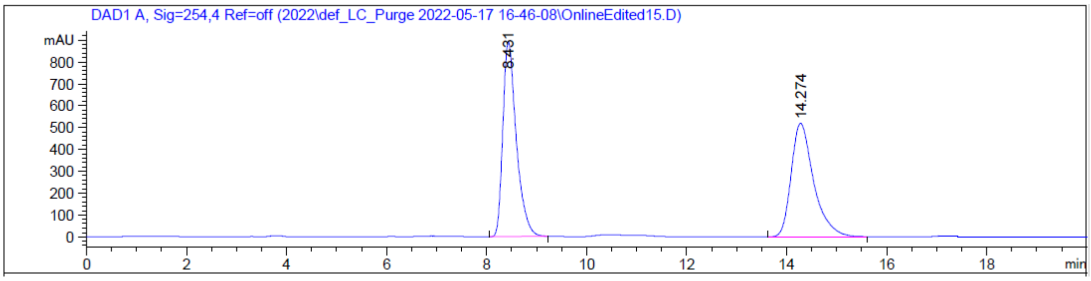

Peak #	RetTime [min]	Type	Width [min]	Area [mAU*s]	Height [mAU]	Area %
1	8.431	BB	0.2834	1.66352e4	892.82367	50.7354
2	14.274	BB	0.4672	1.61530e4	518.82721	49.2646

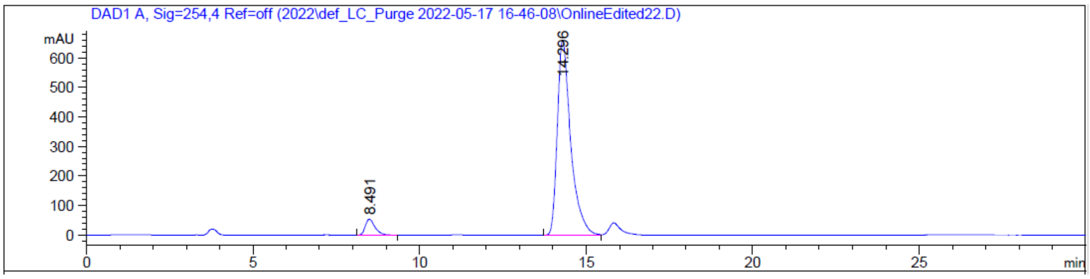

Peak #	RetTime [min]	Type	Width [min]	Area [mAU*s]	Height [mAU]	Area %
1	8.491	BB	0.3065	1095.87280	54.05001	5.5938
2	14.296	BV	0.4193	1.84949e4	659.34137	94.4062

(*R*$_a$, *R*)-1-(2-(1-(4-Methoxyphenyl)propan-2-yl)-1*H*-indol-1-yl)isoquinoline (3p)

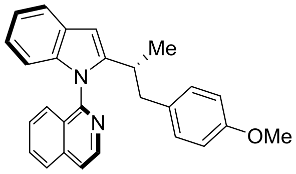

The general procedure was followed using 1-(1*H*-indol-1-yl)isoquinoline (0.1 mmol, 24.4 mg) and 1-allyl-4-methoxybenzene (0.3 mmol, 44.4 mg) to afford **3p** (26.7 mg, 68% yield, >95:5 d.r., b:l >95:5) as a colorless oil. **^{1}H NMR (400 MHz, $CDCl_3$)** δ 8.61 (d, *J* = 5.7 Hz, 1H), 7.96 (d, *J* = 8.3 Hz, 1H), 7.82 (d, *J* = 5.7 Hz, 1H), 7.72 (t, *J* = 7.5 Hz, 1H), 7.66 (d, *J* = 7.8 Hz, 1H), 7.40 (t, *J* = 7.6 Hz, 1H), 7.32 (d, *J* = 8.5 Hz, 1H), 7.13 (t, *J* = 7.4 Hz, 1H), 7.00 (t, *J* = 7.6 Hz, 1H), 6.68 (d, *J* = 8.2 Hz, 1H), 6.63 (s, 1H), 6.63 – 6.52 (m, 4H), 3.69 (s, 3H), 3.26 – 3.09 (m, 1H), 2.70 (dd, *J* = 13.5, 6.1 Hz, 1H), 2.47 (dd, *J* = 13.6, 8.0 Hz, 1H), 1.31 (d, *J* = 6.8 Hz, 3H).

^{13}C NMR (101 MHz, CDCl$_3$) δ 157.83 (C_q), 150.69 (C_q), 147.47 (C_q), 142.05 (CH), 138.68 (C_q), 138.27 (C_q), 132.23 (C_q), 131.01 (CH), 129.87 (CH), 128.49 (C_q), 128.37 (CH), 126.98 (CH), 126.07 (C_q), 126.00 (CH), 121.57 (CH), 121.54 (CH), 120.45 (CH), 120.23 (CH), 113.49 (CH), 110.66 (CH), 100.42 (CH), 55.22 (CH_3), 42.88 (CH_2), 33.82 (CH), 20.03 (CH_3). **IR** (ATR, cm^{-1}) γ 3057, 2962, 2928, 2831, 1511, 1456, 1408, 1247, 828, 748. **HRMS** (ESI) m/z $(M+H)^+$: calculated for $(C_{27}H_{25}N_2O)^+$: 393.1961, found: 393.1957; $[\alpha]_D^{25}$ = +114.8 (c = 0.31, $CHCl_3$); The product was analyzed by HPLC to determine the enantiomeric excess: 88% e.e. (CHIRALPAK ID-3, *n*-hexane/*i*-PrOH = 95/5, flow rate: 1.0 mL/min, T = 20 °C, 250 nm), t_R (minor) = 15.87 min, t_R (major) = 23.24 min.

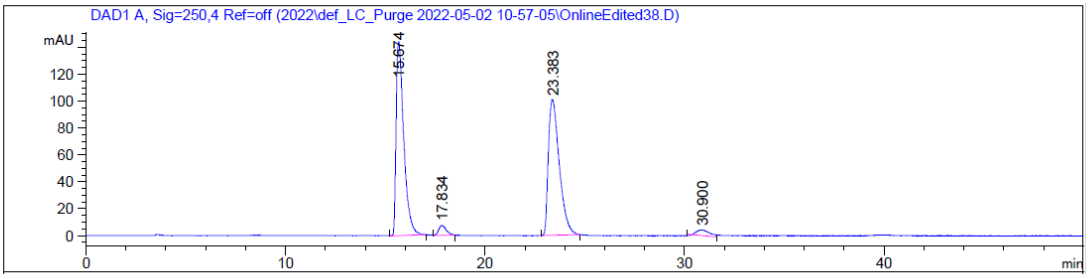

Peak #	RetTime [min]	Type	Width [min]	Area [mAU*s]	Height [mAU]	Area %
1	15.674	MM R	0.4396	3836.50293	144.22986	47.6822
2	17.834	VV R	0.3075	192.24365	7.46276	2.3893
3	23.383	VV R	0.4508	3838.60938	100.90055	47.7084
4	30.900	MM R	0.7095	178.63292	4.19609	2.2201

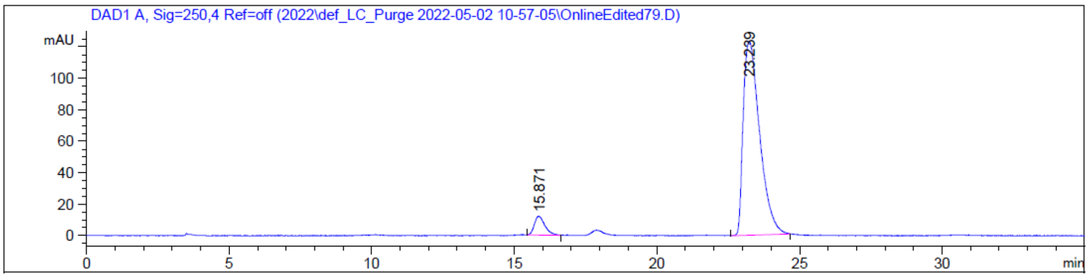

Peak #	RetTime [min]	Type	Width [min]	Area [mAU*s]	Height [mAU]	Area %
1	15.871	BV R	0.3086	307.45047	12.01386	5.8572
2	23.239	VV R	0.4803	4941.63525	123.36059	94.1428

Ethyl (R_a, R)-4-(2-(1-(isoquinolin-1-yl)-1H-indol-2-yl)propyl)benzoate (3q)

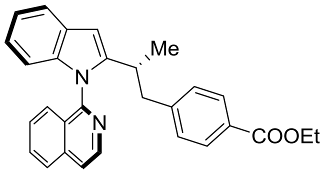

The general procedure was followed using 1-(1*H*-indol-1-yl)isoquinoline (0.1 mmol, 24.4 mg) and ethyl 4-allylbenzoate (0.3 mmol, 57.0 mg) to afford **3q** (23.0 mg, 53% yield, >95:5 d.r., b:l >95:5) as a colorless oil. **^{1}H NMR (400 MHz, $CDCl_3$)** δ 8.64 (d, J = 5.6 Hz, 1H), 7.98 (d, J = 8.3 Hz, 1H), 7.85 (d, J = 5.6 Hz, 1H), 7.74 (t, J = 7.4 Hz, 1H), 7.69 (d, J = 7.9 Hz, 3H), 7.39 (t, J = 7.7 Hz, 1H), 7.29 (d, J = 5.8 Hz, 1H), 7.16 (t, J = 7.4 Hz, 1H), 7.03 (t, J = 7.7 Hz, 1H), 6.76 (d, J = 7.5 Hz, 2H), 6.70 (d, J = 8.3 Hz, 1H), 6.67 (s, 1H), 4.36 (q, J = 7.0 Hz, 2H), 3.38 – 3.23 (m, 1H), 2.83 (dd, J = 13.3, 6.7 Hz, 1H), 2.65 (dd, J = 13.3, 7.6 Hz, 1H), 1.40 (t, J = 7.0 Hz, 3H), 1.39 (d, J = 7.0 Hz, 3H). **^{13}C NMR (101 MHz, $CDCl_3$)** δ 166.59 (C_q), 150.51 (C_q), 146.76 (C_q), 145.48 (C_q), 142.02 (CH), 138.60 (C_q), 138.25 (C_q), 131.04 (CH), 129.41 (CH), 128.88 (CH), 128.39 (CH), 128.36 (C_q), 128.27 (C_q), 127.04 (CH), 125.91 (C_q), 125.86 (CH), 121.73 (CH), 121.61 (CH), 120.56 (CH), 120.30 (CH), 110.74 (CH), 100.68 (CH), 60.85 (CH_2), 43.89 (CH_2), 33.53 (CH), 20.38 (CH_3), 14.49 (CH_3). **IR** (ATR, cm^{-1}) γ 3057, 2978, 2927, 1715, 1456, 1405, 1276, 1106, 1021, 749. **HRMS** (ESI) m/z $(M+H)^+$: calculated for $(C_{29}H_{27}N_2O_2)^+$: 435.2067, found: 435.2068; $[\alpha]_D^{25}$ = +103.8 (c = 0.34, $CHCl_3$); The product was analyzed by HPLC to determine the enantiomeric excess: 87% e.e. (CHIRALPAK IC-3, *n*-hexane/*i*-PrOH = 90/10, flow rate: 1.0 mL/min, T = 20 °C, 254 nm), t_R (minor) = 15.24 min, t_R (major) = 24.26 min.

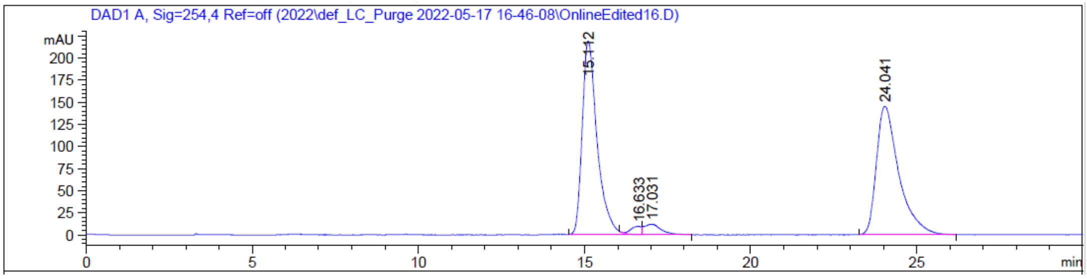

Peak #	RetTime [min]	Type	Width [min]	Area [mAU*s]	Height [mAU]	Area %
1	15.112	BV R	0.4670	6804.11377	218.69150	47.8460
2	16.633	VV E	0.2616	200.78458	9.16463	1.4119
3	17.031	VB E	0.4248	416.30902	11.59663	2.9275
4	24.041	BB	0.6568	6799.65283	145.07655	47.8146

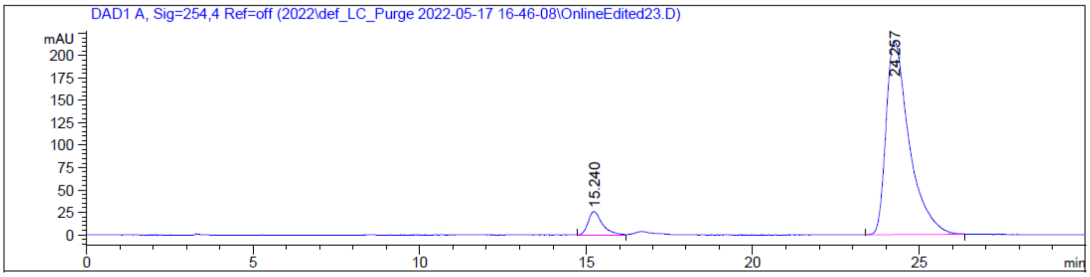

Peak #	RetTime [min]	Type	Width [min]	Area [mAU*s]	Height [mAU]	Area %
1	15.240	BB	0.3998	751.16699	25.78905	6.7293
2	24.257	BB	0.6947	1.04114e4	216.61932	93.2707

(*R*$_a$, *R*)-1-(2-(1-(3-Chlorophenyl)propan-2-yl)-1*H*-indol-1-yl)isoquinoline (3r)

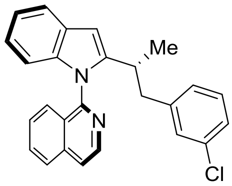

The general procedure was followed using 1-(1*H*-indol-1-yl)isoquinoline (0.1 mmol, 24.4 mg) and 1-allyl-3-chlorobenzene (0.3 mmol, 45.6 mg) to afford **3r** (36.0 mg, 91% yield, >95:5 d.r., b:l >95:5) as a colorless oil. **^{1}H NMR (400 MHz, CDCl$_3$)** δ 8.62 (d, *J* = 5.6 Hz, 1H), 7.97 (d, *J* = 8.3 Hz, 1H), 7.84 (d, *J* = 5.7 Hz, 1H), 7.73 (t, *J* = 7.6 Hz, 1H), 7.67 (d, *J* = 7.8 Hz, 1H), 7.43 (t, *J* = 7.7 Hz, 1H), 7.29 (d, *J* = 8.5 Hz, 1H), 7.14 (t, *J* = 7.4 Hz, 1H), 7.06 – 6.89 (m, 3H), 6.69 (d, *J* = 8.2 Hz, 1H), 6.66 – 6.57 (m, 3H), 3.33 – 3.14 (m, 1H), 2.74 (dd, *J* = 13.6, 6.5 Hz, 1H), 2.53 (dd, *J* = 13.6, 7.8 Hz, 1H), 1.34 (d, *J* = 6.8 Hz, 3H). **^{13}C NMR (101 MHz, CDCl$_3$)** δ 150.51 (C$_q$), 146.79 (C$_q$), 142.23 (C$_q$), 142.04 (CH), 138.68 (C$_q$), 138.31 (C$_q$), 133.88 (C$_q$), 131.12 (CH), 129.31 (CH), 128.93 (CH), 128.53 (CH), 128.37 (C$_q$), 127.19 (CH), 127.10 (CH), 126.19 (CH), 126.00 (C$_q$), 125.74 (CH), 121.75 (CH), 121.70 (CH), 120.54 (CH), 120.30 (CH), 110.72 (CH), 100.55 (CH), 43.38 (CH$_2$), 33.55 (CH), 20.30 (CH$_3$). **IR** (ATR, cm^{-1}) *γ* 3057, 2963, 2927, 2874, 1561, 1456, 1407, 828, 779, 749, 686. **HRMS** (ESI) m/z (M+H)$^+$: calculated for $(C_{26}H_{22}ClN_2)^+$: 397.1466, found: 397.1464; **$[\alpha]_D^{25}$** = +122.7 (c = 0.52, CHCl$_3$); The product was analyzed by HPLC to determine the enantiomeric excess: 93% e.e. (CHIRALPAK IC-3, *n*-hexane/*i*-PrOH = 95/5, flow rate: 1.0 mL/min, T = 20 °C, 250 nm), t$_R$ (minor) = 8.91 min, t$_R$ (major) = 13.74 min.

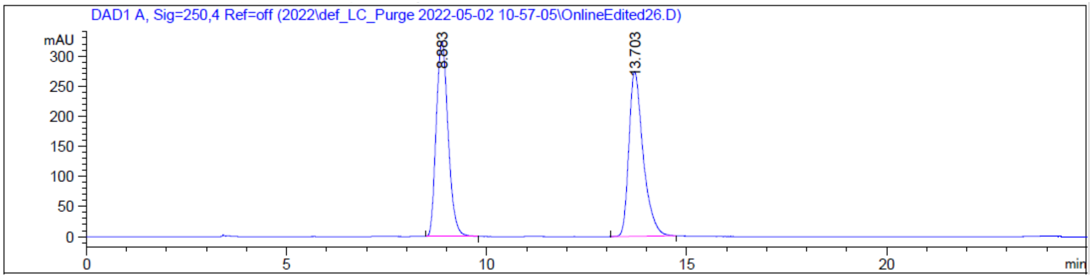

Peak #	RetTime [min]	Type	Width [min]	Area [mAU*s]	Height [mAU]	Area %
1	8.883	BV R	0.3208	6712.37451	323.80896	49.7749
2	13.703	VV R	0.3671	6773.07422	273.31134	50.2251

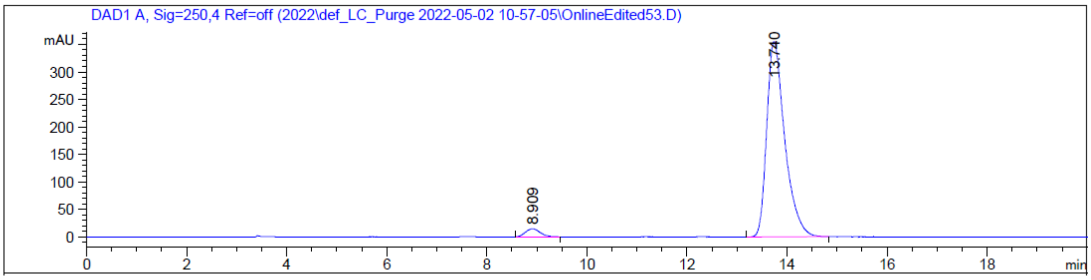

Peak #	RetTime [min]	Type	Width [min]	Area [mAU*s]	Height [mAU]	Area %
1	8.909	BV R	0.2424	304.34155	14.89221	3.3099
2	13.740	VV R	0.3675	8890.61719	354.60275	96.6901

(*R*$_a$, *R*)-1-(2-(1-(2-Bromophenyl)propan-2-yl)-1*H*-indol-1-yl)isoquinoline (3s)

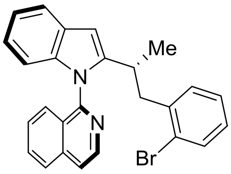

The general procedure was followed using 1-(1*H*-indol-1-yl)isoquinoline (0.1 mmol, 24.4 mg) and 1-allyl-2-bromobenzene (0.3 mmol, 58.8 mg) to afford **3s** (27.3 mg, 62% yield, >95:5 d.r., b:l >95:5) as a colorless oil. **^{1}H NMR (400 MHz, CDCl$_3$)** δ 8.59 (d, *J* = 5.7 Hz, 1H), 7.95 (d, *J* = 8.3 Hz, 1H), 7.80 (d, *J* = 5.7 Hz, 1H), 7.77 – 7.64 (m, 2H), 7.36 (t, *J* = 7.7 Hz, 1H), 7.25 (d, *J* = 7.5 Hz, 1H), 7.19 (d, *J* = 7.9 Hz, 1H), 7.13 (t, *J* = 7.4 Hz, 1H), 7.06 – 6.95 (m, 2H), 6.94 – 6.83 (m, 2H), 6.70 (s, 1H), 6.68 (d, *J* = 8.3 Hz, 1H), 3.45 – 3.24 (m, 1H), 2.96 (dd, *J* = 13.3, 6.4 Hz, 1H), 2.76 (dd, *J* = 13.2, 8.7 Hz, 1H), 1.31 (d, *J* = 6.8 Hz, 3H). **^{13}C NMR (101 MHz, CDCl$_3$)**

δ 150.65 (C_q), 147.40 (C_q), 142.25 (CH), 139.32 (C_q), 138.68 (C_q), 138.31 (C_q), 132.73 (CH), 131.77 (CH), 130.95 (CH), 128.46 (C_q), 128.45 (CH), 127.81 (CH), 127.06 (CH), 126.94 (CH), 126.41 (C_q), 125.83 (CH), 124.47 (C_q), 121.65 (CH), 121.60 (CH), 120.49 (CH), 120.24 (CH), 110.71 (CH), 100.24 (CH), 44.08 (CH_2), 31.46 (CH), 20.31 (CH_3). **IR** (ATR, cm^{-1}) γ 3059, 2962, 2927, 1561, 1457, 1405, 1027, 748. **HRMS** (ESI) m/z $(M+H)^+$: calculated for $(C_{26}H_{22}BrN_2)^+$: 441.0961, found: 441.0957; $[\alpha]_D^{25}$ = +100.0 (c = 0.31, $CHCl_3$); The product was analyzed by HPLC to determine the enantiomeric excess: 92% e.e. (CHIRALPAK IC-3, *n*-hexane/*i*-PrOH = 95/5, flow rate: 1.0 mL/min, T = 20 °C, 250 nm), t_R (minor) = 10.08 min, t_R (major) = 19.35 min.

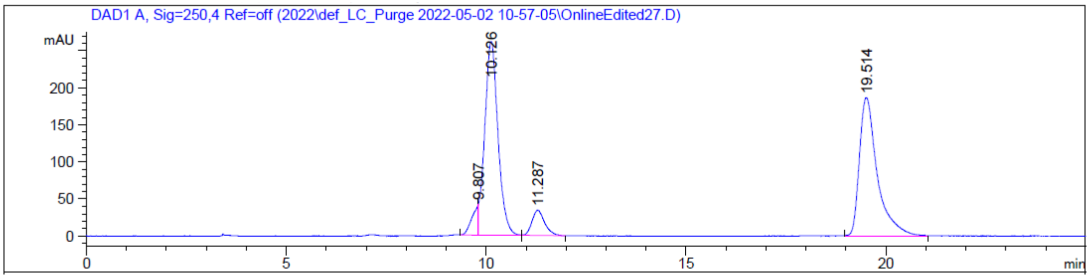

Peak #	RetTime [min]	Type	Width [min]	Area [mAU*s]	Height [mAU]	Area %
1	9.807	MF R	0.1722	462.85767	42.09248	3.5585
2	10.126	FM R	0.3482	6020.56641	261.63965	46.2865
3	11.287	VV R	0.2663	761.03882	34.24734	5.8509
4	19.514	BV R	0.4373	5762.71777	186.59494	44.3041

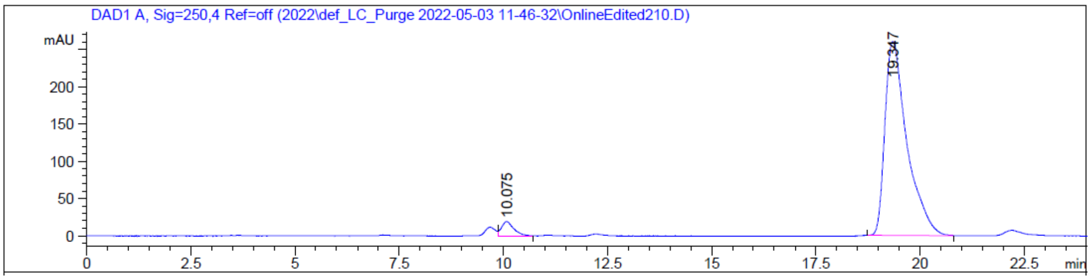

Peak #	RetTime [min]	Type	Width [min]	Area [mAU*s]	Height [mAU]	Area %
1	10.075	VV R	0.2527	393.44550	19.22823	3.9496
2	19.347	VV R	0.5046	9568.22559	260.43936	96.0504

(R_a, *R*)-1-(2-(1-(3,4-Dimethoxyphenyl)propan-2-yl)-1*H*-indol-1-yl)isoquinoline (3t)

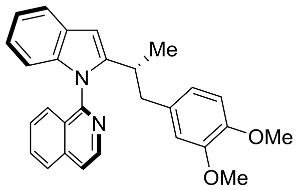

The general procedure was followed using 1-(1*H*-indol-1-yl)isoquinoline (0.1 mmol, 24.4 mg) and 4-allyl-1,2-dimethoxybenzene (0.3 mmol, 53.4 mg) to afford **3t** (19.8 mg, 47% yield, >95:5 d.r., b:l >95:5) as a colorless oil. **^{1}H NMR (400 MHz, $CDCl_3$)** δ 8.60 (d, *J* = 5.6 Hz, 1H), 7.95 (d, *J* = 8.3 Hz, 1H), 7.80 (d, *J* = 5.6 Hz, 1H), 7.72 (t, *J* = 7.6 Hz, 1H), 7.65 (d, *J* = 7.9 Hz, 1H), 7.38 (t, *J* = 7.7 Hz, 1H), 7.25 (d, *J* = 5.7 Hz, 1H), 7.12 (t, *J* = 7.4 Hz, 1H), 6.98 (t, *J* = 7.6 Hz, 1H), 6.66 (d, *J* = 8.4 Hz, 1H), 6.64 (s, 1H), 6.51 (d, *J* = 8.1 Hz, 1H), 6.29 (d, *J* = 7.8 Hz, 1H), 6.02 (s, 1H), 3.75 (s, 3H), 3.50 (s, 3H), 3.29 – 3.05 (m, 1H), 2.68 (dd, *J* = 13.5, 6.4 Hz, 1H), 2.48 (dd, *J* = 13.6, 7.8 Hz, 1H), 1.34 (d, *J* = 6.8 Hz, 3H). **^{13}C NMR (101 MHz, $CDCl_3$)** δ 150.75 (C_q), 148.44 (C_q), 147.39 (C_q), 147.18 (C_q), 142.02 (CH), 138.61 (C_q), 138.24 (C_q), 132.75 (C_q), 131.10 (CH), 128.50 (C_q), 128.35 (CH), 126.89 (CH), 126.05 (C_q), 125.96 (CH), 121.60 (CH), 121.48 (CH), 121.03 (CH), 120.50 (CH), 120.23 (CH), 111.83 (CH), 110.73 (CH), 110.67 (CH), 100.46 (CH), 55.85 (CH_3), 55.57 (CH_3), 43.69 (CH_2), 34.01 (CH), 20.19 (CH_3). **IR** (ATR, cm^{-1}) *γ* 2961, 2933, 1515, 1456, 1405, 1260, 1237, 1157, 1140, 1029, 749. **HRMS** (ESI) m/z $(M+H)^+$: calculated for $(C_{28}H_{27}N_2O_2)^+$: 423.2067, found: 423.2068; $[\alpha]_D^{25}$ = +91.8 (c = 0.11, $CHCl_3$); The product was analyzed by HPLC to determine the enantiomeric excess: 91% e.e. (CHIRALPAK ID-3, *n*-hexane/*i*-PrOH = 90/10, flow rate: 1.0 mL/min, T = 20 °C, 250 nm), t_R (minor) = 22.75 min, t_R (major) = 36.55 min.

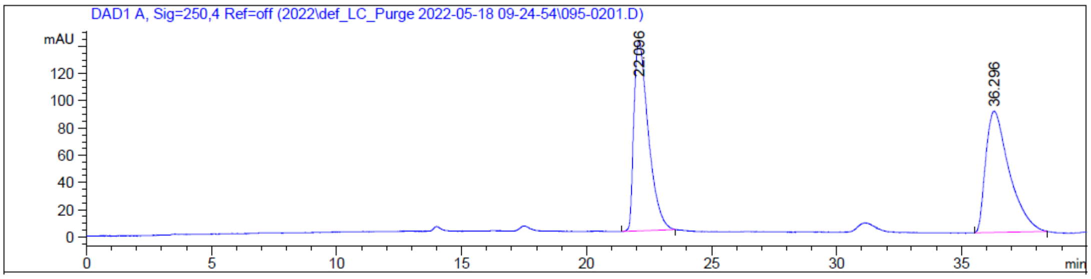

Peak #	RetTime [min]	Type	Width [min]	Area [mAU*s]	Height [mAU]	Area %
1	22.096	BB	0.5234	5603.88330	139.59077	49.0654
2	36.296	BV R	0.7719	5817.36035	88.96185	50.9346

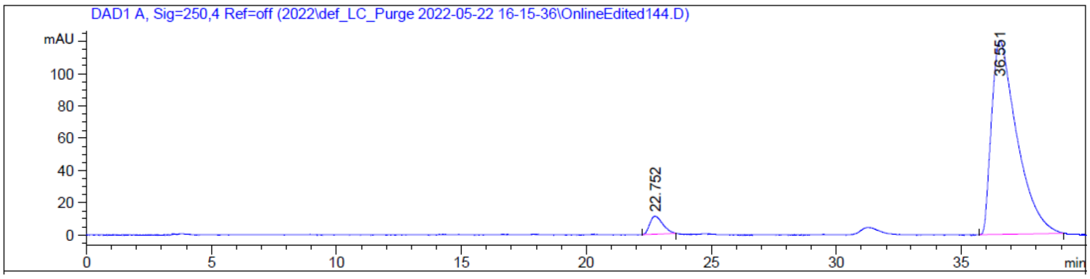

Peak #	RetTime [min]	Type	Width [min]	Area [mAU*s]	Height [mAU]	Area %
1	22.752	BB	0.4333	411.70090	11.31999	4.6171
2	36.551	BV R	0.8305	8505.26172	120.49277	95.3829

(R_a, *R*)-1-(2-(1-(Naphthalen-2-yl)propan-2-yl)-1*H*-indol-1-yl)isoquinoline (3u)

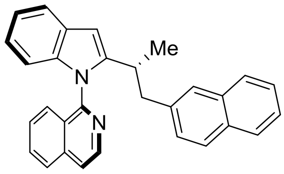

The general procedure was followed using 1-(1*H*-indol-1-yl)isoquinoline (0.1 mmol, 24.4 mg) and 2-allylnaphthalene (0.3 mmol, 50.4 mg) to afford **3u** (33.8 mg, 82% yield, >95:5 d.r., b:l >95:5) as a colorless oil. **^{1}H NMR (400 MHz, $CDCl_3$)** δ 8.62 (d, *J* = 5.7 Hz, 1H), 7.91 (d, *J* = 8.3 Hz, 1H), 7.82 (d, *J* = 5.7 Hz, 1H), 7.72 – 7.60 (m, 2H), 7.59 (ddd, *J* = 8.2, 6.7, 1.4 Hz, 1H), 7.57 – 7.47 (m, 1H), 7.44 (d, *J* = 8.4 Hz, 1H), 7.42 – 7.29 (m, 2H), 7.18 – 7.00 (m, 4H), 6.98 (ddd, *J* = 8.2, 7.1, 1.2 Hz, 1H), 6.74 (dd, *J* = 8.4, 1.7 Hz, 1H), 6.70 (s, 1H), 6.65 (dd, *J* = 8.2, 0.9 Hz, 1H), 3.45 – 3.24 (m, 1H), 2.93 (dd, *J* = 13.4, 6.6 Hz, 1H), 2.73 (dd, *J* = 13.4, 7.7 Hz, 1H), 1.39 (d, *J* = 6.8 Hz, 3H). **^{13}C NMR (101 MHz, $CDCl_3$)** δ 150.62 (C_q), 147.38 (C_q), 141.96 (CH), 138.64 (C_q), 138.21 (C_q), 137.74 (C_q), 133.41 (C_q), 132.04 (C_q), 131.00 (CH), 128.48 (C_q), 128.22 (CH), 127.60 (CH), 127.58 (CH), 127.50 (CH), 127.45 (CH), 127.27 (CH), 126.81 (CH), 126.00 (C_q), 125.83 (CH), 125.71 (CH), 125.20 (CH), 121.60 (CH), 121.58 (CH), 120.48 (CH), 120.27 (CH), 110.70 (CH), 100.47 (CH), 44.16 (CH_2), 33.72 (CH), 20.55 (CH_3). **IR** (ATR, cm^{-1}) γ 3048, 2966, 2932, 1560, 1456, 1407, 815, 748, 479, 468, 419, 410. **HRMS** (ESI) m/z $(M+H)^+$: calculated for $(C_{30}H_{25}N_2)^+$: 413.2012, found: 413.2014; $[\alpha]_D^{25}$ = +106.5 (c = 0.20, $CHCl_3$); The product was analyzed by HPLC to determine the enantiomeric excess: 91% e.e. (CHIRALPAK IC-3, *n*-hexane/*i*-PrOH = 95/5, flow rate: 1.0 mL/min, T = 20 °C, 250 nm), t_R (minor) = 11.25 min, t_R (major) = 20.85 min.

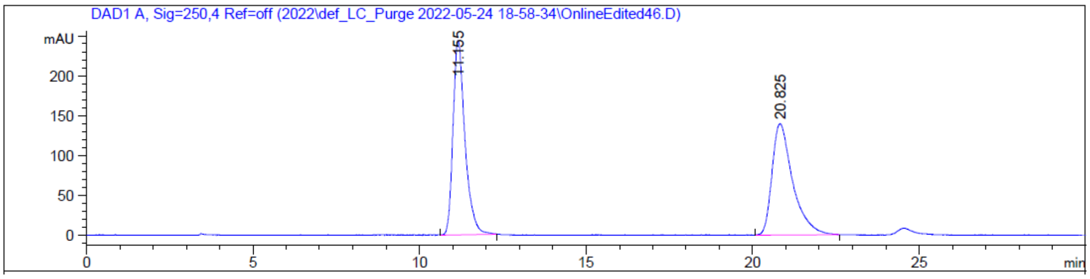

Peak #	RetTime [min]	Type	Width [min]	Area [mAU*s]	Height [mAU]	Area %
1	11.155	BV R	0.3667	6075.31641	243.85959	50.2681
2	20.825	BV R	0.5254	6010.51953	139.58661	49.7319

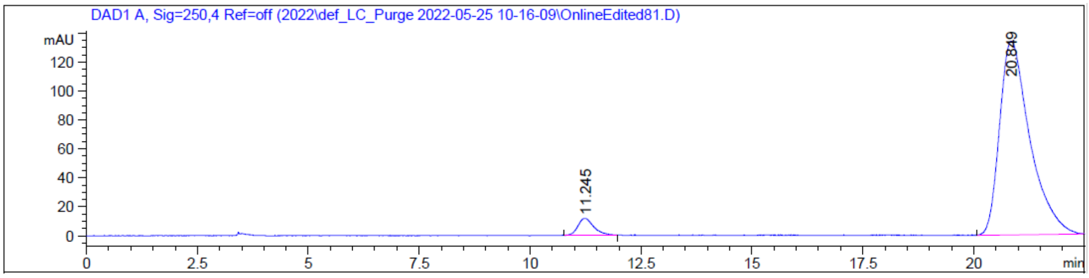

Peak #	RetTime [min]	Type	Width [min]	Area [mAU*s]	Height [mAU]	Area %
1	11.245	BV R	0.2936	288.50061	11.70361	4.3409
2	20.849	BV R	0.6009	6357.52979	133.91025	95.6591

(R_a, *R*)-1-(2-(1-(Naphthalen-1-yl)propan-2-yl)-1*H*-indol-1-yl)isoquinoline (3v)

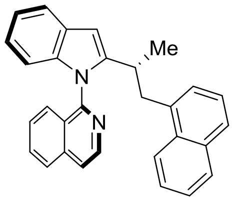

The general procedure was followed using 1-(1*H*-indol-1-yl)isoquinoline (0.1 mmol, 24.4 mg) and 1-allylnaphthalene (0.3 mmol, 50.4 mg) to afford **3v** (23.1 mg, 56% yield, >95:5 d.r., b:l >95:5) as a colorless oil. **^{1}H NMR (400 MHz, $CDCl_3$)** δ 8.59 (d, *J* = 5.7 Hz, 1H), 7.94 (d, *J* = 8.3 Hz, 1H), 7.81 (d, *J* = 5.7 Hz, 1H), 7.75 – 7.60 (m, 3H), 7.54 (d, *J* = 8.2 Hz, 1H), 7.42 – 7.29 (m, 2H), 7.31 – 7.19 (m, 1H), 7.25 – 7.15 (m, 1H), 7.21 – 7.09 (m, 1H), 7.15 – 7.05 (m, 1H), 7.01 (ddd, *J* = 8.2, 7.2, 1.2 Hz, 1H), 6.94 (ddd, *J* = 8.0, 6.7, 1.3 Hz, 1H), 6.86 (d, *J* = 8.3 Hz, 1H), 6.77 (s, 1H), 6.70 (dd, *J* = 8.2, 0.9 Hz, 1H), 3.49 – 3.35 (m, 1H), 3.33 (dd,

J = 13.3, 5.4 Hz, 1H), 2.96 (dd, *J* = 13.3, 9.2 Hz, 1H), 1.30 (d, *J* = 6.7 Hz, 3H). **^{13}C NMR (101 MHz, $CDCl_3$)** δ 150.68 (C_q), 148.17 (C_q), 142.24 (CH), 138.77 (C_q), 138.31 (C_q), 135.88 (C_q), 134.91 (C_q), 133.76 (C_q), 131.66 (C_q), 131.11 (CH), 128.68 (CH), 128.56 (CH), 127.80 (CH), 127.00 (CH), 126.20 (C_q), 125.66 (CH), 125.40 (CH), 125.30 (CH), 125.18 (CH), 123.29 (CH), 121.69 (CH), 121.58 (CH), 120.55 (CH), 120.29 (CH), 110.70 (CH), 100.15 (CH), 41.78 (CH_2), 32.23 (CH), 20.31 (CH_3). **IR** (ATR, cm^{-1}) γ 3058, 3047, 2964, 2927, 1560, 1456, 1406, 829, 790, 777, 749, 737. **HRMS** (ESI) m/z $(M+H)^+$: calculated for $(C_{30}H_{25}N_2)^+$: 413.2012, found: 413.2011; $[\alpha]_D^{25}$ = +63.8 (c = 0.16, $CHCl_3$); The product was analyzed by HPLC to determine the enantiomeric excess: 91% e.e. (CHIRALPAK ID-3, *n*-hexane/*i*-PrOH = 95/5, flow rate: 1.0 mL/min, T = 20 °C, 250 nm), t_R (minor) = 16.91 min, t_R (major) = 21.90 min.

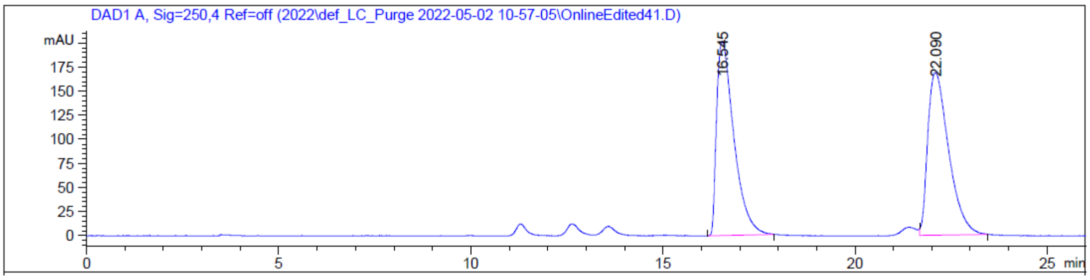

Peak #	RetTime [min]	Type	Width [min]	Area [mAU*s]	Height [mAU]	Area %
1	16.545	BV R	0.4419	6297.96338	201.89565	49.6412
2	22.090	FM R	0.6303	6388.99512	168.93452	50.3588

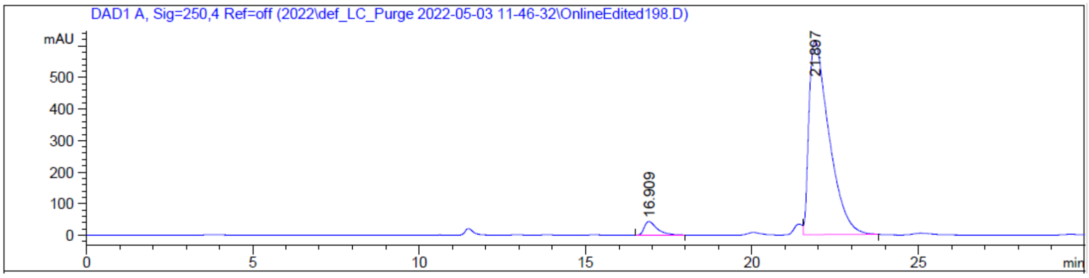

Peak #	RetTime [min]	Type	Width [min]	Area [mAU*s]	Height [mAU]	Area %
1	16.909	VV R	0.3223	1155.22522	43.15112	4.2760
2	21.897	FM R	0.6705	2.58615e4	616.10522	95.7240

(R_a, R)-1-(2-(1-(Perfluorophenyl)propan-2-yl)-1*H*-indol-1-yl)isoquinoline (3w)

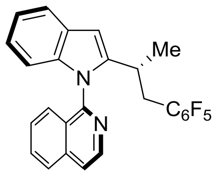

The general procedure was followed using 1-(1*H*-indol-1-yl)isoquinoline (0.1 mmol, 24.4 mg) and 1-allyl-2,3,4,5,6-pentafluorobenzene (0.3 mmol, 62.4 mg) to afford **3w** (22.9 mg, 51% yield, >95:5 d.r., b:l >95:5) as a white solid. M.p.: 123-124 °C. **^{1}H NMR (400 MHz, CDCl$_3$)** δ 8.59 (d, J = 5.7 Hz, 1H), 7.98 (d, J = 8.3 Hz, 1H), 7.82 (d, J = 5.7 Hz, 1H), 7.74 (t, J = 7.6 Hz, 1H), 7.67 (d, J = 7.8 Hz, 1H), 7.43 (t, J = 7.7 Hz, 1H), 7.27 (d, J = 11.6 Hz, 1H), 7.14 (t, J = 7.5 Hz, 1H), 7.01 (t, J = 7.6 Hz, 1H), 6.70 (s, 1H), 6.67 (d, J = 8.2 Hz, 1H), 3.54 – 3.31 (m, 1H), 2.81 (dd, J = 13.8, 7.3 Hz, 1H), 2.69 (dd, J = 13.8, 7.8 Hz, 1H), 1.41 (d, J = 6.8 Hz, 3H). **^{13}C NMR (101 MHz, CDCl$_3$)** δ 150.10 (C_q), 146.35 – 146.06 (m, C_q), 145.78 (C_q), 143.92 – 143.52 (m, C_q), 142.40 – 142.28 (m, C_q), 142.22 (CH), 138.61 (C_q), 138.42 – 138.33 (m, C_q), 138.31 (C_q), 136.12 – 135.57 (m, C_q), 131.09 (CH), 128.28 (C_q), 128.24 (CH), 127.24 (CH), 125.68 (C_q), 125.32 (CH), 122.00 (CH), 121.61 (CH), 120.69 (CH), 120.52 (CH), 113.41 – 112.73 (m, C_q), 110.92 (CH), 100.77 (CH), 31.04 (CH), 30.46 (CH_2), 20.37 (CH_3). **^{19}F NMR (282 MHz, CDCl$_3$)** δ -142.53 – -143.83 (m, 2F), -157.18 (t, J = 21.0 Hz, 1F), -162.29 – -163.42 (m, 2F). **IR** (ATR, cm^{-1}) γ 3057, 2963, 2927, 1520, 1503, 1456, 1407, 1123, 974, 827, 790, 749. **HRMS** (ESI) m/z $(M+H)^+$: calculated for $(C_{26}H_{18}F_5N_2)^+$: 453.1385, found: 453.1382; **$[\alpha]_D^{25}$** = +118.4 (c = 0.25, CHCl$_3$); The product was analyzed by HPLC to determine the enantiomeric excess: 83% e.e. (CHIRALPAK IC-3, *n*-hexane/*i*-PrOH = 95/5, flow rate: 1.0 mL/min, T = 20 °C, 250 nm), t_R (minor) = 6.38 min, t_R (major) = 9.77 min.

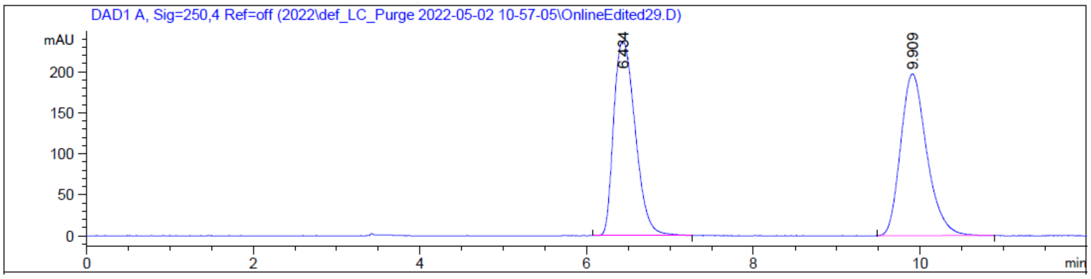

Peak #	RetTime [min]	Type	Width [min]	Area [mAU*s]	Height [mAU]	Area %
1	6.434	VV R	0.2813	4255.28613	237.18747	49.6983
2	9.909	BV R	0.3326	4306.95703	197.39548	50.3017

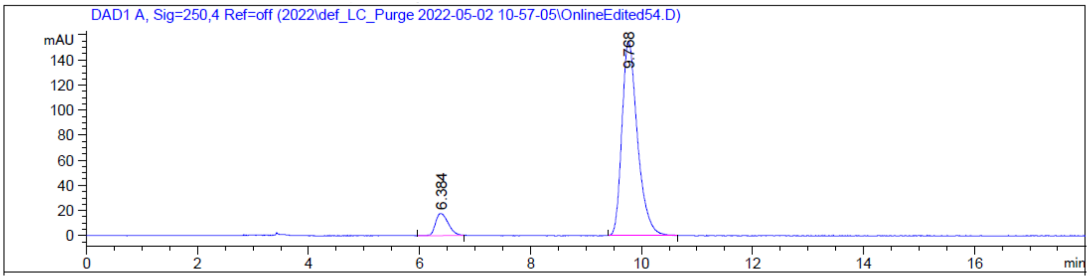

Peak #	RetTime [min]	Type	Width [min]	Area [mAU*s]	Height [mAU]	Area %
1	6.384	VV R	0.1922	278.19537	17.52824	8.4584
2	9.768	BV R	0.2887	3010.79224	154.97589	91.5416

16. X-ray single crystal data for 3c and 3w

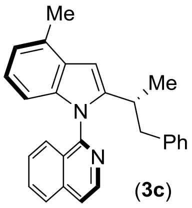

(**3c**)

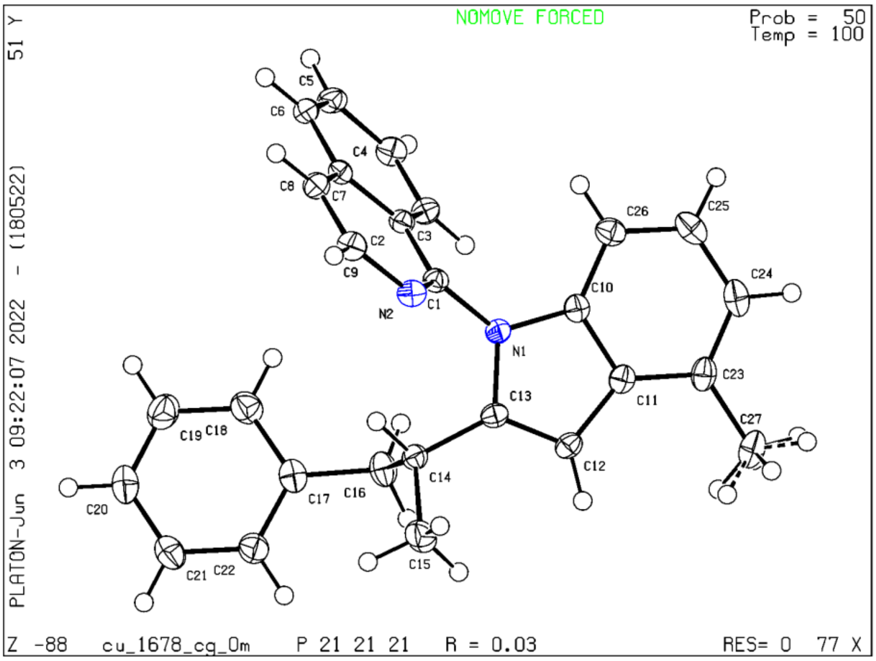

CCDC Number	CCDC 2176897
Empirical formula	$C_{27}H_{24}N_2$
Formula weight	376.48
Temperature/K	100.00
Wavelength/Å	1.54178
Crystal system	Orthorhombic
Space group	$P2_12_12_1$
a/Å	8.8293(7)
b/Å	10.9103(8)
c/Å	21.5469(14)
α/°	90
β/°	90
γ/°	90
Volume/Å^3	2075.6(3)
Z	4
ρ_{calc}/gcm^{-3}	1.205
μ/mm^{-1}	0.537
F(000)	800

Crystal size/mm^3	0.387×0.268×0.24
Crystal color	colorless
Crystal shape	block
Radiation	CuK_α (λ=1.54178 Å)
2Θ range for data collection/°	8.21 to 158.09 (0.79 Å)
Index ranges	-10 ≤ h ≤ 11, -13 ≤ k ≤ 13, -27 ≤ l ≤ 27
Reflections collected	63265
Independent reflections	4445<br>R(int) = 0.0265<br>R(sigma) = 0.0093
Data/restraints/parameters	4445/0/265
Goodness-of-fit on F^2	1.049
Final R indexes [I>=2σ (I)]	R_1 = 0.0273, wR_2 = 0.0701
Final R indexes [all data]	R_1 = 0.0274, wR_2 = 0.0702
Largest diff. peak/hole/eÅ^{-3}	0.14/-0.19
Flack X parameter	0.03(8)

Crystallized from a mixture of dichloromethane and hexane. The data for **3c** were collected from a shock-cooled single crystal at 100.00 K on a Bruker D8 VENTURE dual wavelength Mo/Cu four-circle diffractometer with a microfocus sealed X-ray tube using mirror optics as monochromator and a Bruker PHOTON III detector. The diffractometer was equipped with an Oxford Cryostream 800 low temperature device and used CuKα radiation (λ = 1.54178 Å). All data were integrated with SAINT and a numerical absorption correction using SADABS was applied. The structure were solved by dual methods using XT and refined by full-matrix least-squares methods against F2 by XL. All non-hydrogen atoms were refined with anisotropic displacement parameters. The hydrogen atoms were refined isotropically on calculated positions using a riding model with their Uiso values constrained to 1.5 times the Ueq of their pivot atoms for terminal sp3 carbon atoms and 1.2 times for all other carbon atoms. Crystallographic data for the structures reported in this paper have been deposited with the Cambridge Crystallographic Data Centre. CCDC 2176897 contain the supplementary crystallographic data for this paper. These data can be obtained free of charge from The Cambridge Crystallographic Data Centre via www.ccdc.cam.ac.uk/structures.

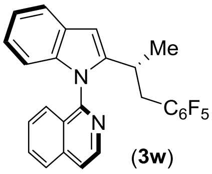

(**3w**)

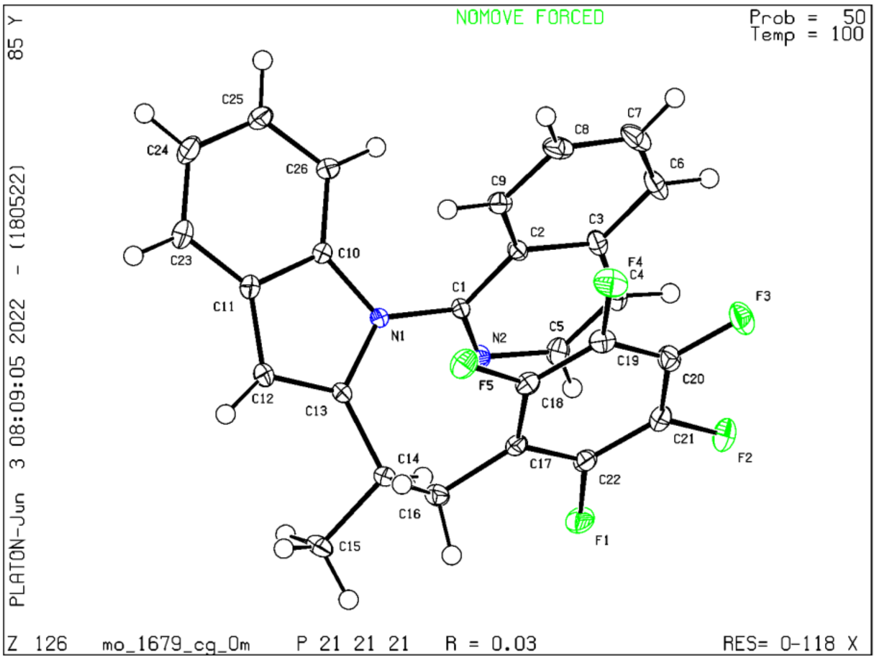

CCDC Number	CCDC 2176898
Empirical formula	$C_{26}H_{17}F_5N_2$
Formula weight	452.41
Temperature/K	100.00
Wavelength/Å	0.71073
Crystal system	Orthorhombic
Space group	$P2_12_12_1$
a/Å	8.5436(3)
b/Å	9.6113(3)
c/Å	24.6842(7)
α/°	90
β/°	90
γ/°	90
Volume/Å^3	2026.95(11)
Z	4
ρ_{calc}/gcm^{-3}	1.483
μ/mm^{-1}	0.120
F(000)	928
Crystal size/mm^3	0.856×0.204×0.11
Crystal color	colorless
Crystal shape	plank

Radiation	MoK_α (λ=0.71073 Å)
2Θ range for data collection/°	4.55 to 69.73 (0.62 Å)
Index ranges	$-13 \le h \le 13$, $-15 \le k \le 15$, $-39 \le l \le 38$
Reflections collected	119829
Independent reflections	8497<br>R(int) = 0.0201<br>R(sigma) = 0.0089
Data/restraints/parameters	8497/0/299
Goodness-of-fit on F^2	1.079
Final R indexes [I>=2σ (I)]	R_1 = 0.0259, wR_2 = 0.0743
Final R indexes [all data]	R_1 = 0.0273, wR_2 = 0.0754
Largest diff. peak/hole/eÅ^{-3}	0.41/-0.18
Flack X parameter	-0.04(4)

Crystallized from dichloromethane and hexane mixture. The data for **3w** were collected from a shock-cooled single crystal at 100.00 K on a Bruker D8 VENTURE dual wavelength Mo/Ag four-circle diffractometer with a microfocus sealed X-ray tube using mirror optics as monochromator and a Bruker PHOTON III detector. The diffractometer was equipped with an Oxford Cryostream 800 low temperature device and used MoK_α radiation (λ = 0.71073 Å). All data were integrated with SAINT and a numerical absorption correction using SADABS was applied. The structure were solved by dual methods using XT and refined by full-matrix least-squares methods against F^2 by XL. All non-hydrogen atoms were refined with anisotropic displacement parameters. The hydrogen atoms were refined isotropically on calculated positions using a riding model with their U_{iso} values constrained to 1.5 times the U_{eq} of their pivot atoms for terminal sp^3 carbon atoms and 1.2 times for all other carbon atoms. Crystallographic data for the structures reported in this paper have been deposited with the Cambridge Crystallographic Data Centre. CCDC 2176898 contain the supplementary crystallographic data for this paper. These data can be obtained free of charge from The Cambridge Crystallographic Data Centre via www.ccdc.cam.ac.uk/structures.

17. NMR spectra

Supplementary Fig. 13. ^{1}H NMR spectrum of **CCA-3**

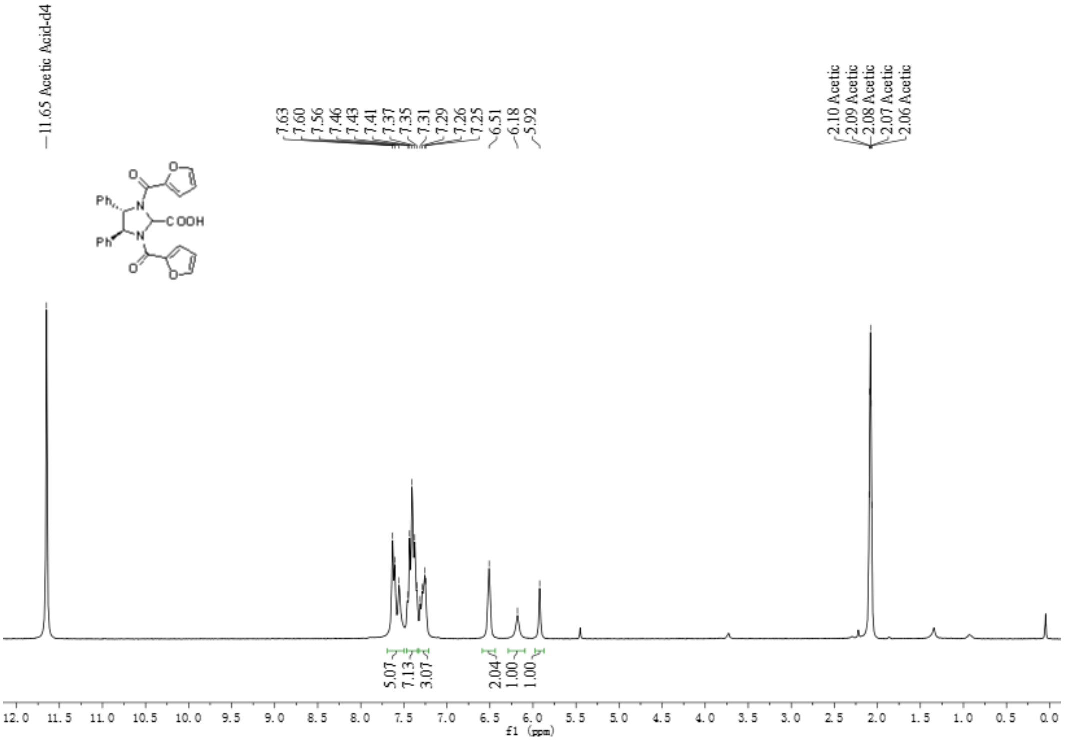

Supplementary Fig. 14. ^{13}C NMR spectrum of **CCA-3**

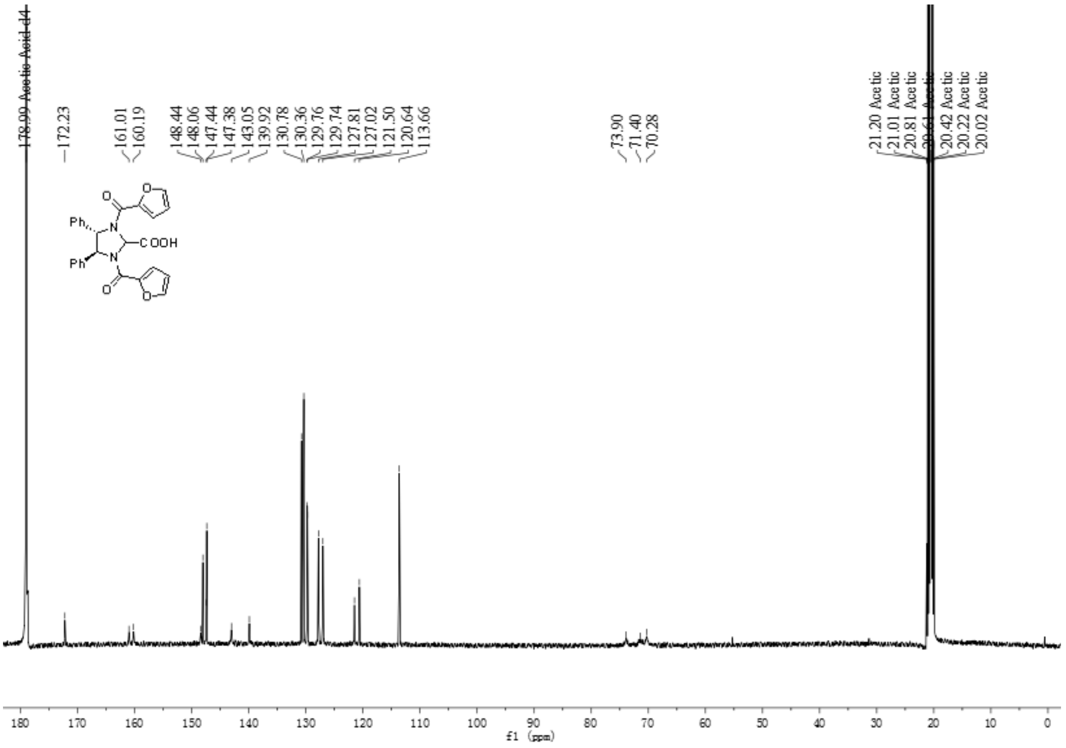

Supplementary Fig. 15. ^{1}H NMR spectrum of **CCA-4**

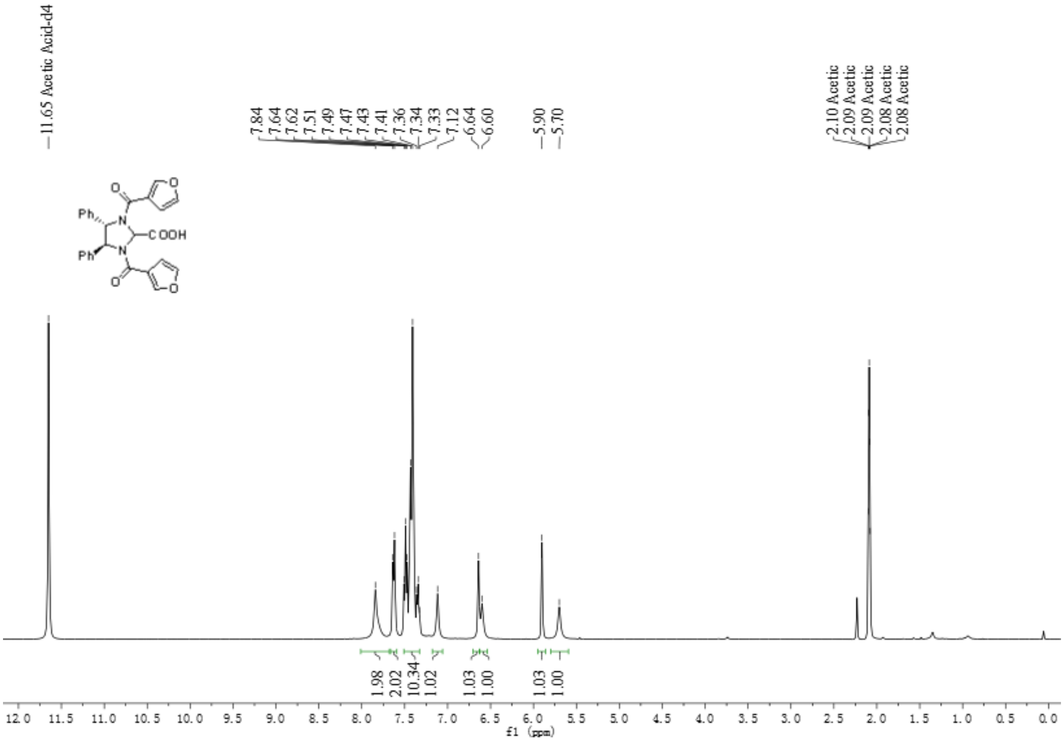

Supplementary Fig. 16. ^{13}C NMR spectrum of **CCA-4**

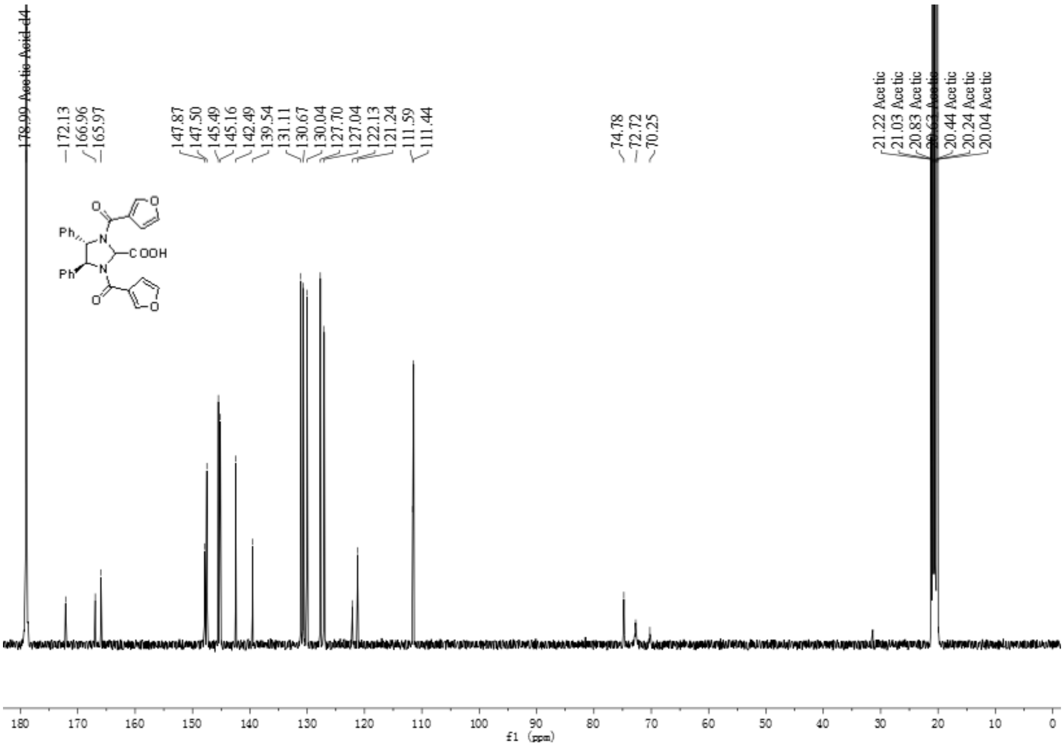

Supplementary Fig. 17. ^{1}H NMR spectrum of **CCA-5**

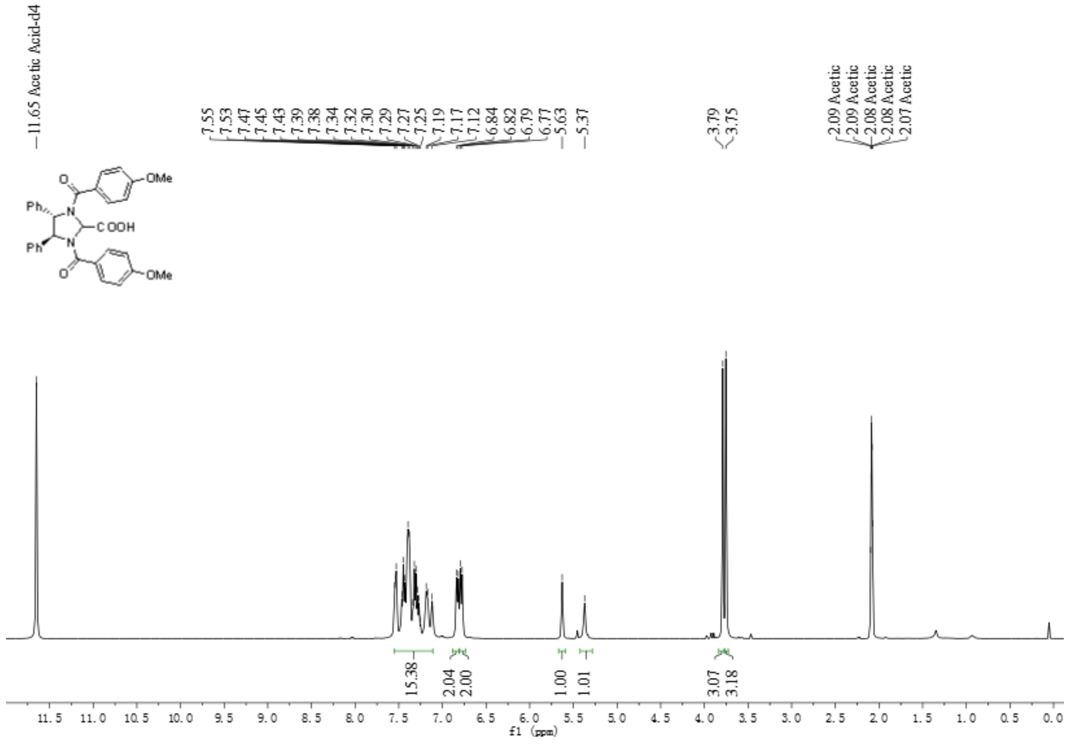

Supplementary Fig. 18. ^{13}C NMR spectrum of **CCA-5**

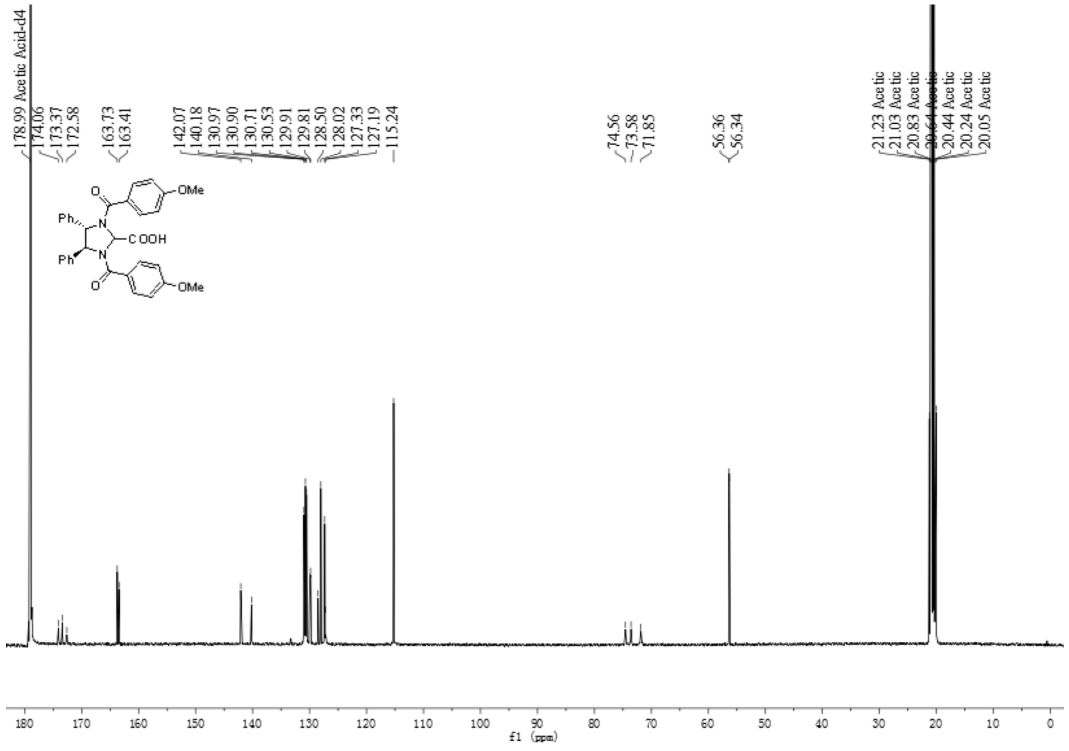

Supplementary Fig. 19. ^{1}H NMR spectrum of **CCA-7**

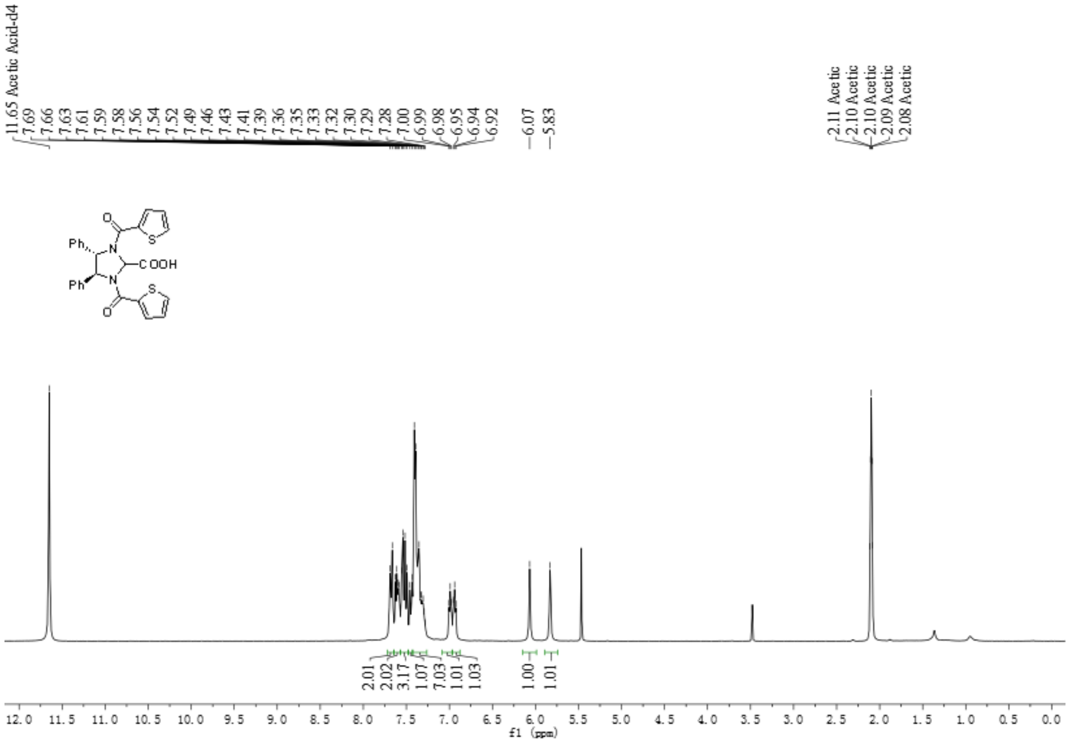

Supplementary Fig. 20. ^{13}C NMR spectrum of **CCA-7**

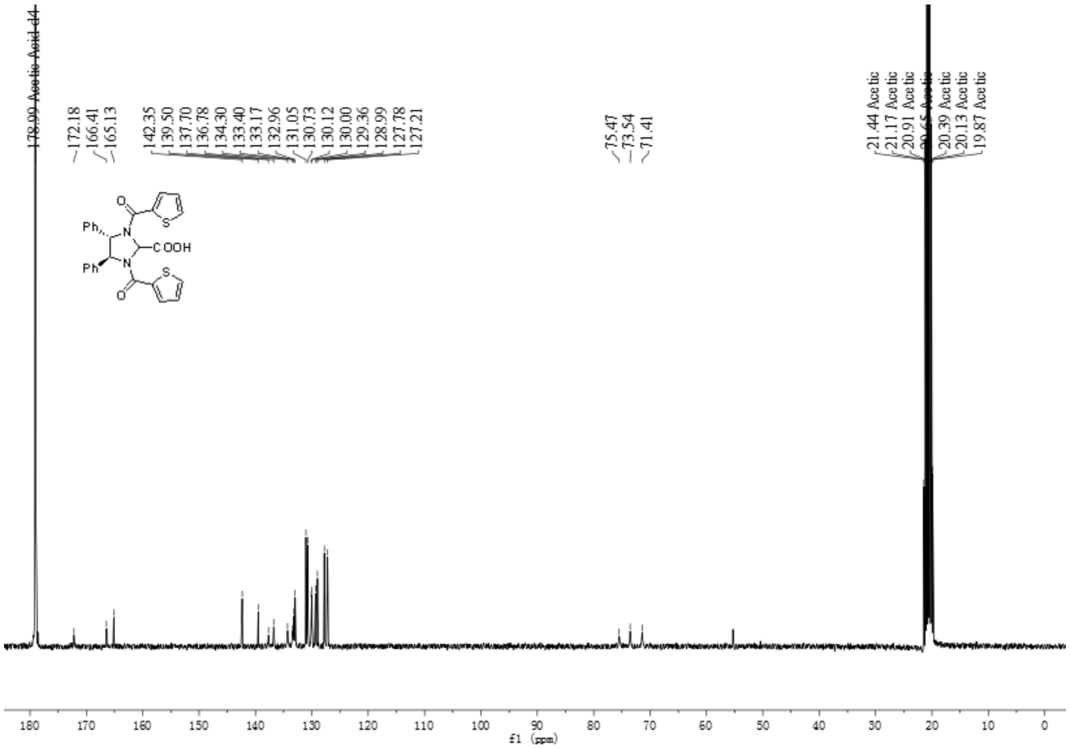

Supplementary Fig. 21. ^{1}H NMR spectrum of **CCA-8**

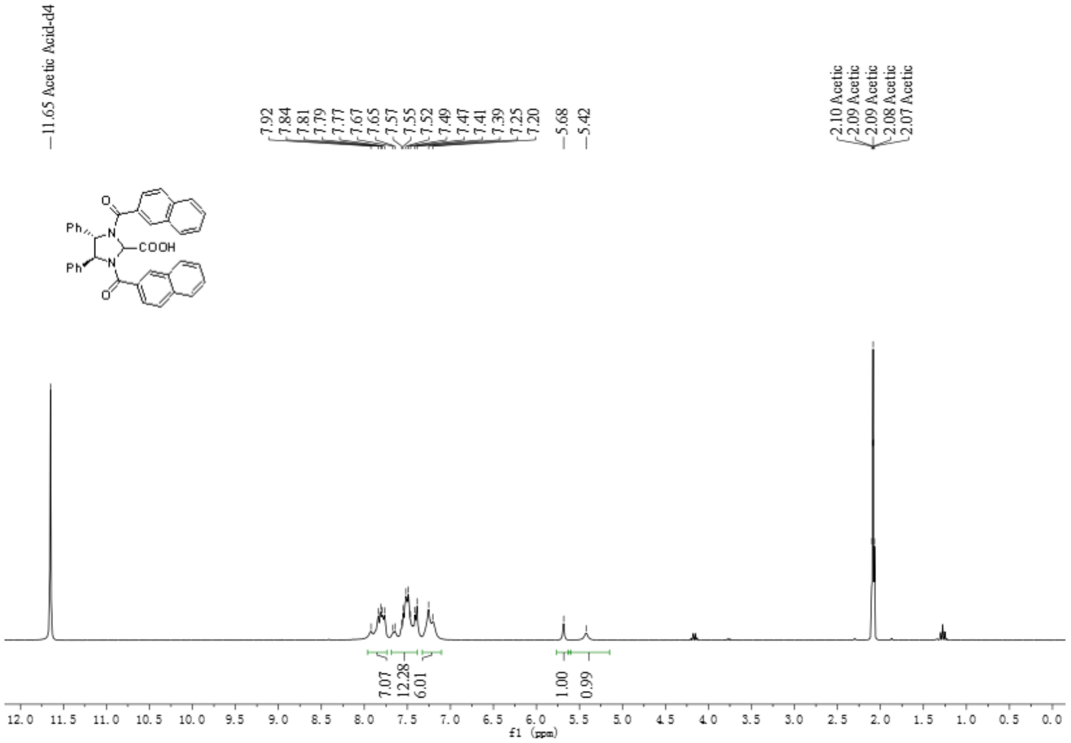

Supplementary Fig. 22. ^{13}C NMR spectrum of **CCA-8**

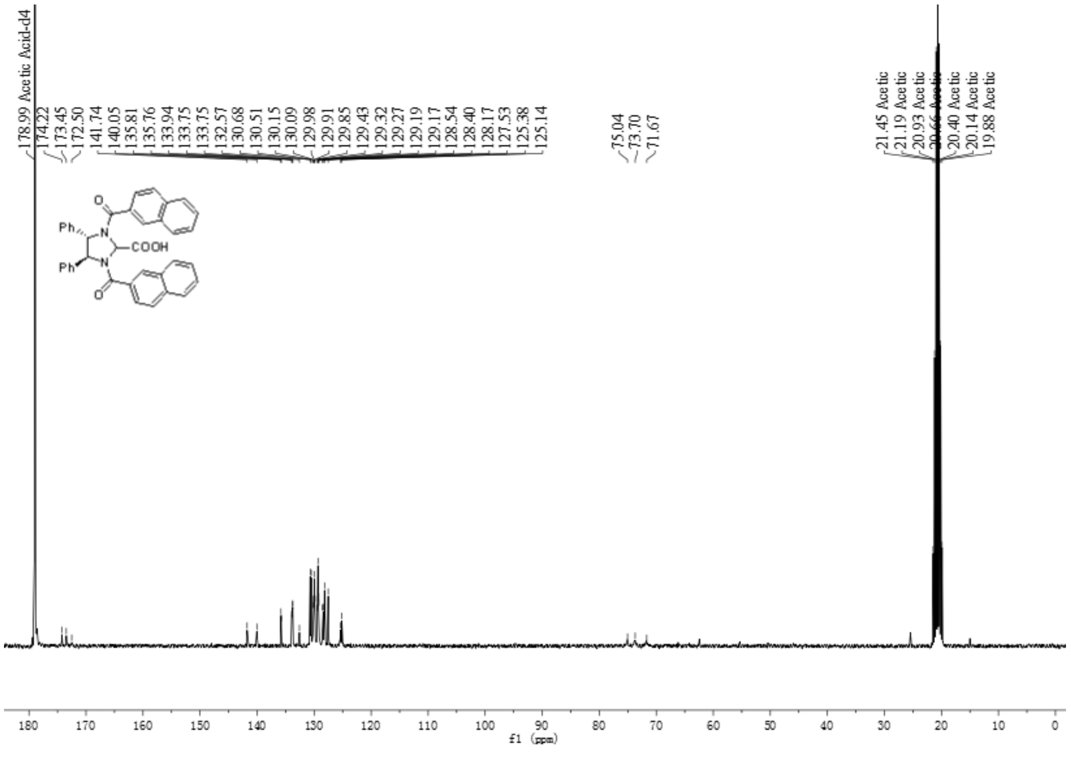

Supplementary Fig. 23. ^{1}H NMR spectrum of **CCA-11**

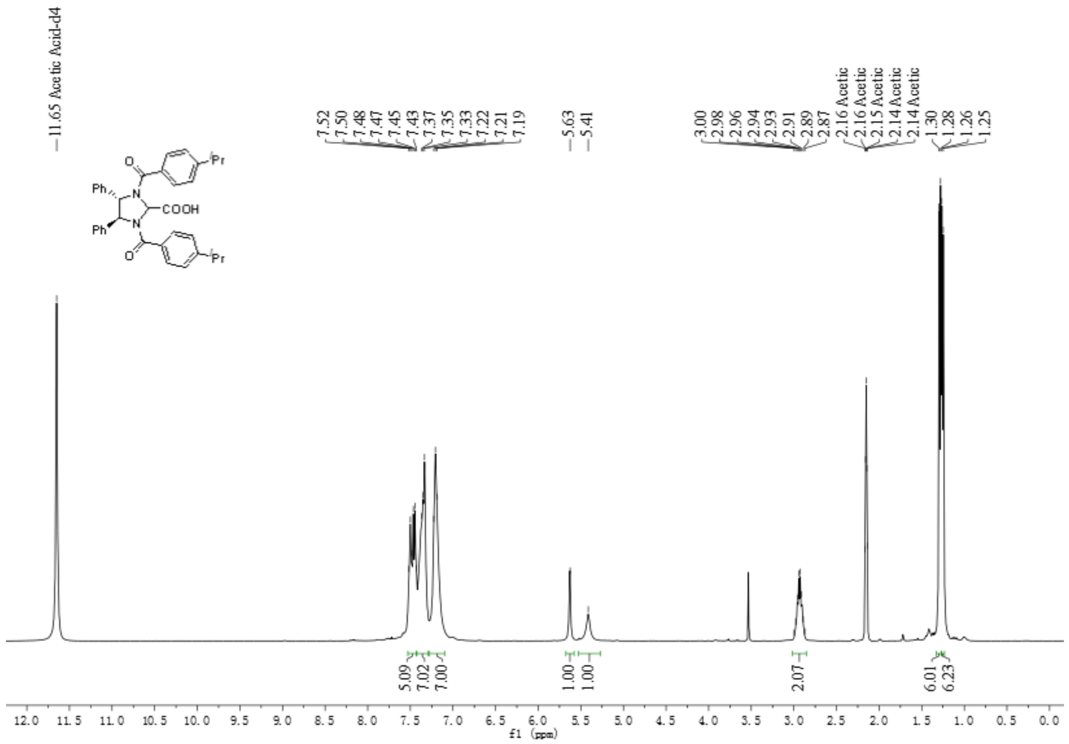

Supplementary Fig. 24. ^{13}C NMR spectrum of **CCA-11**

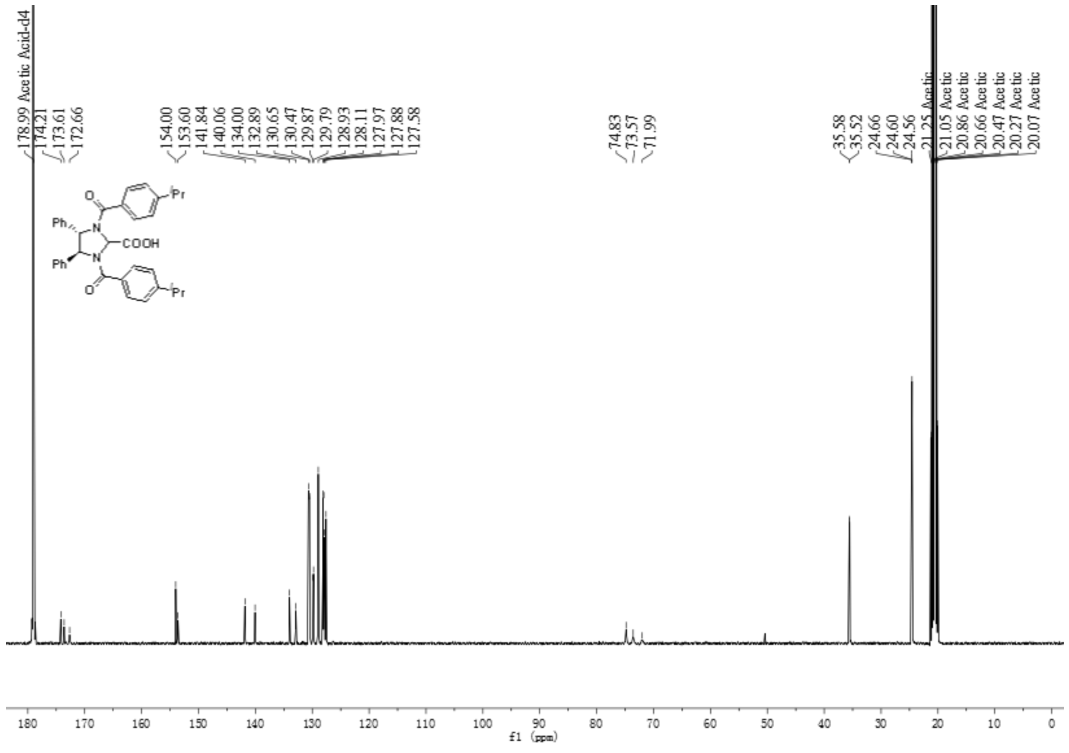

Supplementary Fig. 25. ^{1}H NMR spectrum of **CCA-12**

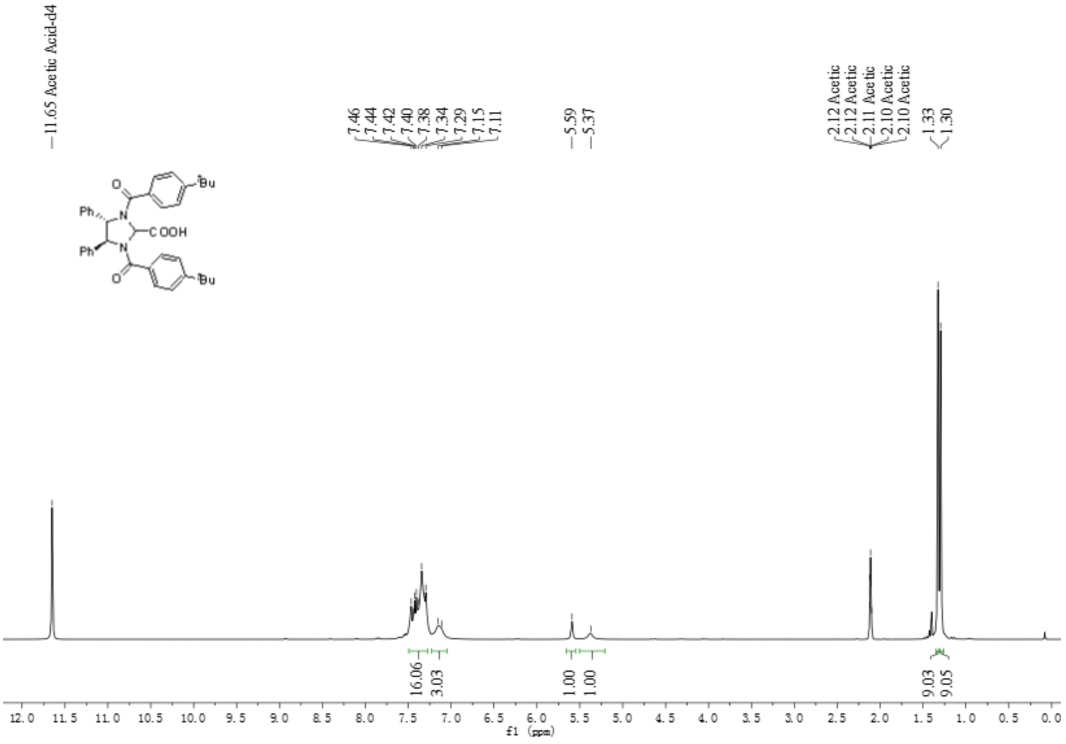

Supplementary Fig. 26. ^{13}C NMR spectrum of **CCA-12**

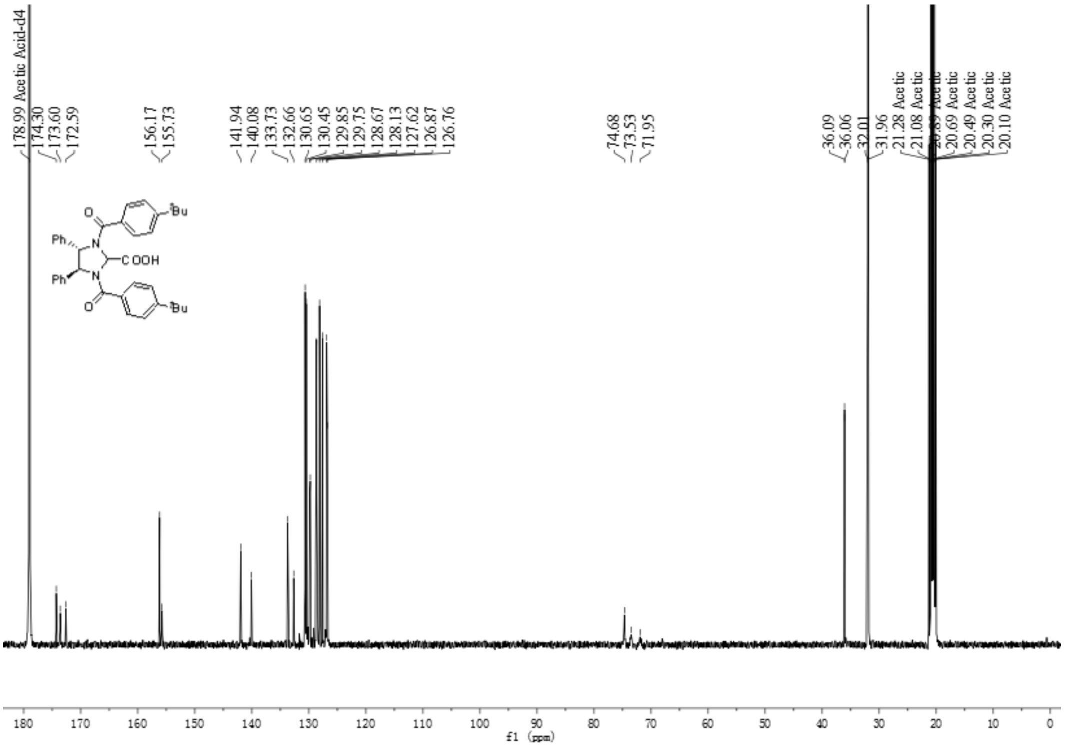

Supplementary Fig. 27. ^{1}H NMR spectrum of **CCA-16**

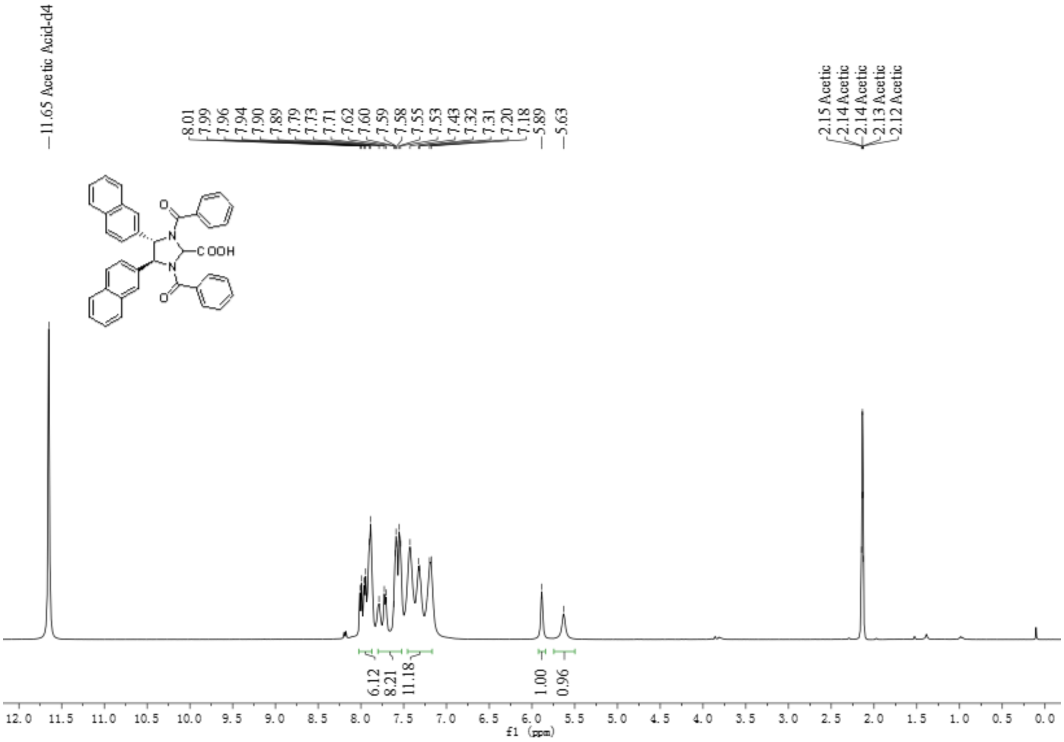

Supplementary Fig. 28. ^{13}C NMR spectrum of **CCA-16**

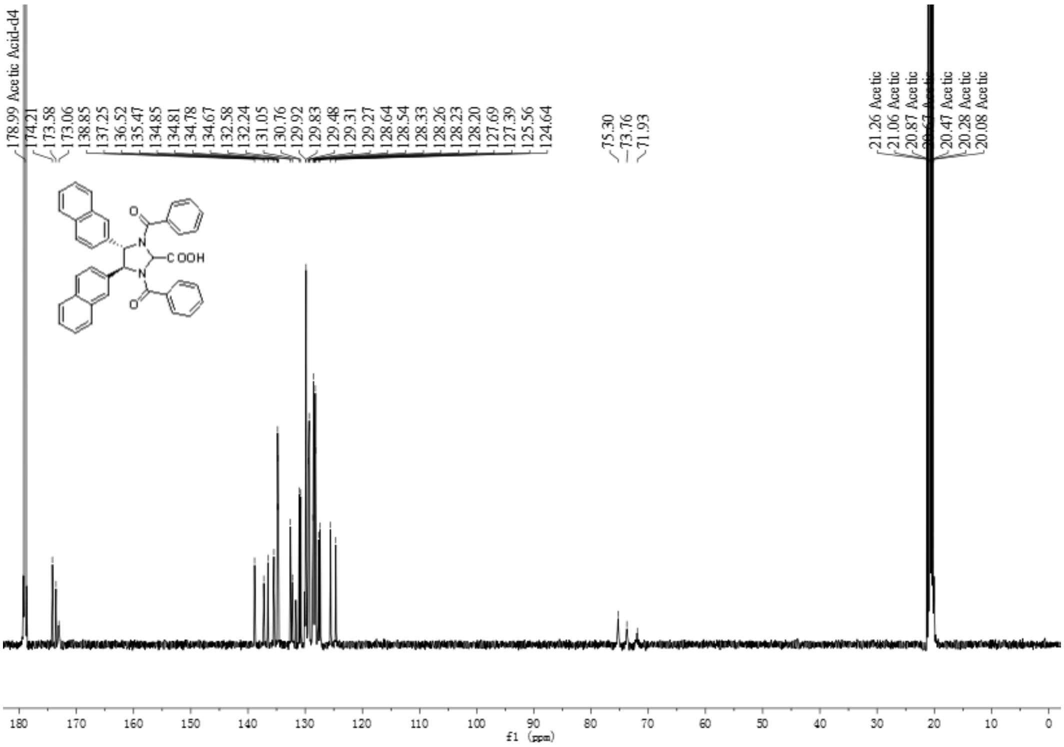

Supplementary Fig. 29. ^{1}H NMR spectrum of **CCA-17**

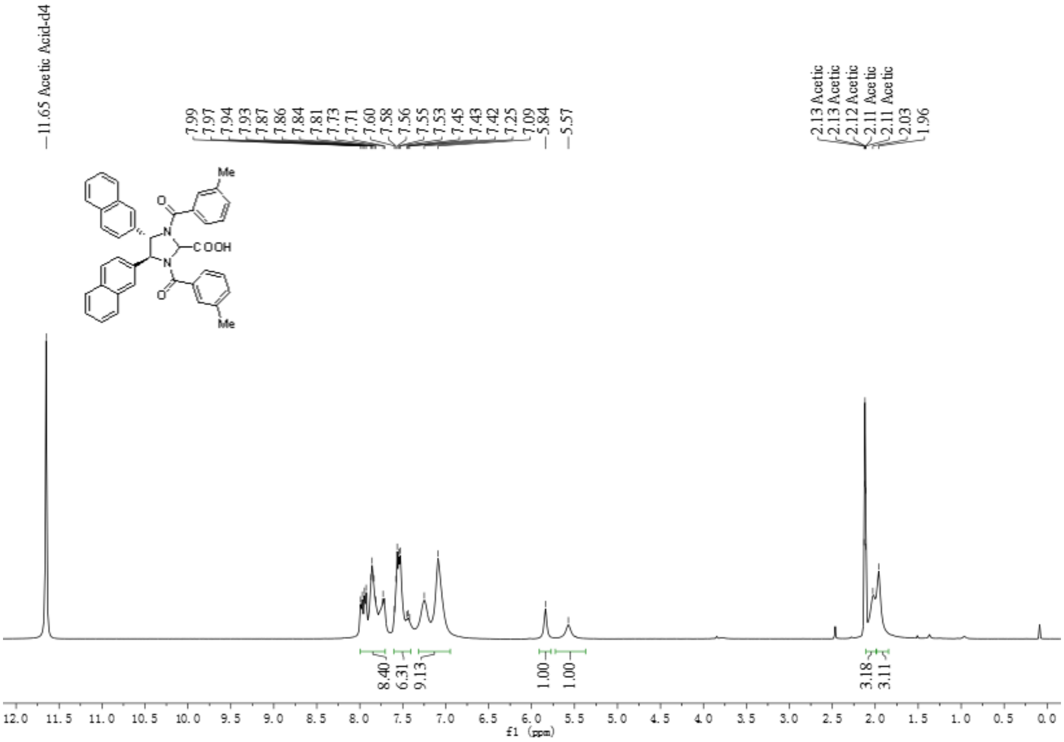

Supplementary Fig. 30. ^{13}C NMR spectrum of **CCA-17**

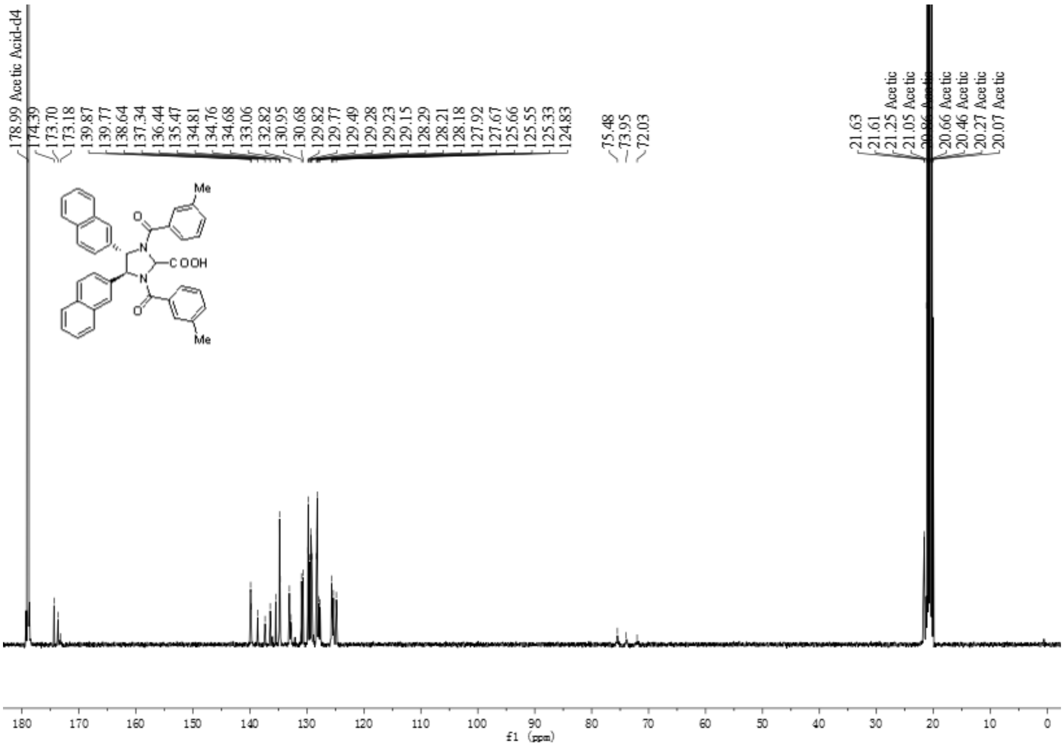

Supplementary Fig. 31. ^{1}H NMR spectrum of **CCA-19**

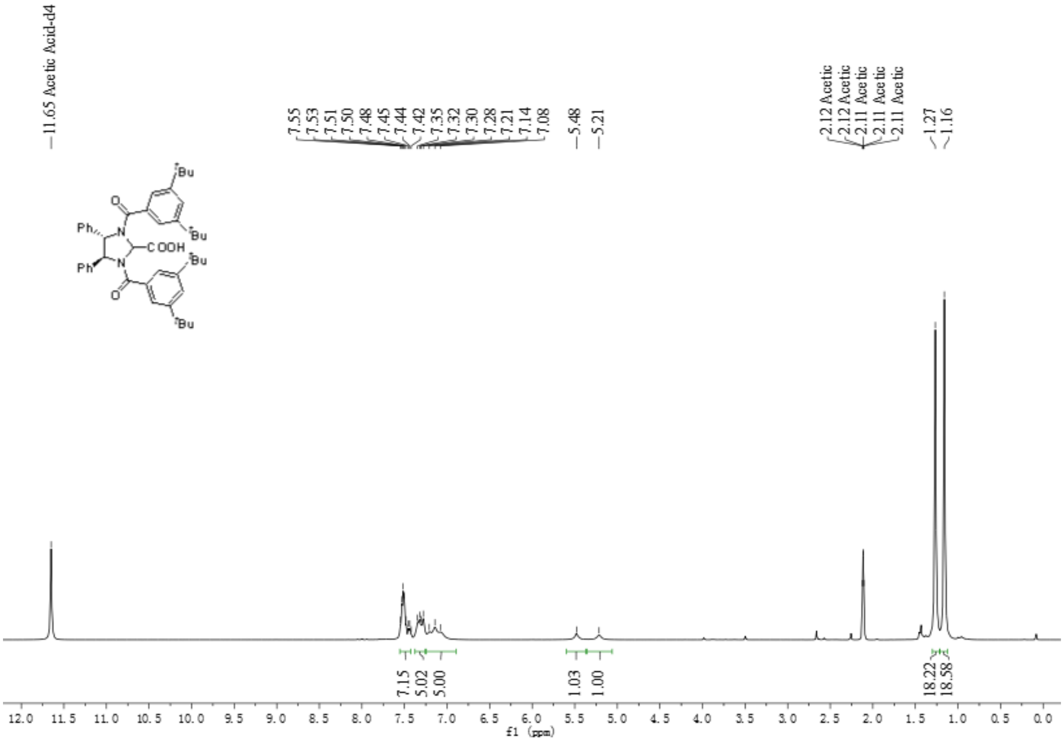

Supplementary Fig. 32. ^{13}C NMR spectrum of **CCA-19**

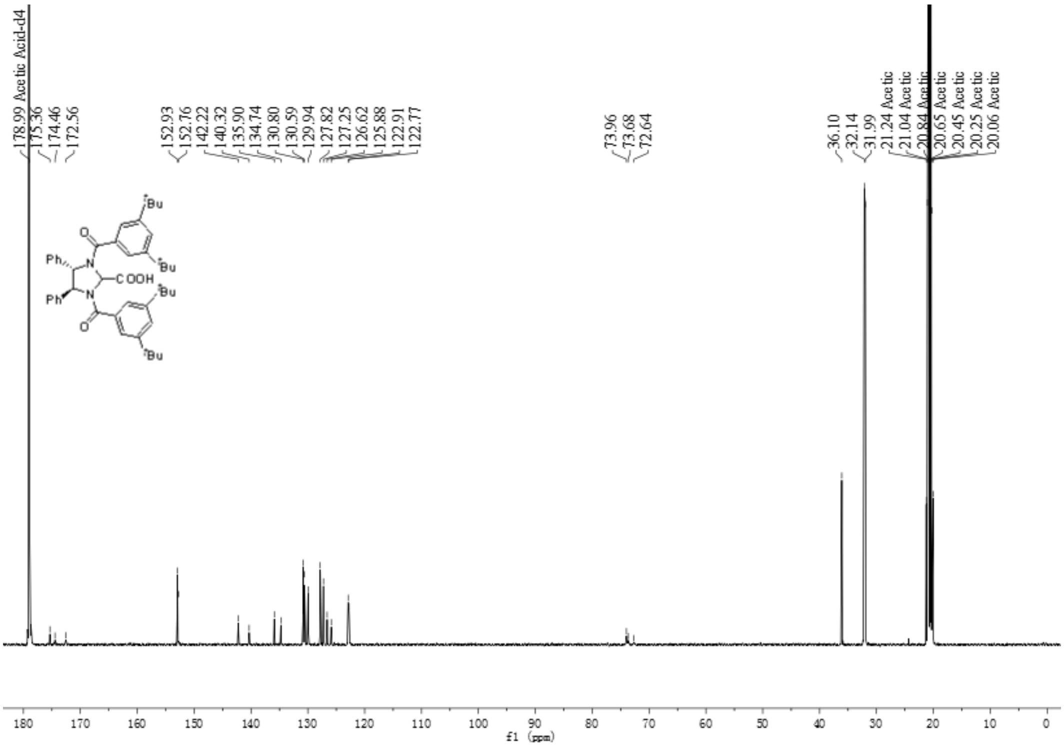

Supplementary Fig. 33. ^{1}H NMR spectrum of **CCA-20**

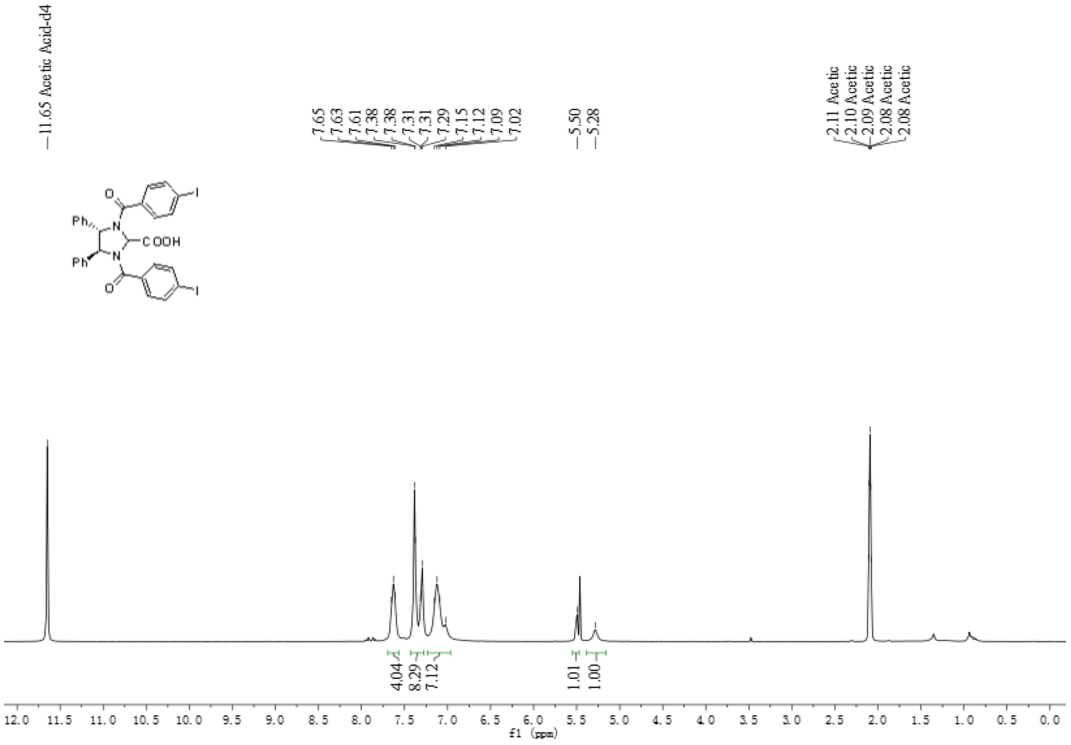

Supplementary Fig. 34. ^{13}C NMR spectrum of **CCA-20**

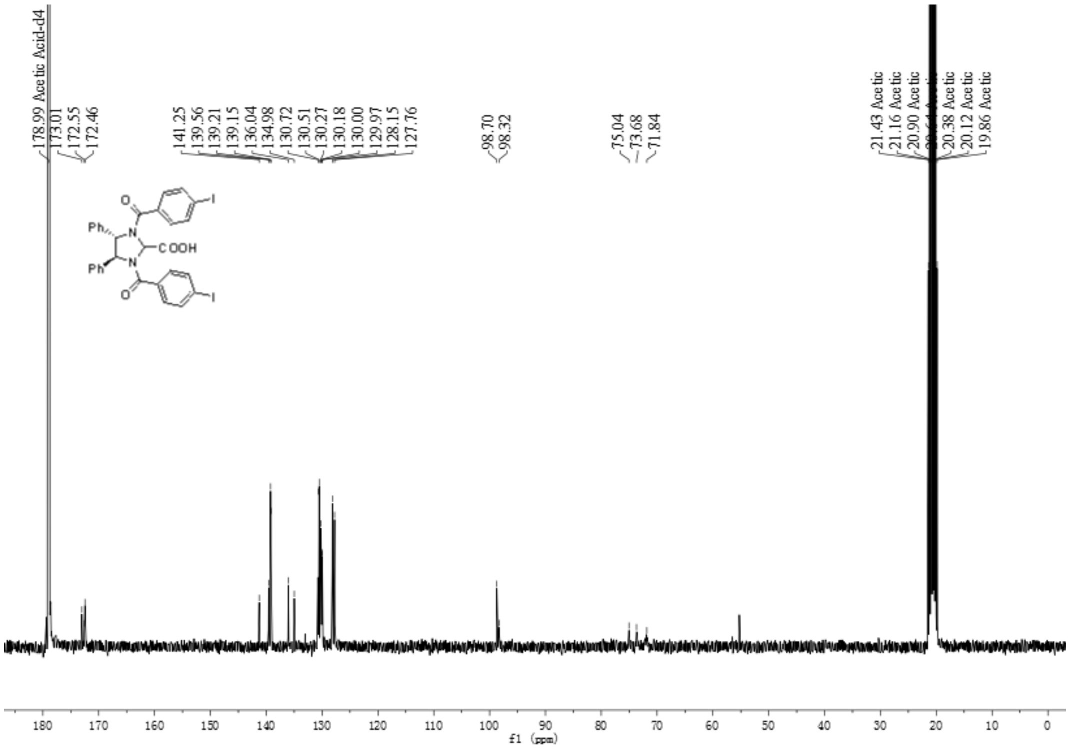

Supplementary Fig. 35. ^{1}H NMR spectrum of **CCA-21**

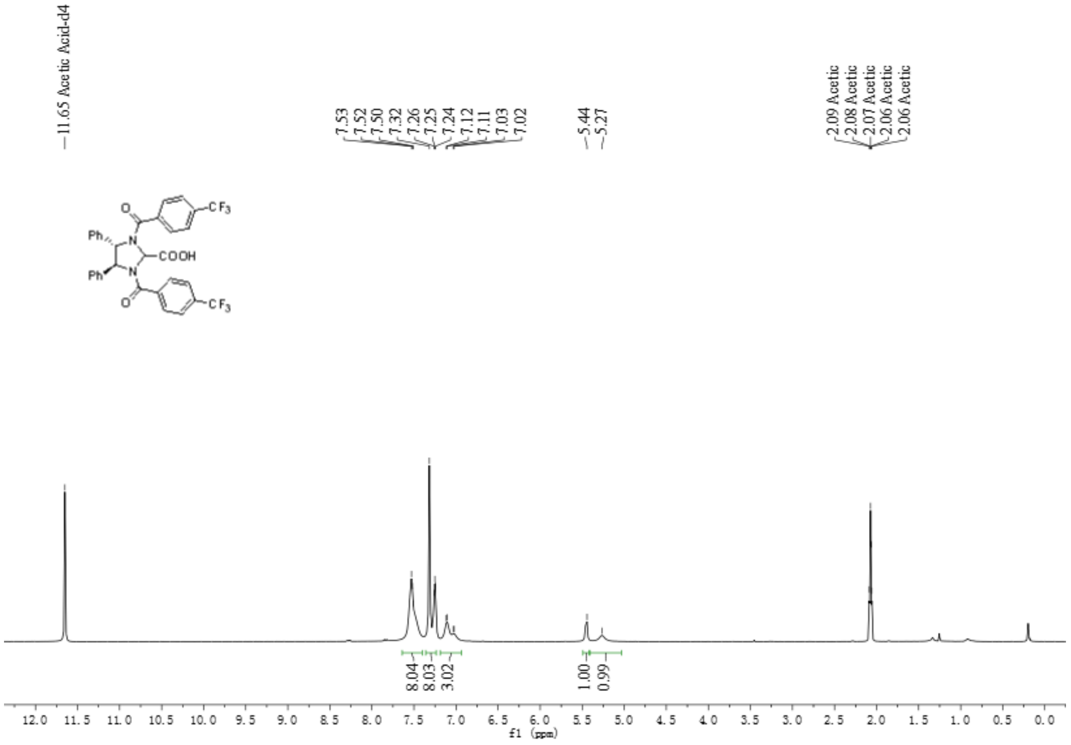

Supplementary Fig. 36. ^{13}C NMR spectrum of **CCA-21**

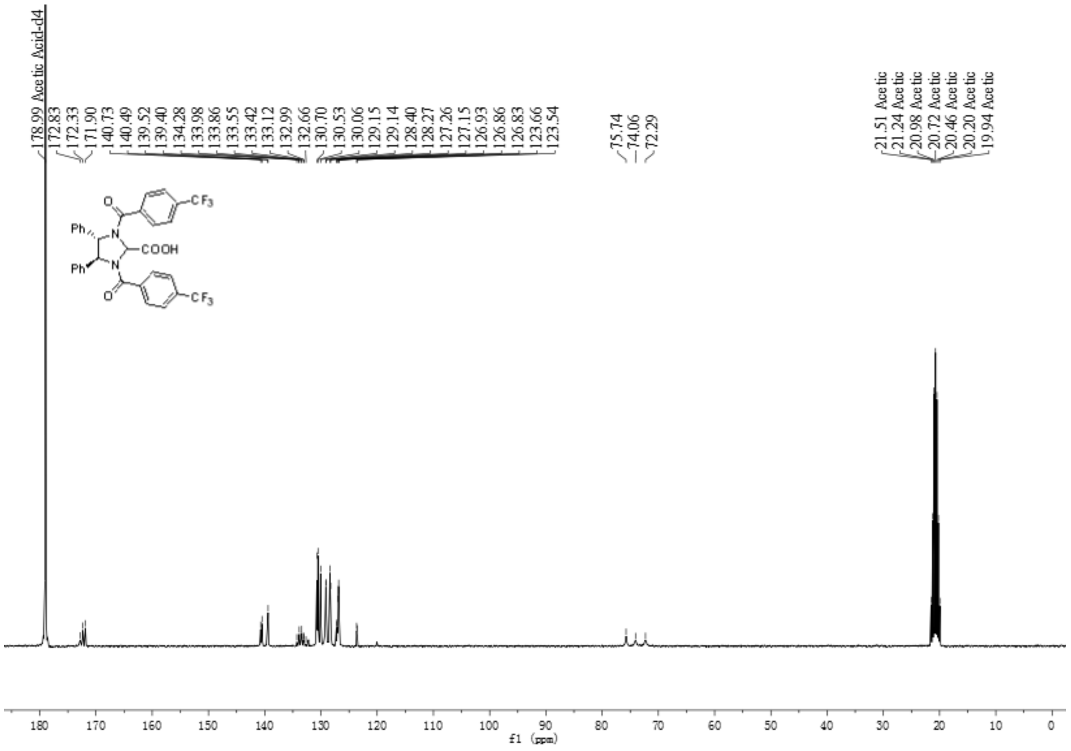

Supplementary Fig. 37. ^{19}F NMR spectrum of **CCA-21**

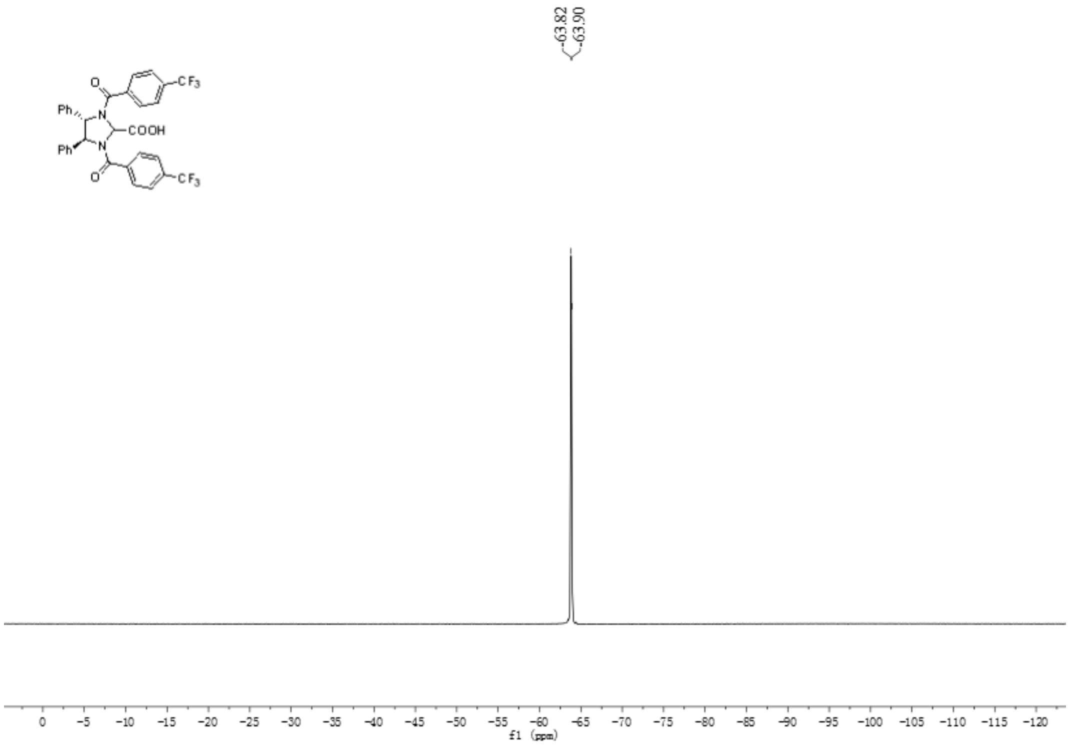

Supplementary Fig. 38. ^{1}H NMR spectrum of **1a**

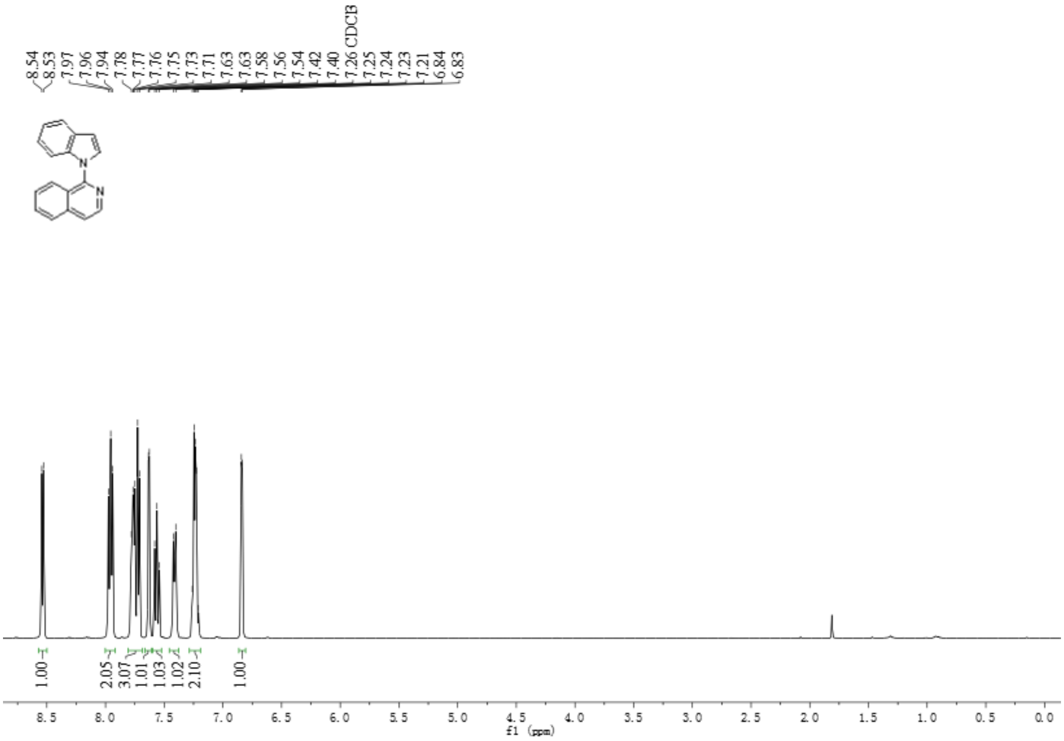

Supplementary Fig. 39. ^{13}C NMR spectrum of **1a**

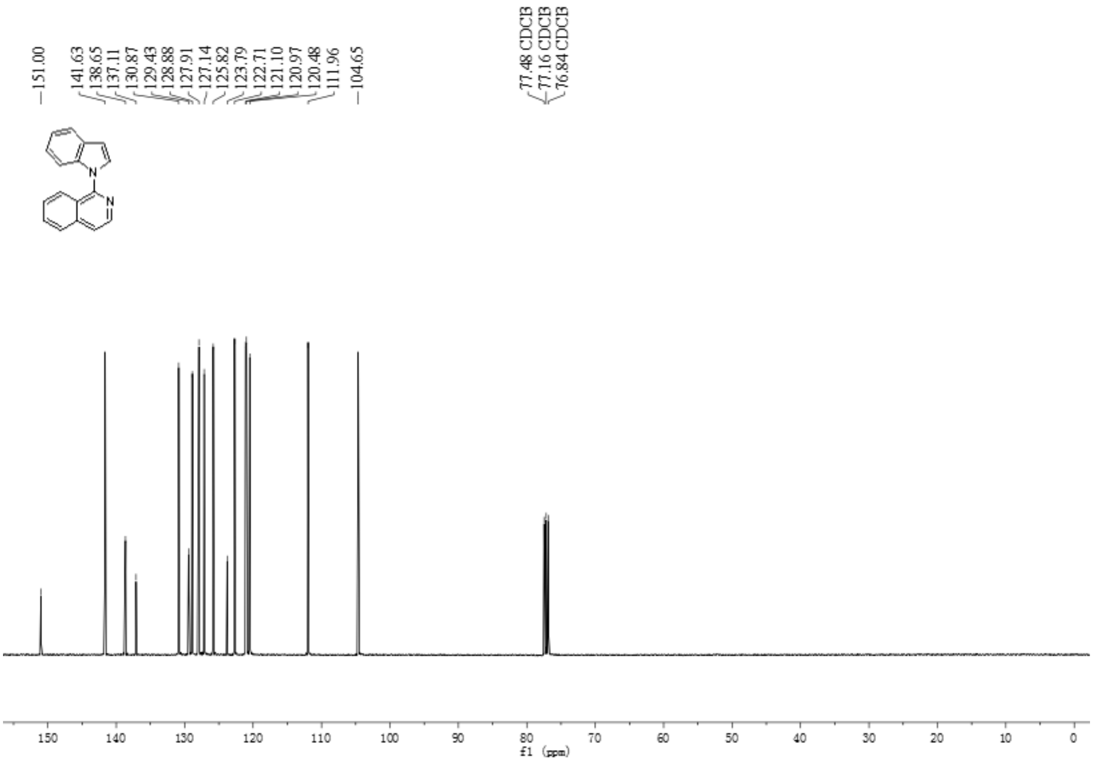

Supplementary Fig. 40. ^{1}H NMR spectrum of **1b**

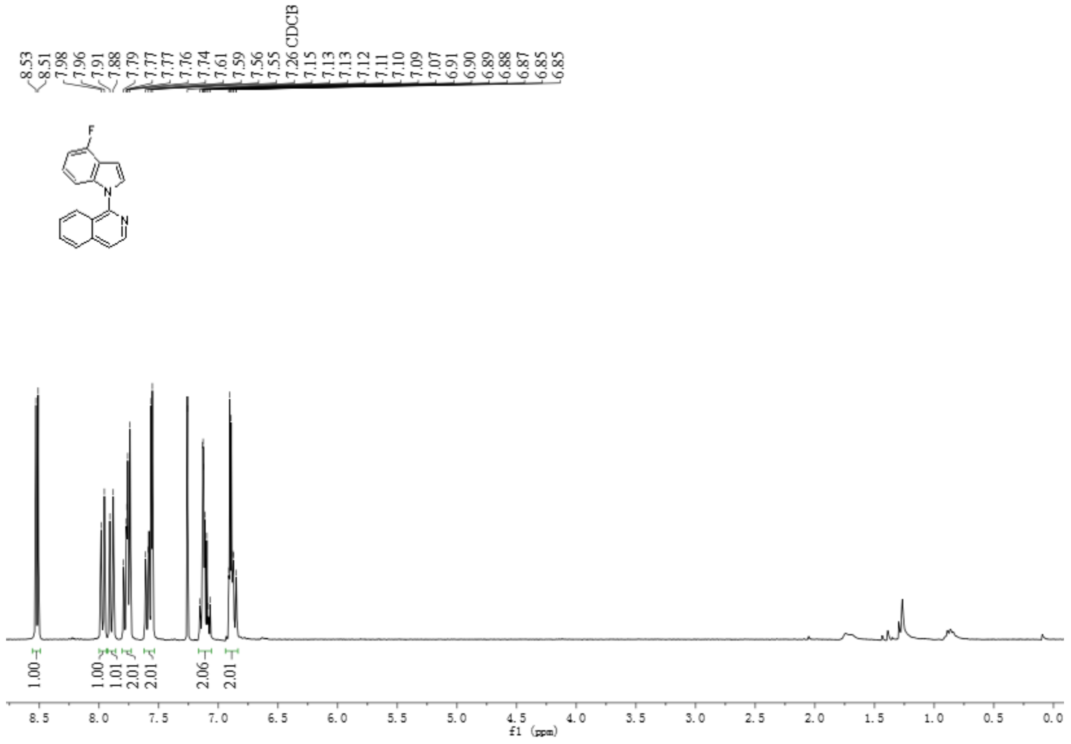

Supplementary Fig. 41. ^{13}C NMR spectrum of **1b**

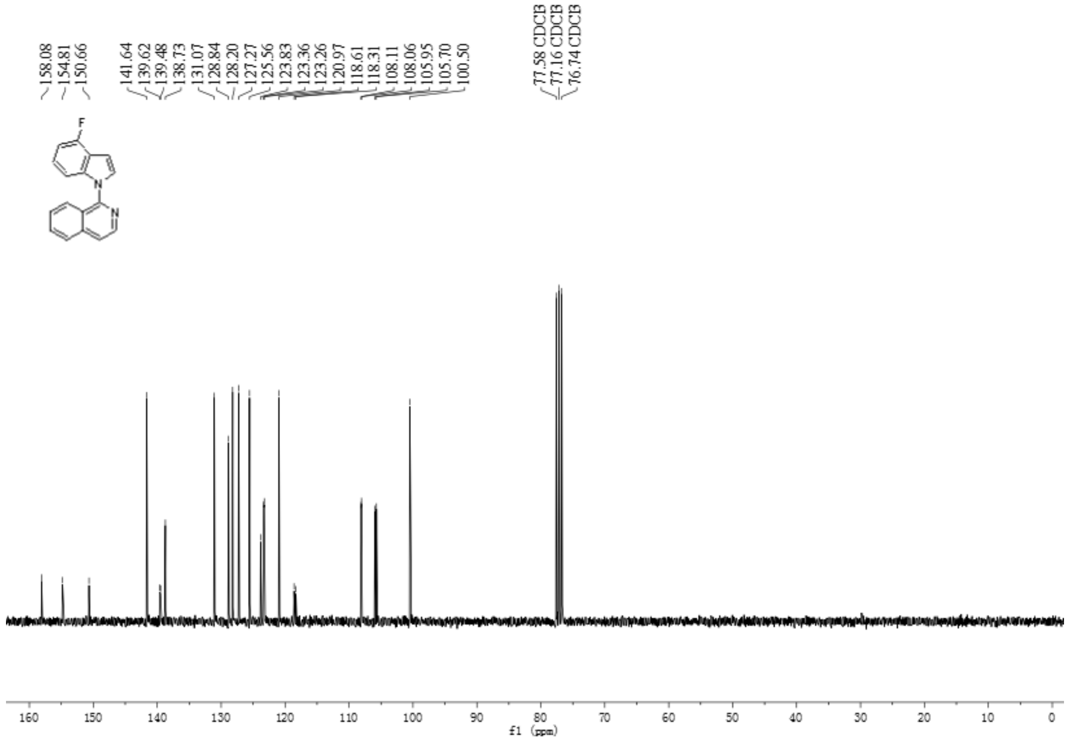

Supplementary Fig. 42. ^{19}F NMR spectrum of **1b**

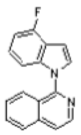

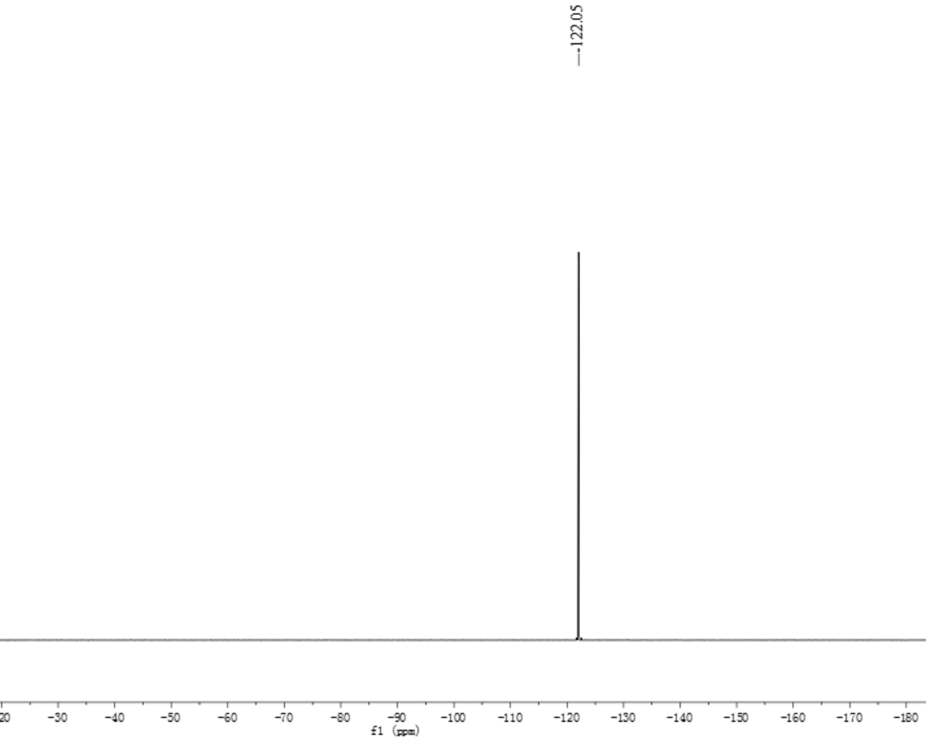

Supplementary Fig. 43. ^{1}H NMR spectrum of **1c**

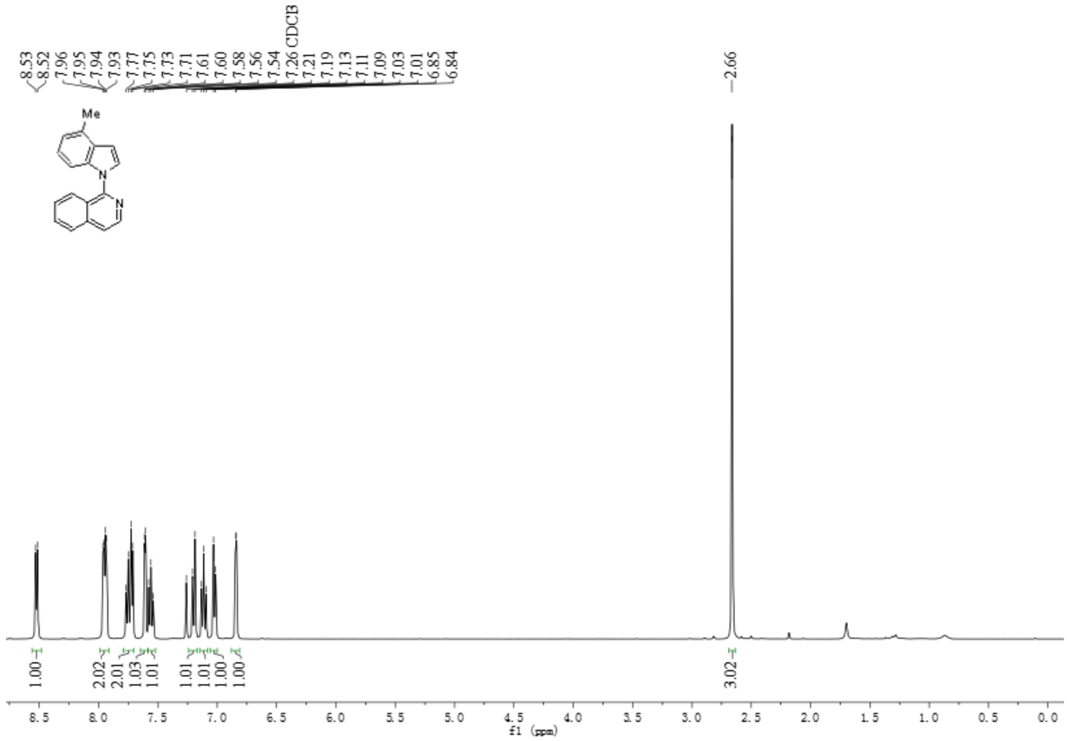

Supplementary Fig. 44. ^{13}C NMR spectrum of **1c**

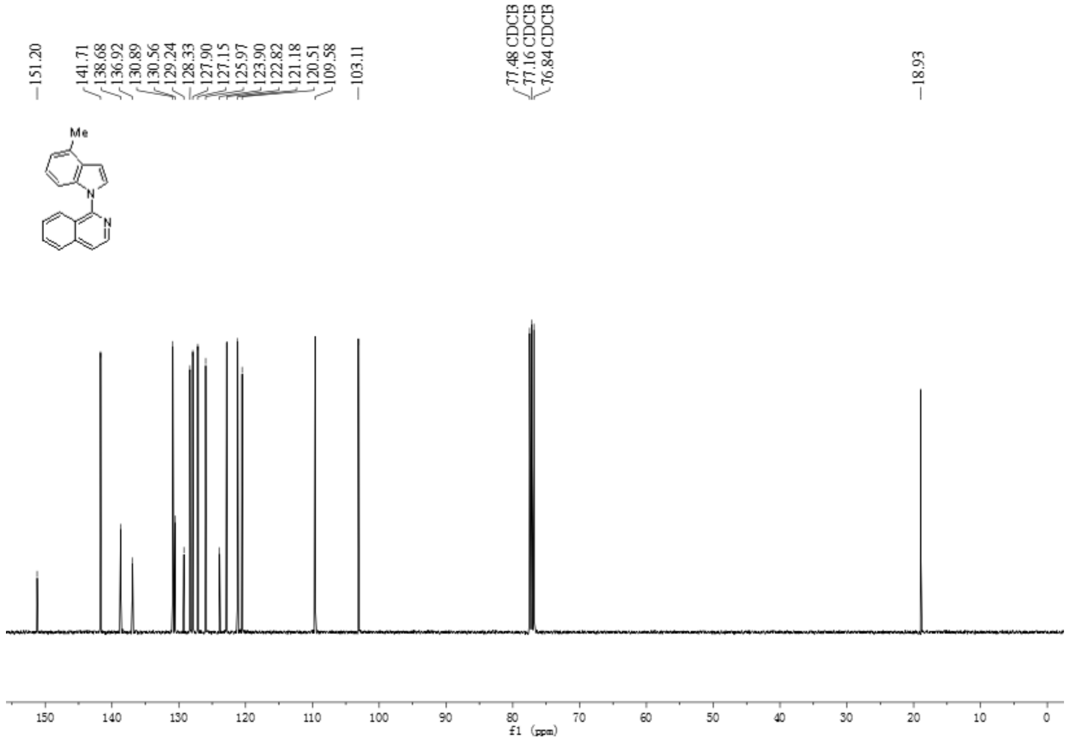

Supplementary Fig. 45. ^{1}H NMR spectrum of **1d**

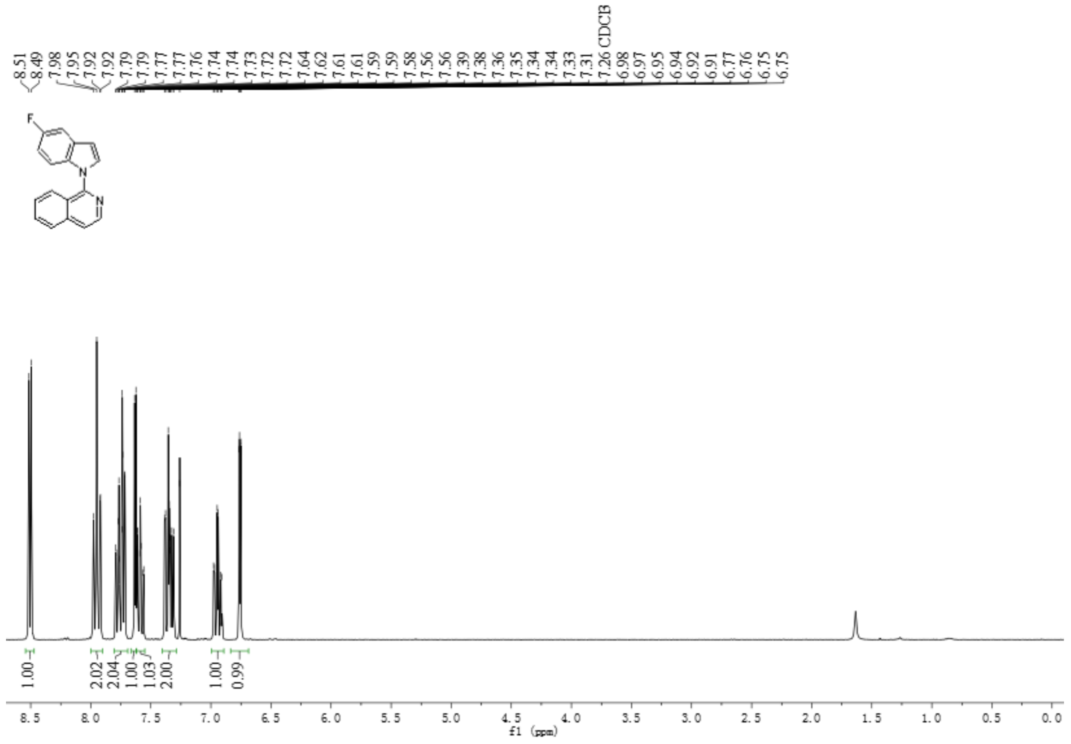

Supplementary Fig. 46. ^{13}C NMR spectrum of **1d**

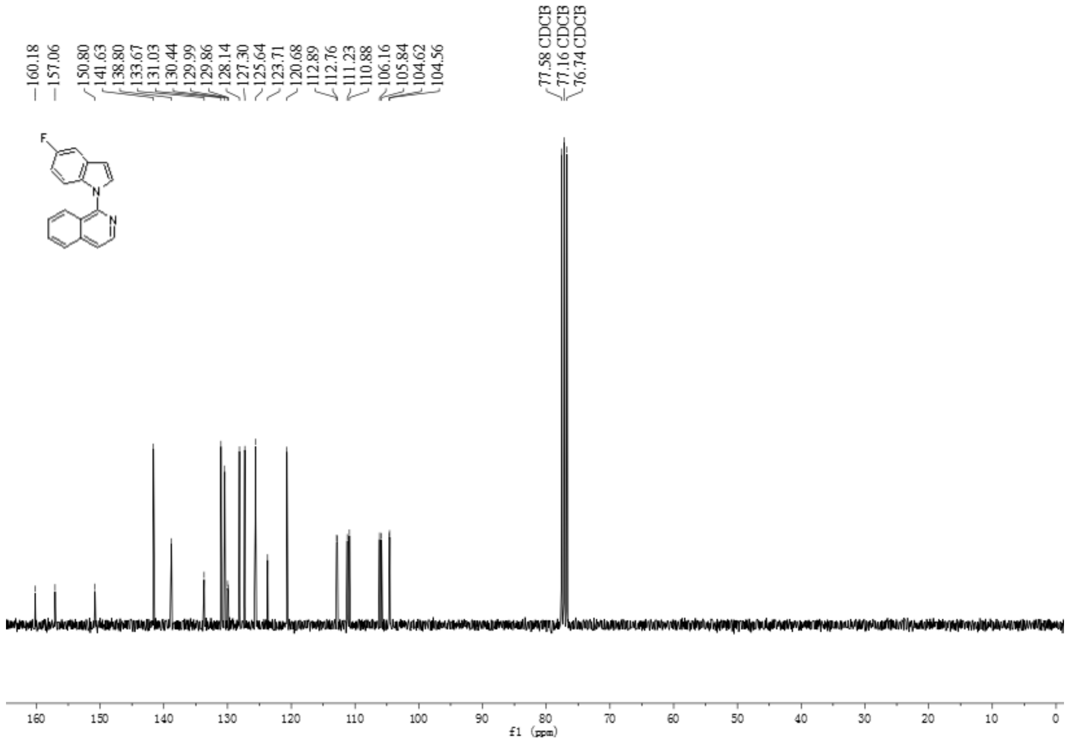

Supplementary Fig. 47. ^{19}F NMR spectrum of **1d**

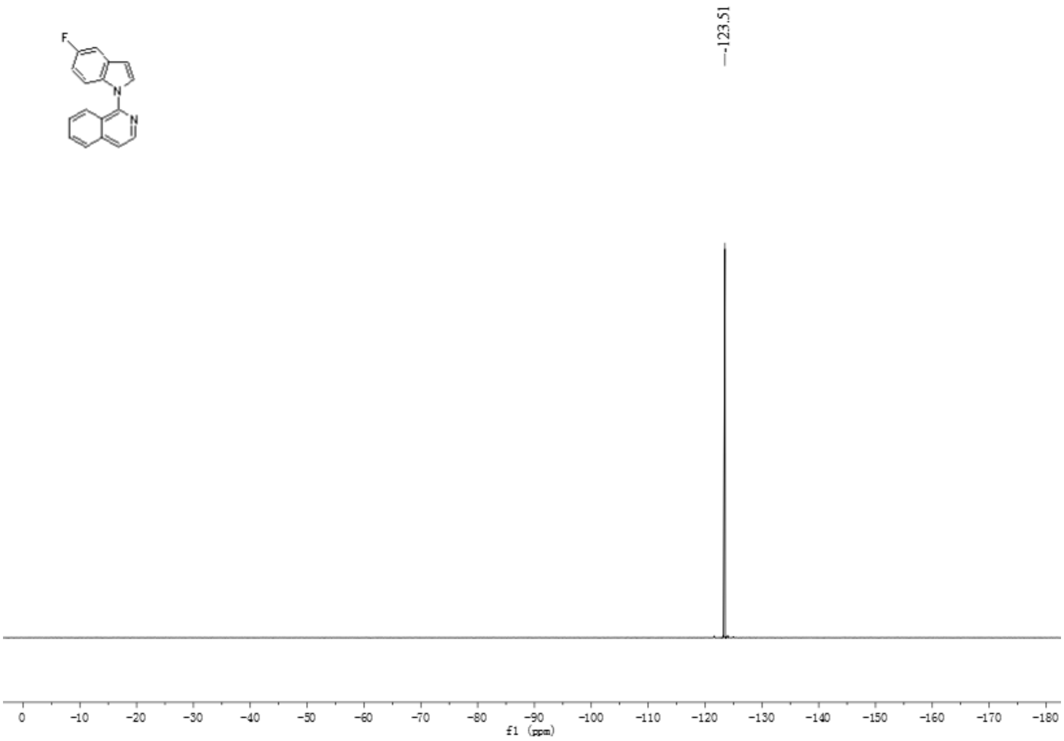

Supplementary Fig. 48. ^{1}H NMR spectrum of **1e**

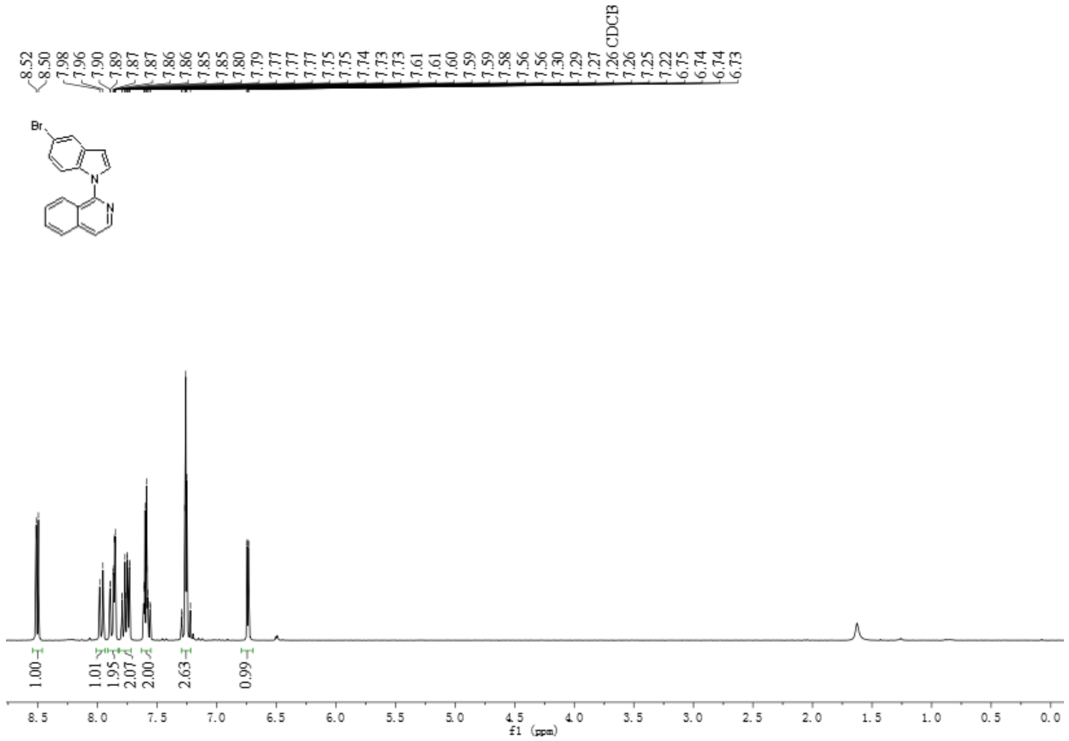

Supplementary Fig. 49. ^{13}C NMR spectrum of **1e**

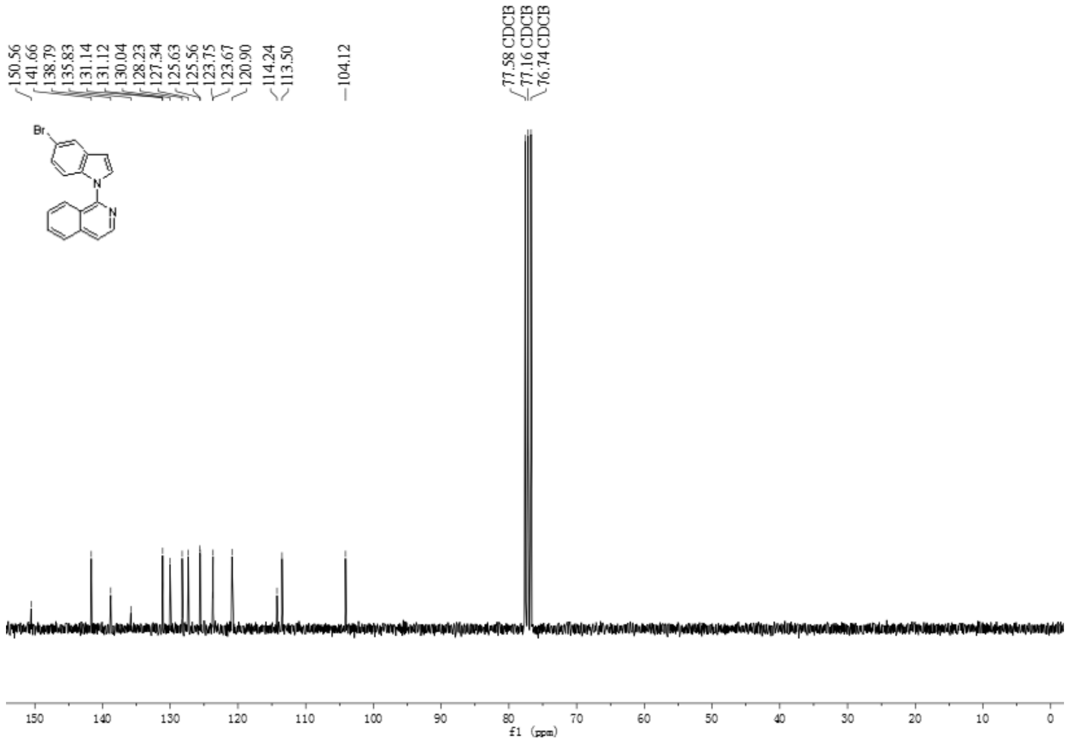

Supplementary Fig. 50. ^{1}H NMR spectrum of **1f**

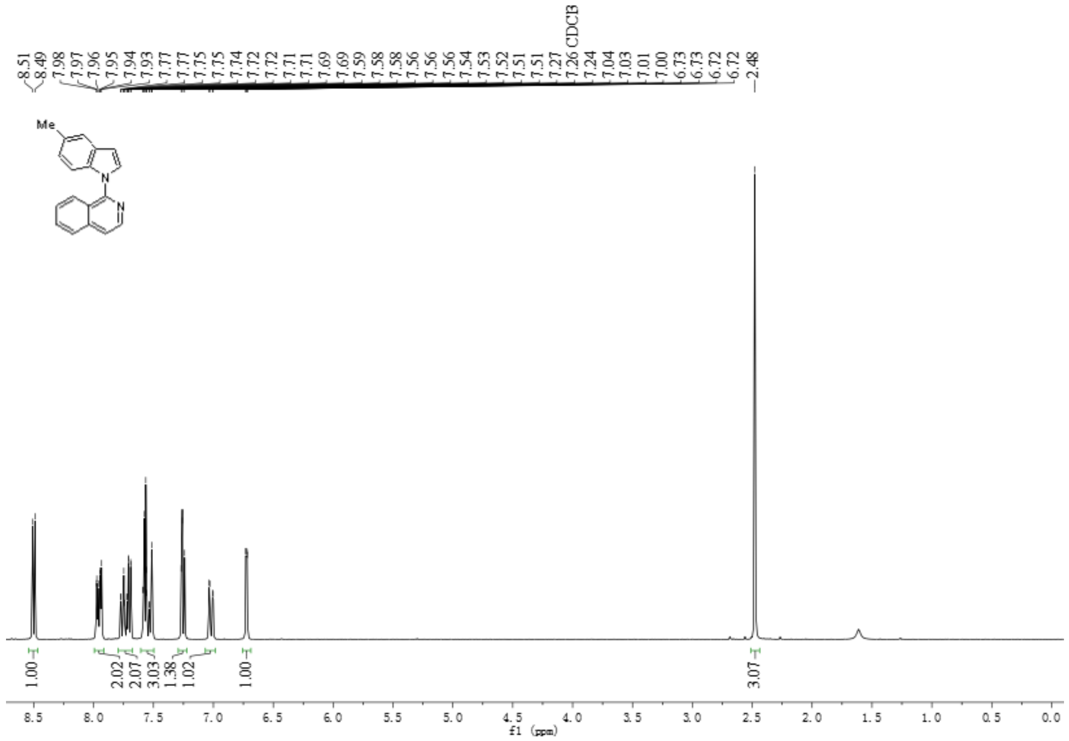

Supplementary Fig. 51. ^{13}C NMR spectrum of **1f**

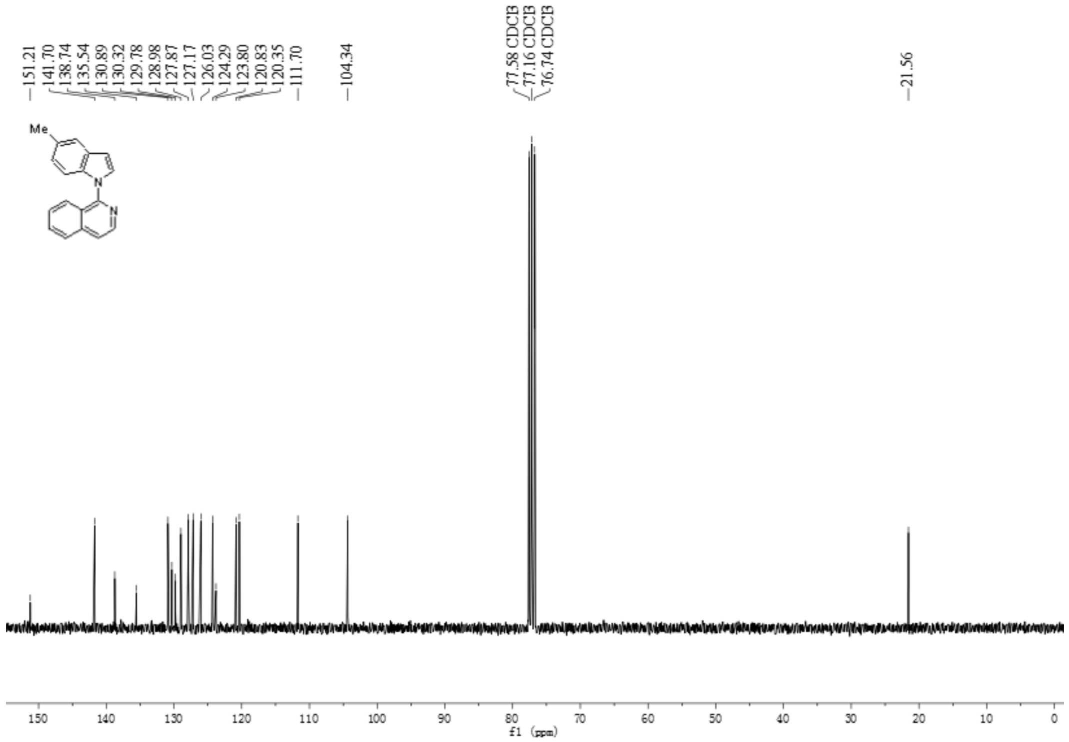

Supplementary Fig. 52. ^{1}H NMR spectrum of **1g**

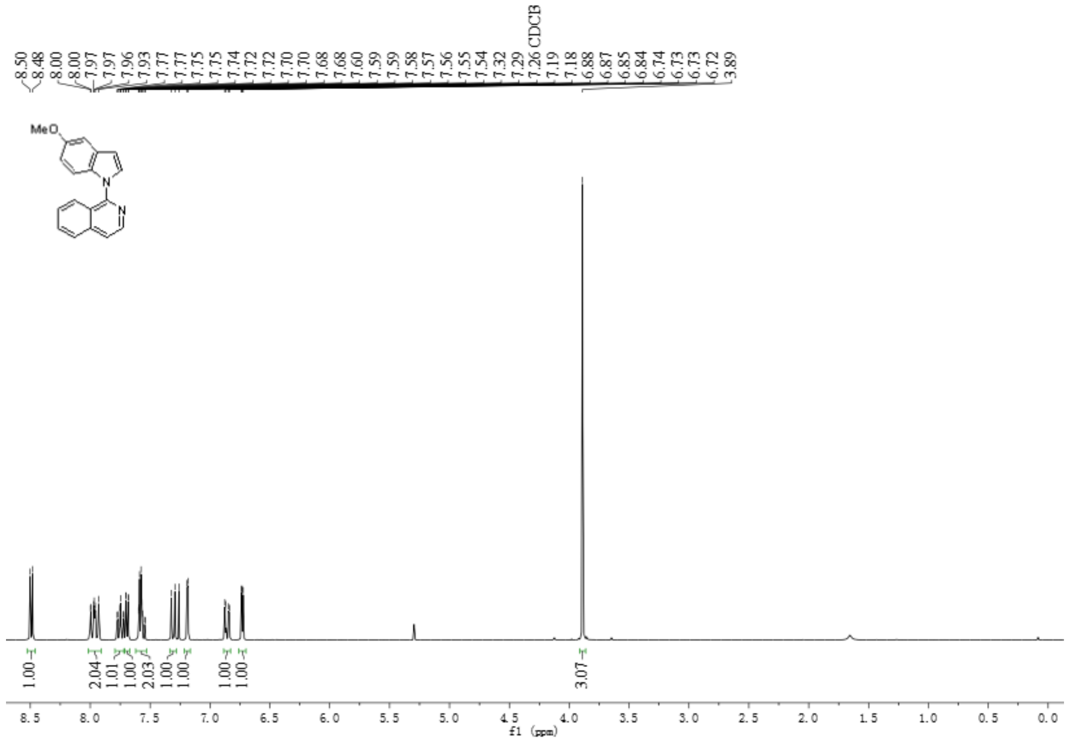

Supplementary Fig. 53. ^{13}C NMR spectrum of **1g**

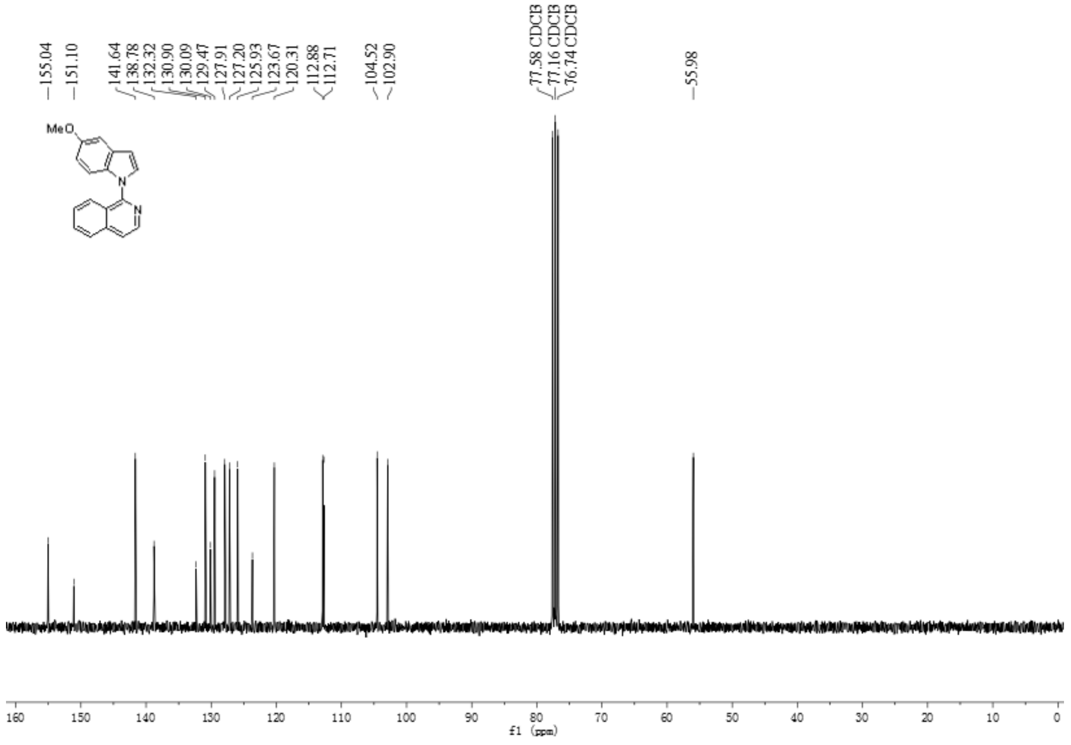

Supplementary Fig. 54. ^{1}H NMR spectrum of **1h**

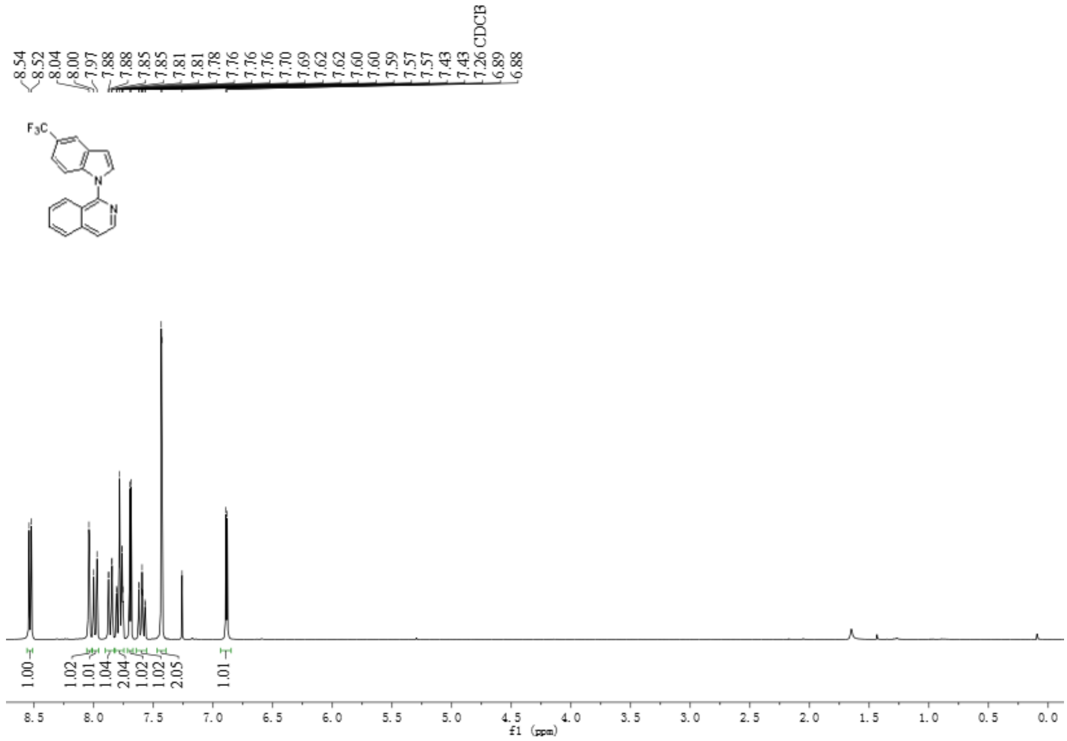

Supplementary Fig. 55. ^{13}C NMR spectrum of **1h**

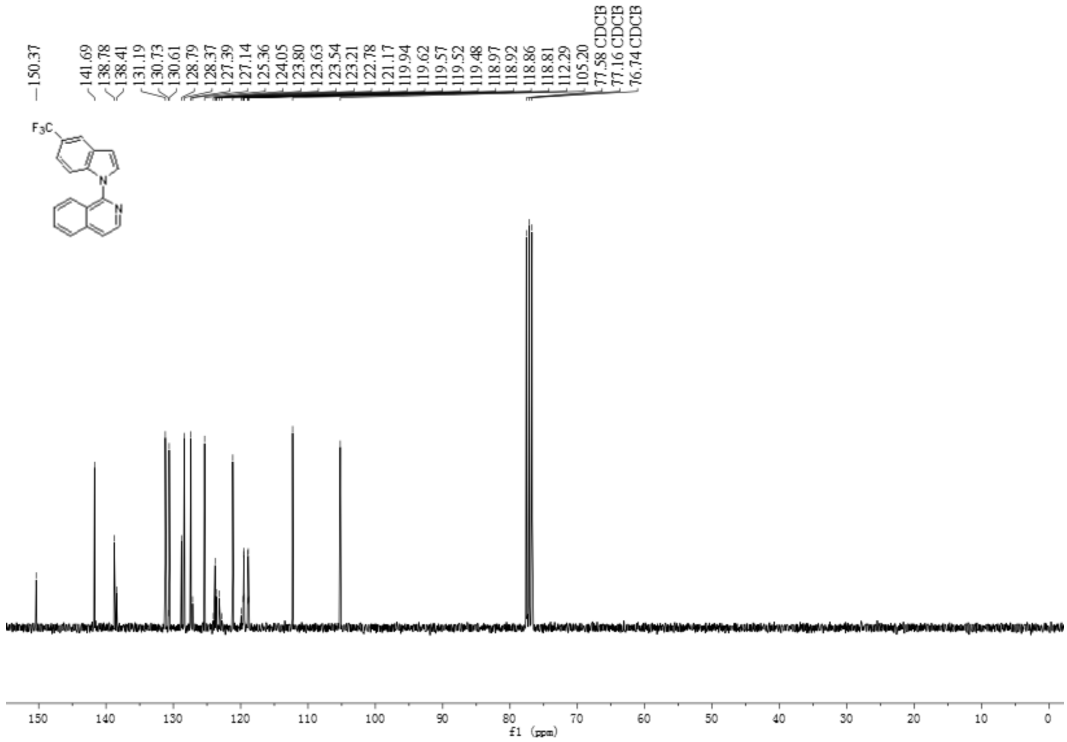

Supplementary Fig. 56. ^{19}F NMR spectrum of **1h**

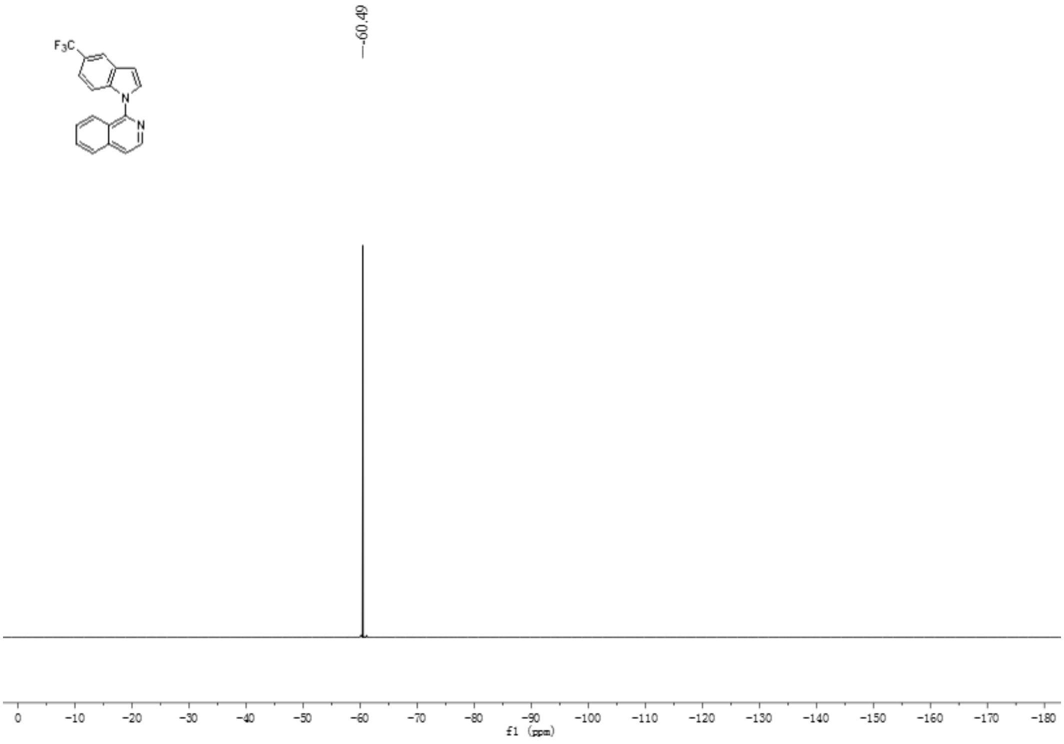

Supplementary Fig. 57. ^{1}H NMR spectrum of **1i**

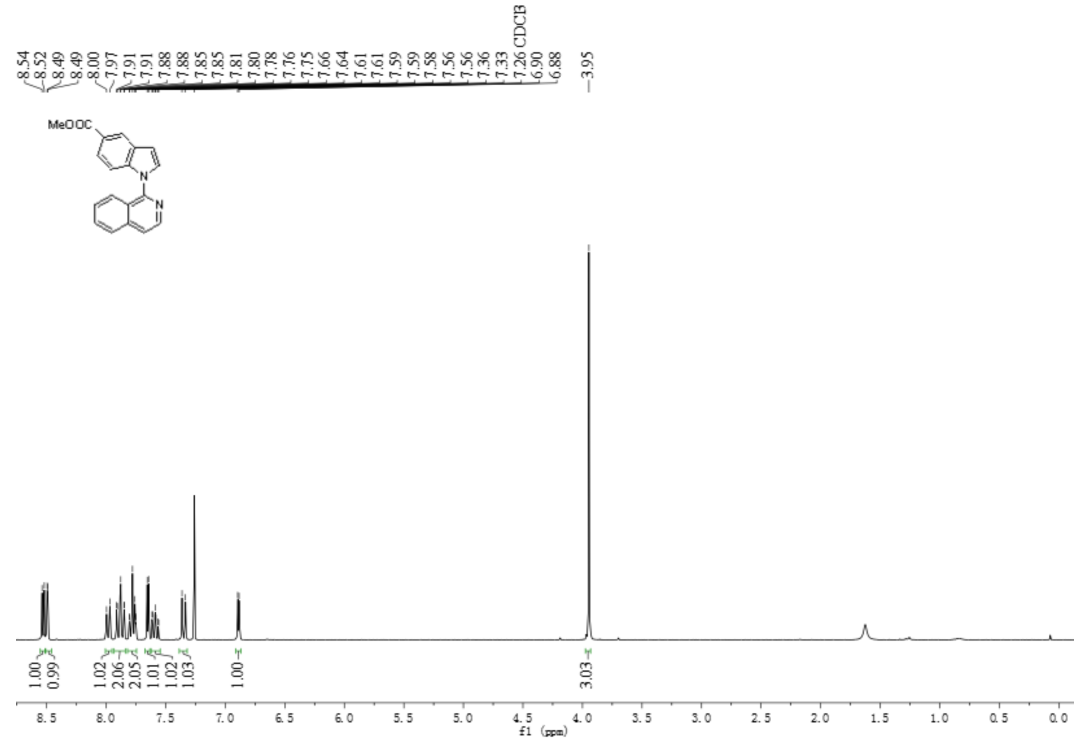

Supplementary Fig. 58. ^{13}C NMR spectrum of **1i**

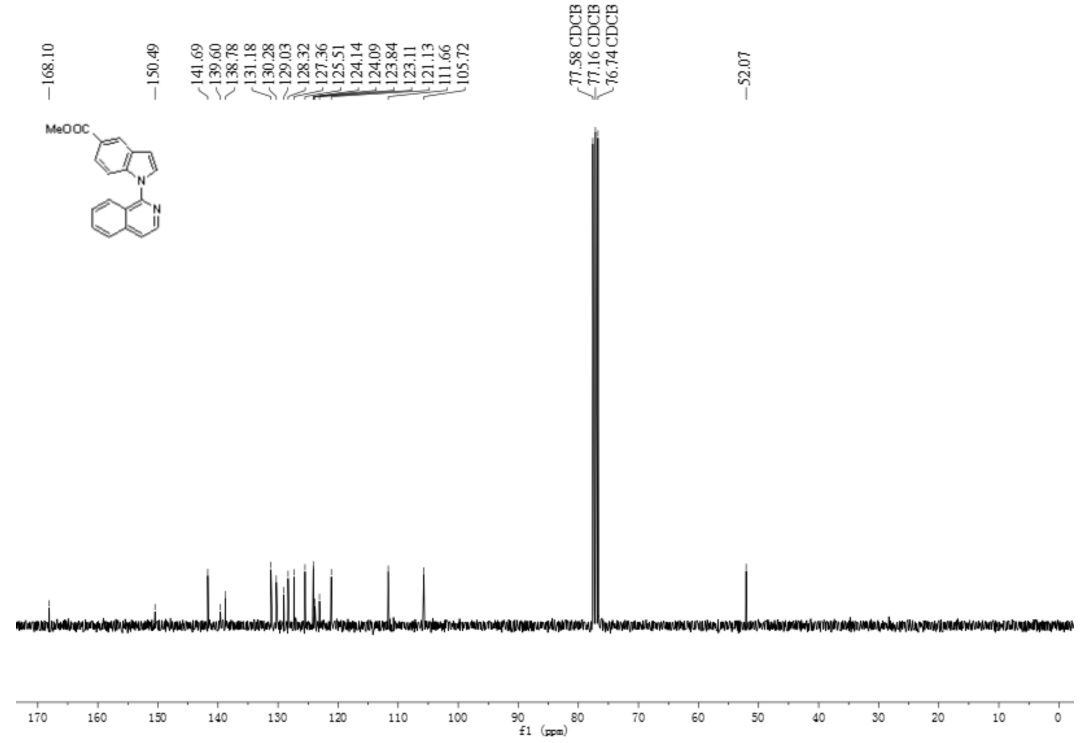

Supplementary Fig. 59. ^{1}H NMR spectrum of **1j**

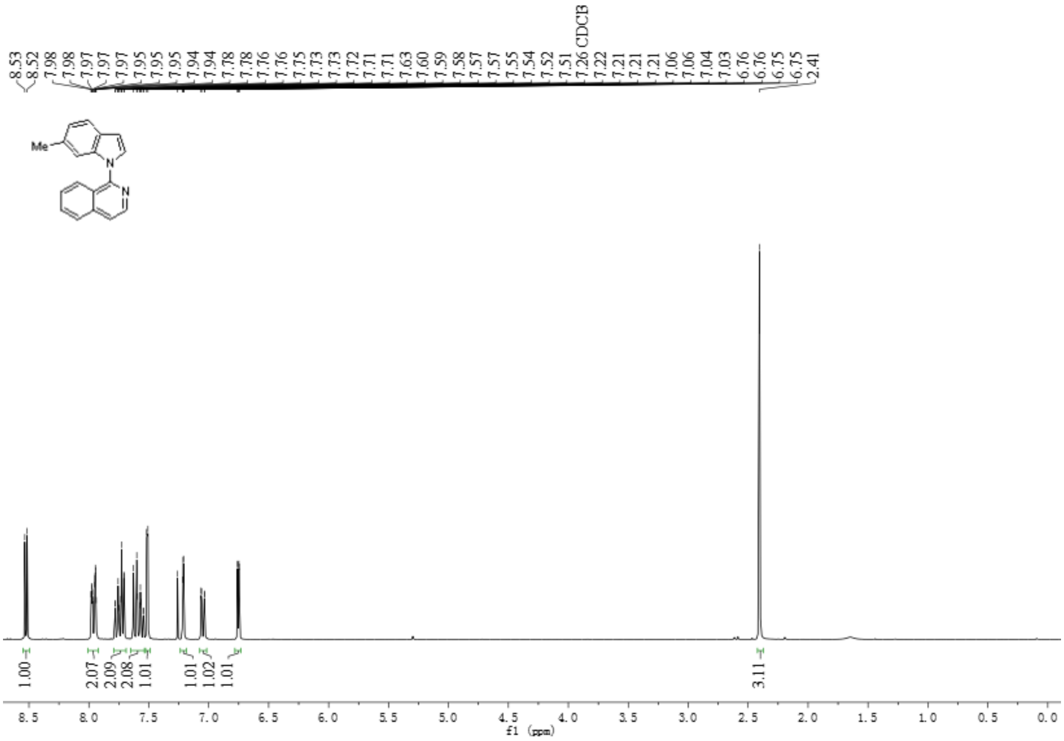

Supplementary Fig. 60. ^{13}C NMR spectrum of **1j**

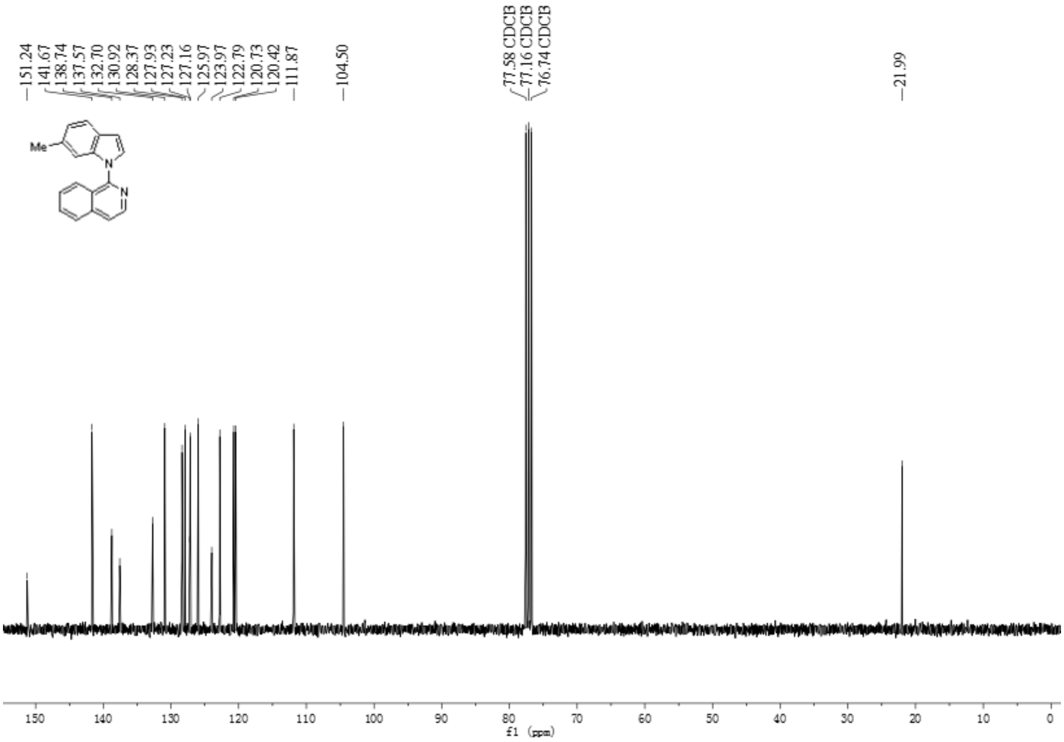

Supplementary Fig. 61. ^{1}H NMR spectrum of **1k**

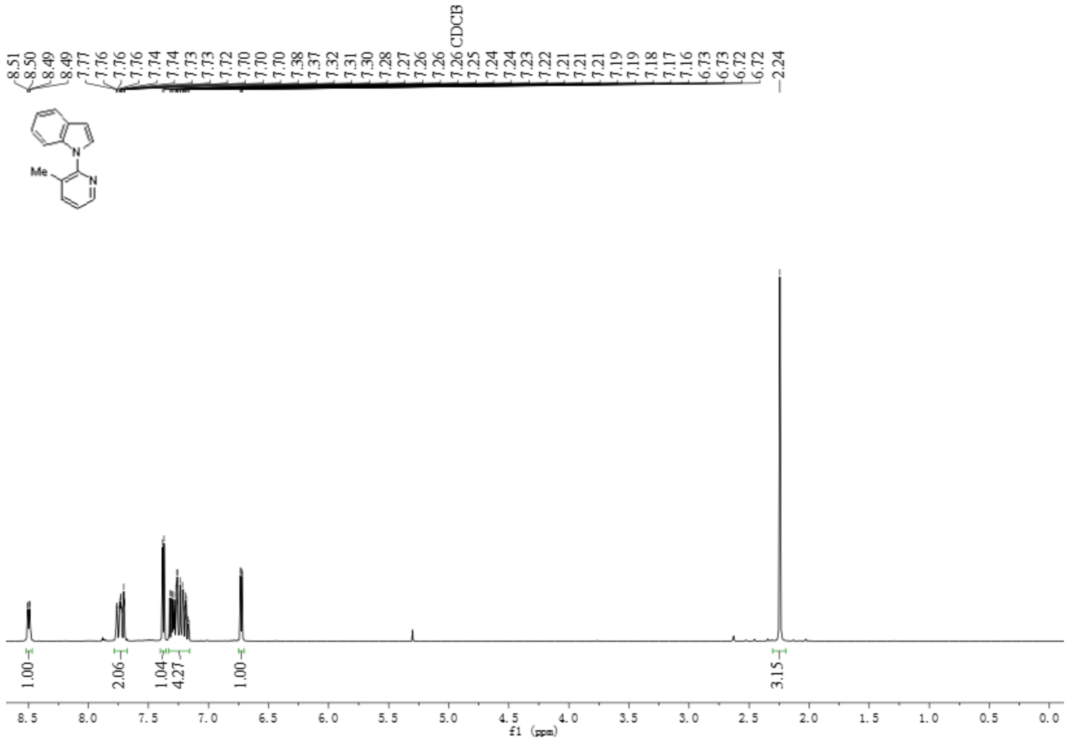

Supplementary Fig. 62. ^{13}C NMR spectrum of **1k**

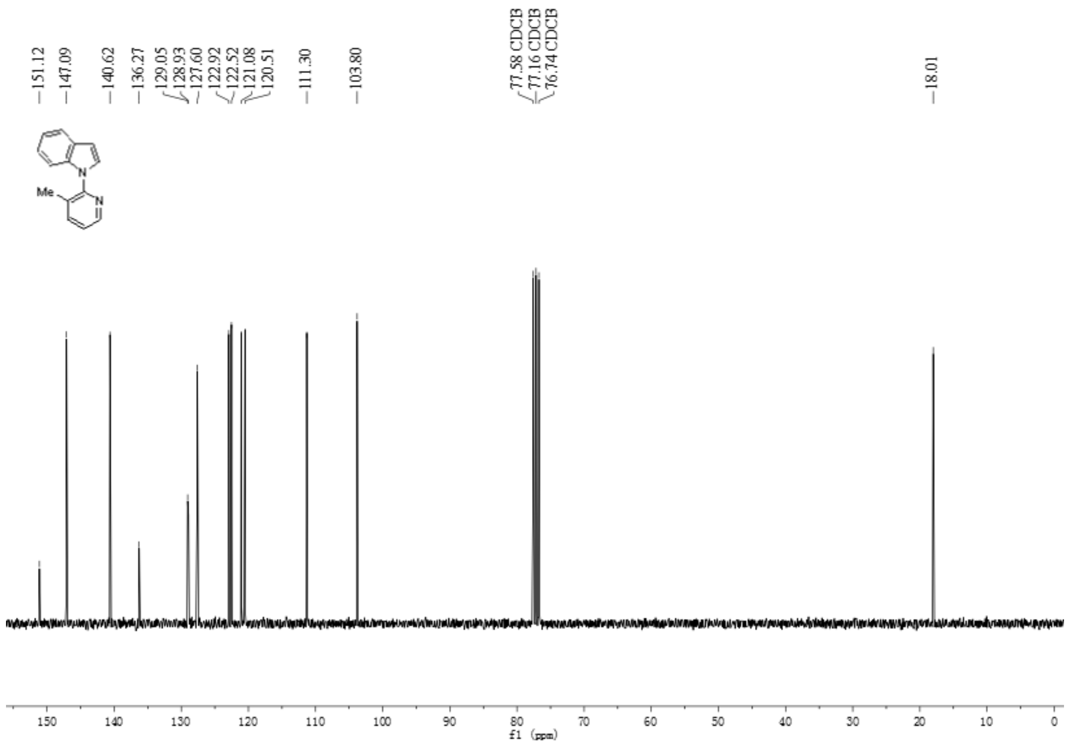

Supplementary Fig. 63. ^{1}H NMR spectrum of **3a**

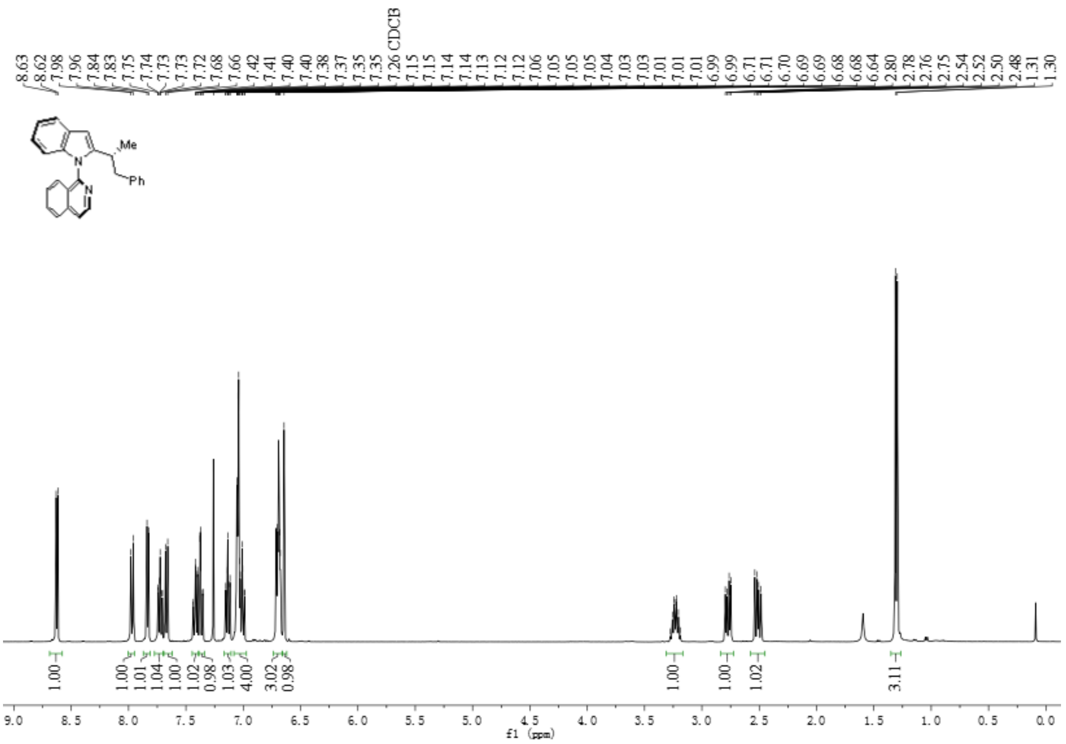

Supplementary Fig. 64. ^{13}C NMR spectrum of **3a**

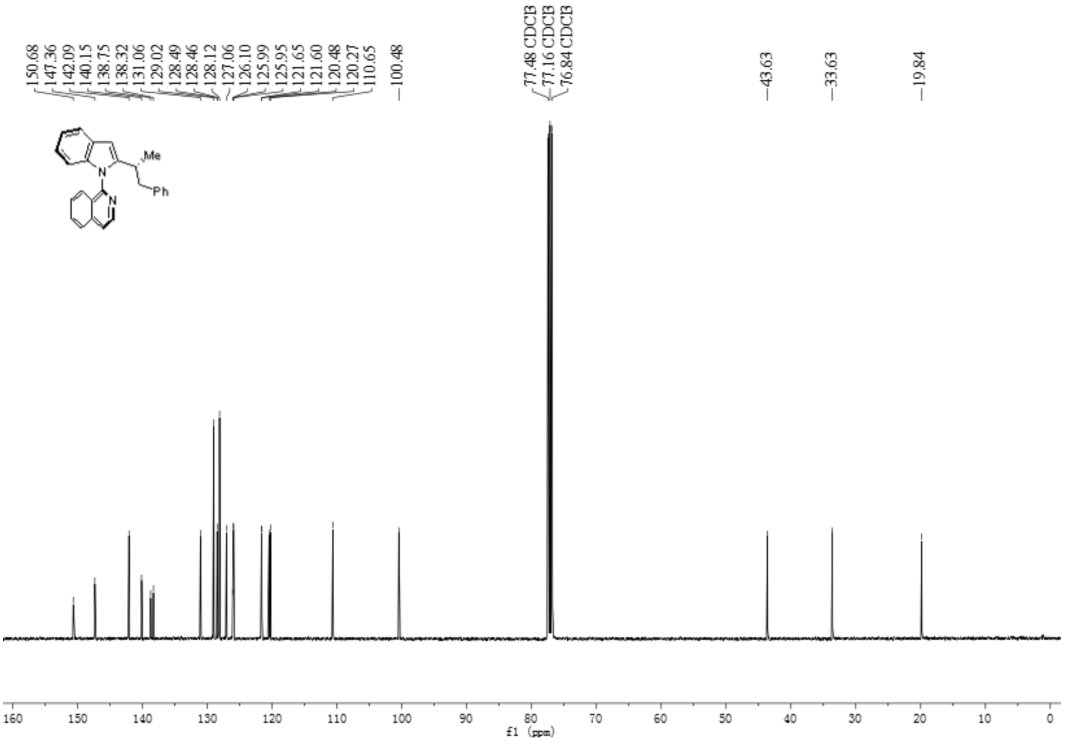

Supplementary Fig. 65. ^{1}H NMR spectrum of **3b**

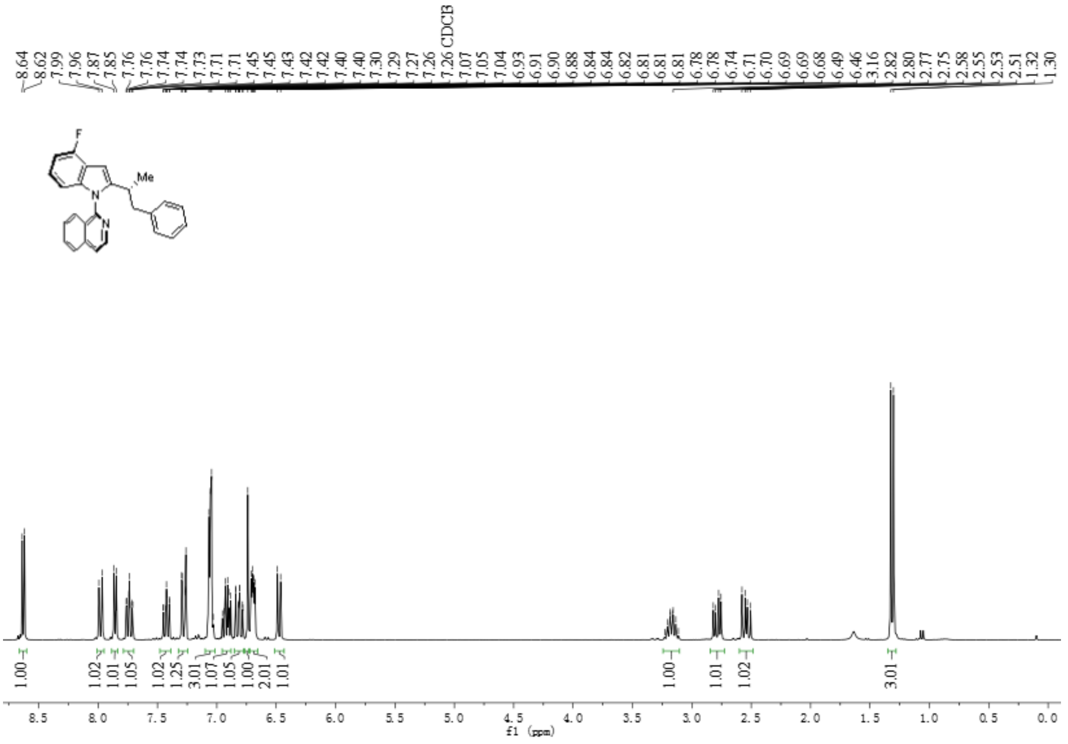

Supplementary Fig. 66. ^{13}C NMR spectrum of **3b**

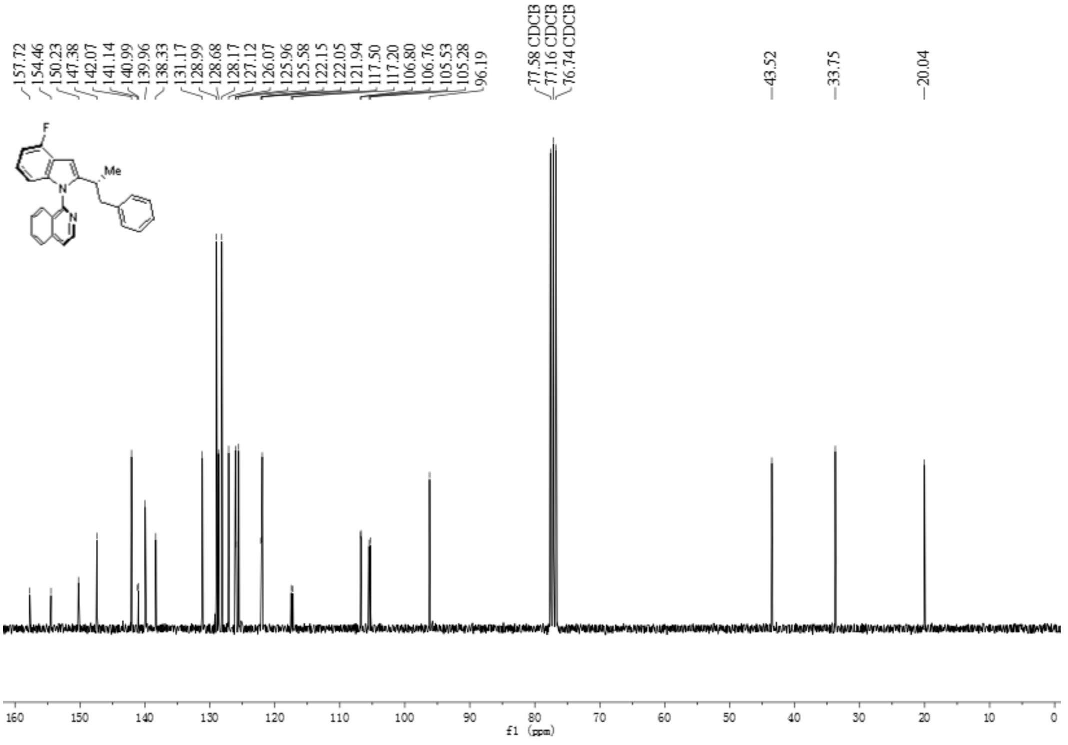

Supplementary Fig. 67. ^{19}F NMR spectrum of **3b**

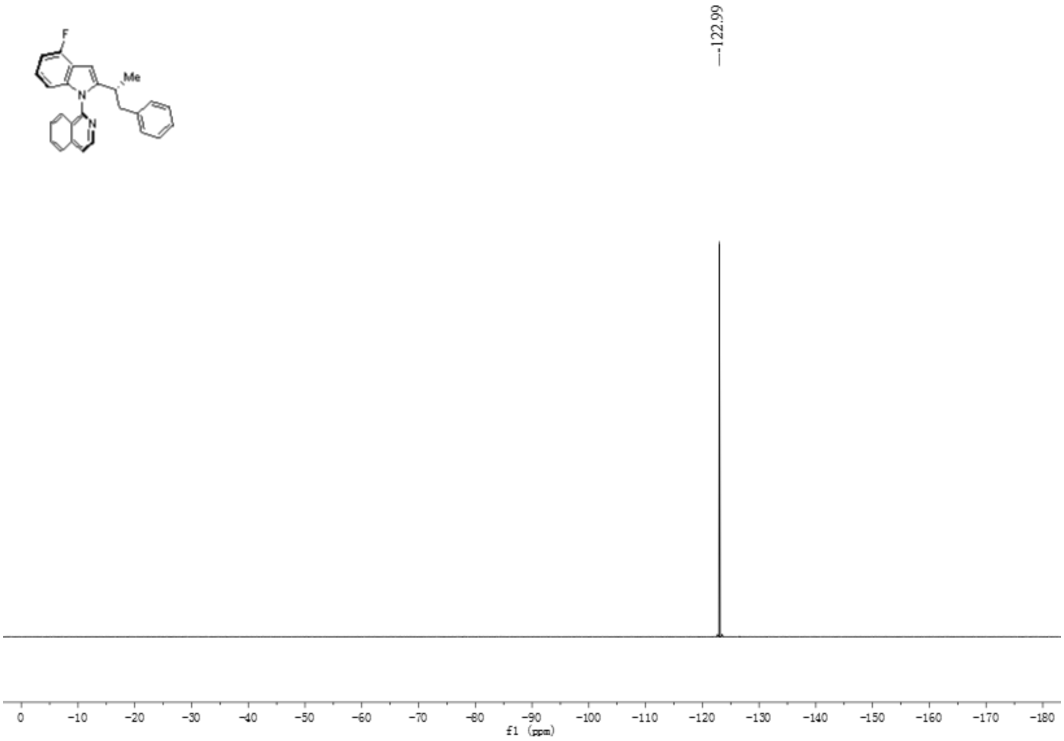

Supplementary Fig. 68. ^{1}H NMR spectrum of **3c**

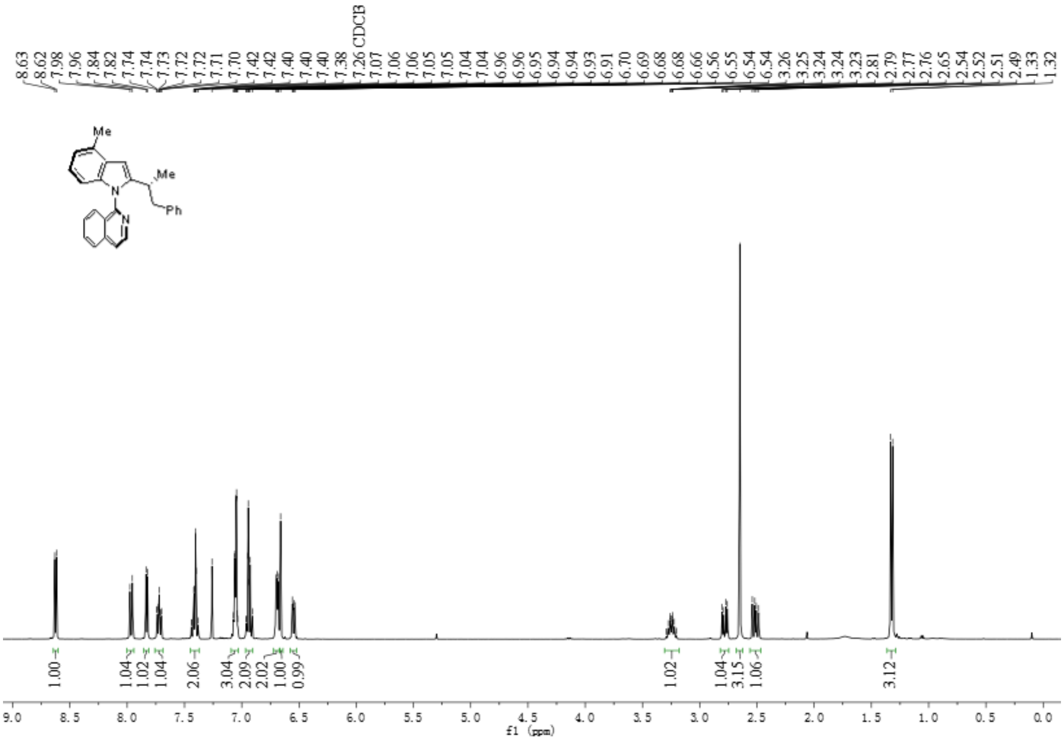

Supplementary Fig. 69. ^{13}C NMR spectrum of **3c**

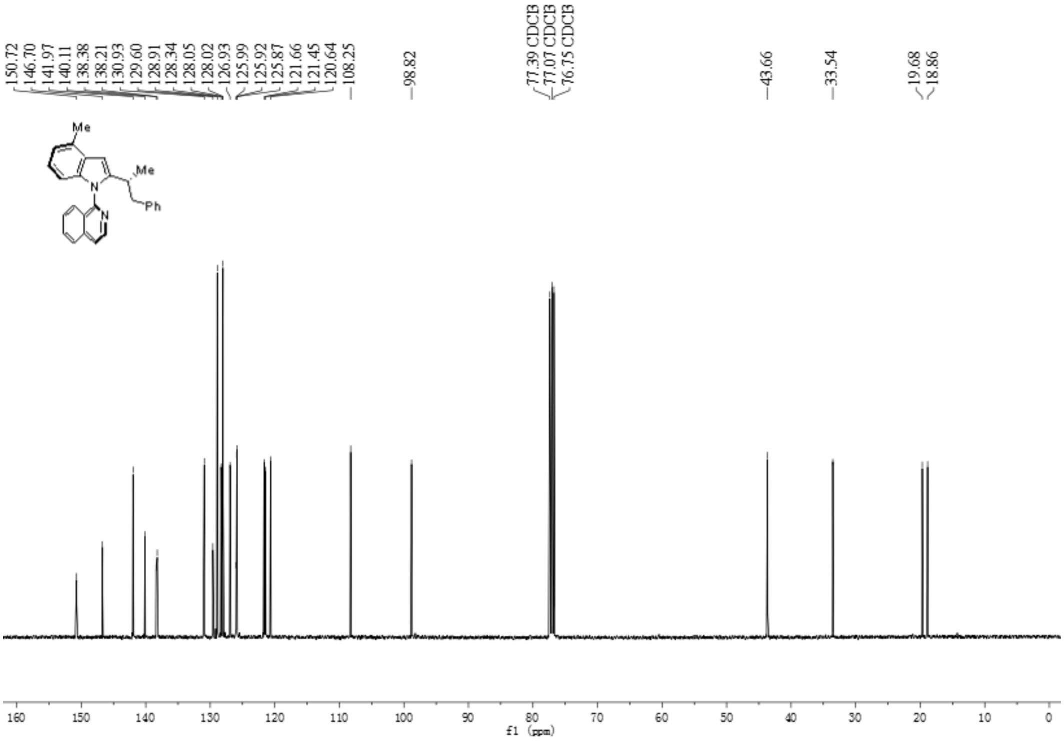

Supplementary Fig. 70. ^{1}H NMR spectrum of **3d**

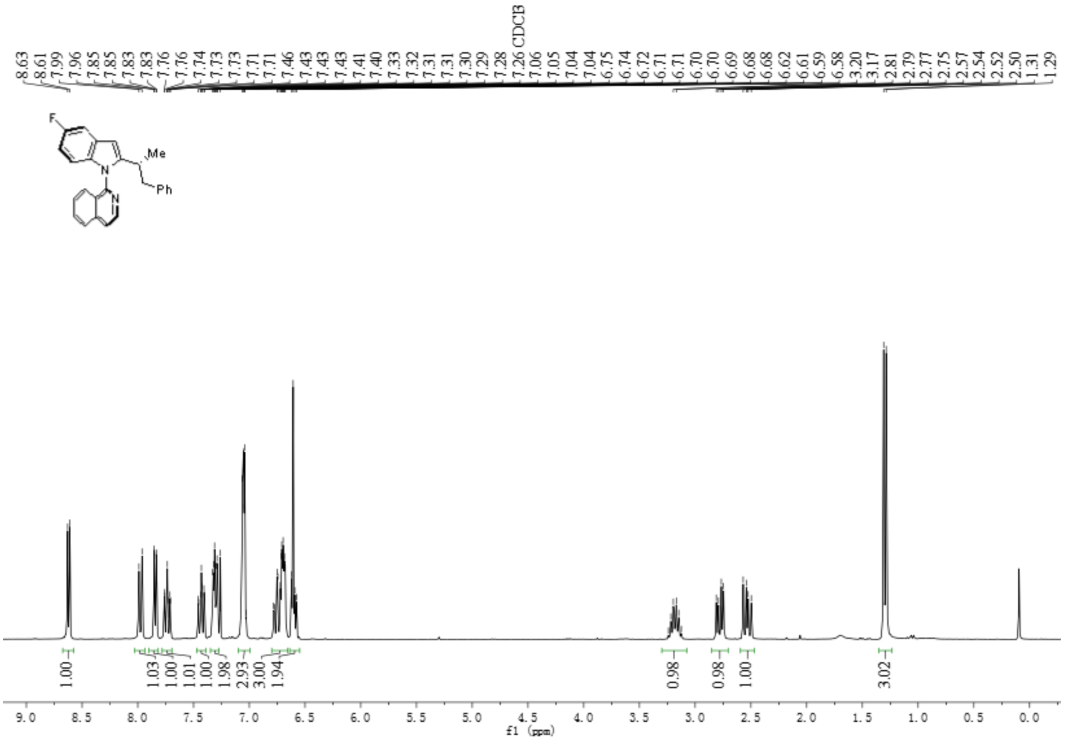

Supplementary Fig. 71. ^{13}C NMR spectrum of **3d**

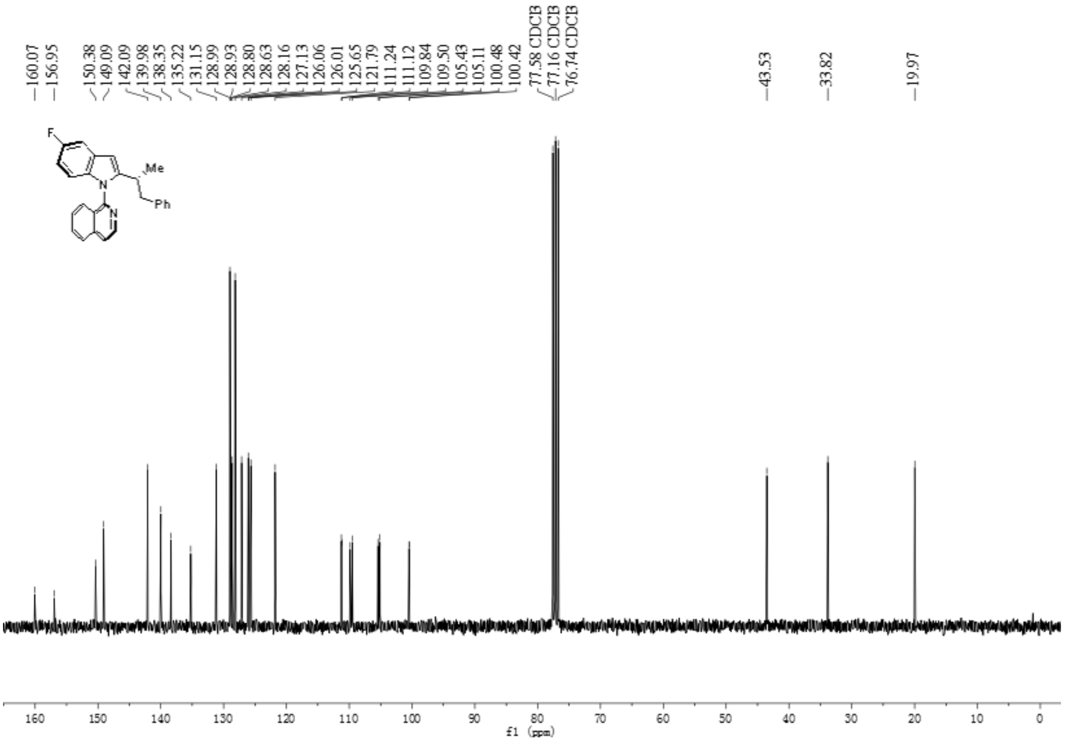

Supplementary Fig. 72. ^{19}F NMR spectrum of **3d**

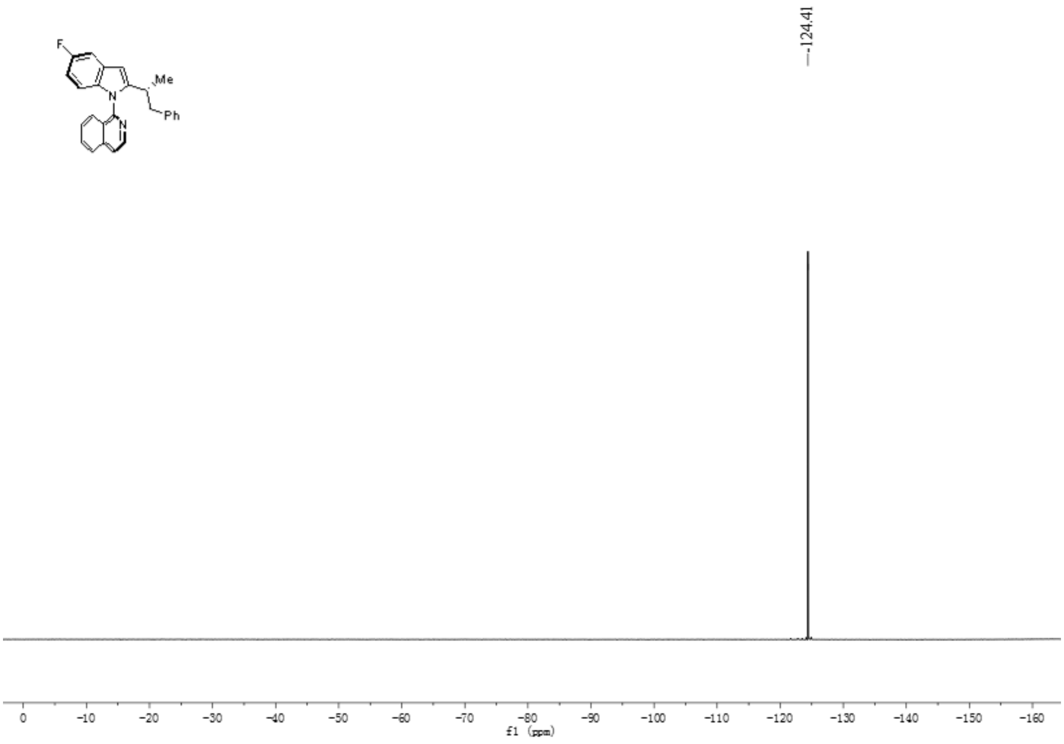

Supplementary Fig. 73. ^{1}H NMR spectrum of **3e**

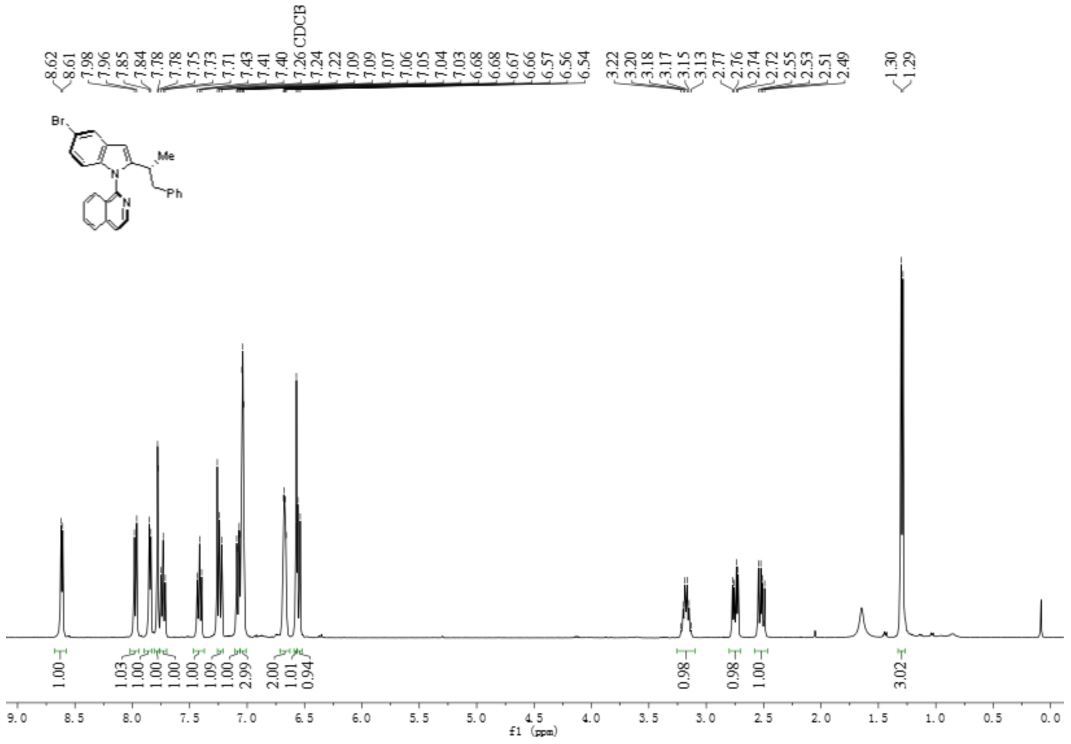

Supplementary Fig. 74. ^{13}C NMR spectrum of **3e**

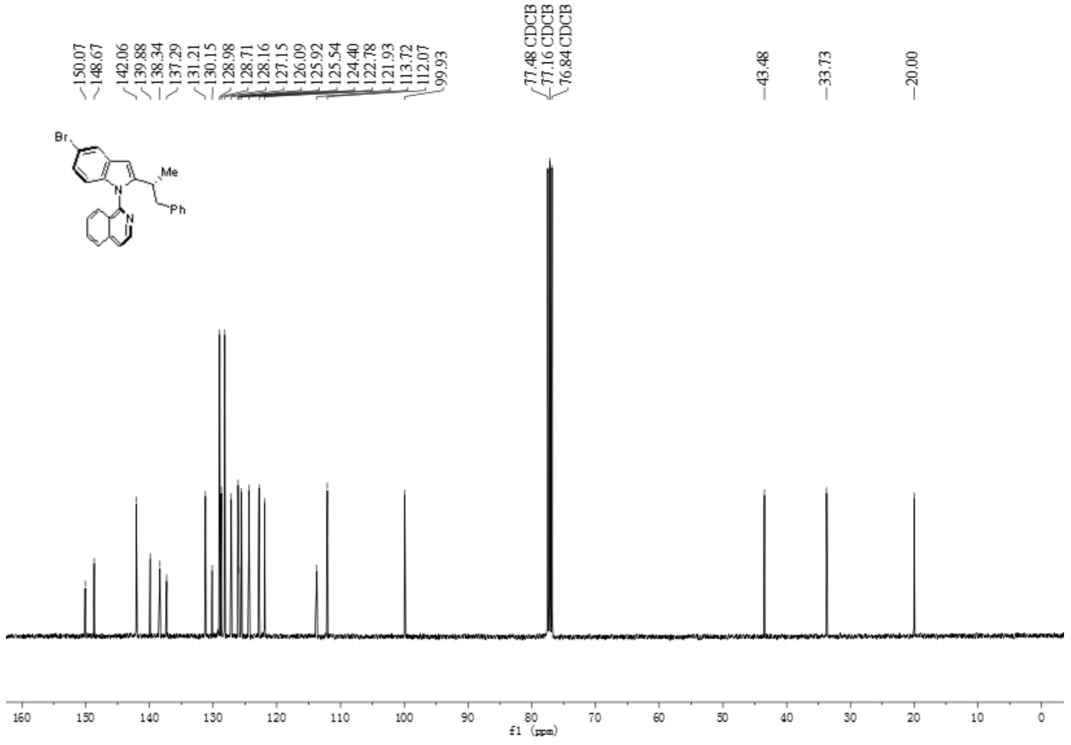

Supplementary Fig. 75. ^{1}H NMR spectrum of **3f**

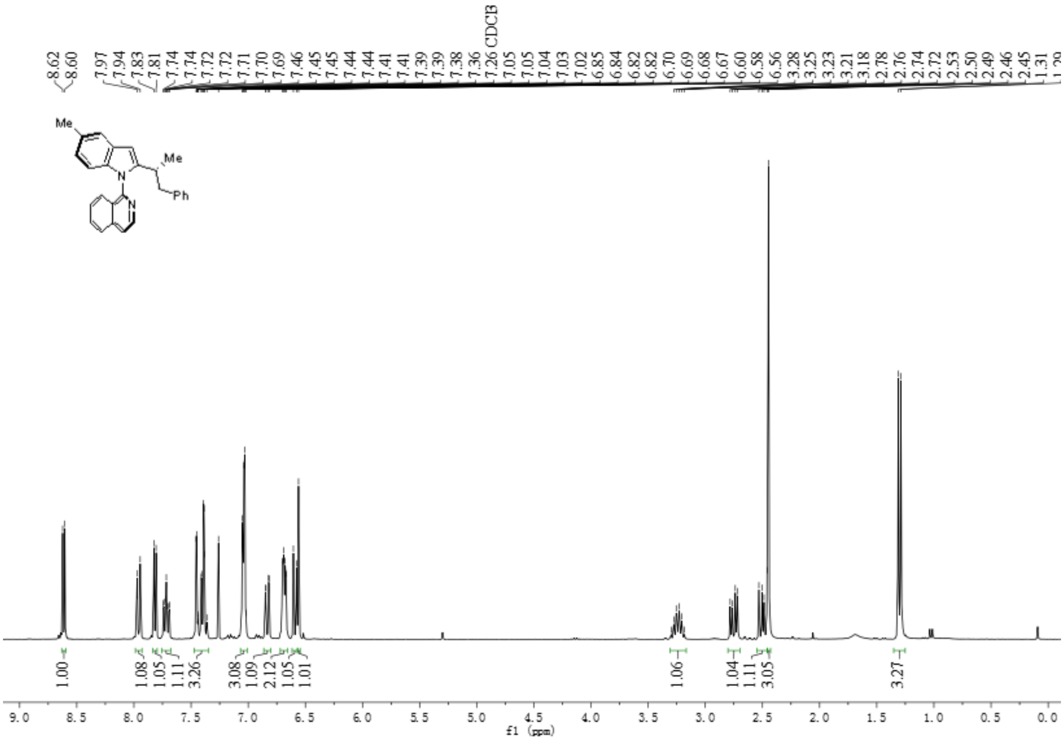

Supplementary Fig. 76. ^{13}C NMR spectrum of **3f**

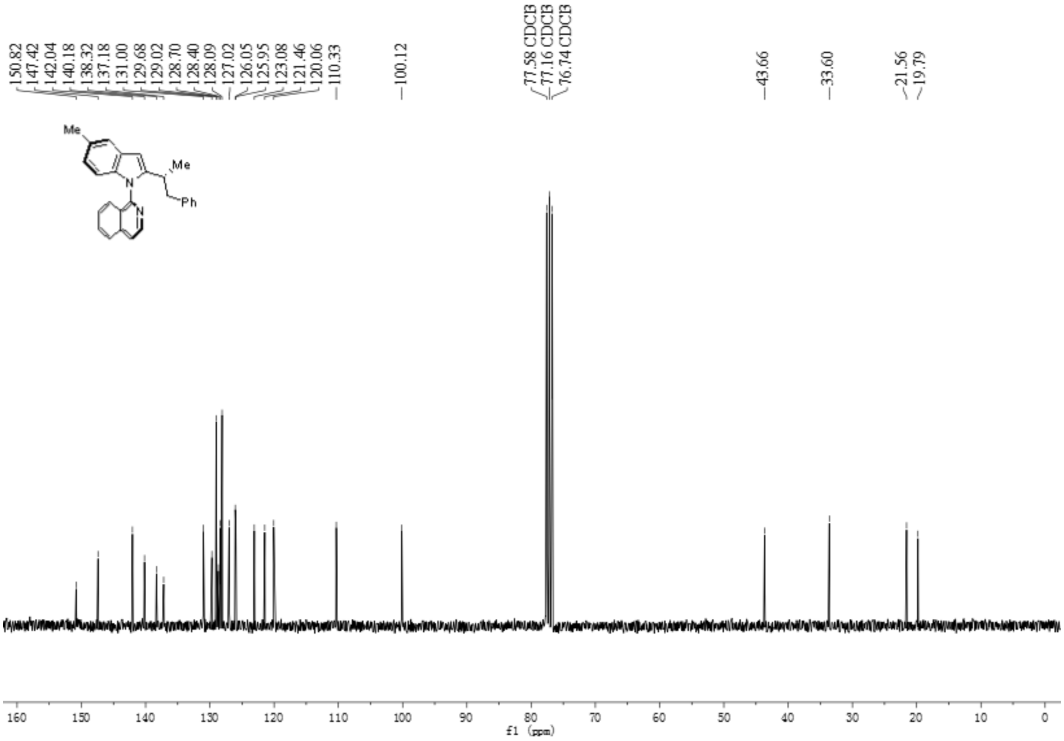

Supplementary Fig. 77. ^{1}H NMR spectrum of **3g**

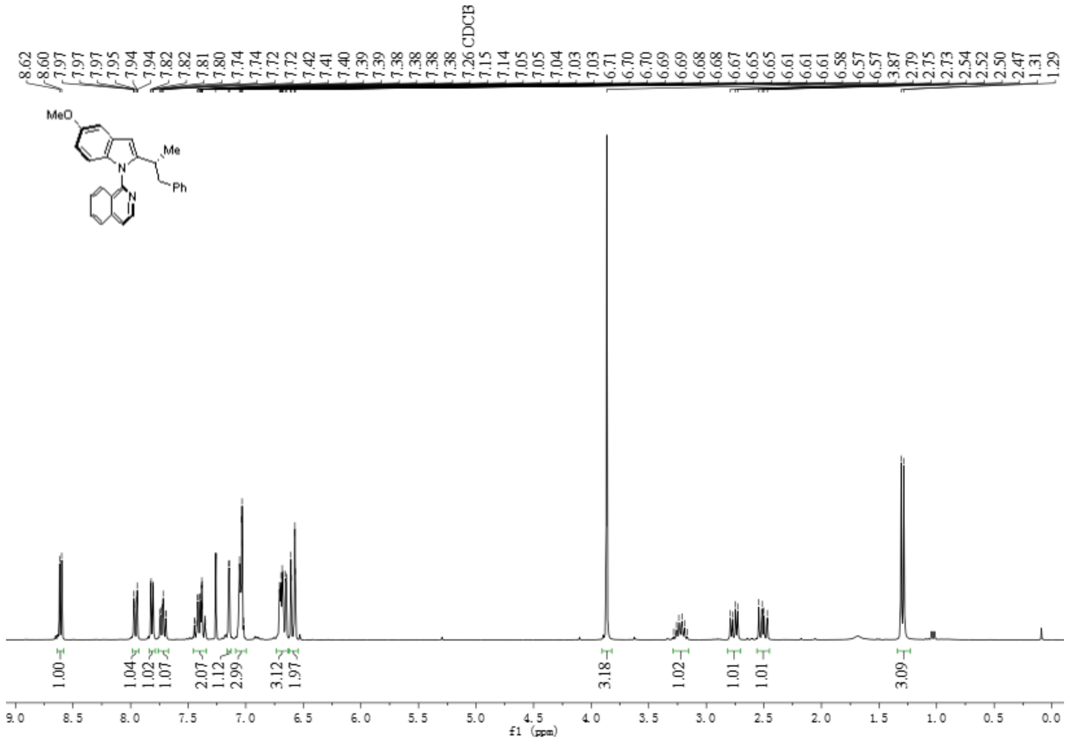

Supplementary Fig. 78. ^{13}C NMR spectrum of **3g**

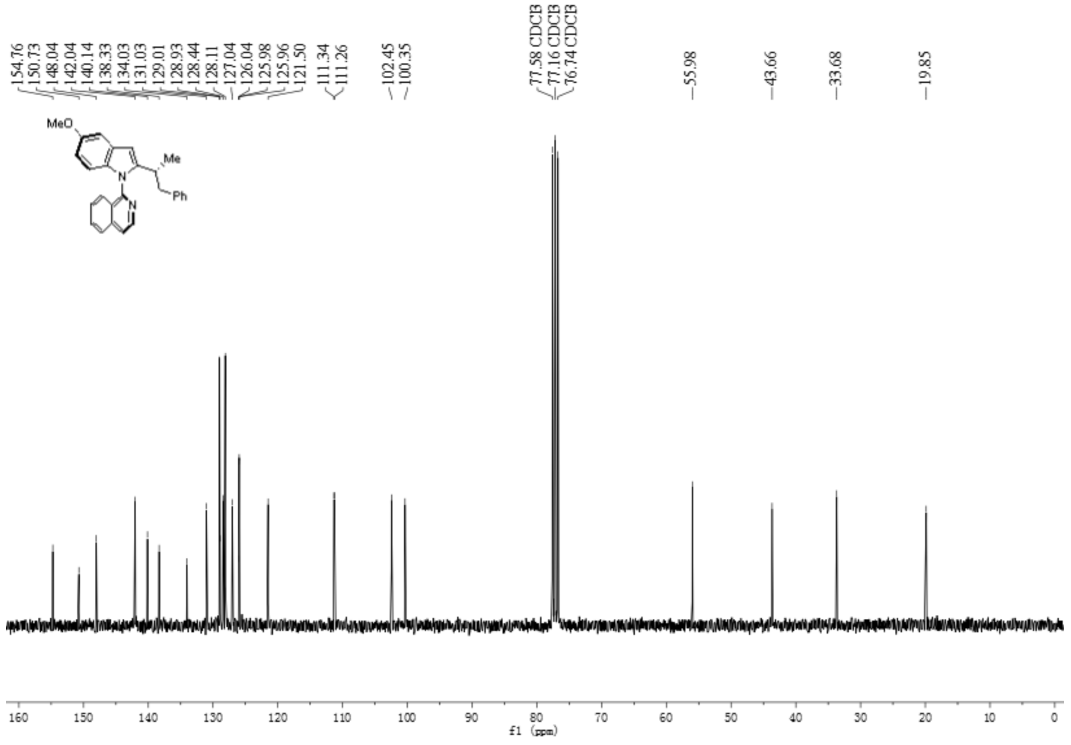

Supplementary Fig. 79. ^{1}H NMR spectrum of **3h**

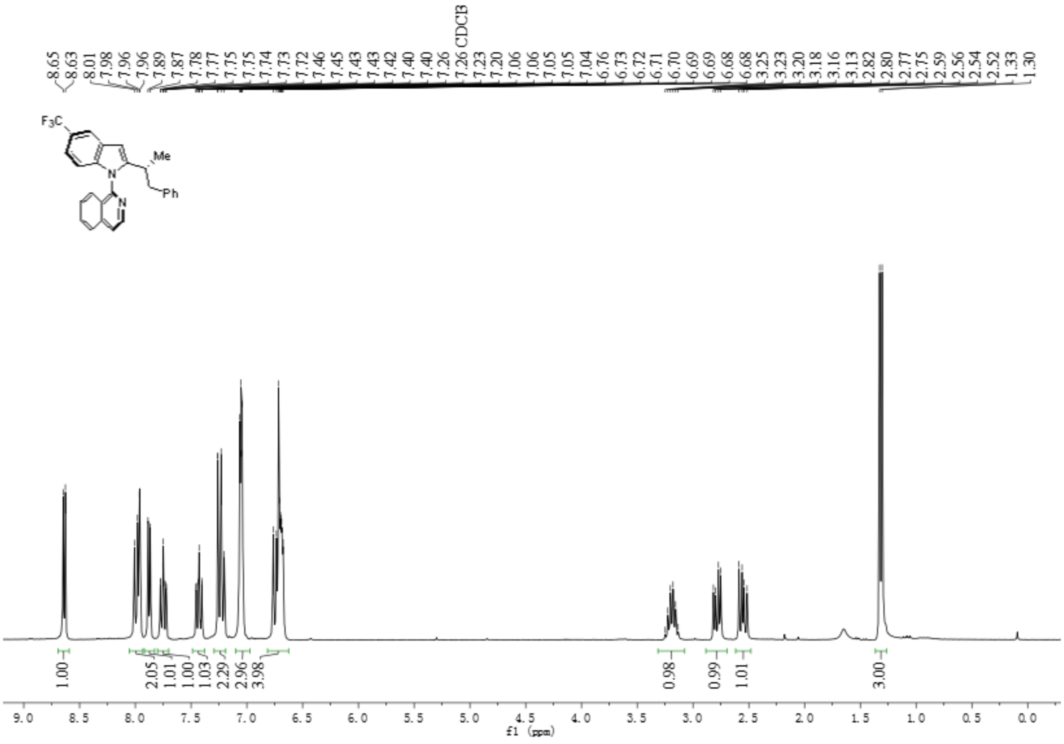

Supplementary Fig. 80. ^{13}C NMR spectrum of **3h**

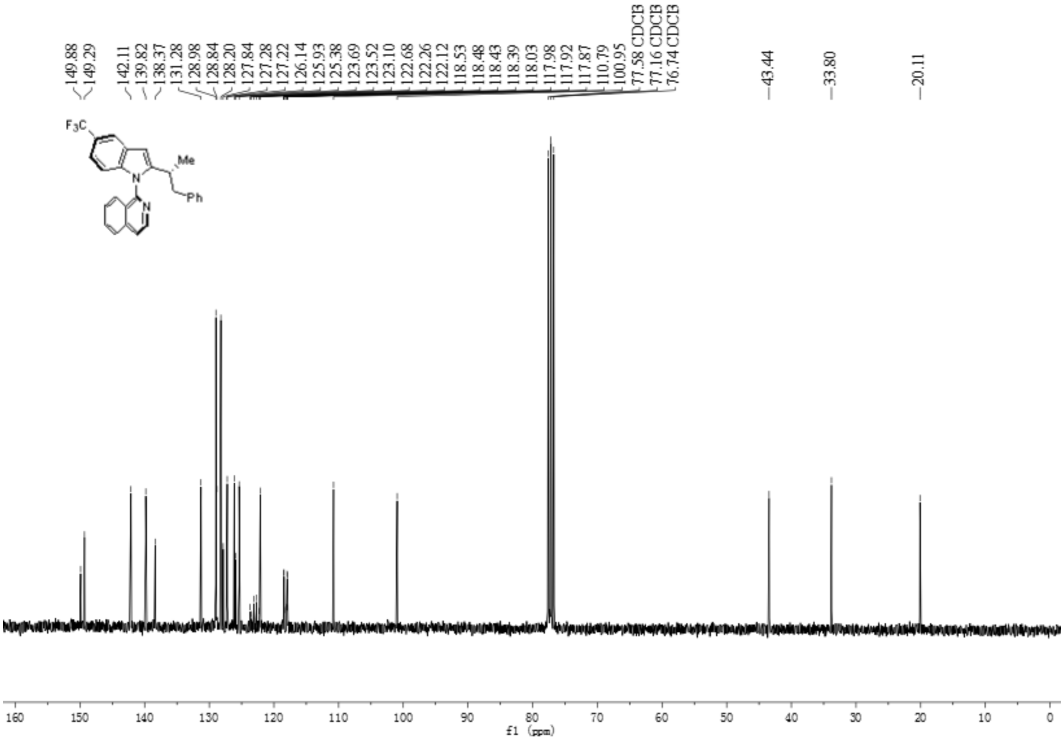

Supplementary Fig. 81. ^{19}F NMR spectrum of **3h**

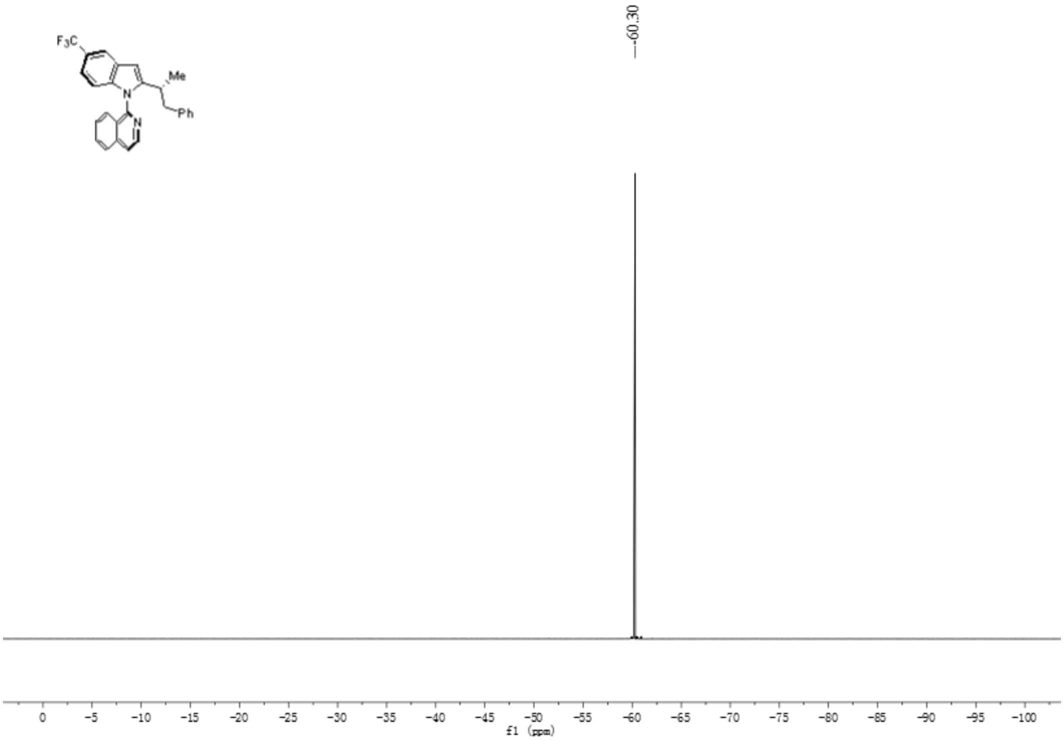

Supplementary Fig. 82. ^{1}H NMR spectrum of **3i**

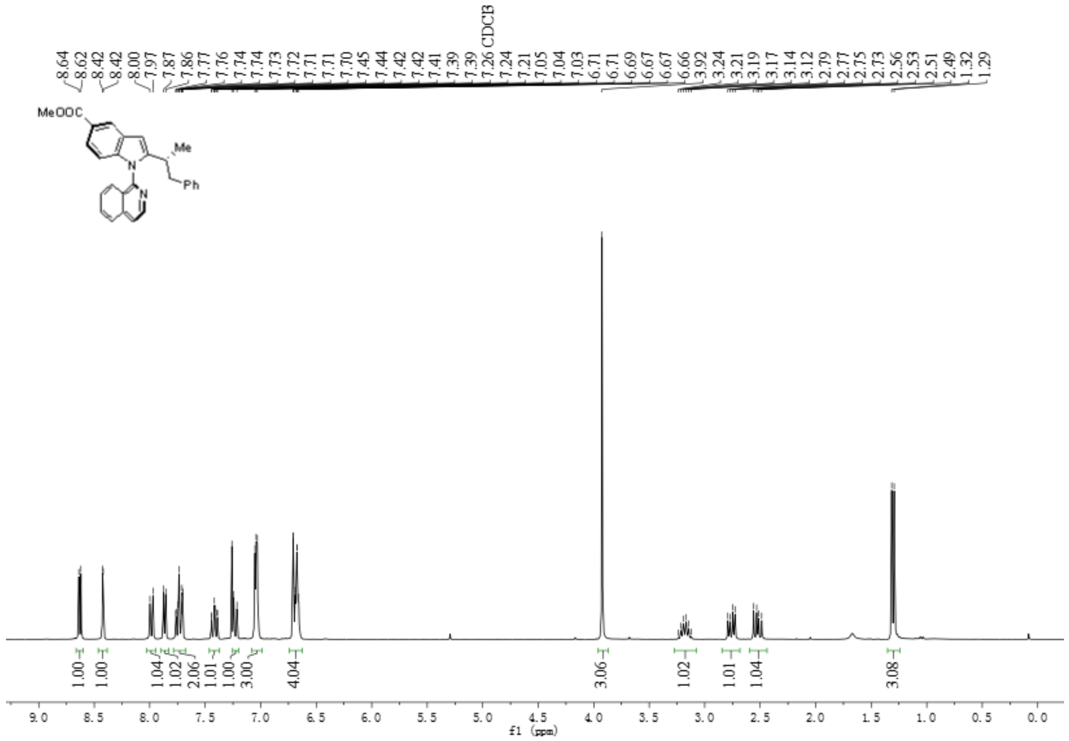

Supplementary Fig. 83. ^{13}C NMR spectrum of **3i**

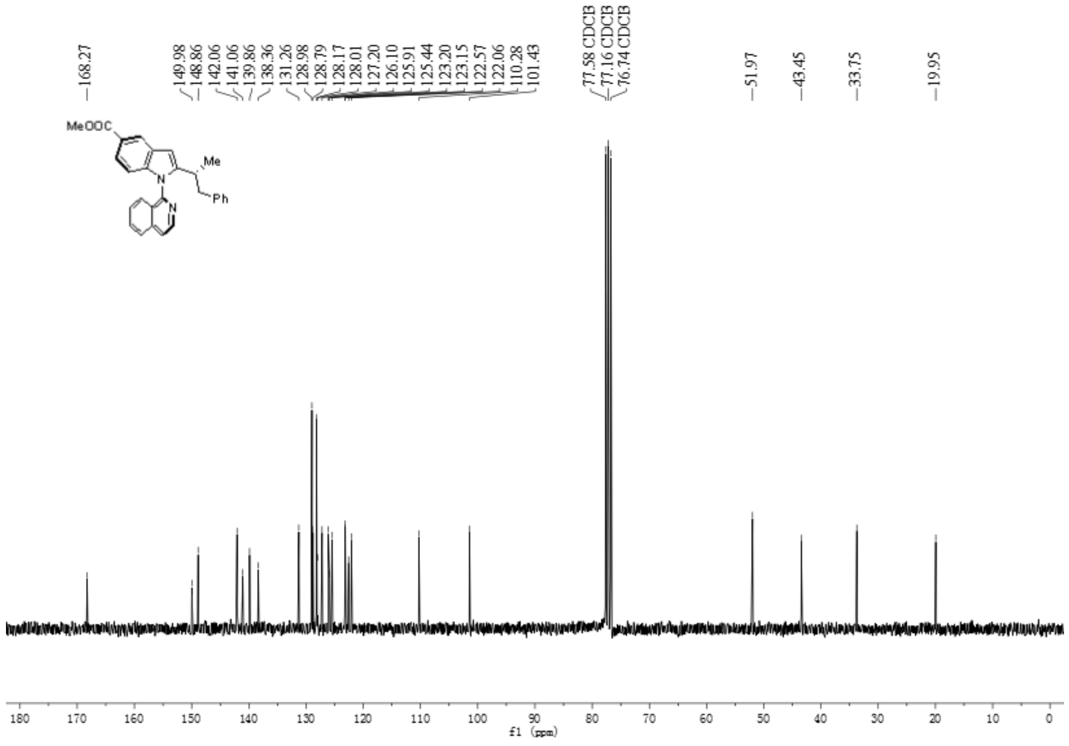

Supplementary Fig. 84. ^{1}H NMR spectrum of **3j**

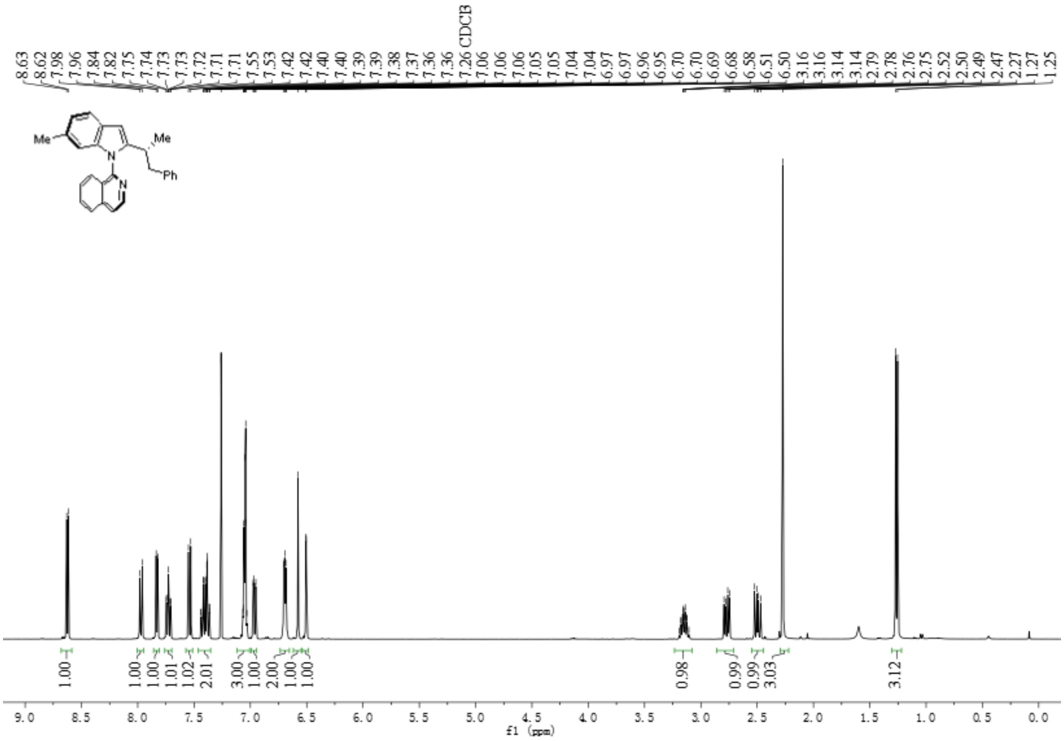

Supplementary Fig. 85. ^{13}C NMR spectrum of **3j**

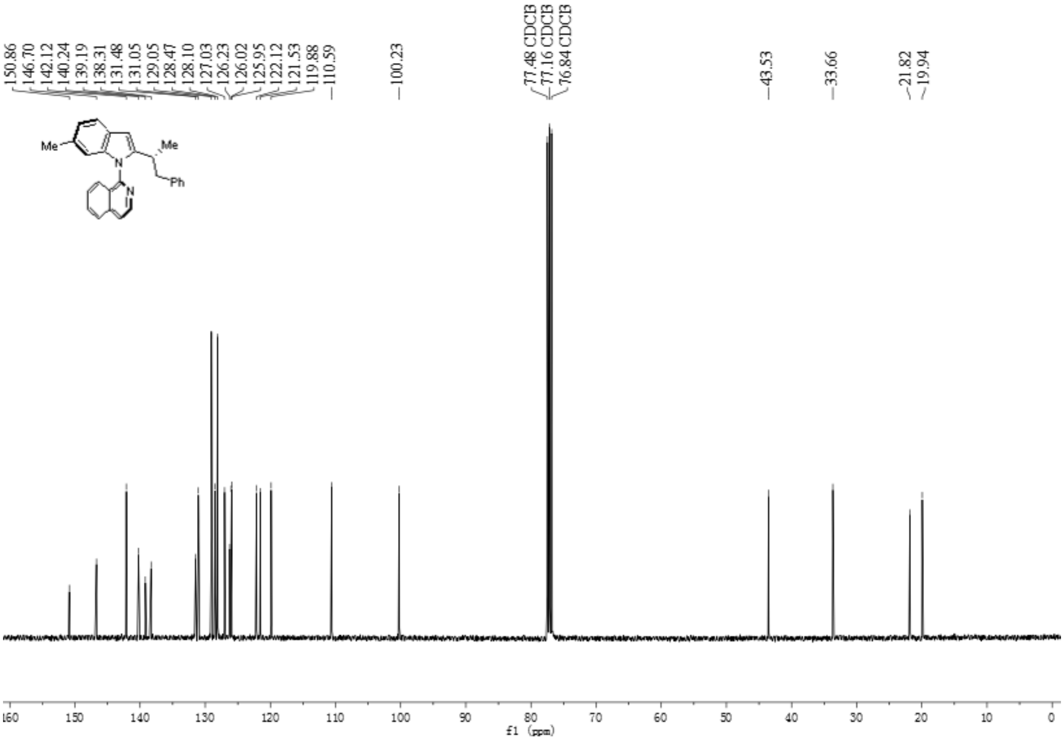

Supplementary Fig. 86. ^{1}H NMR spectrum of **3k**

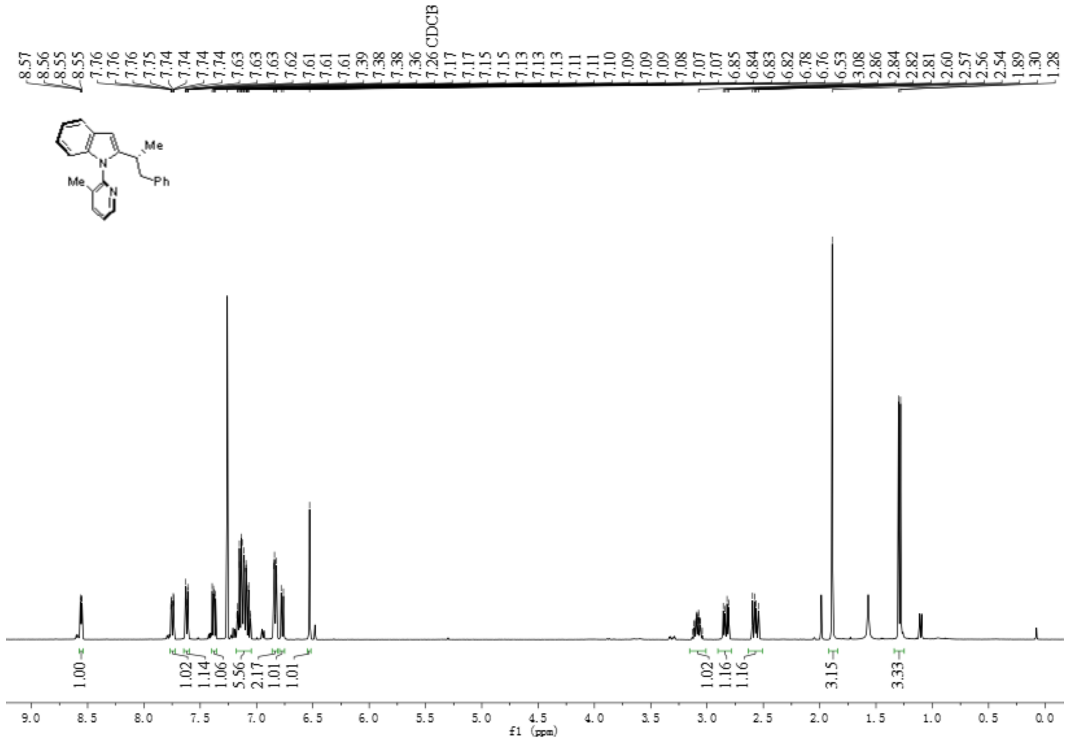

Supplementary Fig. 87. ^{13}C NMR spectrum of **3k**

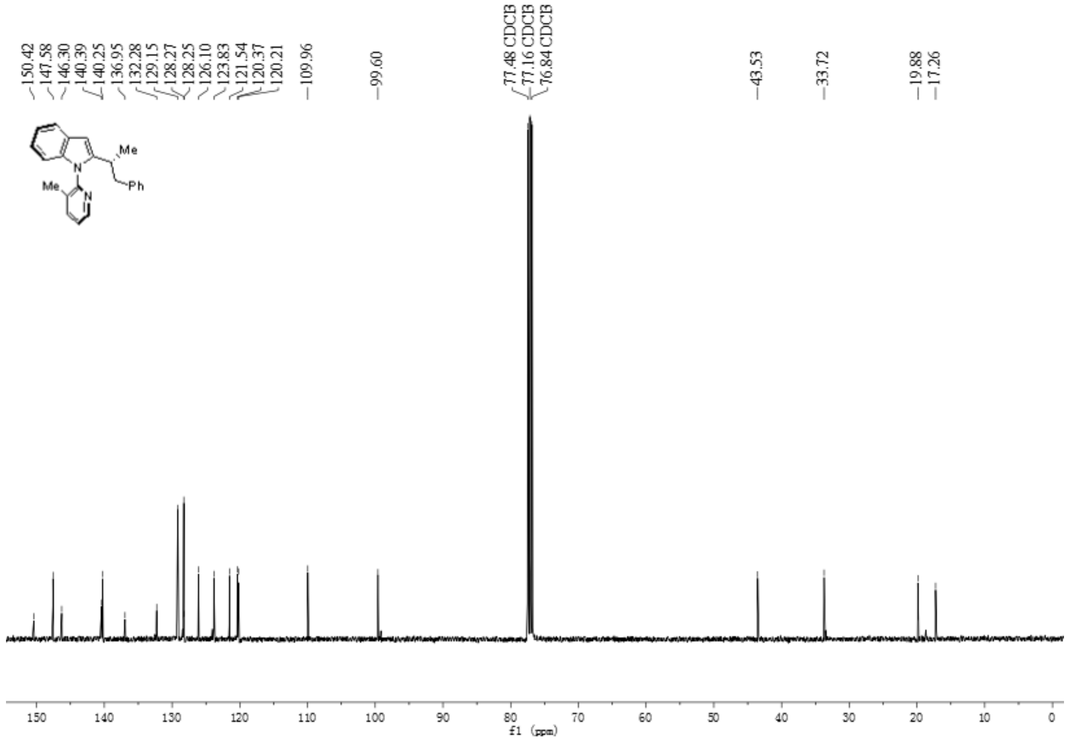

Supplementary Fig. 88. ^{1}H NMR spectrum of **3l**

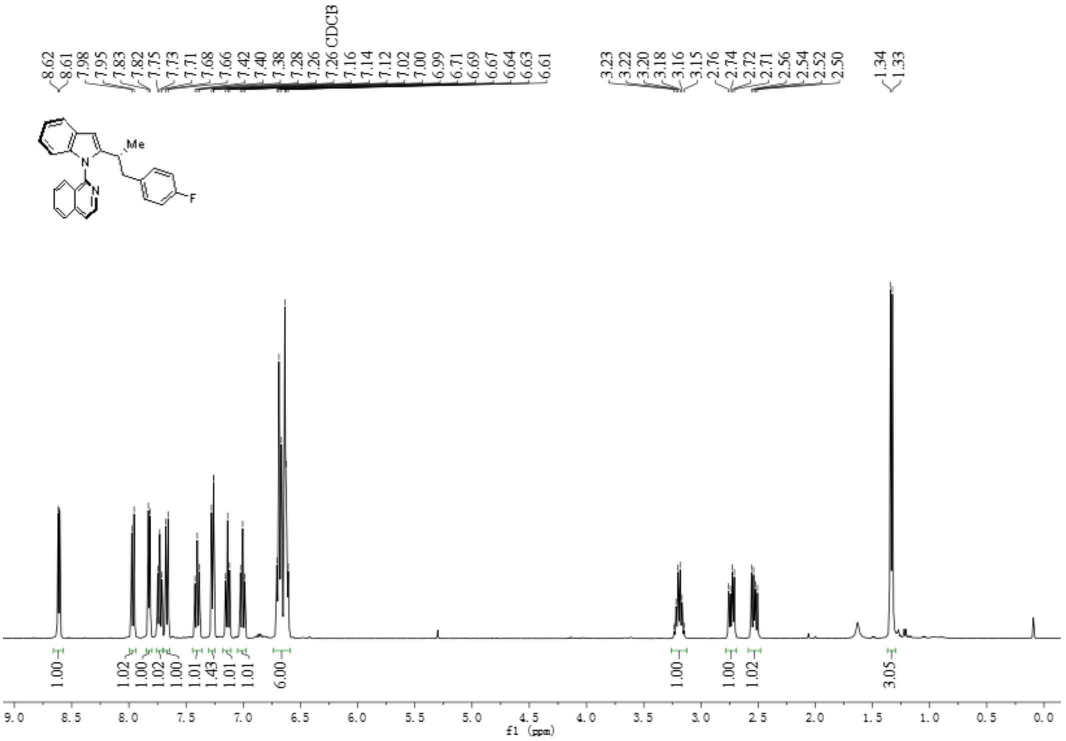

Supplementary Fig. 89. ^{13}C NMR spectrum of **3l**

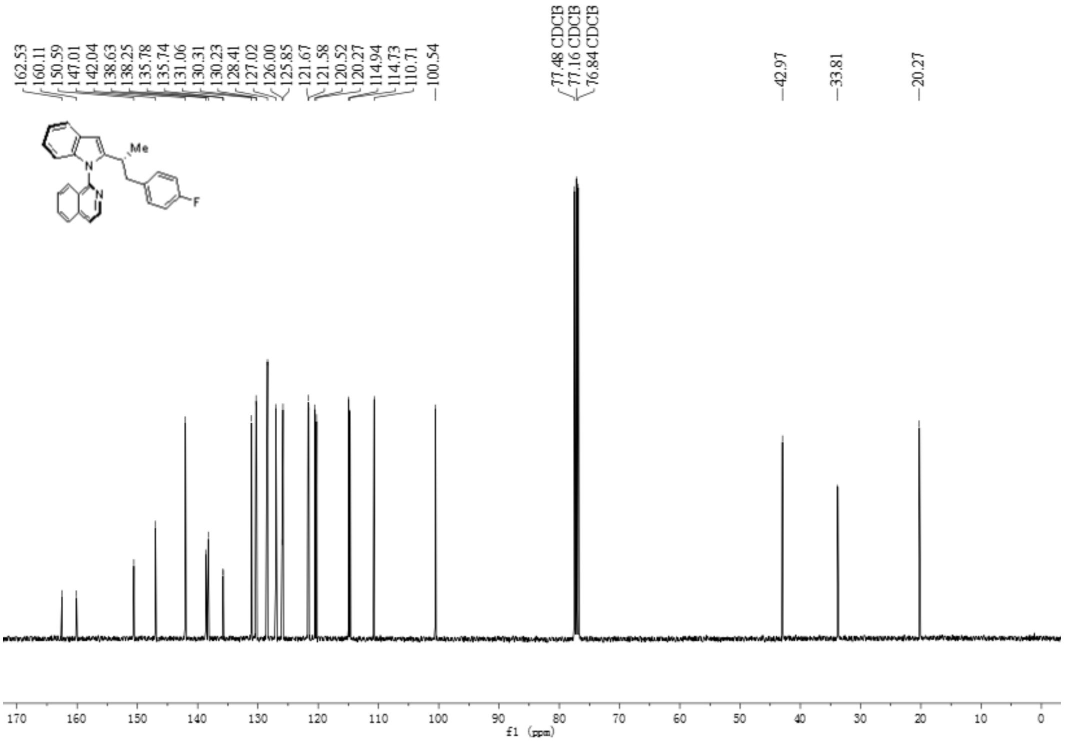

Supplementary Fig. 90. ^{19}F NMR spectrum of **3l**

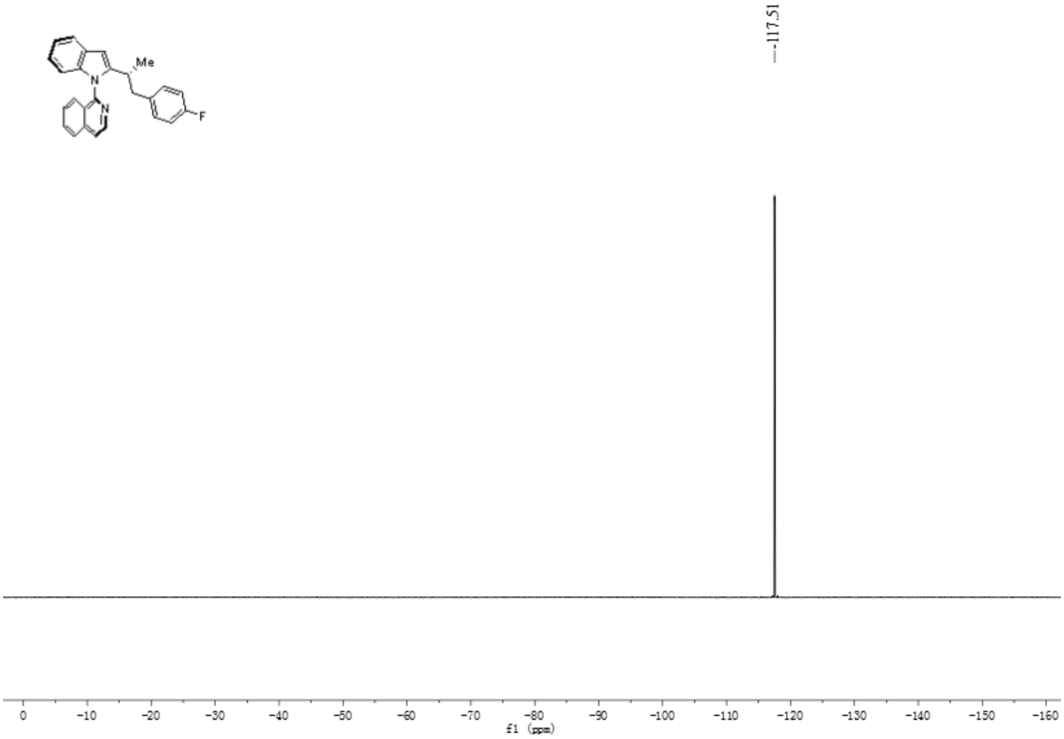

Supplementary Fig. 91. ^{1}H NMR spectrum of **3m**

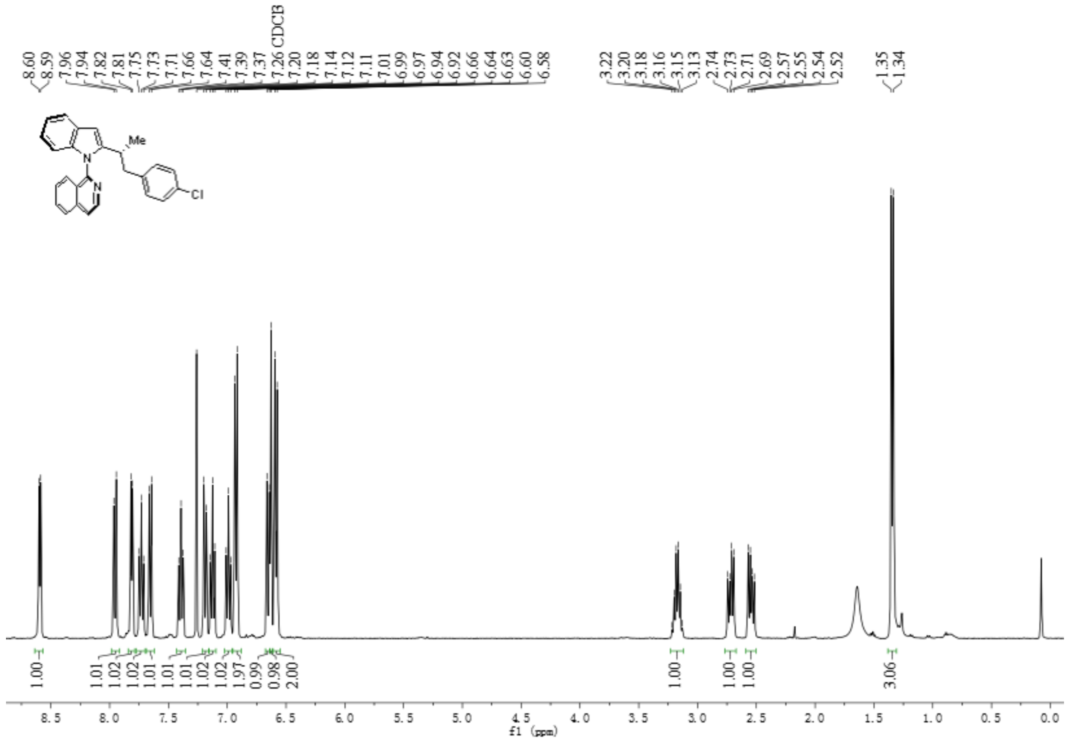

Supplementary Fig. 92. ^{13}C NMR spectrum of **3m**

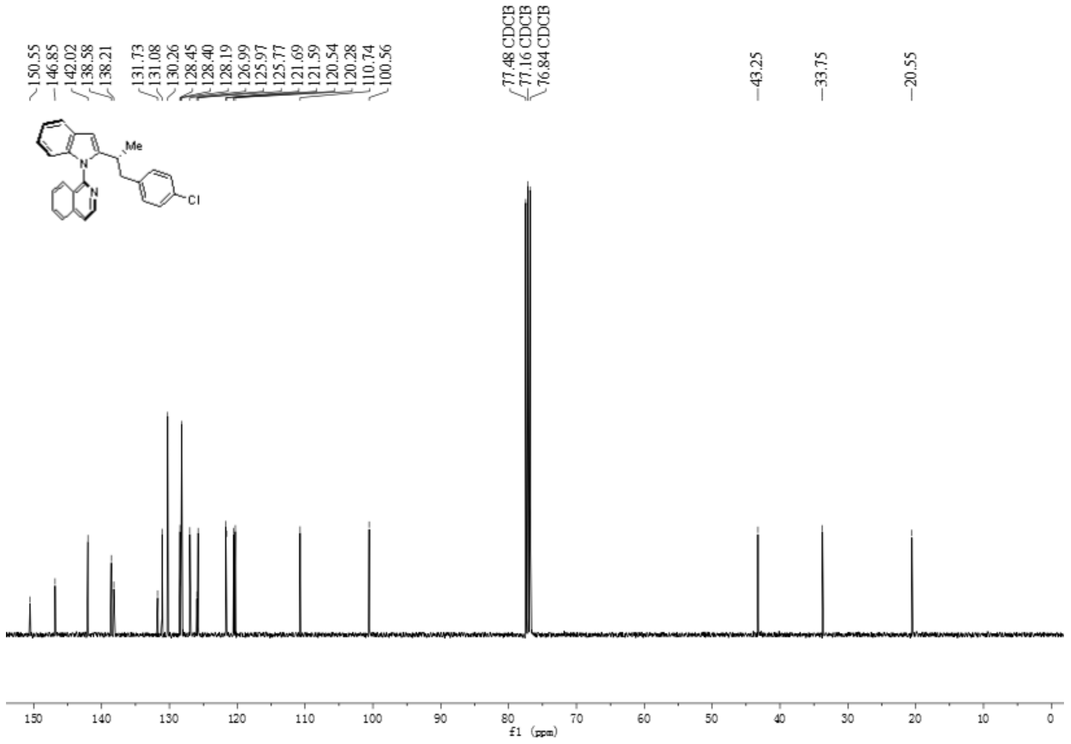

Supplementary Fig. 93. ^{1}H NMR spectrum of **3n**

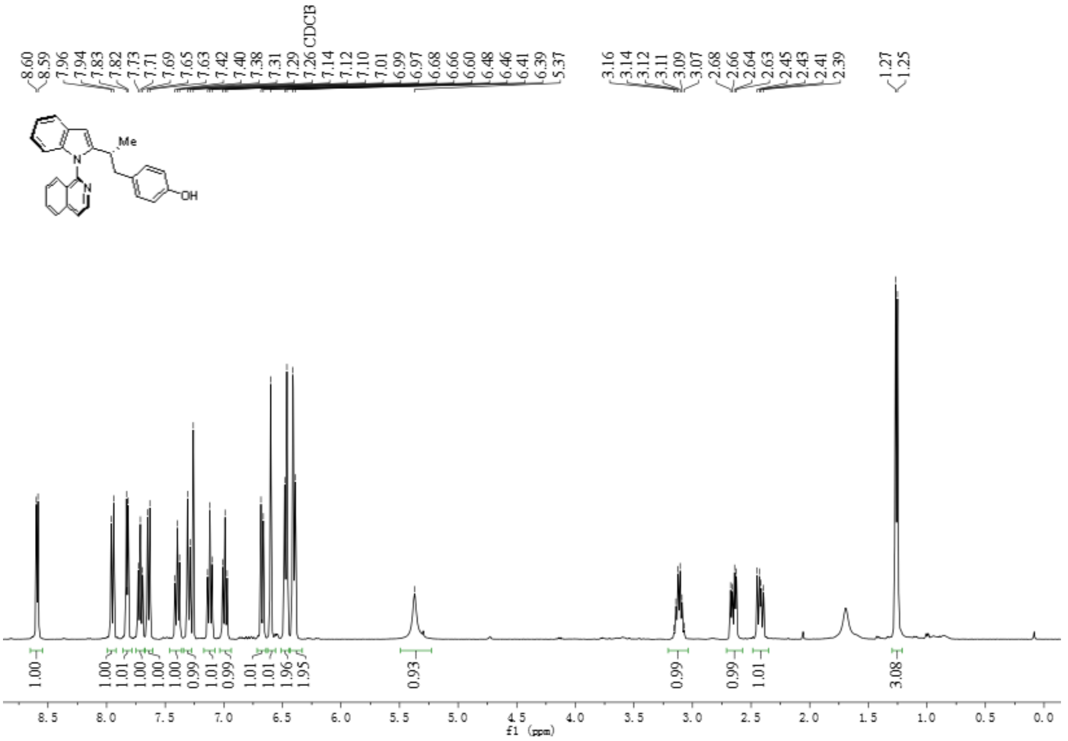

Supplementary Fig. 94. ^{13}C NMR spectrum of **3n**

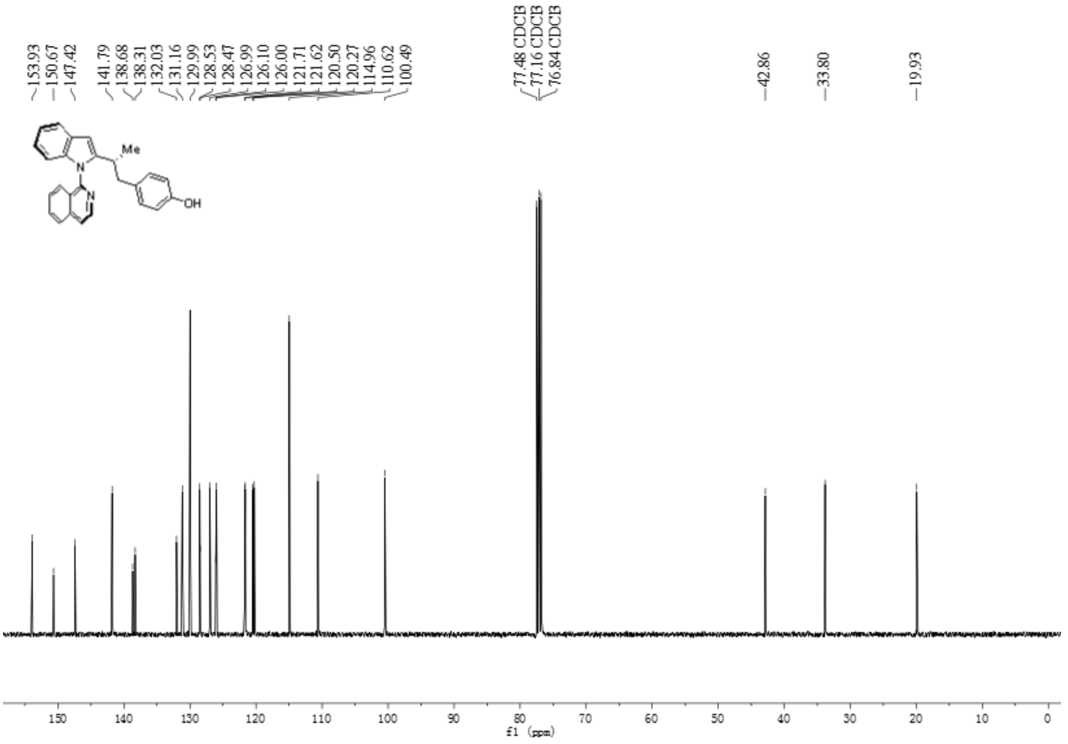

Supplementary Fig. 95. ^{1}H NMR spectrum of **3o**

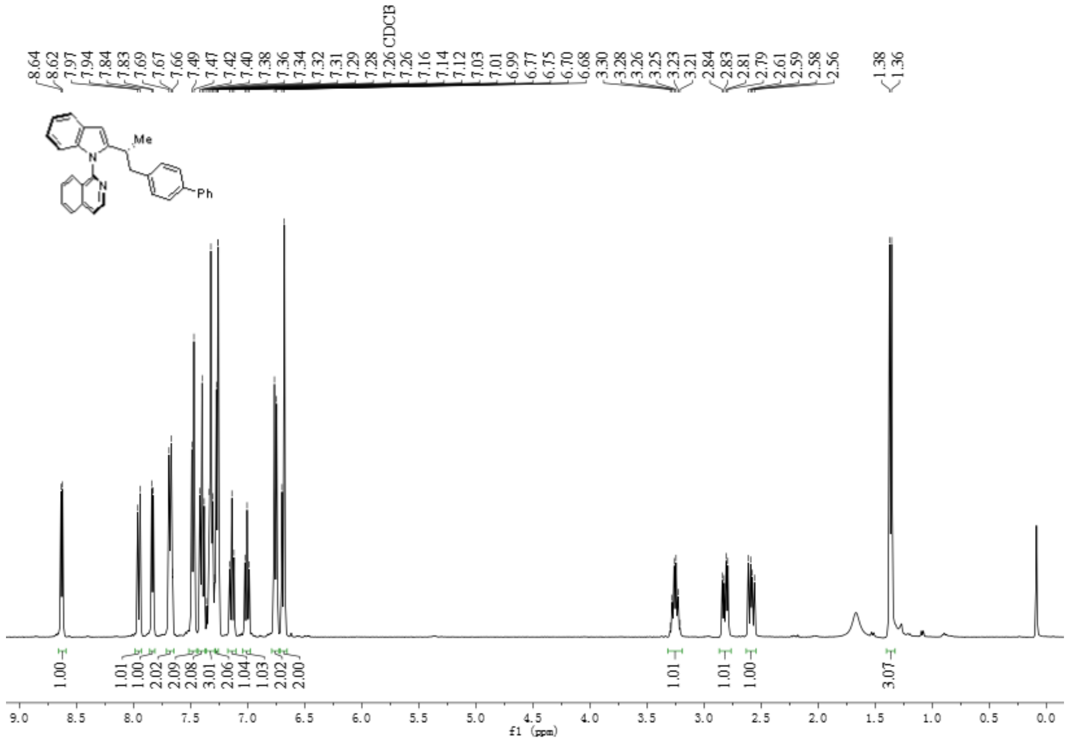

Supplementary Fig. 96. ^{13}C NMR spectrum of **3o**

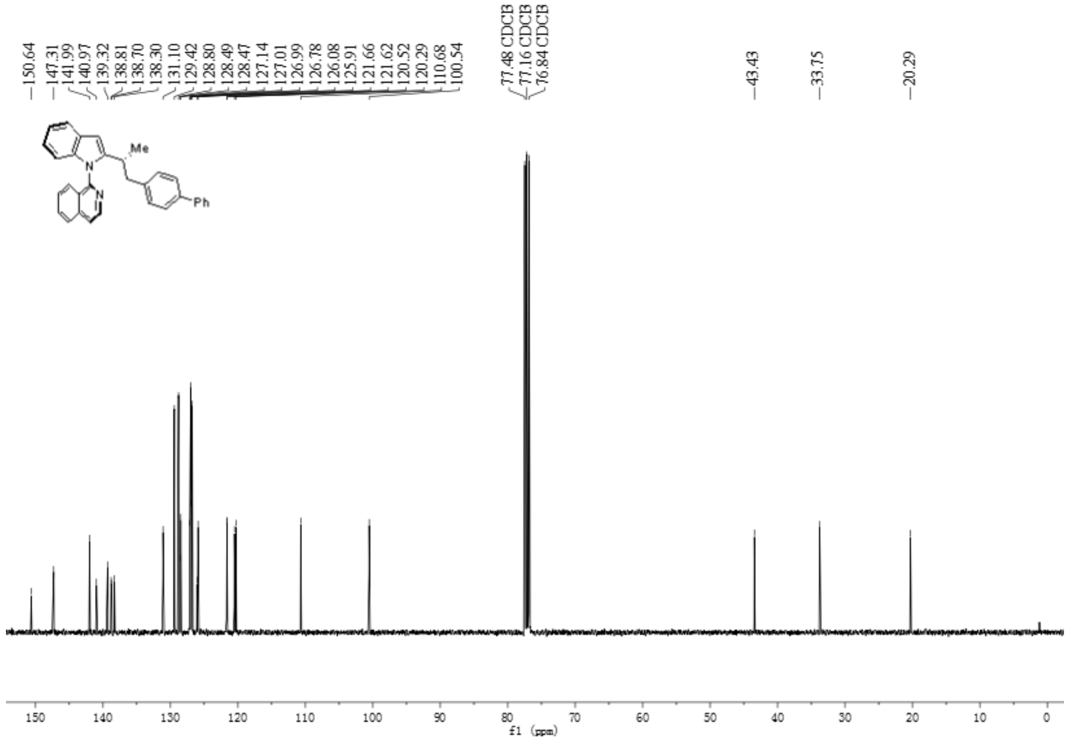

Supplementary Fig. 97. ^{1}H NMR spectrum of **3p**

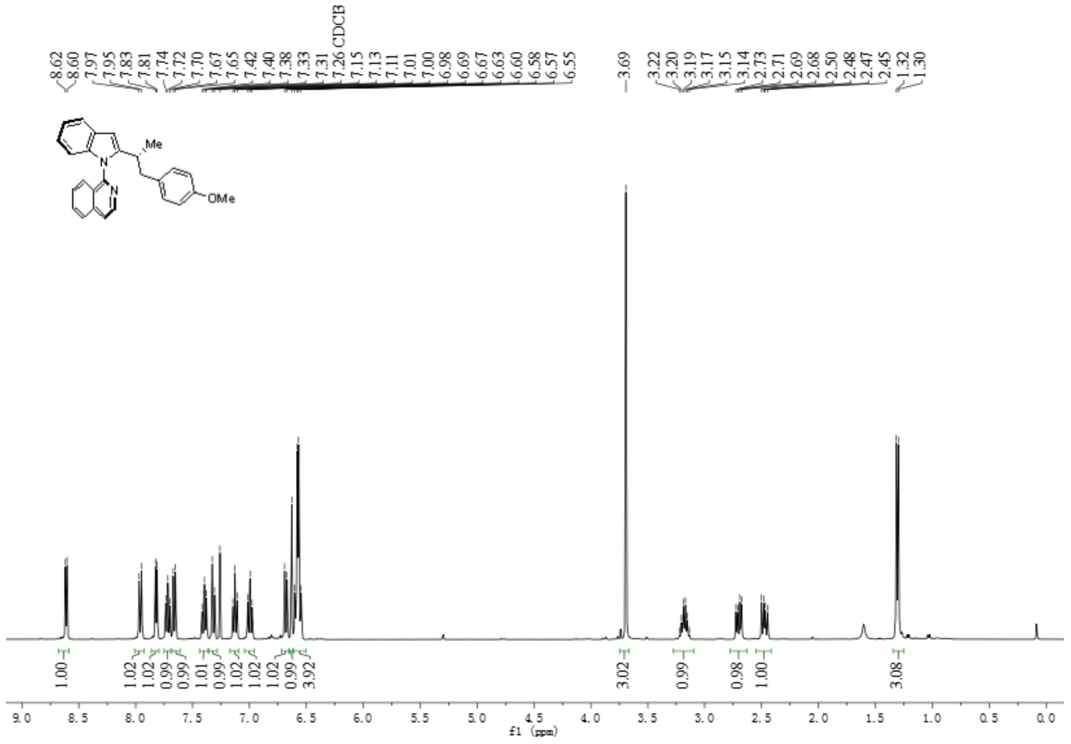

Supplementary Fig. 98. ^{13}C NMR spectrum of **3p**

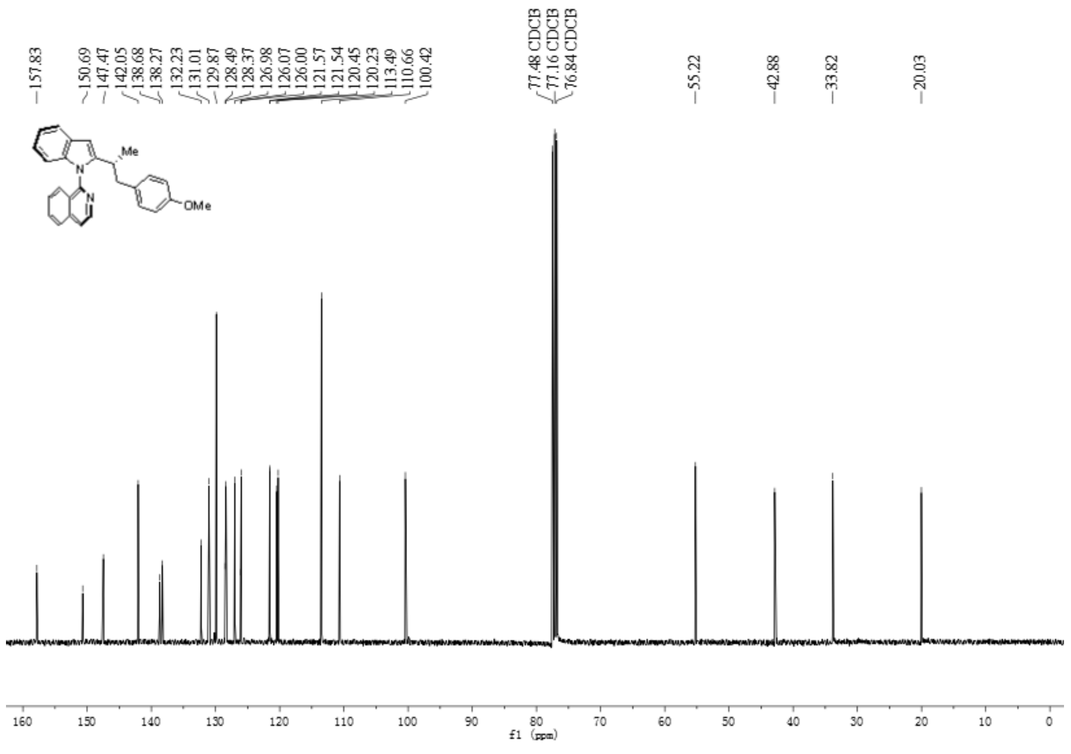

Supplementary Fig. 99. ^{1}H NMR spectrum of **3q**

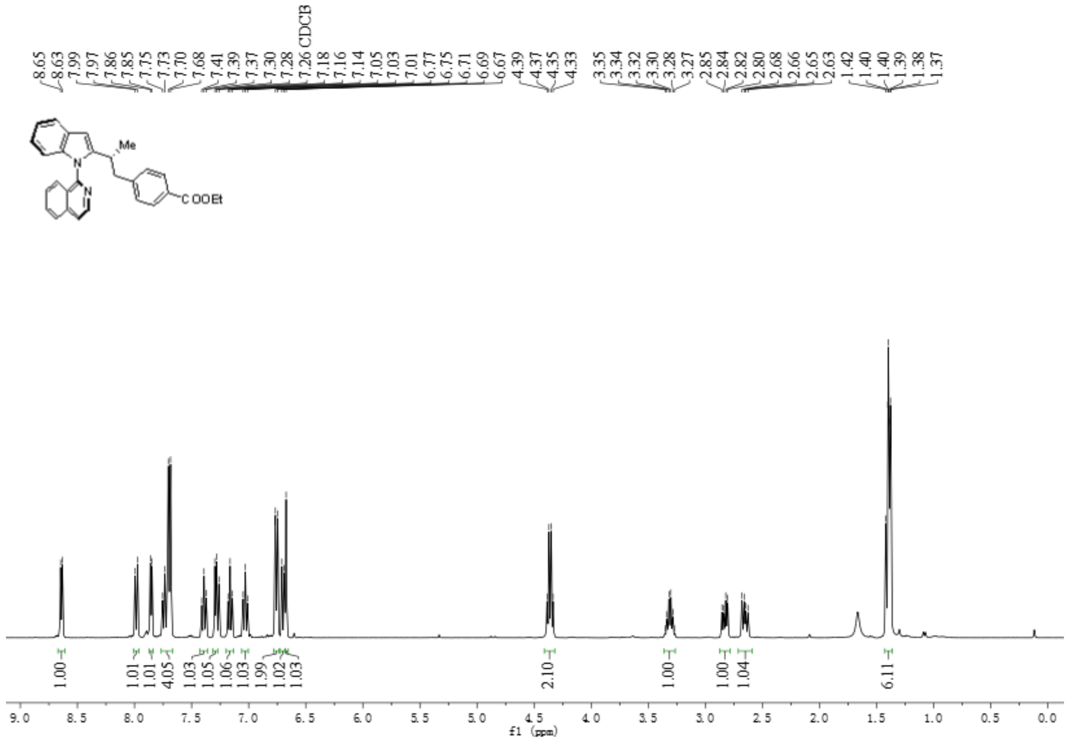

Supplementary Fig. 100. ^{13}C NMR spectrum of **3q**

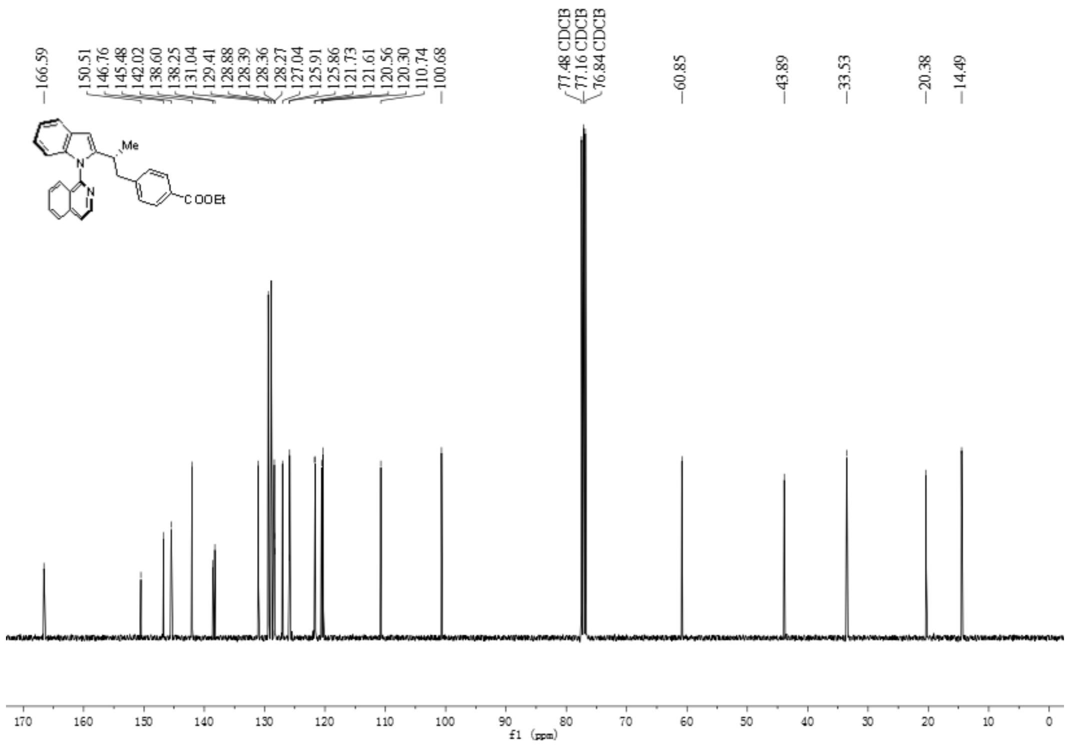

Supplementary Fig. 101. ^{1}H NMR spectrum of **3r**

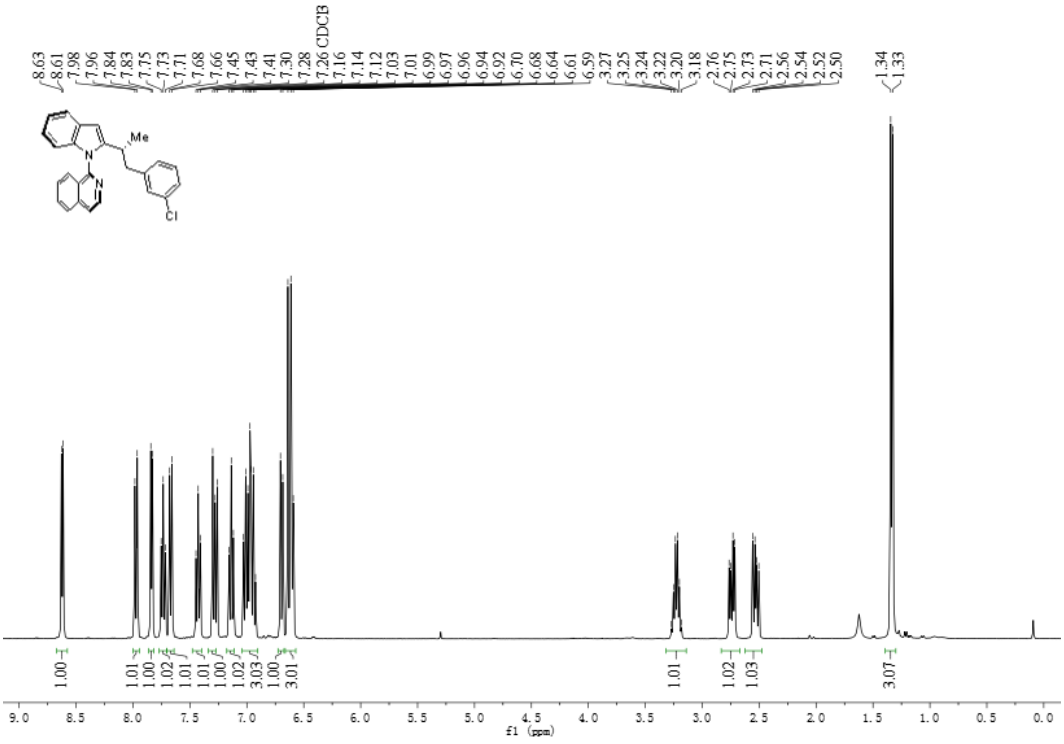

Supplementary Fig. 102. ^{13}C NMR spectrum of **3r**

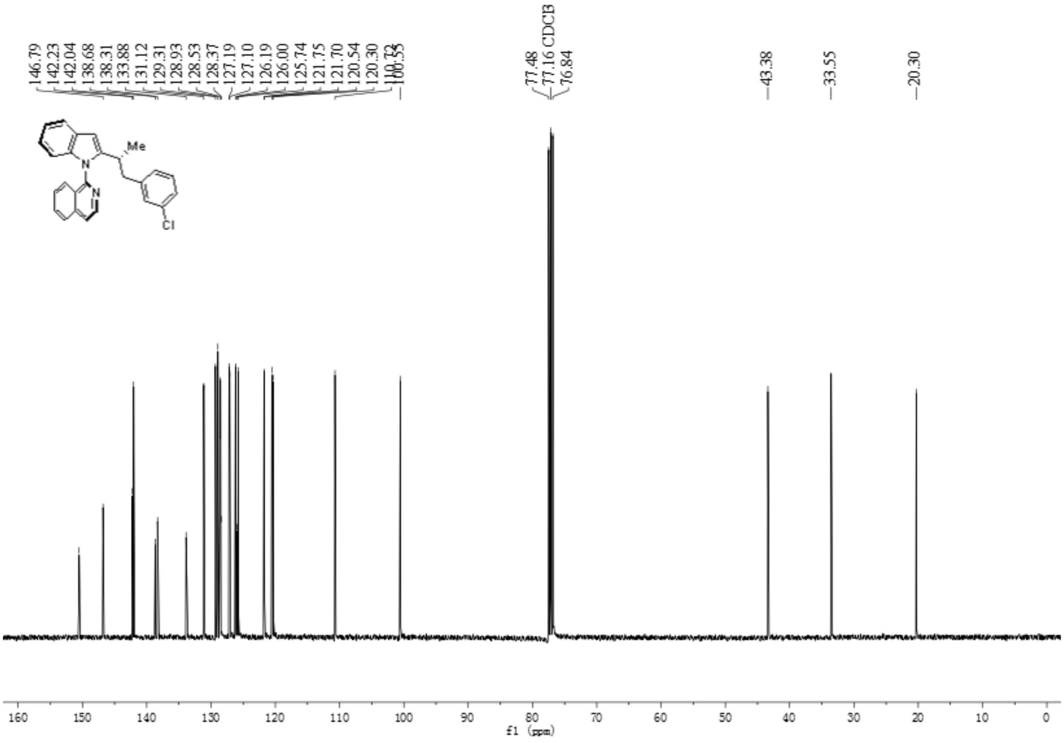

Supplementary Fig. 103. ^{1}H NMR spectrum of **3s**

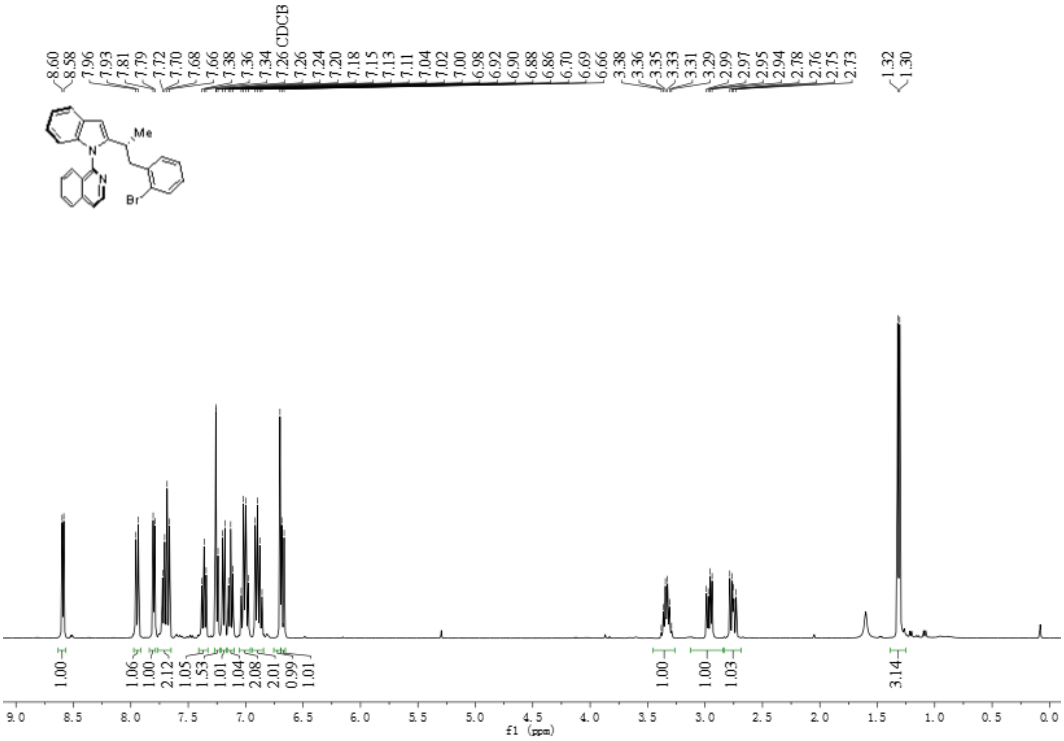

Supplementary Fig. 104. ^{13}C NMR spectrum of **3s**

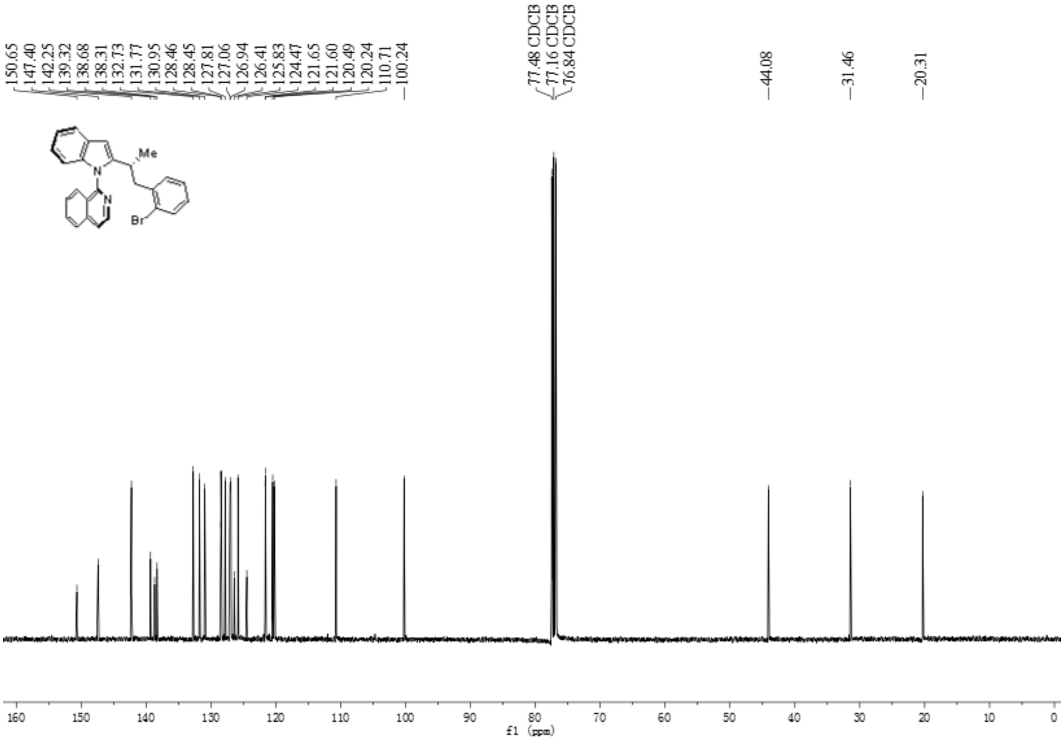

Supplementary Fig. 105. ^{1}H NMR spectrum of **3t**

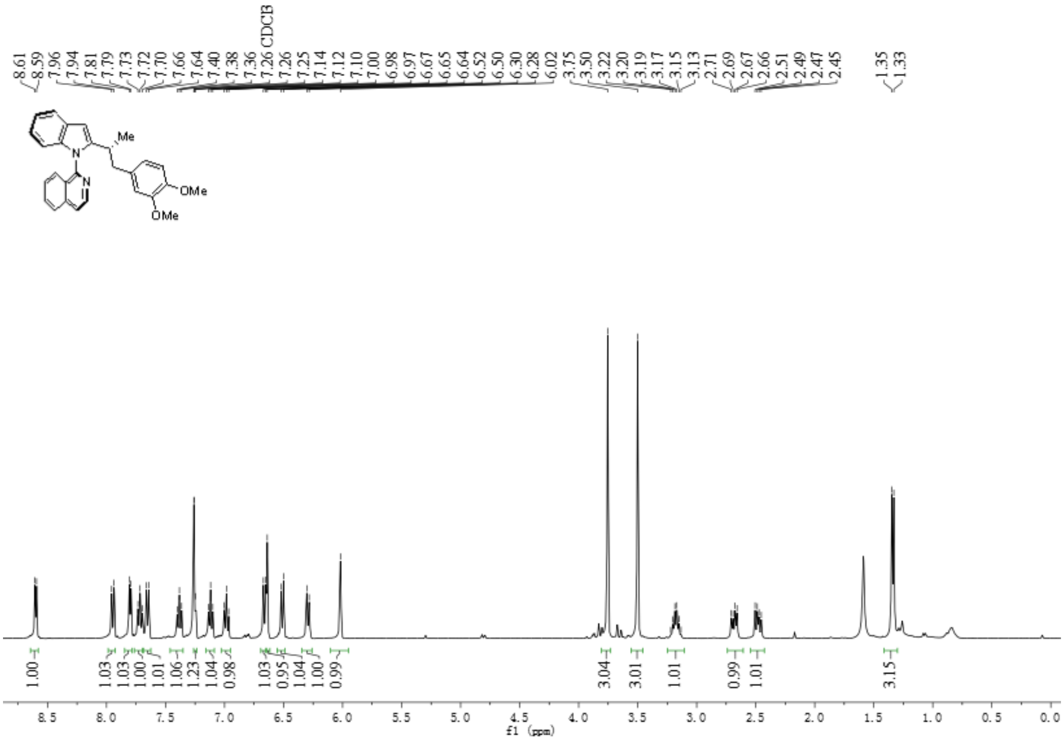

Supplementary Fig. 106. ^{13}C NMR spectrum of **3t**

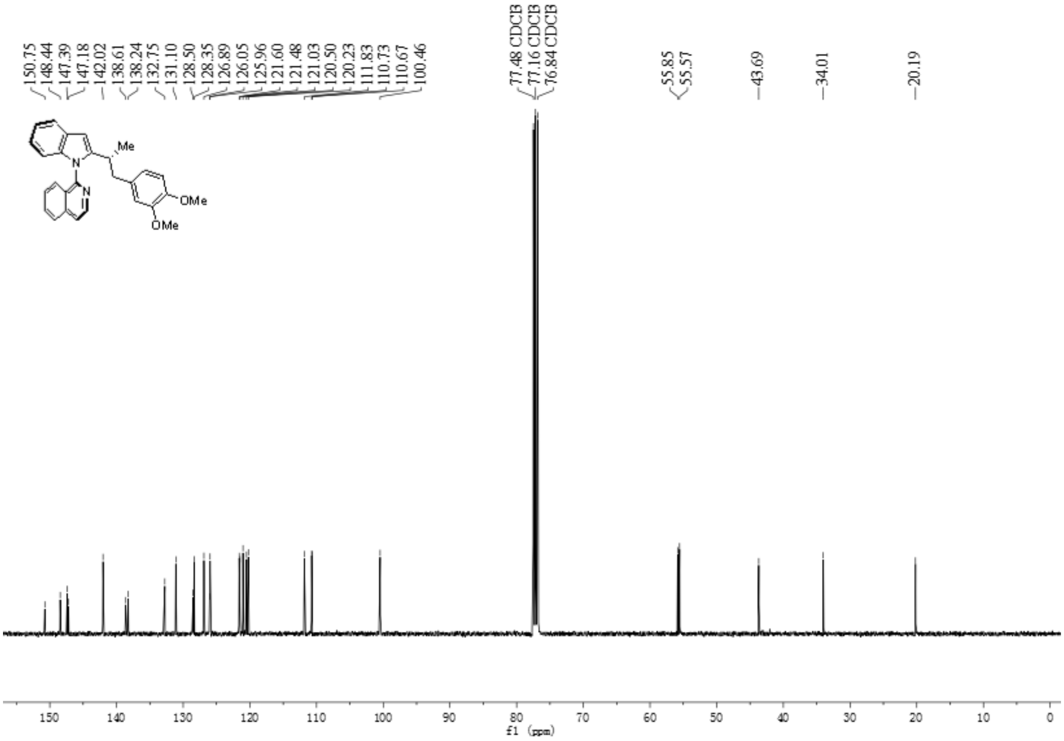

Supplementary Fig. 107. ^{1}H NMR spectrum of **3u**

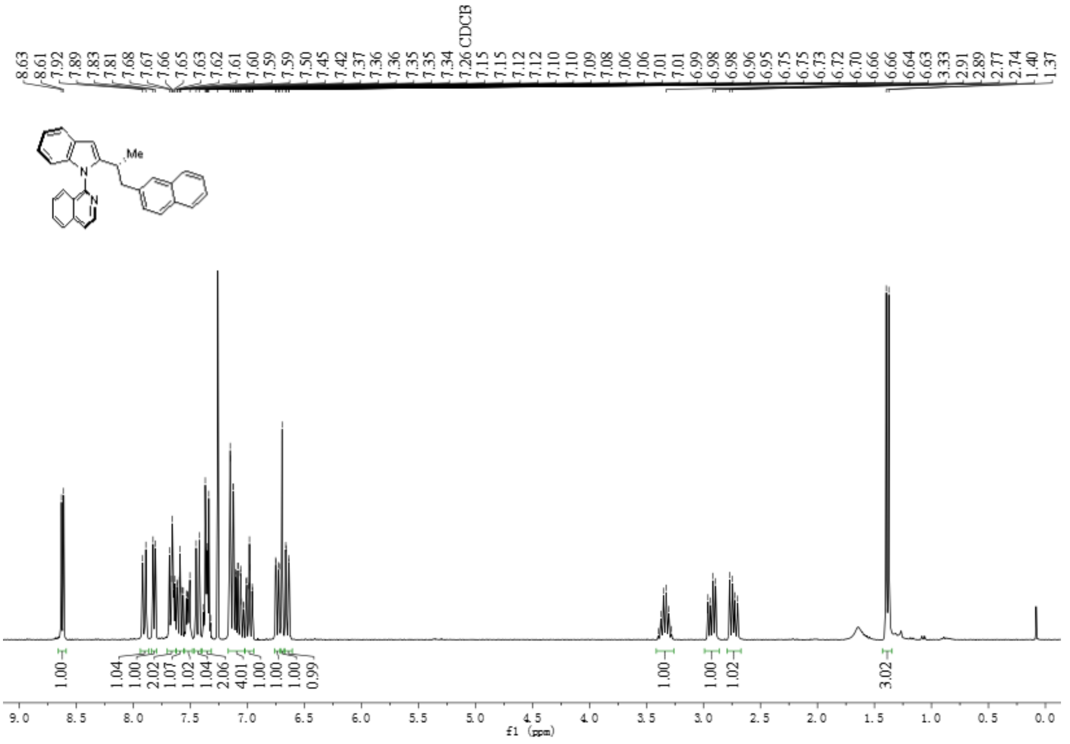

Supplementary Fig. 108. ^{13}C NMR spectrum of **3u**

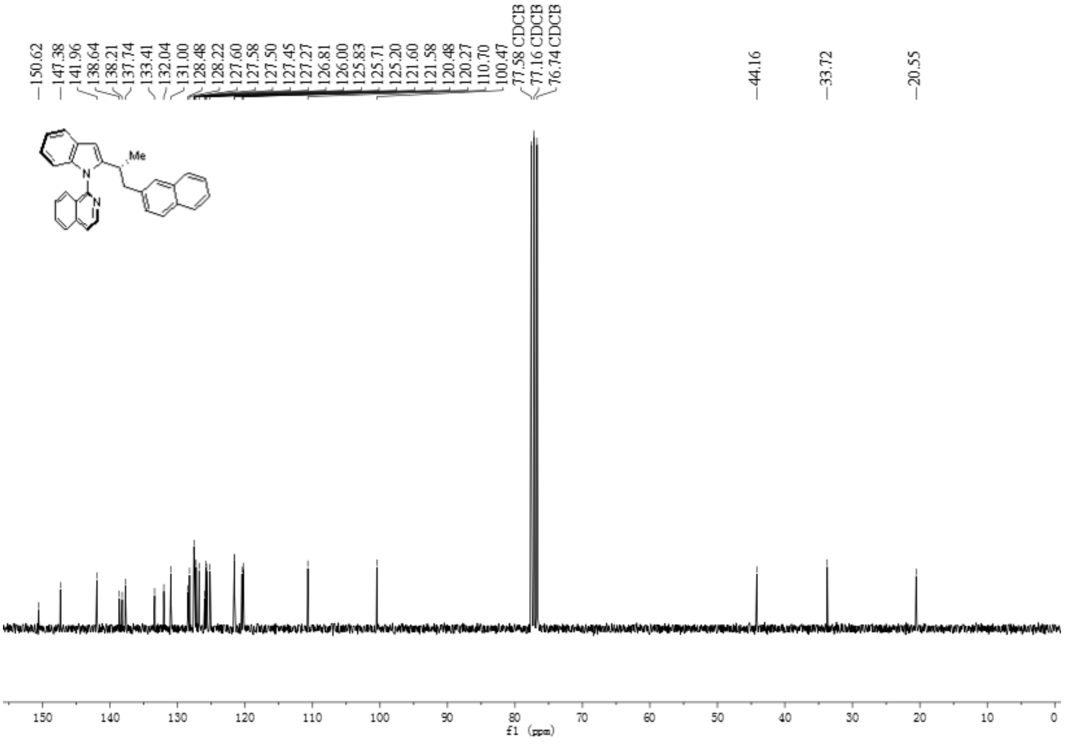

Supplementary Fig. 109. ^{1}H NMR spectrum of **3v**

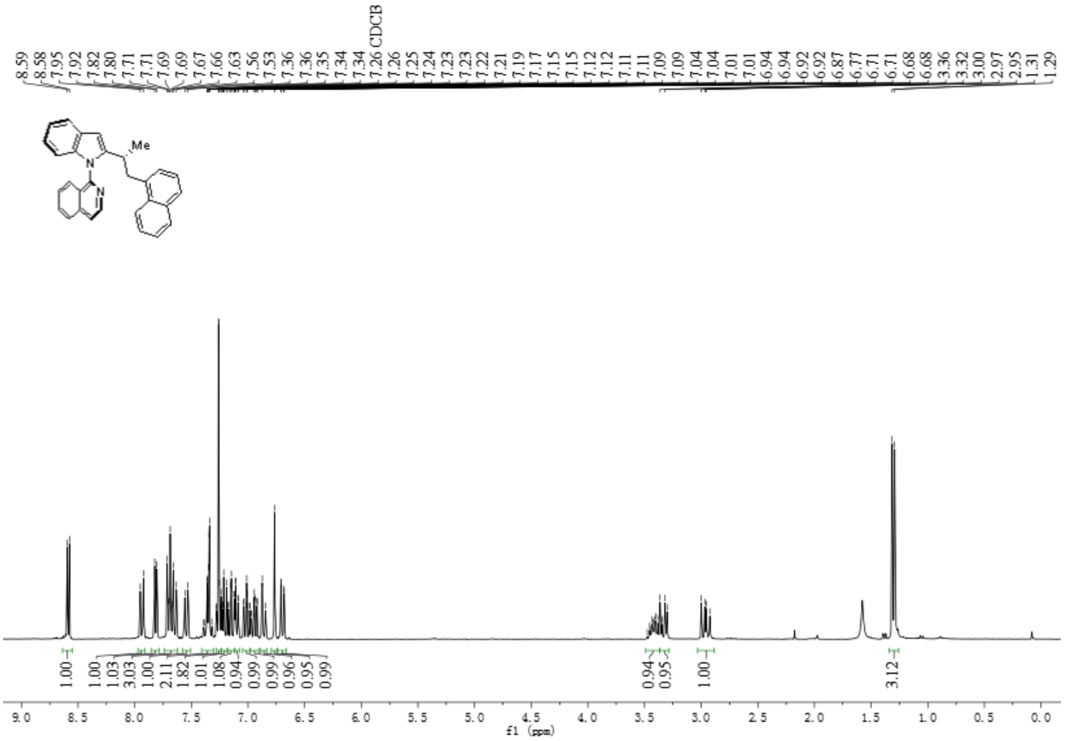

Supplementary Fig. 110. ^{13}C NMR spectrum of **3v**

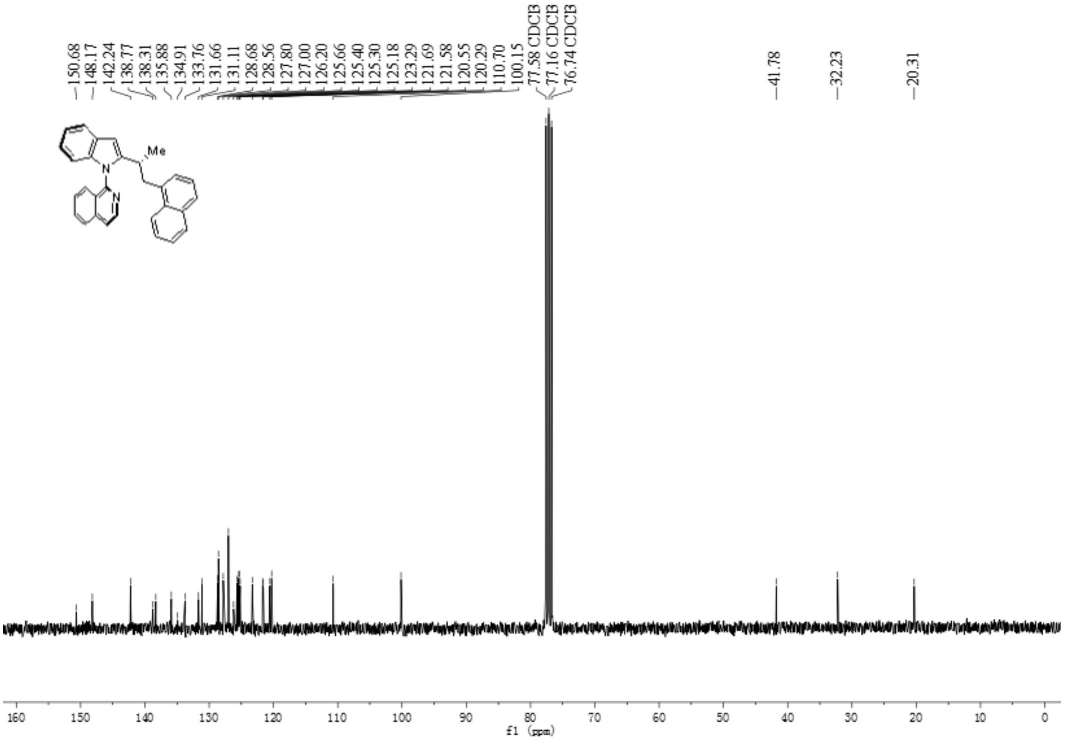

Supplementary Fig. 111. ^{1}H NMR spectrum of **3w**

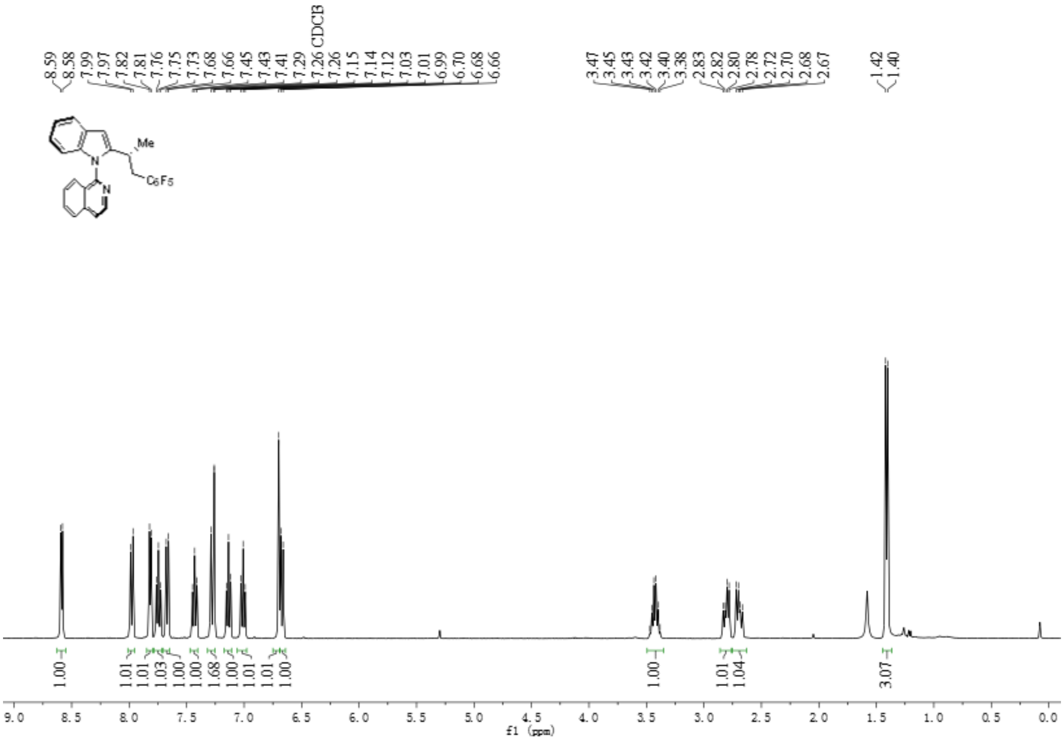

Supplementary Fig. 112. ^{13}C NMR spectrum of **3w**

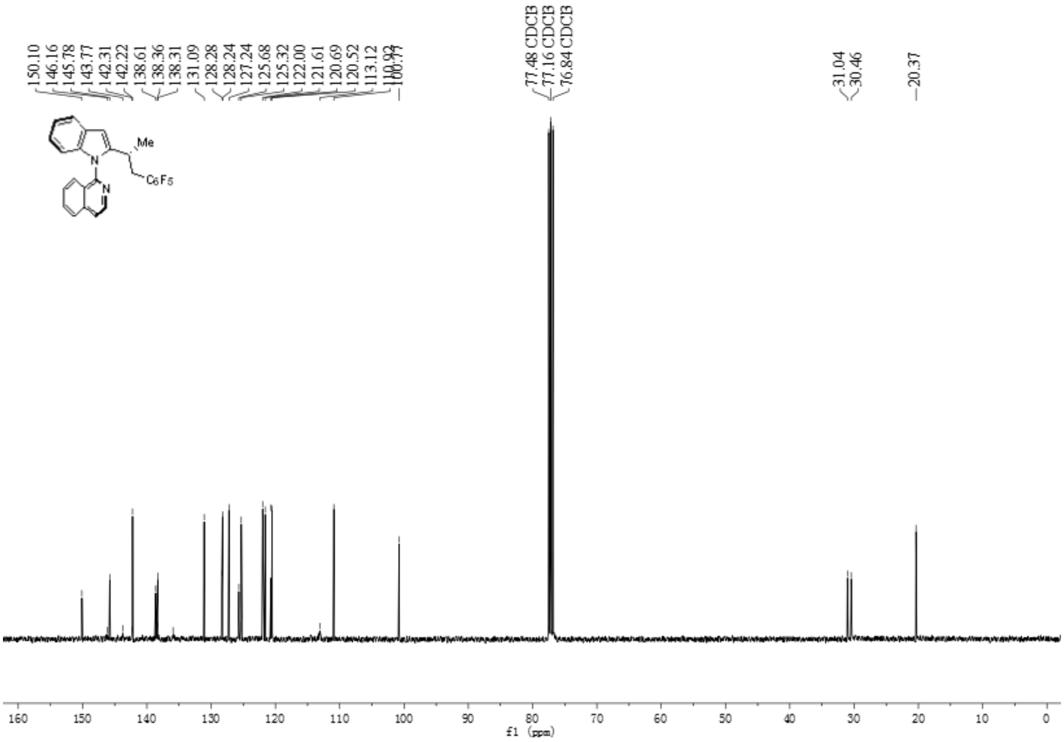

Supplementary Fig. 113. ^{19}F NMR spectrum of **3w**

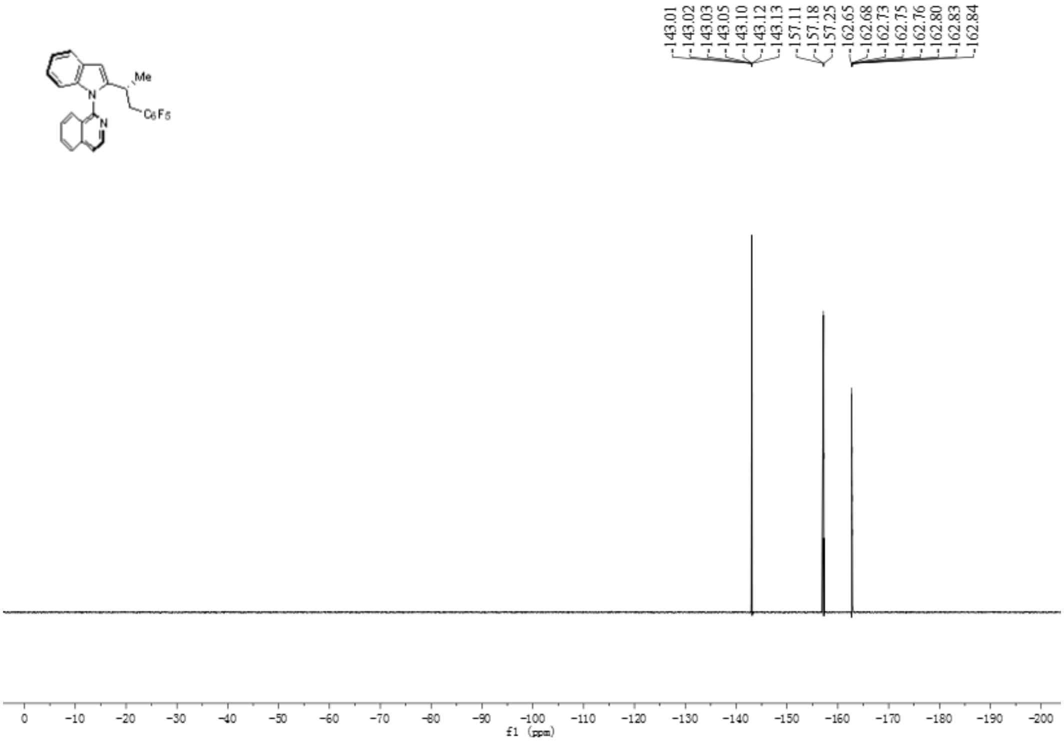

18. References

1. Pesciaioli, F. et al. Enantioselective cobalt(III)-catalyzed C–H activation enabled by chiral carboxylic acid cooperation. *Angew. Chem. Int. Ed.* **57**, 15425-15429 (2018).
2. Dhawa, U., Connon, R., Oliveira, J. C. A., Steinbock, R. & Ackermann, L. Enantioselective ruthenium-catalyzed C–H alkylations by a chiral carboxylic acid with attractive dispersive interactions. *Org. Lett.* **23**, 2760-2765 (2021).
3. Li, Y., Liou, Y.-C., Oliveira, J. C. A. & Ackermann, L. Ruthenium(II)/imidazolidine carboxylic acid-catalyzed C−H alkylation for central and axial double enantio induction. *Angew. Chem. Int. Ed.* **61**, e202212595 (2022).
4. Tiwari, V. K., Kamal, N. & Kapur, M. Ruthenium-catalyzed heteroatom-directed regioselective C–H arylation of indoles using a removable tether. *Org. Lett.* **17**, 1766-1769 (2015).
5. Whyte, A. et al. Cobalt-catalyzed enantioselective hydroarylation of 1,6-enynes. *J. Am. Chem. Soc.* **142**, 9510-9517 (2020).
6. RDKit: Open-source cheminformatics, http://www.rdkit.org.
7. Riniker, S. & Landrum, G. A. Better Informed Distance Geometry: Using What We Know To Improve Conformation Generation. *J. Chem. Inf. Model.* **55**, 2562-2574 (2015).
8. Bannwarth, C. et al. Extended tight-binding quantum chemistry methods. *WIREs Comput. Mol. Sci.* **11**, e1493 (2021).
9. Grimme, S., Bannwarth, C. & Shushkov, P. A Robust and Accurate Tight-Binding Quantum Chemical Method for Structures, Vibrational Frequencies, and Noncovalent Interactions of Large Molecular Systems Parametrized for All spd-Block Elements (Z = 1-86). *J. Chem. Theory Comput.* **13**, 1989-2009 (2017).
10. Molecular features for machine learning. https://kjelljorner.github.io/morfeus/.
11. Skurichina, M. & Duin, R. P. W. Bagging for linear classifiers. *Mach. Learn.* **24**, 123-140 (1996).
12. Breiman, L., Friedman, J., Olshen, R. & Stone, C. Classification and regression trees. *Encyclopedia of Ecology* **57**, 582-588 (2015).
13. Geurts, P., Ernst, D. & Wehenkel, L. Extremely randomized trees. *Mach. Learn.* **63**, 3-42 (2006).
14. Jerome, H. F. Greedy function approximation: A gradient boosting machine. *Ann. Stat.* **29**,

1189-1232 (2001).

15. Fix, E. & Hodges, J. L. Discriminatory analysis. Nonparametric discrimination: Consistency properties. *Int. Stat. Rev.* **57**, 238-247 (1989).
16. Cawley, G. C. & Talbot, N. L. C. Reduced rank kernel ridge regression. *Neural Processing Lett.* **16**, 293-302 (2002).
17. Cortes, C. & Vapnik, V. Support-vector networks. *Mach. Learn.* **20**, 273-297 (1995).
18. Biau, G. Analysis of a random forests model. *J. Mach. Learn. Res.* **13**, 1063-1095 (2012).
19. Garcia, C. B., Garcia J., Martin M. M. L. & Salmeron R. Collinearity: Revisiting the variance inflation factor in ridge regression. *J. Appl. Stat.* **42**, 648-661 (2015).
20. Zhang, Y. & Chen, L. A study on forecasting the default risk of bond based on xgboost algorithm and over-sampling method. *Theor. Econ. Lett.* **11**, 258-267 (2021).
21. Pedregosa, F. et al. Scikit-learn: Machine learning in python. Preprint at https://www.semanticscholar.org/paper/Scikit-learn%3A-Machine-Learning-in-Python-Pedregosa-Varoquaux/168f28ac3c8c7ea63bf7ed25f2288e8b67e2fe74 (2018).
22. Xgb: Scalable and flexible gradient boosting. https://xgboost.ai/.
